# PIK3CA inhibition in models of proliferative glomerulonephritis and lupus nephritis

**DOI:** 10.1172/JCI176402

**Published:** 2024-06-06

**Authors:** Junna Yamaguchi, Pierre Isnard, Noémie Robil, Pierre de la Grange, Clément Hoguin, Alain Schmitt, Aurélie Hummel, Jérôme Megret, Nicolas Goudin, Marine Luka, Mickaël M. Ménager, Cécile Masson, Mohammed Zarhrate, Christine Bôle-Feysot, Michalina Janiszewska, Kornelia Polyak, Julien Dairou, Sara Baldassari, Stéphanie Baulac, Christine Broissand, Christophe Legendre, Fabiola Terzi, Guillaume Canaud

**Affiliations:** 1 Université Paris Cité, Paris, France.; 2 Unité de Médecine Translationnelle et Thérapies Ciblées, Hôpital Necker-Enfants Malades, Assistance Publique-Hôpitaux de Paris (AP-HP), Paris, France.; 3 INSERM U1151, Institut Necker-Enfants Malades, Paris, France.; 4 Service d’Anatomie pathologique, Hôpital Necker-Enfants Malades, AP-HP, Paris, France.; 5 Genosplice Technology, Paris Biotech Santé, Paris, France.; 6 Institut Cochin, Paris, France.; 7 Service de Néphrologie, Transplantation Adultes, Hôpital Necker-Enfants Malades, AP-HP, Paris, France.; 8 Structure Fédérative de Recherche Necker, INSERM US24, CNRS UAR 3633, Institut Necker-Enfants Malades, Paris, France.; 9 Inflammatory Responses and Transcriptomic Networks in Diseases,; 10 INSERM U1163,; 11 Bioinformatics Platform, Structure Fédérative de Recherche Necker, INSERM UMR1163, Université de Paris, and; 12 Plateforme Génomique, INSERM U1163, Institut Imagine, Paris, France.; 13 Department of Molecular Medicine, The Herbert Wertheim UF Scripps Institute for Biomedical Innovation and Technologies, Jupiter, Florida, USA.; 14 Department of Medical Oncology, Dana-Farber Cancer Institute, Boston, Massachusetts, USA.; 15 Department of Medicine, Harvard Medical School, Boston, Massachusetts, USA.; 16 Laboratoire de Chimie et Biologie Pharmacologiques et Toxicologiques, Paris, France.; 17 Sorbonne Université, Institut du Cerveau - Paris Brain Institute - ICM, Inserm, CNRS, Hôpital de la Pitié Salpêtrière, Paris, France.; 18 Pharmacie, Hôpital Necker-Enfants Malades, AP-HP, Paris, France.

**Keywords:** Nephrology, Molecular biology

## Abstract

Proliferative glomerulonephritis is a severe condition that often leads to kidney failure. There is a significant lack of effective treatment for these disorders. Here, following the identification of a somatic *PIK3CA* gain-of-function mutation in podocytes of a patient, we demonstrate using multiple genetically engineered mouse models, single-cell RNA sequencing, and spatial transcriptomics the crucial role played by this pathway for proliferative glomerulonephritis development by promoting podocyte proliferation, dedifferentiation, and inflammation. Additionally, we show that alpelisib, a PI3Kα inhibitor, improves glomerular lesions and kidney function in different mouse models of proliferative glomerulonephritis and lupus nephritis by targeting podocytes. Surprisingly, we determined that pharmacological inhibition of PI3Kα affects B and T lymphocyte populations in lupus nephritis mouse models, with a decrease in the production of proinflammatory cytokines, autoantibodies, and glomerular complement deposition, which are all characteristic features of PI3Kδ inhibition, the primary PI3K isoform expressed in lymphocytes. Importantly, PI3Kα inhibition does not impact lymphocyte function under normal conditions. These findings were then confirmed in human lymphocytes isolated from patients with active lupus nephritis. In conclusion, we demonstrate the major role played by PI3Kα in proliferative glomerulonephritis and show that in this condition, alpelisib acts on both podocytes and the immune system.

## Abstract 2

The PI3Kα pathway plays a pivotal role in the development of proliferative glomerulonephritis, providing a potential pharmacological target.

## Graphical abstract

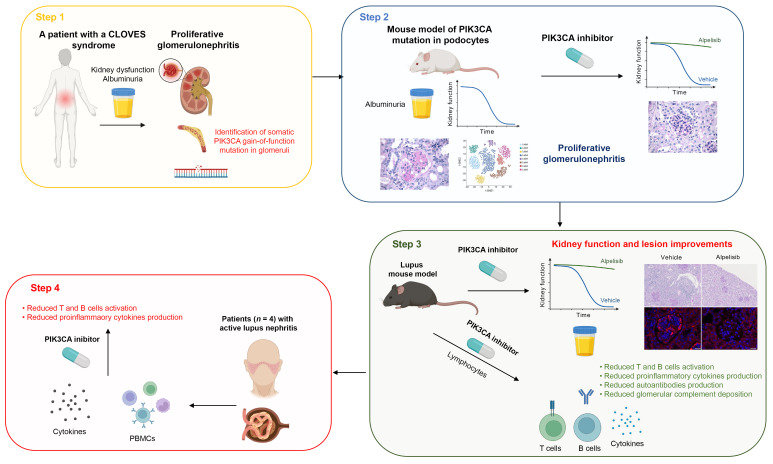

## Introduction

PI3Kα is a lipid kinase that is widely expressed in various tissues and plays a crucial role in controlling signaling pathways involved in cell proliferation, motility, survival, and metabolism (1). The *PIK3CA* gene codes for the α subunit of PI3K (p110α), which has a molecular weight of 110 kDa. Following its activation through tyrosine kinase receptors, p110α converts phosphatidylinositol 4,5-bisphosphate (PtdIns[4,5]P_2_) to phosphatidylinositol 3,4,5-trisphosphate (PtdIns[3,4,5]P_3_ or PIP_3_) at the plasma membrane. This conversion leads to the recruitment of PDK1, which phosphorylates AKT on Thr^308^ and initiates downstream cellular effects (1). Additionally, PI3Kα regulates various other pathways, including the Rho/Rac1 signaling cascade (2).

Somatic gain-of-function mutations in the *PIK3CA* gene have been described in patients and are associated with *PIK3CA*-related overgrowth syndrome (PROS) (3, 4). Individuals with PROS typically exhibit complex tissue malformations such as abnormal vessels, disorganized adipose tissue, muscle hypertrophy, and bone deformations (5–10). In previous research, we identified alpelisib, a PI3Kα inhibitor, as a promising treatment option and demonstrated its effectiveness in both mouse models and patients with PROS (11). These findings were further validated in the EPIK P1 clinical trial (ClinicalTrials.gov NCT04285723) (12), which led to the recent accelerated approval of alpelisib by the US FDA for PROS patients above 2 years of age.

In our initial report, the first PROS patient (referred to as patient 1) had kidney dysfunction with nephrotic-range proteinuria before beginning alpelisib treatment (11). The patient was carrying a somatic *PIK3CA* p.H1047R variant that had been previously identified in a skin biopsy (c.3140 A>G; COSMIC genomic mutation ID: 55873195) (11). The kidney disease of patient 1 occurred in a complex medical context involving severe vascular malformations (including a combination of vein and lymphatic anomalies as well as arteriovenous shunts), paraplegia with vesicoureteral reflux, severe congestive heart failure, and the use of rapamycin (an mTOR inhibitor) (13). While administering alpelisib to address the patient’s overall condition, we noticed an improvement in both kidney function and proteinuria (11). This prompted us to investigate the role of PI3Kα in the patient’s kidney disease and ultimately identify this kinase as a promising target for addressing proliferative glomerulonephritis.

## Results

### A patient with PIK3CA gain-of-function mutation in podocytes.

**Figure 1:**
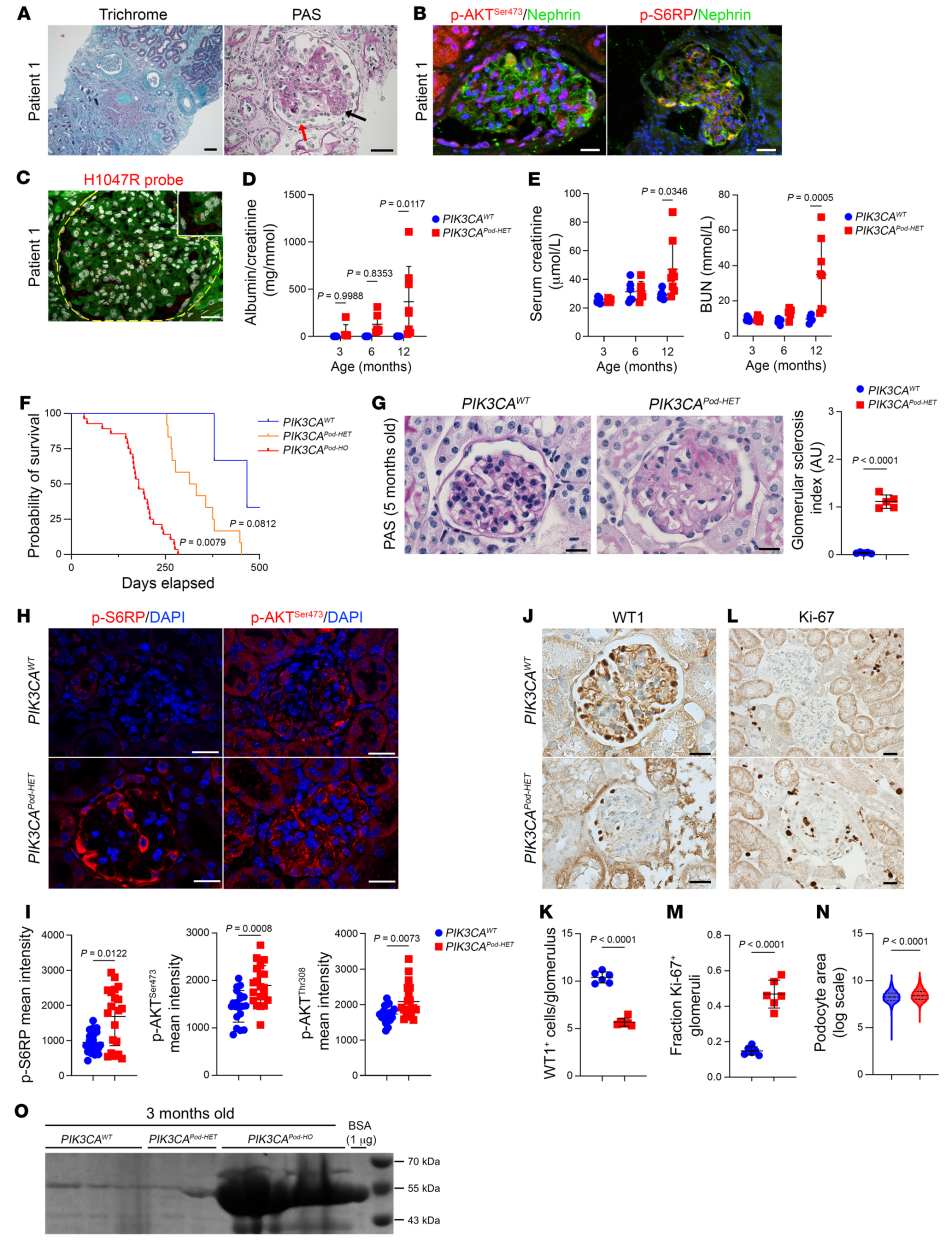
*PIK3CA* gain-of-function mutation in podocytes leads to severe glomerular disease. (**A**–**C**) Patient 1 kidney. (**A**) Masson’s trichrome and PAS staining. Black arrow shows hypercellularity. Red arrow shows hypertrophy and hyperplasia of the overlying podocytes. (**B**) p-AKT^Ser473^ or p-S6RP coimmunofluorescent staining with nephrin. (**C**) In situ *PIK3CA* *H1047R* hybridization. The yellow dashed line outlines the glomerulus. Red dots are mutation-positive points. (**D** and **E**) Urinary albumin/creatinine ratio, serum creatinine, and blood urea nitrogen (BUN) of *PIK3CA^WT^* and *PIK3CA^Pod-HET^* mice with aging. Three-month-old *PIK3CA^WT^*, *n =* 5; 3-month-old *PIK3CA^HET^*, *n =* 6; 6-month-old *PIK3CA^WT^*, *n =* 6; 6-month-old *PIK3CA^HET^*, *n =* 6; 12-month-old *PIK3CA^WT^*, *n =* 6; 12-month-old *PIK3CA^HET^*, *n =* 8. (**F**) Kaplan-Meier curves of *PIK3CA^WT^* (*n =* 27), *PIK3CA^Pod-HET^* (*n =* 12), and *PIK3CA^Pod-HO^* mice (*n =* 28). (**G**–**M**) Five-month-old *PIK3CA^WT^* and *PIK3CA^Pod-HET^* mouse kidneys. (**G**) PAS and glomerular sclerosis (GS) index quantification (*n =* 5 mice per group). (**H**) p-S6RP and p-AKT^Ser473^ immunofluorescent staining. (**I**) Glomerular p-S6RP, p-AKT^Ser473^, and p-AKT^Thr308^ quantification (*n =* 5 mice per group). (**J**–**M**) Representative WT1 or Ki-67 immunostaining and quantification (*n =* 6 mice per group). (**N**) Amnis ImageStream analysis of podocytes of 4-month-old *PIK3CA^WT^* and *PIK3CA^Pod-HET^* mice (*n =* 3 per group). (**O**) Coomassie blue staining of *PIK3CA^WT^*, *PIK3CA^Pod-HET^*, and *PIK3CA^Pod-HO^* mice. Values are the mean ± SD. *P* values calculated using 2-way ANOVA with Tukey’s post hoc test (**D** and **E**), log-rank (Mantel-Cox) test (**F**), 2-tailed Mann-Whitney *U* test (**I**), or 2-tailed Student’s *t* test (**G**, **K**, **M**, and **N**). Scale bars: 100 μm (**A**, trichrome), 40 μm (**A**, PAS), 20 μm (**B**, **C**, and **H**), 25 μm (**G**), and 32.2 μm (**J** and **L**).

Patient 1 underwent a kidney biopsy prior to initiating alpelisib treatment, which revealed a complex glomerulonephritis characterized by focal and segmental glomerulosclerosis (FSGS), with podocyte hyperplasia and hypertrophy, collapsing aspect, and pseudocrescent formation (Figure 1A and Supplemental Figure 1A; supplemental material available online with this article; https://doi.org/10.1172/JCI176402DS1). Notably, there were no immune deposits observed. The biopsy also indicated extensive fibrosis (>80% of the parenchyma) accompanied by tubular dilation, casts, and infiltration of inflammatory cells (Figure 1A and Supplemental Figure 1A). Immunofluorescence studies showed activation of the AKT/mTOR pathway in podocytes (Figure 1B and Supplemental Figure 1B). Initially, we speculated that these lesions were a consequence of the patient’s complicated medical condition involving vesicoureteral reflux, congestive heart failure, and rapamycin usage. After discontinuing rapamycin, no improvement was observed regarding proteinuria or kidney function (11). As previously reported, because of the severity of the PROS condition, we were granted the authorization to administer alpelisib, which is an approved isoform-selective PI3Kα inhibitor (11). Treatment with this medication resulted in the reduction of different malformations and a complete recovery from congestive heart failure (11). Intriguingly, we observed improvement in the high amount of proteinuria and the stabilization of kidney function (11). This indicated that the patient may carry a *PI3KCA* mutation in the kidney epithelial cells. To investigate this possibility, we first performed in situ PCR using fluorescently labeled mismatched primers designed to specifically amplify the mutant that was previously identified in a skin biopsy using next-generation sequencing (11) (p.H1047R, c.3140 A>G; COSMIC genomic mutation ID: 55873195) and WT *PIK3CA* alleles on a paraffin-embedded kidney biopsy section (14). T-47D cells were employed as positive controls (Supplemental Figure 1C). We observed the presence of the *PIK3CA* mutant alleles in various types of glomerular cells, including cells evocative of podocytes (Figure 1C). Unfortunately, due to technical limitations, we were unable to perform coimmunostaining to specifically identify the affected cell types. To confirm the presence of the mutation, we performed droplet digital PCR (ddPCR) of glomeruli isolated from the kidney biopsy from patient 1 using laser capture microdissection (Supplemental Figure 1D). While control biopsies obtained from diabetic patients did not reveal any alteration in *PIK3CA* alleles, ddPCR of the glomeruli from patient 1 demonstrated the presence of the *PIK3CA H1047R* mutation at a mosaic fraction of 8% (Supplemental Figure 1E). We concluded that patient 1 was carrying the *PIK3CA* gain-of-function mutation in glomerular epithelial cells.

### PIK3CA gain-of-function mutation in podocytes causes progressive proliferative glomerulonephritis in mice.

Considering the glomerular profile of kidney dysfunction in patient 1, the presence of the *PIK3CA* variant in glomerular cells, and the substantial decrease in proteinuria upon the introduction of alpelisib, we decided to create a mouse model of the *PIK3CA* gain-of-function mutation within podocytes. We took advantage of the transgenic mouse strain, R26StopFLP110*, which after breeding with Cre recombinase mice expresses a dominant active *PI3KCA* transgene (11). R26StopFLP110* mice were crossed with *Podocin*-Cre mice to generate *PIK3CA^Pod^* animals that expressed the overactivated form of PIK3CA. To follow Cre recombination, *PIK3CA^Pod^* mice were interbred with *Gt(ROSA)26Sor^tm4(ACTB-tdTomato,-EGFP)Luo^*/J mice to create *PIK3CA^Pod-HET^* mice (15). These mice express a cell membrane–localized, fluorescent tdTomato protein in all tissues that is replaced by GFP after Cre recombination. At birth, *PIK3CA^Pod-HET^* mice were indistinguishable from WT control littermates (referred to hereafter as *PIK3CA^WT^*). However, *PIK3CA^Pod-HET^* mice progressively developed albuminuria after 3 months of age, with slowly declining kidney function (Figure 1, D and E, and Supplemental Figure 1F) and reduced survival (Figure 1F). Compared with the controls, starting at the age of 3 months, *PIK3CA^Pod-HET^* mice demonstrated glomerular lesion–mixing pseudocrescent formation with cellular proliferation, such as that observed in patient 1 (Figure 1, A and G, and Supplemental Figure 1G). Optical microscopy revealed hypertrophic podocytes with intracytoplasmic vacuoles as well as protein casts and infiltration of inflammatory cells (Figure 1G and Supplemental Figure 1, G and H). Costaining for nephrin and CD44 revealed progressive and extensive formation of crescentic lesions in glomeruli from *PIK3CA^Pod-HET^* mice compared with controls (Supplemental Figure 1I). Crescents were visible starting from the age of 5 months and worsened with age (Supplemental Figure 1, I and J). Transmission electron microscopy showed swollen hypertrophic podocytes with microvillous transformation, lipid vacuolization, and focal foot process effacement (Supplemental Figure 2A). The glomerular basement membrane demonstrated thickening and wrinkling, typical features of FSGS lesions and more specifically collapsing glomerulopathy (Supplemental Figure 2A). Immunofluorescence experiments confirmed the activation of the AKT/mTOR pathway specifically in podocytes of *PIK3CA^Pod-HET^* mice (Figure 1, H and I, and Supplemental Figure 2B). One of the main characteristics of podocyte injury is the loss of differentiation markers. We decided to study cellular differentiation by analyzing the expression of WT1, which is a transcription factor that marks mature podocytes, as well as that of nephrin, podocin, and nestin, proteins that contribute to foot process formation in differentiated podocytes. Staining experiments revealed a significant loss of podocyte differentiation markers in *PIK3CA^Pod-HET^* mice compared with controls (Figure 1, J and K, and Supplemental Figure 2, C–E). Mechanistically, PI3Kα plays a role in cell growth and proliferation. Therefore, we examined for proliferation within the glomeruli of these mouse models using Ki-67, which is a marker for cellular proliferation. While the number of Ki-67^+^ cells was very low in control mice, a notable increase in the number of Ki-67^+^ cells was observed within the glomeruli of *PIK3CA^Pod-HET^* mice. Immunofluorescence experiments using Ki-67, GFP, and PDGFRβ revealed that both podocytes and mesangial cells were undergoing proliferation (Figure 1, L and M, and Supplemental Figure 2, F–H). We further observed that proliferation started at 3 months of age and increased with aging (Supplemental Figure 2, I and J). These findings align with the notion that cells undergoing cell cycle entry tend to lose their differentiation markers (16). Then, using an Amnis ImageStream system, we confirmed that podocytes isolated from *PIK3CA^Pod-HET^* mice were hypertrophic compared with controls (Figure 1N). Flow cytometry experiments further demonstrated a correlation between podocyte size and the level of activation of the AKT/mTOR pathway, particularly the mTORC1 pathway (Supplemental Figure 2K). Indeed, we concluded that the excessive activation of PI3Kα in podocytes leads to both dedifferentiation and proliferation, which results in FSGS with progressive proteinuria and kidney dysfunction. This mouse model effectively recapitulates the kidney phenotype observed in patient 1.

To investigate the impact of allele dosage on the overactivation of the PI3Kα pathway, we generated mice that were homozygous for the *PIK3CA* mutation specifically in podocytes and referred to them as *PIK3CA^Pod-HO^* mice. At birth, *PIK3CA^Pod-HO^* mice were initially indistinguishable from controls. However, these mice rapidly developed a significant amount of albuminuria (Figure 1O) with reduced survival rates (Figure 1F). Histological analysis conducted at the age of 12 weeks revealed severe proliferative glomerulonephritis (Supplemental Figure 3, A and B). Transmission electron microscopy revealed hypertrophic podocytes, lipid vacuolization, extensive focal foot process effacement, and widespread glomerular basement membrane thickening (Supplemental Figure 3C). Consistently, we observed dedifferentiation, recruitment of the AKT/mTOR pathway, immune cell infiltration, and increased proliferation in both podocytes and mesangial cells (Supplemental Figure 3, D–L). These findings demonstrate that the cumulative activation of the PI3Kα pathway is associated with a more severe disease phenotype.

### Overactivation of the PI3Kα pathway in podocytes is associated with changes in cell fate determination.

**Figure 2:**
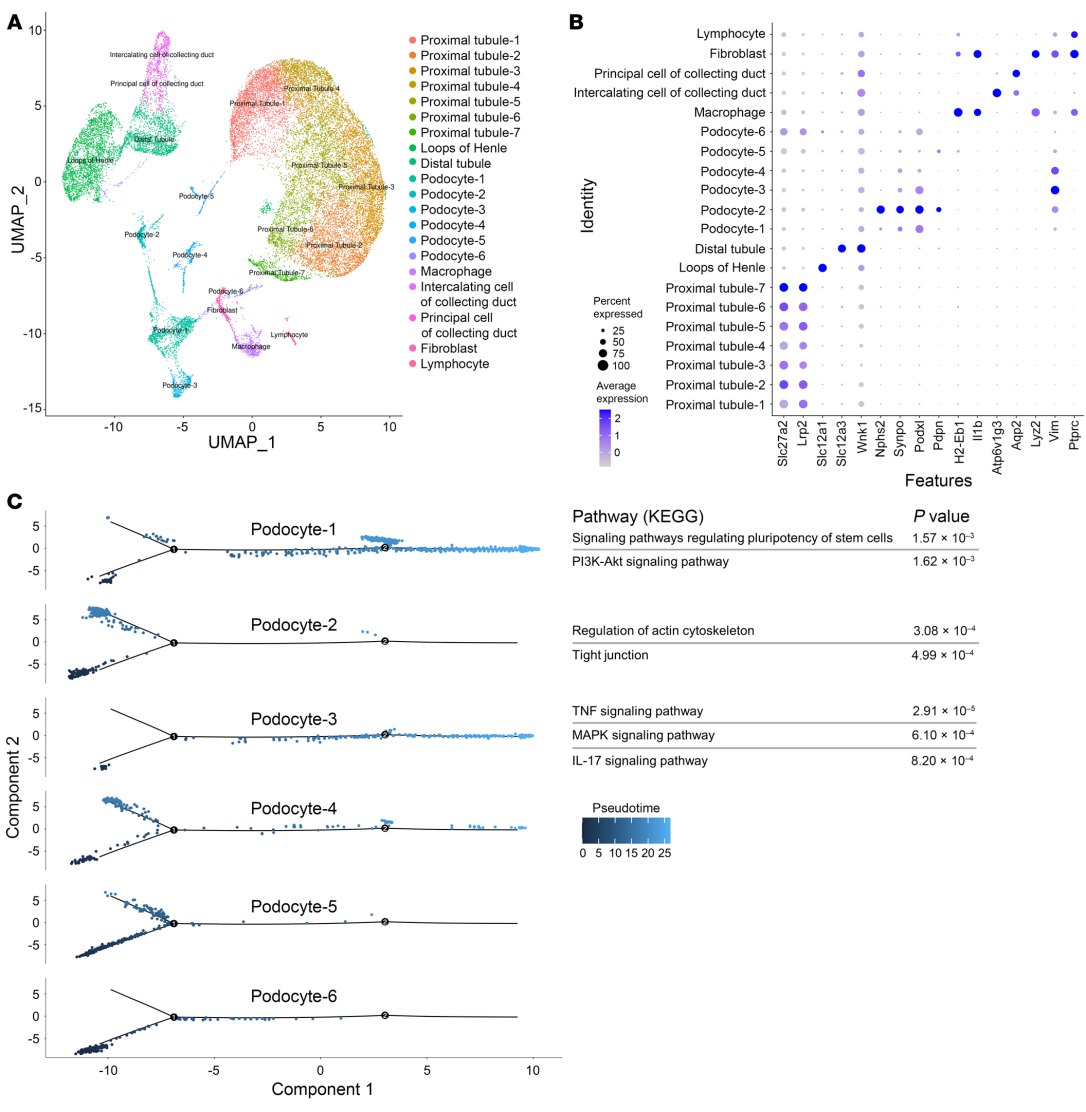
*PIK3CA* gain-of-function mutation in podocytes is associated with changes in cell fate determination. (**A**) Unsupervised clustering resulted in 20 distinct cell types in the UMAP plot. (**B**) Expression of cell-type-specific markers in the annotated clusters. The cell percentage expressing the gene is indicated by the dot’s size, and the average expression of the gene is indicated by the color concentration. (**C**) Cell trajectory map of each podocyte cluster showing the pseudotime. The significant pathways resulting from the KEGG pathway analysis (see Supplemental Table 2) are displayed adjacent to podocyte clusters 1, 2, and 3.

**Figure 3:**
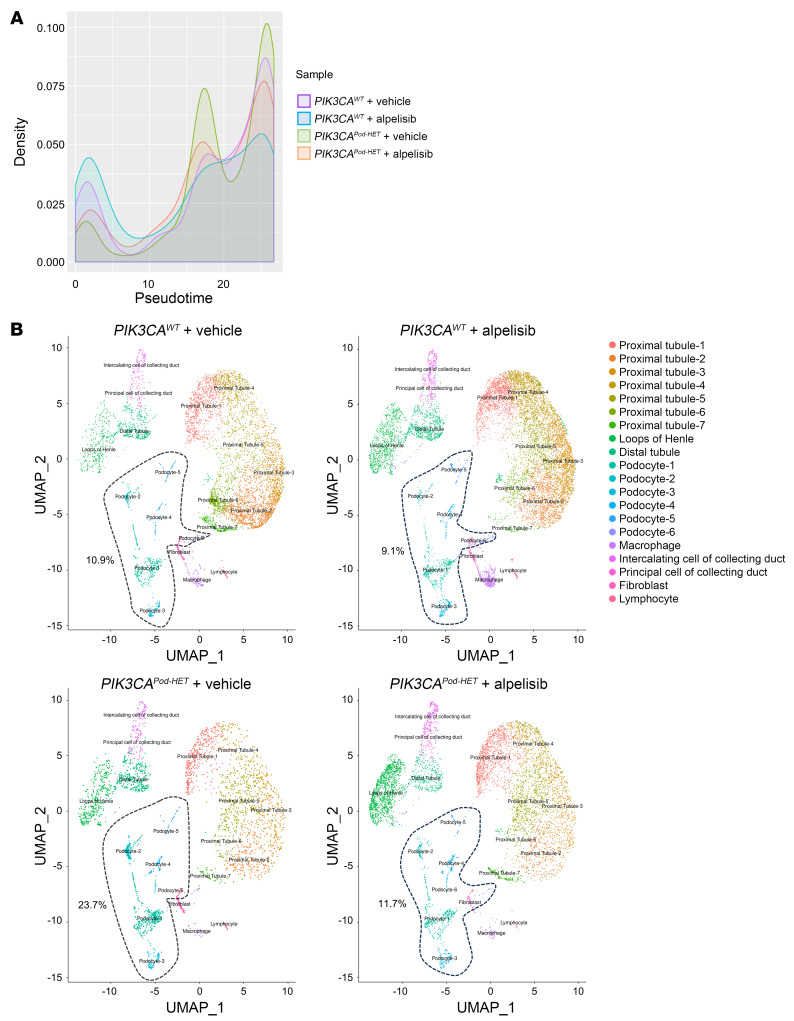
Alpelisib modifies podocyte cell fate determination in *PIK3CA^Pod-HET^* mice. (**A**) Histogram showing the distribution of podocyte clusters 1–6 along pseudotime for each sample. *PIK3CA^Pod-HET^* vehicle-treated mouse podocytes show a distinct distribution. (**B**) UMAP plot per sample showing the populational change for podocyte clusters. Podocyte clusters 1–6 are indicated by dotted lines that encompass their respective percentages of total cells in each sample.

Podocytes are postmitotic cells with limited possibilities of proliferation and renewal. However, under certain pathological circumstances, podocytes can reenter the cell cycle and become dedifferentiated (17). To identify the early transcriptional changes in podocytes associated with podocyte *PI3KCA* gain-of-function mutation, we performed single-cell RNA sequencing (scRNA-seq) and mapped kidney cells from *PIK3CA^WT^* and *PIK3CA^Pod-HET^* mice (Supplemental Figure 4A). To increase the podocyte population, which counts as only 0.18% of the normal mouse kidney (18), we enriched the glomeruli with an injection of magnetic beads. Two mice for each condition were pooled to increase the number of cells. A total of 49,600 cells were isolated and sequenced. After data normalization and filtering (Supplemental Figure 4B), 26,508 cells were further analyzed. The unsupervised clustering of the entire pooled data set identified 20 clusters (Figure 2A and Supplemental Figure 4C). With well-established cell-type markers from the literature, we annotated broad cluster classes (Figure 2, A and B, Supplemental Figure 4D, and Supplemental Table 1). We annotated 6 podocyte clusters based on the expression of *Nphs2*, *Synpo*, *Podxl*, *Pdpn*, and *EGFP* (Figure 2B and Supplemental Figure 4D). We decided to include some of the clusters with weaker expression of podocyte markers (Podocyte-4, -5, and -6) with the aim of understanding different cell states of podocytes. The monocle single-cell trajectory from 6 podocyte clusters resulted in 5 distinct states (Figure 2C and Supplemental Figure 4E). While podocyte-2, -4, and -5 clusters showed similar pseudotemporal ordering distributions, podocyte-1 and -3 clusters were distinct (Figure 2C). The podocyte-1 cluster was activated for the PI3K/AKT pathway and signaling pathways regulating pluripotency; the podocyte-3 cluster was enriched for inflammatory pathways (Supplemental Table 2). In *PIK3CA^WT^* mice, podocyte clusters accounted for approximately 10% of the whole population of analyzed cells, while in *PIK3CA^Pod-HET^* mice, it increased to 23.7%, which supported the proliferation of podocytes (Figure 3, A and B). We finally observed that podocytes of *PIK3CA^Pod-HET^* mice showed a significant differential expression of genes that are known to be upregulated in FSGS (19), such as *Wnt4*, metallothioneins *Mt1* and *Mt2*, *Col1a2*, *Col4a3*, *Col4a4*, or *Il18*, which are related to the inhibition of apoptosis, dedifferentiation, and proliferation (Supplemental Figure 4F). These results indicate that PI3K/AKT pathway activation in podocytes drives podocyte fate transition.

### Alpelisib improves PIK3CA^Pod^ mouse models.

Patient 1 demonstrated renal failure amelioration upon the introduction of alpelisib (11). However, the concurrent shrinkage of vascular malformations and correction of severe congestive heart failure caused by alpelisib treatment added potential confounding factors (11). To gain a better understanding of whether alpelisib was effective in improving kidney dysfunction in our patient, we first investigated the ability of the drug to diffuse into the kidneys. Liquid chromatography–mass spectrometry (LC-MS) analysis of mouse kidneys revealed that alpelisib was detectable 1.5 hours after oral administration at concentrations similar to those found in skeletal muscle and the liver (Supplemental Figure 5A). Subsequently, we administered alpelisib orally over a period of 3 weeks to 3-month-old *PIK3CA^Pod-HET^* mice (Supplemental Figure 5B). Treatment with alpelisib resulted in a gradual reduction in albuminuria (Supplemental Figure 5C) along with inhibition of the AKT/mTOR pathway in glomeruli (Supplemental Figure 5, D and E), increased expression of nephrin and synaptopodin (Supplemental Figure 5, F and G), and a decrease in podocyte size (Supplemental Figure 5H). scRNA-seq analysis of kidney cells from alpelisib-treated *PIK3CA^Pod-HET^* mice revealed that podocyte populations were restored to 11.7% compared with vehicle-treated *PIK3CA^Pod-HET^* mice (Figure 3, A and B). In addition, we observed that alpelisib treatment was associated with the correction of various gene expression anomalies (Supplemental Figure 4, F and G). These results indicate that alpelisib was able to successfully interfere with podocyte fate transition induced by overactivation of the PI3K/AKT pathway.

Then, we administered either the vehicle or alpelisib to the most severe model, *PIK3CA^Pod-HO^* mice, at 10 weeks of age when proteinuria had already been established (Supplemental Figure 6, A and B). The treatment period lasted for 2 consecutive weeks. Throughout the treatment period, we observed a progressive reduction in albuminuria in the alpelisib-treated mice, which was accompanied by a significant improvement in kidney function (Supplemental Figure 6, C–E). At the end of the treatment period, the mice were sacrificed and histological analysis of the kidneys revealed that *PIK3CA^Pod-HO^* mice treated with alpelisib exhibited preserved glomeruli compared with the vehicle-treated group (Supplemental Figure 6, F and G). Podocyte differentiation markers were still expressed at the protein level in the alpelisib-treated group compared with vehicle-treated mice (Supplemental Figure 6H). The activation of the AKT/mTOR pathway was attenuated following alpelisib treatment (Supplemental Figure 6, H–J). Mechanistically, the alpelisib treatment was associated with a decrease in glomerular cell proliferation (Supplemental Figure 6, K and L).

**Figure 4:**
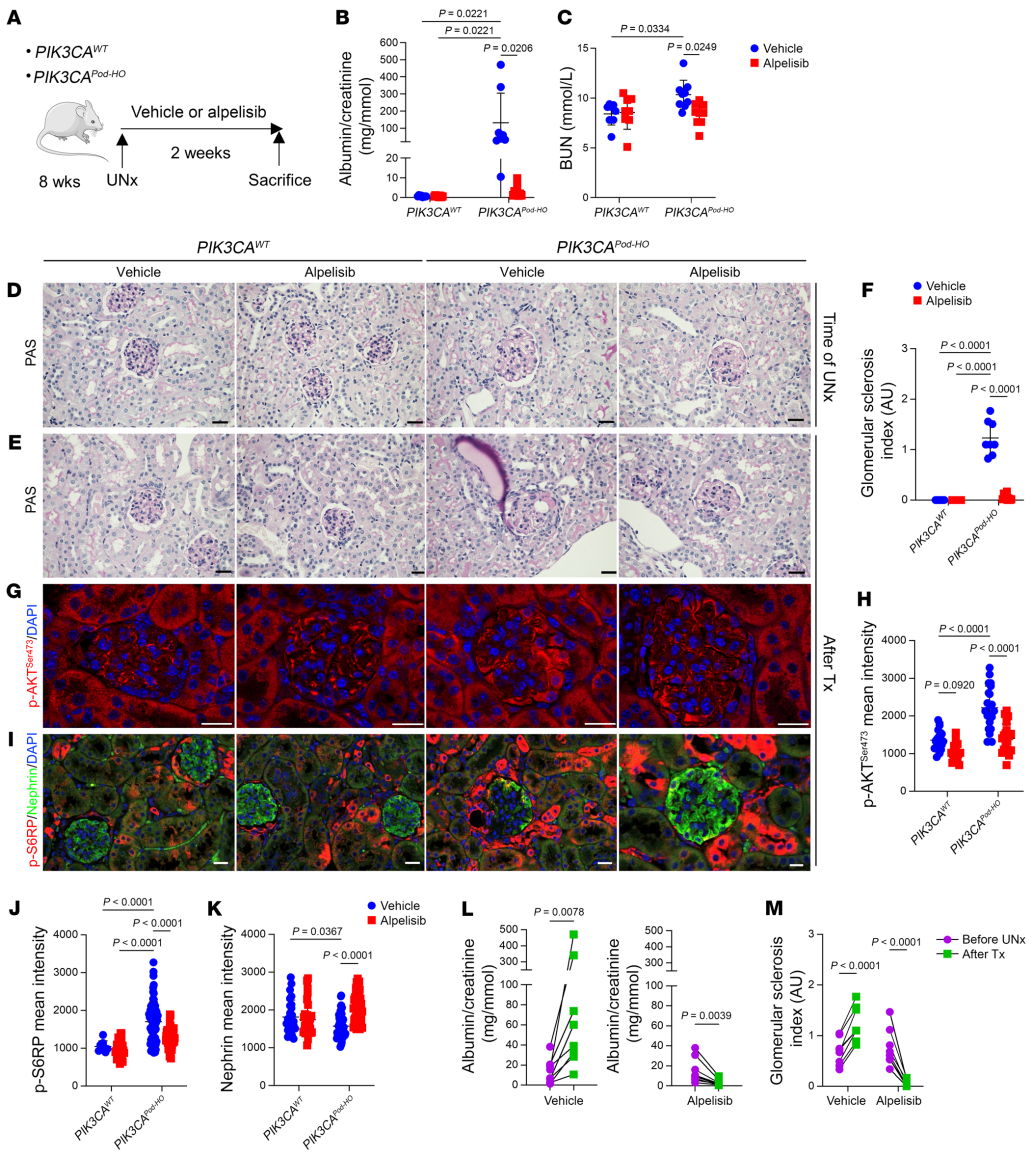
Alpelisib improves kidney lesions in uninephrectomized *PIK3CA^Pod-HO^* mice. (**A**) Experimental protocol design. UNx, uninephrectomy. (**B**–**M**) *n* = 8 for *PIK3CA^WT^*-vehicle, *PIK3CA^WT^*-alpelisib, *PIK3CA^HO^*-vehicle, and *n =* 9 for *PIK3CA^HO^*-alpelisib, unless otherwise stated. (**B** and **C**) Urinary albumin/creatinine ratio and BUN of *PIK3CA^WT^* and *PIK3CA^Pod-HO^* mice at sacrifice (2 weeks of treatment following UNx). (**D**) Representative PAS staining of UNx kidneys from *PIK3CA^WT^* and *PIK3CA^Pod-HO^* mice. (**E**–**K**) Kidneys from *PIK3CA^WT^* and *PIK3CA^Pod-HO^* mice at sacrifice. (**E**) PAS staining and (**F**) glomerular sclerosis (GS) index. (**G**) p-AKT^Ser473^ immunofluorescent staining and (**H**) p-AKT^Ser473^ quantification of glomeruli (*n =* 5 mice per group). Tx, treatment. (**I**) p-S6RP/nephrin coimmunofluorescent staining and their glomerular quantification (**J** and **K**) (*n =* 5 mice per group). (**L**) Trajectory of the albumin/creatinine ratio in the urine, and (**M**) trajectory of GS index of *PIK3CA^Pod-HO^* mice following UNx and treated with either vehicle or alpelisib. Data are represented as mean ± SD from 3 independent experiments (**B**, **C**, **F**, **L**, and **M**) or mean ± SD and representative of 3 independent experiments (**H**, **J**, and **K**). *P* values were calculated using 2-way ANOVA with Tukey’s post hoc test (**B**, **C**, **F**, **H**, **J**, and **K**), Wilcoxon’s matched-pairs signed-rank test (**L**), or 2-way ANOVA with Bonferroni’s multiple-comparison test (**M**). Scale bars: 32.2 μm (**D** and **E**) and 20 μm (**G** and **I**). Some data are identical between **B** and **L**.

To better assess the improvement and potential reversibility of kidney lesions, we conducted unilateral nephrectomy in both *PIK3CA^Pod-HO^* mice and controls at 8 weeks of age (Figure 4A). The mice were randomly assigned to receive either the vehicle or alpelisib treatment from postoperative day 1 for a duration of 2 weeks (Supplemental Figure 7, A and B). We first observed that uninephrectomized *PIK3CA^WT^* mice, whether treated with the vehicle or alpelisib, did not display any notable phenotype, with no presence of proteinuria or kidney dysfunction (Figure 4, B–K, and Supplemental Figure 7C). In contrast, *PIK3CA^Pod-HO^* mice treated with the vehicle following surgery rapidly developed a significant amount of proteinuria, kidney dysfunction, severe glomerular lesions, increased AKT activity, and decreased expression of podocyte markers (Figure 4, B–K, and Supplemental Figure 7C). However, *PIK3CA^Pod-HO^* mice treated with alpelisib following surgery demonstrated a significantly reduced amount of proteinuria, no kidney dysfunction, preserved glomerular lesions, a higher expression of podocyte markers, and decreased activation of the podocyte AKT/mTOR pathway (Figure 4, B–K, and Supplemental Figure 7C). Interestingly, the trajectory of albuminuria had an opposite effect following uninephrectomy in *PIK3CA^Pod-HO^* mice treated with alpelisib compared with those treated with the vehicle (Figure 4L). Furthermore, a comparison of glomerular lesions before and after surgery indicated that alpelisib treatment was associated with a potential reversal of kidney lesions in *PIK3CA^Pod-HO^* mice (Figure 4M). Based on these findings, we concluded that alpelisib was able to prevent and potentially reverse the disease in a mouse model carrying *PIK3CA* gain-of-function mutation in podocytes.

### Alpelisib improves different mouse models of proliferative glomerulonephritis.

**Figure 5:**
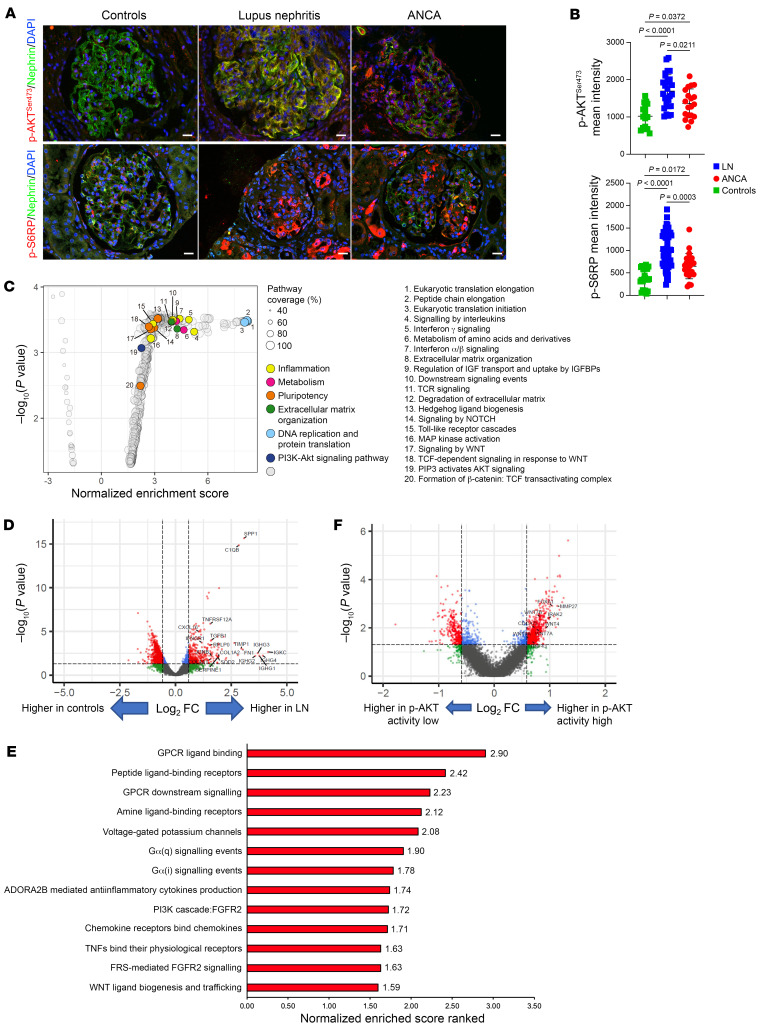
Spatial transcriptomic changes observed in glomeruli from patients with class 4 lupus nephritis. (**A**) Representative coimmunofluorescent staining of nephrin/p-AKT^Ser473^ (frozen section) and nephrin/p-S6RP in kidney biopsies from patients with lupus nephritis (LN), ANCA vasculitis, or controls (*n =* 4 patients per group). (**B**) Glomerular p-AKT^Ser473^ and p-S6RP quantification (*n =* 4 patients per group). (**C**) GSEA mapping from the Reactome database for LN versus controls. Some of the pathways of interest that are enriched in LN samples are highlighted in colored dots as follows: inflammation related in yellow, metabolism related in magenta, pluripotency related in orange, extracellular matrix organization related in green, DNA replication and protein translation related in light blue, and PI3K/AKT signaling pathway related in navy blue. Gray circles indicate “other.” (**D**) Volcano plot between controls and LN samples. Significantly differentially expressed genes are indicated by red dots. FC, fold change. (**E**) Bar plot of the top normalized enrichment score–ranked gene sets (Hallmark, GSEA) with *P* < 0.001 in LN p-AKT activity–high kidneys compared with LN p-AKT activity–low ROIs. (**F**) Volcano plot between the LN p-AKT activity–high and LN p-AKT activity–low ROIs. Significantly differentially expressed genes are indicated by red dots. Data are represented as mean ± SD, and *P* values were calculated using 1-way ANOVA with Tukey’s post hoc test (**B**). Scale bars: 20 μm (**A**).

*PIK3CA^Pod^* mouse models developed characteristics resembling collapsing glomerulopathy and extracapillary disorders. In humans, collapsing glomerulopathy can occur as a primary condition or as a secondary manifestation of other diseases such as HIV infection or toxic exposures (20). On the other hand, crescentic glomerulonephritis is the hallmark of severe autoimmune diseases affecting the kidneys such as lupus nephritis (LN) and antineutrophil cytoplasmic antibody (ANCA) vasculitis. Both types of glomerular lesions are associated with significant kidney damage and poor renal survival. To further investigate whether targeting the AKT/mTOR pathway in the context of these disorders is reasonable, we first conducted immunofluorescence experiments in human patient kidneys. These experiments confirmed the activation of the AKT/mTOR pathway in podocytes compared with kidney biopsies from healthy individuals who underwent a biopsy because of a low-rate proteinuria (<1 g/L) or mild hematuria, with normal histological examination and no immunofluorescent deposits (Figure 5, A and B, and Supplemental Figure 8, A and B).

We further investigated the glomerular spatial transcriptomic changes in patients with extracapillary disorders using GeoMx digital spatial profiler technology. Kidney biopsies from 4 patients with highly active LN and 4 healthy controls were selected for analysis (Supplemental Figure 8, B and C). A total of 49 glomeruli were selected as regions of interest (ROIs) (33 ROIs for LN and 16 ROIs for controls). More than 18,000 genes were assayed in these ROIs (Supplemental Figure 8D). The glomerular transcriptomes of LN samples showed distinct and broader clustering patterns compared with those of control samples (Supplemental Figure 8E). Consistent with scRNA-seq analysis in *PIK3CA^Pod-HET^* mice, the gene expression profiles in LN glomeruli indicated a dedifferentiated state and elevated levels of inflammation-related transcripts (Figure 5, C and D). We separately stained p-AKT^Ser473^ in LN samples and classified each ROI based on the level of p-AKT^Ser473^ activity, with 20 ROIs classified as activity-high and 13 ROIs classified as activity-low (Supplemental Figure 8F). The higher p-AKT^Ser473^ activity correlated with gene set enrichment of the WNT pathway and significant upregulation of *Wnt4* (Figure 5, E and F). Indeed, *PIK3CA^Pod-HET^* mutant mice demonstrate similar transcriptomic changes that closely resemble patterns observed in LN patients.

Based on these findings and previous reports on the association between podocytes and their disease pathology (21–24), we investigated whether the inhibition of PIK3CA could be a potential therapeutic approach for collapsing glomerulopathy and extracapillary glomerular nephritis.

**Figure 6:**
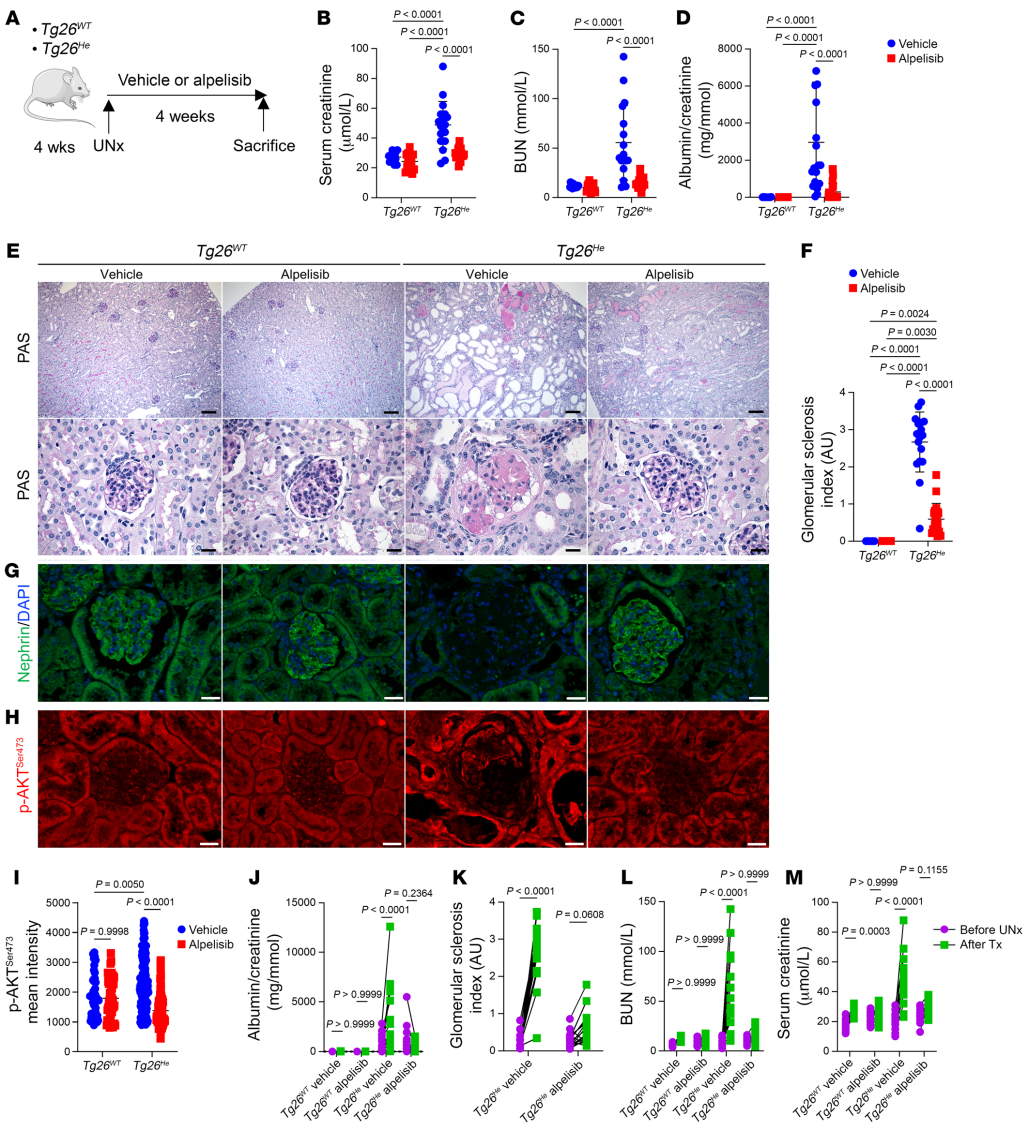
Alpelisib improves kidney lesions in an accelerated mouse model of collapsing glomerulopathy. (**A**) Experimental protocol design. (**B**–**M**) *Tg26^WT^* and *Tg26^He^* mice at the time of sacrifice (*n =* 16 for *Tg26^WT^*-vehicle, *n =* 15 for *Tg26^WT^*-alpelisib, *n =* 18 for *Tg26^He^*-vehicle, *n =* 19 for *Tg26^He^*-alpelisib, unless otherwise stated). (**B**) Serum creatinine. (**C**) BUN. (**D**) Urinary albumin/creatinine ratio. (**E**) Representative PAS staining of kidneys. (**F**) Glomerular sclerosis (GS) index quantification. (**G**) Representative nephrin immunofluorescent staining of kidneys. (**H**) Representative p-AKT^Ser473^ immunofluorescence and its glomerular quantification (**I**) from *Tg26^WT^* and *Tg26^He^* mouse kidneys at sacrifice (*n =* 6 mice per group). (**J**) The urinary albumin/creatinine ratio trajectory of *Tg26^WT^* and *Tg26^He^* mice following uninephrectomy (UNx) and treatment (Tx). (**K**) GS index trajectory between UNx and sacrifice kidneys of *Tg26^He^* mice. (**L**) BUN. (**M**) Serum creatinine level trajectory of *Tg26^WT^* and *Tg26^He^* mice following UNx and Tx. Data are represented as mean ± SD and are from 6 independent experiments (**B**–**F** and **J**–**M**), or are mean ± SD and representative of 3 independent experiments (**H** and **I**). *P* values calculated using 2-way ANOVA with Tukey’s post hoc test (**B**–**D**, **F**, and **I**) or 2-way ANOVA with Bonferroni’s multiple-comparison test (**J**–**M**). Scale bars: 130 μm (**E**, upper), 32.2 μm (**E**, lower), and 20 μm (**G** and **H**). Note: some data are shared between **B** and **M**, **C** and **L**, **D** and **J**, and **G** and **K**.

We initially examined *Tg26^He^* mice, which serve as a model for collapsing glomerulopathy (25). Typically, these transgenic mice develop proteinuria around 24 days of age, exhibit severe kidney lesions, and die between 2 and 9 months of age (25). We observed activation of the AKT/mTOR pathway in podocytes of 4-week-old *Tg26^He^* mice compared with controls (Supplemental Figure 9, A and B). Since *Tg26^He^* mice are very variable in terms of severity and course of the disease (Supplemental Figure 9C), we decided to employ the experimental model of unilateral nephrectomy, to standardize the variability and accelerate the disease progression. This approach would in addition expedite the development of kidney lesions and allow a direct comparison of paired kidneys before and after surgery. At 4 weeks old, both *Tg26^He^* and *Tg26^WT^* mice underwent uninephrectomy followed by a random assignment to receive either the vehicle (*Tg26^WT^ n =* 16, *Tg26^He^* *n =* 15) or alpelisib (*Tg26^WT^ n =* 18, *Tg26^He^* *n =* 19) treatment for a duration of 4 weeks (Figure 6A). Upon sacrifice, we observed that while the surfaces of the kidneys in uninephrectomized *Tg26^WT^* or *Tg26^He^* mice treated with alpelisib appeared regular and smooth, the surfaces of the kidneys from *Tg26^He^* mice treated with the vehicle exhibited irregularities (Supplemental Figure 9D). Kidney function and albuminuria were significantly improved in *Tg26^He^* mice treated with alpelisib compared with those treated with the vehicle (Figure 6, B–D). Additionally, the kidney-to–body weight ratio showed that kidney hypertrophy caused by uninephrectomy was improved in uninephrectomized *Tg26^He^* mice treated with alpelisib (Supplemental Figure 9E). Histological examination of the kidneys revealed that alpelisib-treated uninephrectomized *Tg26^He^* mice exhibited fewer glomerular lesions (Figure 6, E and F) and maintained the expression of podocyte markers (Figure 6G and Supplemental Figure 9F). Furthermore, the expression of *Col1a*, *Col3a*, and *Lcn2* was reduced in the alpelisib-treated group (Supplemental Figure 9, G–I). Mechanistically, the AKT/mTOR pathway in glomeruli showed significant suppression in mice treated with alpelisib (Figure 6, H and I, and Supplemental Figure 9, J and K). Finally, we compared the kidney function, albuminuria, and glomerular lesion score before and after nephrectomy and observed that the rate of disease progression in mice treated with alpelisib was lower compared with those treated with the vehicle (Figure 6, J–M).

**Figure 7:**
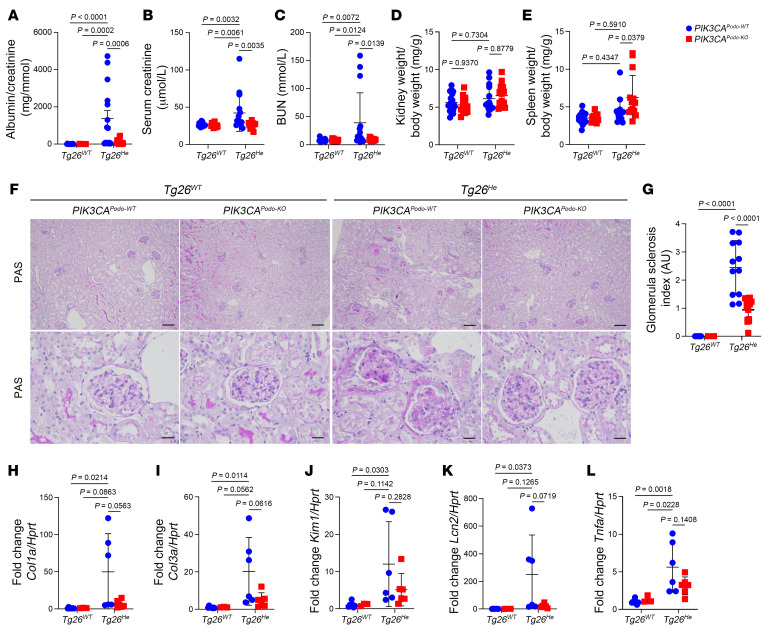
*PIK3CA* deletion in podocytes improves kidney lesions in an accelerated mouse model of collapsing glomerulopathy. (**A**–**G**) Fifteen-week-old *Tg26^WT^* *PIK3CA^Podo-WT^* (*n =* 18), *Tg26^WT^* *PIK3CA^Podo-KO^* (*n =* 14), *Tg26^He^* *PIK3CA^Podo-WT^* (*n =* 15), and *Tg26^He^* *PIK3CA^Podo-KO^* mice (*n =* 15). (**A**) Urinary albumin/creatinine ratio. (**B**) Serum creatinine level. (**C**) BUN level. (**D**) Kidney-to–body weight ratio. (**E**) Spleen-to–body weight ratio. (**F**) Representative PAS staining of kidneys. (**G**) Glomerular sclerosis (GS) index quantification. (**H**) *Col1a*, (**I**) *Col3a*, (**J**) *Kim1*, (**K**) *Lcn2*, and (**L**) *Tnfa* quantification of qRT-PCR analysis in the kidney cortex from 15-week-old *Tg26^WT^* *PIK3CA^Podo-WT^* (*n =* 7), *Tg26^WT^* *PIK3CA^Podo-KO^* (*n =* 3), *Tg26^He^* *PIK3CA^Podo-WT^* (*n =* 6), and *Tg26^He^* *PIK3CA^Podo-KO^* mice (*n =* 6). Data are represented as mean ± SD from at least 3 independent experiments (**A**–**E** and **G**–**L**). *P* values calculated using 2-way ANOVA with Tukey’s post hoc test (**A**–**E** and **G**–**L**). Scale bars: 130 μm (**F**, upper) and 32.2 μm (**F**, lower).

**Figure 8:**
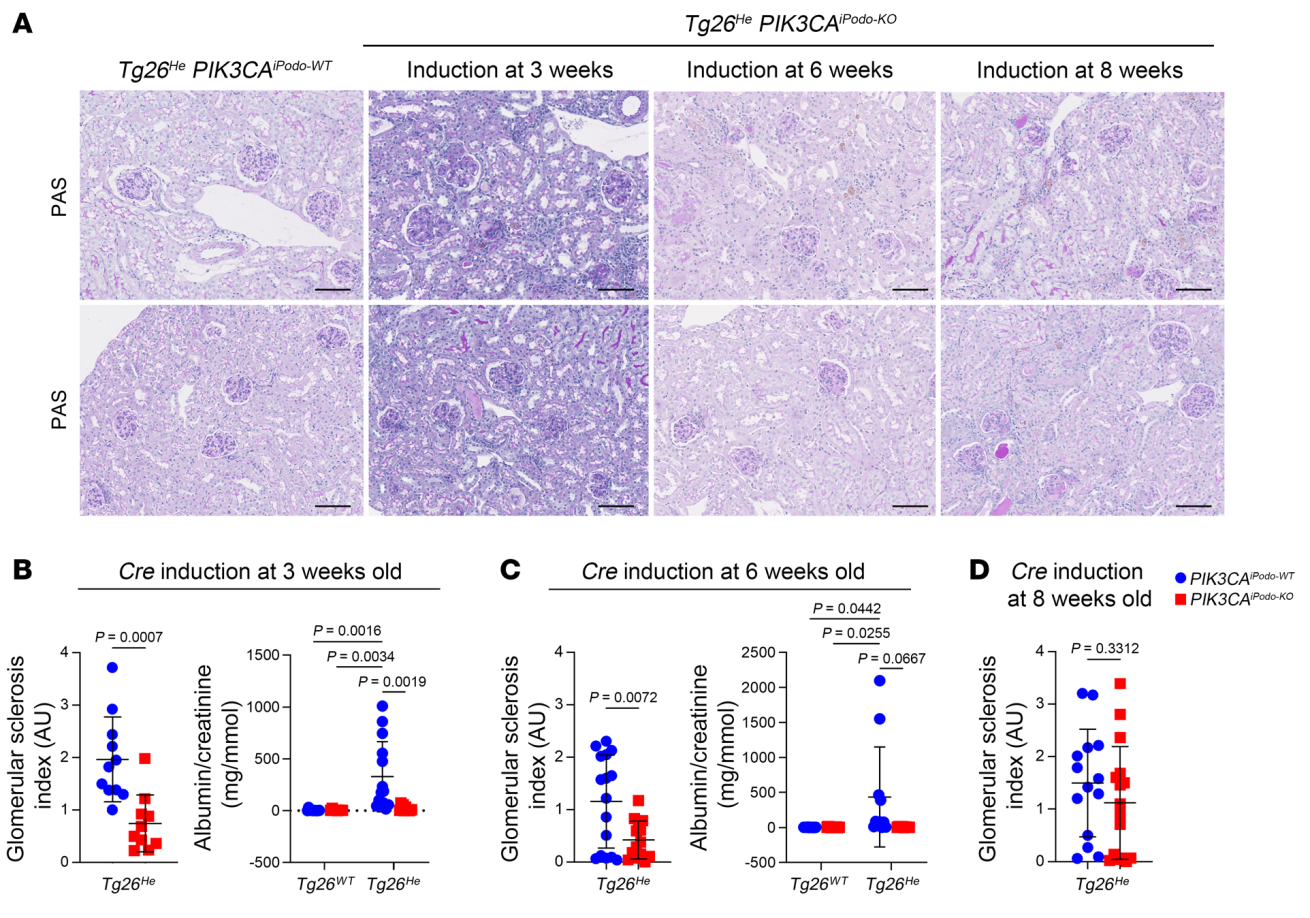
*PIK3CA* deletion in podocytes up to the age of 6 weeks is able to reverse kidney lesions in *Tg26* mice. (**A**) Representative PAS staining of the kidneys at 12 weeks from *Tg26^He^* with either *PIK3CA^iPodo-WT^* or *PIK3CA^iPodo-KO^* mice, which were induced with tamoxifen at different time points (3-week-old *Tg26^WT^* *PIK3CA^iPodo-WT^*, *n =* 10; 3-week-old *Tg26^WT^* *PIK3CA^iPodo-KO^*, *n =* 8; 3-week-old *Tg26^He^* *PIK3CA^iPodo-WT^*, *n =* 14; 3-week-old *Tg26^He^* *PIK3CA^iPodo-KO^*, *n =* 11; 6-week-old *Tg26^WT^* *PIK3CA^iPodo-WT^*, *n =* 10; 6-week-old *Tg26^WT^* *PIK3CA^iPodo-KO^*, *n =* 14; 6-week-old *Tg26^He^* *PIK3CA^iPodo-WT^*, *n =* 16; 6-week-old *Tg26^He^* *PIK3CA^iPodo-KO^*, *n =* 14; 8-week-old *Tg26^WT^* *PIK3CA^iPodo-WT^*, *n =* 10; 8-week-old *Tg26^WT^* *PIK3CA^iPodo-KO^*, *n =* 11; 8-week-old *Tg26^He^* *PIK3CA^iPodo-WT^*, *n =* 14; 8-week-old *Tg26^He^* *PIK3CA^iPodo-KO^*, *n =* 16). (**B**–**D**) Glomerular sclerosis (GS) index quantification, and the urinary albumin/creatinine ratio of mice that were induced at indicated time points. Data are represented as mean ± SD from at least 3 independent experiments (**B**–**D**). *P* values calculated using 2-way ANOVA with Tukey’s post hoc test (**B**, right; **C**, right) or 2-tailed Student’s *t* test (**B**, left; **C**, left; and **D**). Scale bars: 100 μm (**A**).

To specifically investigate the role of PI3Kα in podocytes during collapsing glomerulopathy, we employed a genetic approach to remove *PIK3CA* specifically in these cells. To achieve this, we generated *PIK3CA^Podo-KO^* mice by crossing *Podocin*-Cre mice with *PIK3CA^lox/lox^* mice. These mice were subsequently backcrossed with the FVB/NJ strain for 10 generations and bred with *Tg26^He^* mice to obtain *Tg26^He^* *PIK3CA^Podo-KO^* mice. These mice were viable and had no particular phenotype at birth. However, as they aged, we observed that *Tg26^He^* *PIK3CA^Podo-KO^* mice displayed reduced levels of albuminuria, milder glomerular lesions, decreased kidney fibrosis, and improved kidney function compared with control littermate *Tg26^He^* *PIK3CA^Podo-WT^* mice (Figure 7, A–L). Importantly, the deletion of *PIK3CA* in podocytes alone was sufficient to alleviate the severity of collapsing glomerular lesions and improve the kidney function in the *Tg26^He^* mouse model. Next, we attempted to determine whether removing *PIK3CA* in podocytes later in life, when glomerular lesions were already established, could lead to an improvement in kidney lesions. For this purpose, we generated *PIK3CA^iPodo-KO^* mice by backcrossing tamoxifen-inducible *Podocin*-Cre mice with *PIK3CA^lox/lox^* mice on the FVB/NJ strain (10 generations) and crossing them with *Tg26^He^* mice to obtain *Tg26^He^* *PIK3CA^iPodo-KO^*. Cre recombination was induced in 3-week-old *Tg26^He^* *PIK3CA^iPodo-KO^* mice, and the mice were sacrificed at 12 weeks. We observed that these mice exhibited improved glomerular lesion scores and reduced albuminuria compared with the control group (Figure 8, A and B). Similarly, when Cre recombination was induced at 6 weeks of age, kidney lesions were reversed by 12 weeks (Figure 8, A and C). However, when Cre recombination was induced at a later time point, specifically at 8 weeks of age, the progression of the disease was no longer affected, which indicated that reversing the phenotype becomes challenging once the kidney lesions have become too severe (Figure 8, A and D). In this model of collapsing glomerulopathy, we determined that the activation of PI3Kα in podocytes is significantly important and inhibition holds great promise as a potential therapeutic approach.

**Figure 9:**
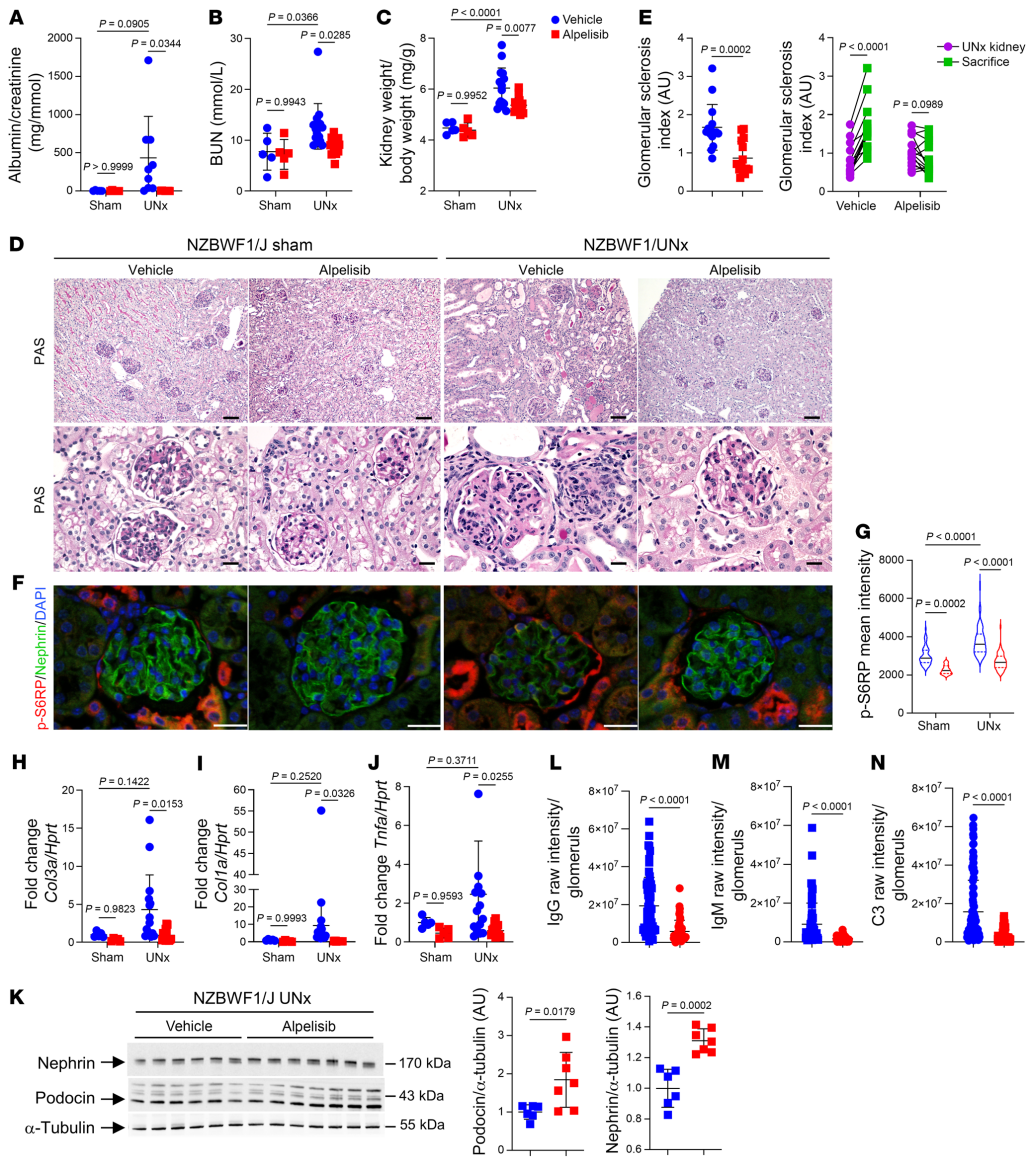
Alpelisib improves kidney lesions in NZBWF1/J lupus nephritis models. (**A**–**J**) NZBWF1/J mice at sacrifice unless otherwise stated (sham-vehicle and sham-alpelisib, *n =* 5; UNx-vehicle and UNx-alpelisib, *n =* 15). (**A**) Urinary albumin/creatinine ratio. (**B**) BUN. (**C**) Kidney-to–body weight ratio. (**D**) Representative PAS staining. (**E**) Left: Glomerular sclerosis (GS) index quantification at sacrifice. Right: GS trajectory between uninephrectomy (UNx) and sacrifice of the same mouse. GS at sacrifice shared. (**F**) Representative p-S6RP/nephrin coimmunofluorescent staining and (**G**) quantification (*n =* 6 per group). (**H**) *Col3a*, (**I**) *Col1a*, and (**J**) *Tnfa* quantification by qRT-PCR in kidney cortex. (**K**) Western blot and quantification of nephrin, podocin, and α-tubulin in kidney cortex (UNx-vehicle, *n =* 6; UNx-alpelisib, *n =* 7). (**L**) IgG, (**M**) IgM, and (**N**) C3 raw intensity per glomerulus quantification of the immunofluorescent staining (UNx-vehicle, *n =* 6; UNx-alpelisib, *n =* 7). Data represented as mean ± SD from 3 independent experiments (**A**–**C**, **E**, and **G**–**N**). *P* values calculated using 2-way ANOVA with Tukey’s post hoc test (**A**–**C** and **G**–**J**), 2-way ANOVA with Bonferroni’s multiple-comparisons test (**E**, right), 2-tailed Student’s *t* test (**E**, left and **K**), or 2-tailed Mann-Whitney *U* test (**L**–**N**). Scale bars: 130 μm (**D**, upper), 32.2μm (**D**, lower), and 20 μm (**F**).

Next, we investigated the impact of PI3Kα inhibition in extracapillary glomerulonephritis, particularly in lupus mouse models. Our initial approach involved using NZBWF1/J mice, a well-established model that exhibits lupus-like nephritis (26). NZBWF1/J mice gradually develop immune glomerulonephritis characterized by proteinuria and kidney dysfunction starting around 25 weeks of age (26). However, this model is also known for its variability (26). To standardize and expedite the formation of lesions, we employed the uninephrectomy model and performed uninephrectomy on 30 female mice at 24 weeks of age (Supplemental Figure 10A). Notably, the incidence and severity of symptoms are more pronounced in females and we used only females for our study. The mice were then randomly assigned to receive either the vehicle (*n =* 15) or alpelisib treatment (*n =* 15) for a duration of 4 weeks. At the end of the treatment period, the mice were sacrificed and their kidney histology was compared to samples obtained during the uninephrectomy procedure. At the time of uninephrectomy, no discernible differences were observed between the 2 groups in terms of phenotypic characteristics, including proteinuria and the kidney-to–body weight ratio (Supplemental Figure 10, B and C). However, upon sacrifice, mice treated with alpelisib exhibited significant reductions in albuminuria (Figure 9A) and blood urea nitrogen (BUN) levels (Figure 9B). Furthermore, the kidney-to–body weight ratio was notably decreased in uninephrectomized NZBWF1/J mice receiving alpelisib (Figure 9C). From a histological standpoint, the alpelisib-treated mice displayed preserved glomeruli compared with those receiving the vehicle (Figure 9, D and E). While glomerular lesions worsened considerably in the vehicle-treated uninephrectomized NZBWF1/J mice, they remained stable in the alpelisib group (Figure 9E). Additionally, the alpelisib treatment resulted in the attenuation of AKT/mTOR pathway activation within the glomeruli (Figure 9, F and G). The expression of *Col1a*, *Col3a*, and *Tnfa* mRNA was significantly reduced in mice treated with alpelisib (Figure 9, H–J). Moreover, the group of uninephrectomized NZBWF1/J mice treated with alpelisib demonstrated marked improvements in podocyte differentiation markers (Figure 9, F and K, and Supplemental Figure 10D). In NZBWF1/J mice treated with alpelisib, we observed a partial correction of anemia and a significant increase in platelet count (Supplemental Figure 10E). Then, we explored glomerular IgG, IgM, and C3 deposits and unexpectedly observed a significant reduction in deposits in glomeruli mice receiving alpelisib (Figure 9, L–N, and Supplemental Figure 10F). To obtain further insight, we measured the level of circulating double-stranded DNA (dsDNA), which is a marker of systemic lupus erythematosus disease activity, and noticed a significant decrease in NZBWF1/J mice treated with alpelisib (Supplemental Figure 10G). Notably, the spleen size was significantly reduced in both sham and uninephrectomized mice treated with alpelisib (Supplemental Figure 10H). A blood test revealed that alpelisib treatment was associated with a reduction in white blood cell count, particularly lymphocytes and monocytes (Supplemental Figure 10E). Flow cytometry of peripheral blood mononuclear cells (PBMCs) did not indicate any significant changes in the distribution of the lymphocyte population (Supplemental Figure 10I). An examination of the spleen revealed a decrease in both the B and T cell populations in NZBWF1/J mice treated with alpelisib (Supplemental Figure 10I). The B cell population was also significantly impacted in lymph nodes and bone marrow (Supplemental Figure 10I). These findings were unexpected because PI3Kδ is the predominantly expressed isoform in leukocytes (27–30), and it was not anticipated that alpelisib would have an impact on the lymphocyte population. To ascertain whether alpelisib was able to modify the lymphocyte population in WT mice, we conducted a study where we administered either the vehicle or alpelisib daily to 8-week-old FVB/NJ WT mice for 4 consecutive weeks. Afterwards, we examined the lymphocyte population and found no changes in white blood cell count (Supplemental Figure 11A) or in the lymphocyte population in lymph nodes, spleen, or PBMCs of alpelisib-treated mice compared to vehicle-treated mice (Supplemental Figure 11B). These results indicate that alpelisib specifically targeted lymphocytes in NZBWF1/J mice. Next, we measured the serum levels of circulating proinflammatory cytokines in NZBWF1/J mice treated with either the vehicle or alpelisib and observed a reduction in cytokines in alpelisib-treated mice (Supplemental Figure 10J). Collectively, these findings indicate that alpelisib might exert its effects on both podocytes and lymphocytes in NZBWF1/J mice, which results in the attenuation of LN.

**Figure 10:**
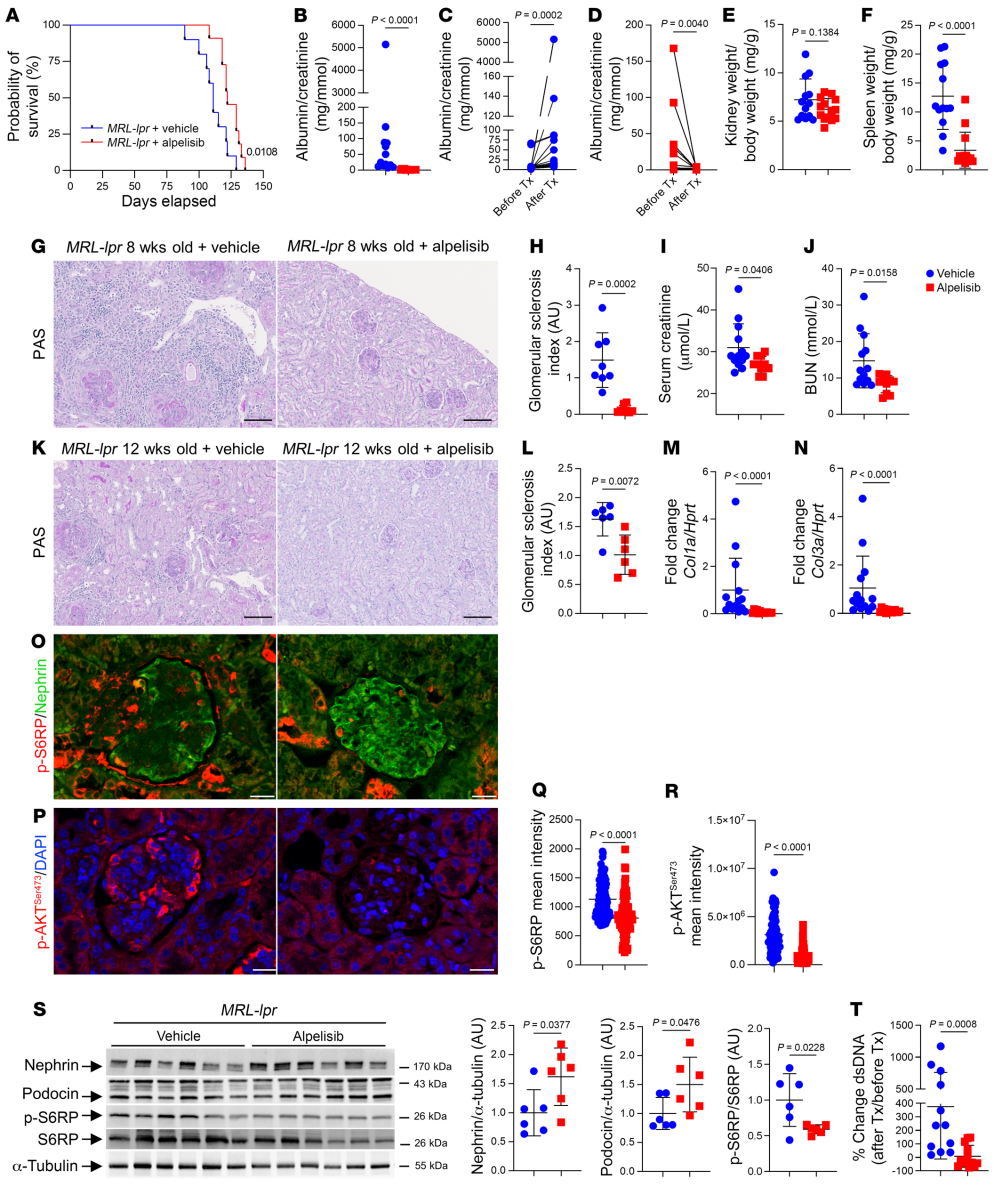
Alpelisib improves kidney lesions in *MRL-lpr* lupus nephritis models. (**A**–**J**) *MRL-lpr* mouse survival experiment (vehicle, *n =* 13, alpelisib, *n =* 14, unless otherwise stated). (**A**) Kaplan-Meier curves.(**B**) Urinary albumin/creatinine ratio at sacrifice. (**C** and **D**) Trajectory of urinary albumin/creatinine ratio (after treatment [Tx] are identical to **B**). (**E**) Kidney-to–body weight ratio. (**F**) Spleen-to–body weight ratio. (**G**) Representative PAS staining. (**H**) Glomerular sclerosis (GS) index quantification (*n =* 8 per group). (**I**) Serum creatinine. (**J**) BUN at sacrifice. (**K**–**T**) *MRL-lpr* treated either with vehicle or alpelisib from 12 until 16 weeks old (*n =* 6 per group except otherwise stated). (**K**) Representative PAS staining. (**L**) GS index quantification. (**M**) *Col1a* and (**N**) *Col3a* quantification by qRT-PCR in kidney cortex (*n =* 14 per group). (**O**) Representative p-S6RP/nephrin, (**P**) p-AKT^Ser473^ immunofluorescent staining, and (**Q** and **R**) their quantification. (**S**) Western blot and quantification of nephrin, podocin, p-S6RP, S6RP, and α-tubulin in kidney cortex. (**T**) Serum dsDNA titer change ratio (after Tx vs. before Tx) (vehicle, *n =* 12; alpelisib, *n =* 13). Data represented as mean ± SD from 3 independent experiments (**B**, **E**, **F**, **H**–**J**, **L**–**N**, and **Q**–**T**). *P* values calculated using log-rank (Mantel-Cox) test (**A**), 2-tailed Student’s *t* test (**E**, **F**, **I**, **J**, **L**, and **S**), Wilcoxon’s matched-pairs signed-rank test (**C** and **D**), or 2-tailed Mann-Whitney *U* test (**B**, **H**, **M**, **N**, **Q**, **R**, and **T**). Scale bars: 100 μm (**G** and **K**) and 20 μm (**O** and **P**).

To validate the significance of these findings, we conducted a further investigation on the effects of alpelisib in MRL/MpJ-*Fas^lpr^*/J mice (referred to here as *MRL-lpr* mice), which is another model of LN (31). These mice possess homozygous Fas mutations and typically develop an autoimmune disease resembling systemic lupus, which is characterized by lymphadenoproliferation, progressive renal failure, and skin lesions (32). Female *MRL-lpr* mice usually succumb to the disease by 18–20 weeks of age. To begin, we randomly assigned 8-week-old female *MRL-lpr* mice prior to the complete onset of the disease to receive either the vehicle or alpelisib. The administration of alpelisib resulted in a modest but significant increase in life expectancy (Figure 10A). It is important to note that mice were dying from well-characterized skin lesions and voluminous compressive lymph nodes around the neck (33). Furthermore, *MRL-lpr* mice treated with alpelisib exhibited reduced proteinuria compared with those treated with the vehicle (Figure 10B). More strikingly, the comparison of albuminuria before and after treatment introduction showed opposite trajectories. Specifically, *MRL-lpr* mice treated with alpelisib demonstrated an improvement in proteinuria, which suggests potential reversibility of the disease (Figure 10, C and D). At sacrifice, *MRL-lpr* mice treated with alpelisib showed a tendency toward a lower kidney-to–body weight ratio and a significant decrease in spleen weight compared with the vehicle-treated mice (Figure 10, E and F). An examination of the kidneys revealed that alpelisib treatment was associated with fewer glomerular lesions compared with the vehicle-treated mice (Figure 10, G and H) and improved kidney function (Figure 10, I and J). To further demonstrate the potential reversibility of glomerular lesions with alpelisib treatment, we randomly assigned either the vehicle or alpelisib to 12-week-old *MRL-lpr* male and female mice. At this age, kidney lesions are already established. Mice were then treated for 4 consecutive weeks (Supplemental Figure 12A). Alpelisib was again associated with proteinuria reduction (Supplemental Figure 12B). At the histological level, we found that the glomerular lesion score was improved in the alpelisib group, as was *Cola1* and *Cola3* mRNA expression (Figure 10, K–N). Consistently, the glomerular AKT/mTOR pathway was inhibited in the group of alpelisib-treated mice (Figure 10, O–S). The expression of podocyte differentiation markers was increased in alpelisib mice compared with the vehicle (Figure 10, O–S, and Supplemental Figure 12C). Therefore, we explored circulating anti-dsDNA antibodies. At the initiation of the treatment, there were no significant differences between the 2 groups (Supplemental Figure 12D). However, as the treatment progressed, the alpelisib-treated group exhibited a significant reduction in circulating anti-dsDNA antibodies (Figure 10T). Additionally, we observed a significant decrease in spleen weight in the alpelisib-treated group of mice (Supplemental Figure 12E). Consistent with the results obtained in the previous mouse model, blood tests revealed that anemia and platelet count were corrected in *MRL-lpr* mice treated with alpelisib compared with the control group (Supplemental Figure 12F). Furthermore, alpelisib-treated mice demonstrated a decrease in white blood cell count, which was primarily due to reductions in lymphocytes and monocytes (Supplemental Figure 12F). Flow cytometric analysis revealed a significant reduction in B cells, both in their absolute number and percentage, within the spleen of alpelisib-treated *MRL-lpr* mice and a decrease in B cell percentage in PBMCs, bone marrow, and lymph nodes (Supplemental Figure 12G). These changes were associated with reduced serum levels of circulating proinflammatory cytokines in the alpelisib-treated group of mice (Supplemental Figure 12H). Finally, IgG, IgM, and C3 deposits were significantly reduced in glomeruli from *MRL-lpr* mice receiving alpelisib (Supplemental Figure 12, I and J).

We concluded that alpelisib exerts its effects on both podocytes and the immune compartment in *MRL-lpr* and NZBWF1/J mice.

**Figure 11:**
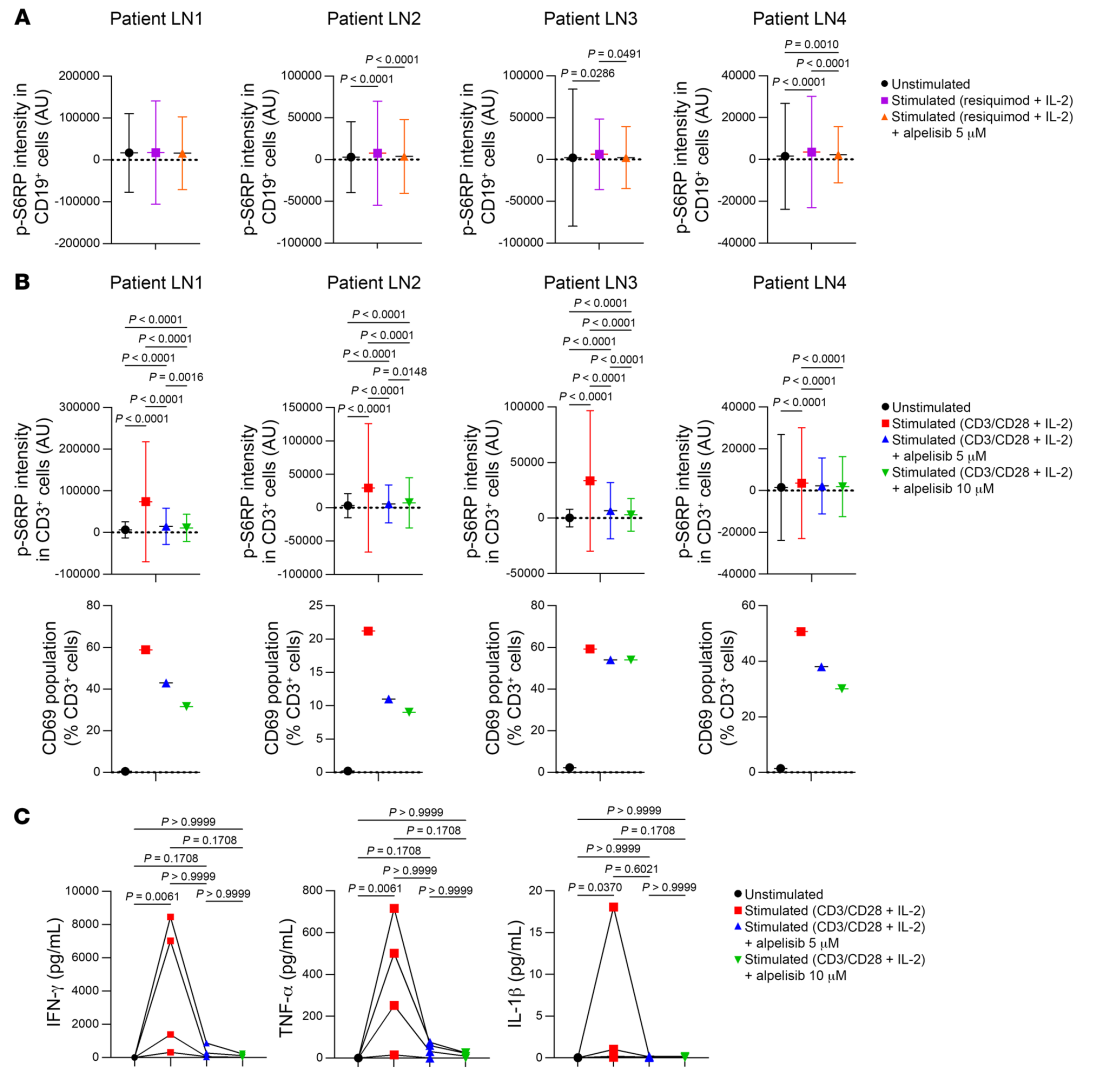
Alpelisib impaired, in vitro, activation of B and T cells from lupus patients. (**A**) p-S6RP intensity on day 1 in CD19^+^ B cells from 4 patients with active lupus nephritis (LN1–4) following stimulation and treated with vehicle or 5 μM alpelisib. LN1 unstimulated (*n =* 687 cells), LN1 stimulated (*n =* 1031 cells), LN1 stimulated + alpelisib (*n =* 668 cells), LN2 unstimulated (*n =* 6059 cells), LN2 stimulated (*n =* 7289 cells), LN2 stimulated + alpelisib (*n =* 7533 cells), LN3 unstimulated (*n =* 2623 cells), LN3 stimulated (*n =* 2442 cells), LN3 stimulated + alpelisib (*n =* 2161 cells), LN4 unstimulated (*n =* 12,542 cells), LN4 stimulated (*n =* 9224 cells), LN4 stimulated + alpelisib (*n =* 29,800 cells). (**B**) p-S6RP intensity and CD69^+^ population on day 1 among CD3^+^ T cells from LN1–4 following stimulation and treated with vehicle or alpelisib. LN1 unstimulated (*n =* 14,521 cells), LN1 stimulated (*n =* 5047 cells), LN1 stimulated + 5 μM alpelisib (*n =* 6589 cells), LN1 stimulated + 10 μM alpelisib (*n =* 7834 cells), LN2 unstimulated (*n =* 18,284 cells), LN2 stimulated (*n =* 8106 cells), LN2 stimulated + 5 μM alpelisib (*n =* 10,208 cells), LN2 stimulated + 10 μM alpelisib (*n =* 13,758 cells), LN3 unstimulated (*n =* 9066 cells), LN3 stimulated (*n =* 6176 cells), LN3 stimulated + 5 μM alpelisib (*n =* 6707 cells), LN3 stimulated + 10 μM alpelisib (*n =* 6570 cells), LN4 unstimulated (*n =* 12,542 cells), LN4 stimulated (*n =* 9224 cells), LN4 stimulated + 5 μM alpelisib (*n =* 29,800 cells), LN4 stimulated + 10 μM alpelisib (*n =* 27,863 cells). (**C**) Cytokine measurements on day 7 in supernatant from nonstimulated or stimulated CD3^+^ T cells, treated with the vehicle or 5 μM or 10 μM alpelisib. Patients LN1–4 are included in the analysis. IFN-γ, TNF-α, and IL-1β are shown here. Data presented as mean ± SD. *P* values calculated using 1-way ANOVA with Tukey’s post hoc test (**A** and **B**) or Friedman’s test with Dunn’s multiple-comparisons test (**C**).

Next, we attempted to determine the relevance of these observations in patients with LN. We obtained PBMCs from 4 patients with active class 4 LN. The treatment regimens for each patient are detailed in Supplemental Figure 13A. CD19^+^ B cells and CD3^+^ T cells were isolated from PBMCs and cultured in vitro (Supplemental Figure 13, B–E). These cells were then stimulated and exposed to either the vehicle or varying doses of alpelisib. Stimulation induced a significant level of S6RP phosphorylation in both CD19^+^ and CD3^+^ cells (Figure 11, A and B, and Supplemental Figure 13E), and it also led to the expression of CD69, an activation marker, in CD3^+^ cells (Figure 11B). Importantly, this response was observed in all patients except for CD19^+^ cells derived from patient LN 1, who received obinutuzumab, which is a humanized anti-CD20 monoclonal antibody, a few weeks prior (Figure 11A and Supplemental Figure 13A). Crucially, the treatment with alpelisib effectively suppressed S6RP phosphorylation in both types of cells and reduced CD69 expression in T cells (Figure 11, A and B). Finally, we observed that the levels of inflammatory cytokines in the supernatant of cultured CD3^+^ cells were decreased with the alpelisib treatment (Figure 11C and Supplemental Figure 14A). These findings suggest that alpelisib treatment affects both activated B and T cells in patients with lupus, which leads to a decrease in their inflammatory markers. These results support the findings observed in mouse models of LN.

## Discussion

In this work, starting from the first description to our knowledge of a patient carrying a somatic gain-of-function mutation of *PIK3CA* in glomerular epithelial cells, we demonstrated the crucial role of this pathway in podocyte proliferation, hypertrophy, and crescentic formation. scRNA-seq revealed that PI3Kα overactivation triggered developmental programs in podocytes and resulted in dedifferentiation and inflammation. Using genetic tools and pharmacological PI3Kα inhibition, we present compelling evidence supporting the targeting of this pathway as a potential treatment approach for proliferative glomerulonephritis. Furthermore, we show for the first time to our knowledge that B and T cells from lupus mouse models and from patients with LN activate the PI3Kα pathway, and treatment with alpelisib reduces the synthesis of proinflammatory cytokines.

The PI3Kα pathway is widely recognized for its ability to regulate cell growth, cell proliferation, and stemness in different cellular settings (34, 35). Notably, the activation of PI3Kα contributes to the maintenance of stemness and influences the destiny of stem cells, particularly through its interaction with the WNT pathway (36–38). Our research findings align with previous studies demonstrating that activation of the WNT pathway in podocytes may promote proliferation, dedifferentiation, and the formation of collapsing glomerulopathy lesions (39). Interestingly, we also observed a certain level of reversibility in glomerular lesions, which has been previously suggested (39, 40), and which is important when we think of the drug application for chronic kidney disease in clinics. These studies highlighted the role of mTORC1 in podocytes in physiological and disease states (41), while this work is the first devoted to PI3Kα.

Targeting podocytes as therapy for kidney disease therapy has been of great interest over recent decades, but has not been achieved (42). Rapamycin, an mTOR pathway inhibitor, has been investigated for treating various kidney diseases, but it has been mainly restricted to kidney transplantation (43–45) and tuberous sclerosis disorders (46) due to its potential nephrotoxicity (13). By acting at a higher level in the pathway, alpelisib, which is a cytostatic drug with an acceptable safety profile in patients with PROS and without described nephrotoxicity, holds promise for treating various proliferative glomerular diseases by directly targeting podocytes.

This study further presents the first evidence to our knowledge of the impact of pharmacological inhibition of PI3Kα on lymphocytes derived from lupus models and patients. In the field of leukocyte biology, the role of PI3Kα is not prominent and its inhibition in lupus, particularly compared with PI3Kδ (27–30), has not been explored until now. Surprisingly, our findings indicate that the use of alpelisib, which is not typically associated with immunosuppression (47–51), produced unexpected results. At least 2 hypotheses can be proposed to explain these discrepancies. Initially, it is possible that we observed an off-target effect of alpelisib in mouse models and ex vivo. However, alpelisib is a potent and selective inhibitor of PI3Kα and the dosages employed in our experiments were extensively investigated and demonstrated to have no impact on other isoforms (12, 47–52). Another explanation could be that in the context of inflammatory disorders like lupus, PI3Kα is upregulated and plays a significant role in cytokine production and the immune response.

In conclusion, our findings highlight that pharmacological inhibition of PI3Kα represents a very promising target for FSGS, LN, and more generally for proliferative glomerulonephritis.

## Methods

### Sex as a biological variant.

In this study, sex was considered a biological variable in 2 LN models: NZBWF1/OlaHsd and *MRL-lpr* mice. These models are known to exhibit a more pronounced incidence and severity of symptoms in females. For the NZBWF1/OlaHsd model, only females were used. In the *MRL-lpr* model, only females were used for the survival experiment. However, in other studies involving *MRL-lpr* mice and in all other mouse models, both male and female mice were included. For human studies, both males and females were included, although the incidence of lupus is higher in females.

### Animal studies.

R26StopFLP110* (stock 012343), *R26StopCAG-EGFP* (stock 006071), *Podocin*-Cre mice (stock 008523), and *Podocin*-iCre mice on the C57BL/6 background (53), *Tg26*/HIV mice (stock 022354) on the FVB/NJ background, *Pik3Ca^lox/lox^* mice (stock 017704), and MRL/MpJ-*Fas^lpr^*/J (stock 000485) were obtained from The Jackson Laboratory. NZBWF1/OlaHsd mice were obtained from Envigo. When required, at least 10 backcrossings were conducted before using mice for experiments. Mice were randomly allocated to each experimental group in a sex-, age-, and body weight–matched manner, except when indicated otherwise. For further details on mouse experiments, please refer to the Supplemental Methods.

### Histopathology and immunohistochemical analysis.

Paraffin-embedded kidney sections (4 μm) were stained with periodic acid–Schiff (PAS), Masson’s trichrome, periodic acid metenamine silver, or hematoxylin and eosin staining for histological analysis. The degree of glomerular lesions was evaluated using the following scoring system: 0 = no lesion, 1 = affecting up to 25% of the glomerulus, 2 = affecting 25%–50% of the glomerulus, 3 = affecting 50%–75% of the glomerulus, and 4 = affecting 75%–100%. At least 50 glomeruli per sample were measured for each analysis. An indirect immunoperoxidase method was used for immunohistochemistry. For further details, please refer to the Supplemental Methods. The images were captured on a Zeiss LSM 700 confocal microscope or Nikon AX confocal microscope. Immunohistochemistry revelation was performed with the appropriate horseradish peroxidase–conjugated (HRP-conjugated) antibodies and images were captured with a Nikon Eclipse E800 microscope. ImageJ software (NIH) was used for analysis. Interactive machine learning for (bio)image analysis (Ilastik, version 1.33post3) was used to select the positive signal area in the glomeruli, where its mean intensity for a target protein was measured.

### Electron microscopy analysis.

Small pieces of renal cortex (1 mm^3^) were fixed in 2.5% glutaraldehyde. For further sample processing, please refer to the Supplemental Methods. The prepared samples were examined in a JEOL 1011 transmission electron microscope with an ORIUS 1000 CCD camera (GATAN), operated with Digital Micrograph software (GATAN) for acquisition.

### Mouse cell preparation and flow cytometry.

Mononuclear cells from peripheral blood, spleen, bone marrow, and lymph nodes of mice treated with either the vehicle or alpelisib were prepared as described previously (54). For further information, please refer to the Supplemental Methods. Flow cytometry was performed using the Sony SP6800 Spectral Cell Analyzer, and data were analyzed with FlowJo software (TreeStar).

### Preparation of single-cell suspensions.

For imaging flow cytometry (Amnis ImageStream) or scRNA-seq analysis, single-cell suspensions were prepared as follows. Euthanized mice were intravenously injected with M-450 Dynabeads (Thermo Fisher Scientific, 14013) and saline. Kidneys were rinsed in PBS and cut into small pieces in RPMI 1640 (Sigma-Aldrich) media on ice. Multi Tissue Dissociation Kit 1 (Miltenyi Biotec) was used for digesting the kidney. To enrich the glomeruli, kidney pieces were first stirred in the digestion buffer for 20 minutes at 37°C. Cells were then filtered (100 μm, Miltenyi Biotec) and centrifuged 5 minutes at 300*g* and 4°C. Cell pellets were resuspended in RPMI 1640 media and rinsed. The cell pellets were again resuspended in digestion buffer and digested with the half protocol 37C_Multi_B to make the single-cell suspension. Kidney cell suspensions were filtered (70 μm, Miltenyi Biotec), centrifuged 5 minutes at 300*g* and 4°C, and resuspended in RPMI 1640 media. Cell pellets were incubated with RBC lysis buffer (Miltenyi Biotec) on ice for 3 minutes, centrifuged at 300*g* for 5 minutes at 4°C, and resuspended in 1 mL PBS with 0.04% BSA. Cell numbers and viability were analyzed under a microscope with a 0.4% trypan blue solution. The protocol was elaborated to minimize the experimental time to achieve the single-cell suspension with the maximum possible cell viability. For further scRNA-seq and ImageStream experimental and analytical procedures, please refer to the Supplemental Methods.

### Human samples.

Human samples were obtained from patients at Hôpital Necker-Enfants Malades. Clinical and biological data that were available at the time of the kidney biopsy or LN flare visit were collected. Classical histological and immunofluorescence analyses were performed with kidney biopsies fixed with formalin, alcohol, and acetic acid, which were subsequently embedded in paraffin. Some of the immunofluorescence studies and ddPCR were performed with OCT-frozen sections. Human PBMCs and LN serum were obtained from flaring patients. PBMCs were isolated from whole blood using a Ficoll-Paque gradient. Serum cytokines were measured with a V-PLEX proinflammatory panel 1 human kit (Meso Scale Discovery).

### STAR-FISH and ddPCR.

STAR-FISH (Specific-To-Alele PCR–FISH) was performed as previously described (14). The T-47D human breast tumor cell line (Sigma-Aldrich) was cultured in DMEM (Sigma-Aldrich) with 10% FCS (Sigma-Aldrich). For ddPCR, 20-μm frozen kidney sections were mounted on PEN-membrane slides (Leica) and rapidly stained with hematoxylin to recognize the kidney structure. Laser capture microdissection was performed using a Leica LMD7000 system. Pools of glomeruli and tubules were collected for each patient. ddPCR (QX200 system, Bio-Rad Laboratories) was performed using the Mutation Detection Assay (FAM + HEX, Bio-Rad Laboratories) to specifically detect the PIK3CA p.H1047R variant in the different pools, as previously described (55).

### Spatial transcriptomics.

GeoMx digital spatial profiling experiments were performed according to the Nanostring GeoMx-NGS DSP instrument manual and as previously reported (56). Mouse anti–pan-cytokeratin (clone AE1/AE3, Novus Biologicals), rabbit anti-CD10 (clone EPR22867-118, Abcam), and mouse anti-CD31 (clone JC/70A, Abcam) were used as morphology markers. For further information, please refer to the Supplemental Methods. Data analysis was performed using GeoMx DSP software v2.5.1.145. The Reactome database v78 (https://reactome.org/about/news/169-version-78-released) was used for pathway analysis.

### Statistics.

Data are expressed as the mean ± SD. Survival curves were analyzed with the log-rank (Mantel-Cox) test. Two-way ANOVA with Tukey’s post hoc test, repeated-measures 2-way ANOVA with Bonferroni’s post hoc test, or the mixed-effects model with Bonferroni’s post hoc test were used to determine the statistical significance between experimental groups. A 2-tailed Student’s *t* test, a 2-tailed Mann-Whitney *U* test, or Wilcoxon’s matched-pairs signed-rank test was used to compare 2-group experiments. One-way ANOVA with Tukey’s post hoc test or Friendman’s test with Dunn’s multiple-comparison test was used to compare 1-group experiments. A *P* value of less than 0.05 was considered significant. The statistical analyses were performed using GraphPad Prism software (v9.4.0).

### Study approval.

All animal procedures were approved by the Ministère de l’Enseignement Supérieur, de la Recherche et de l’Innovation (APAFIS nos. 20439-2018121913526398 and 30133-2020111914293579) and performed in accordance with the guidelines of Université Paris Cité to ensure animal welfare. The human studies were approved by the ethical committee (2021-A02719-32) and written informed consent was obtained from participants.

### Data availability.

The scRNA-seq and spatial transcriptomics data have been deposited in the NCBI Gene Expression Omnibus (GEO) database (GSE263909 for the former and GSE264194 for the latter). All raw data are available in the Supporting Data Values file.

## Author contributions

JY designed and evolved the study, performed most of the experiments, analyzed the data, elaborated figures, and wrote the manuscript. PI analyzed human tissue sections and was involved in the spatial transcriptomic and TEM analyses. NR and PG provided support for scRNA-seq analysis. CH was in charge of mouse experiments, including genotyping, breeding, tamoxifen administration, nephrectomy, and sacrifice. AH identified lupus nephritis patients and was in charge of their follow-up. AS was involved in TEM image acquisition and analysis. JM provided help and support for flow cytometry experiments. NG designed the algorithms for image analysis and quantification. ML, MMM, CM, MZ, and CBF provided help and support for scRNA-seq experiments. MJ and KP performed the STAR-FISH PCR experiment. JD performed the LC-MS analysis. S Baldassari and S Baulac were involved in laser capture microdissection and droplet digital PCR. CB was involved in the follow-up with patient 1. CL was involved in the follow-up with patient 1 and provided input for the manuscript. FT provided input for the manuscript. GC provided the conceptual framework, designed the study, supervised the project, elaborated figures, and wrote the manuscript.

## Supplementary Material

Supplemental data

Unedited blot and gel images

Supporting data values

## Version 1 06/06/2024

In-Press Preview

## Version 2 08/01/2024

Electronic publication
