# Supplementary material for: PIK3CA inhibition in models of proliferative glomerulonephritis and lupus nephritis
Source: J Clin Invest. 2024 Jun 6;134(15):e176402. doi: 10.1172/JCI176402 (PMC11290976; doi:10.1172/JCI176402)
Supplement: Supplemental data [file jci-134-176402-s109.pdf]

SUPPLEMENTAL MATERIALS

PIK3CA inhibition in models of proliferative glomerulonephritis and lupus nephritis

Junna Yamaguchi (MD, PhD)[1,2,3], Pierre Isnard (MD, PhD)[1,3,4], Noémie Robil (PhD)[5], Pierre de la Grange (PhD)[5], Clément Hoguin (BSc)[1,2,3], Alain Schmitt (BSc)[6], Aurélie Humel (MD)[7], Jérôme Megret (PhD)[8], Nicolas Goudin (PhD)[8], Marine Luka (PhD)[9,10], Mickael Menager (PhD)[9,10], Cécile Masson (PhD)[11], Mohammed Zarhrate (BSc)[12], Christine Bôle-Feysot (PhD)[12], Michalina Janiszewska (MD, PhD)[13], Kornelia Polyak (MD, PhD)[14,15], Julien Dairou (PhD)[1,16], Sara Baldassari (PhD)[17], Stéphanie Baulac (PhD)[17], Christine Broissand (PhD)[18], Christophe Legendre (MD, PhD)[1,3,7], Fabiola Terzi (MD, PhD)[3], Guillaume Canaud (MD, PhD)[1,2,3]

List of Supplemental Materials

Supplemental Methods

Supplemental references

Supplemental Figures 1-14

Supplemental Tables 1-2

Supplemental methods

Animal studies

For the uninephrectomy experiments, the right kidneys were removed under anesthesia. For the medical treatment of mice, 50 mg kg^{-1} alpelisib (MedChem Tronica) in 1% carboxymethylcellulose (Sigma Aldrich) + 0.5% Tween (Sigma Aldrich) or vehicle (1% carboxymethylcellulose + 0.5% Tween) was administered by oral gavage daily for the indicated time. In addition, blood and urine were obtained at the indicated times. At euthanasia, blood, urine, and kidneys were harvested. In some experiments, the spleen, heart, and bone marrow (BM) in femurs and tibias were also harvested. Tissues were fixed in 4% paraformaldehyde and embedded in paraffin for immunohistochemical analysis, snap-frozen in the Optimal Cutting Temperature (OCT), stored at −80 °C for mRNA or protein analysis, or fixed in 2.5% glutaraldehyde solution for electron microscopy analysis.

Mouse blood and urine measurements

Mouse blood counts were analyzed using a hematology analyzer (ProCyte Dx; IDEXX Laboratories). Mouse serum creatinine, blood urea nitrogen, urinary albumin, and urinary creatinine were evaluated using an AU5800 (Beckman Coulter) autoanalyzer. Serum anti-dsDNA measurement was performed according to the manufacturer's instructions using mouse anti-dsDNA ELISA kit (LBIS), and its absorbance was measured using an Infinite M Nano (TECAN). Serum cytokine measurements were performed according to the manufacturer's instructions using the V-PLEX® proinflammatory panel 1 mouse kit (Meso Scale Discovery, MSD).

Histopathology and immunohistochemistry analysis

For paraffin embedded sections, after deparaffinization, antigen retrieval was performed with citrate buffer (pH6) or Tris-EDTA buffer (pH9 or pH6) using a microwave, pressure cooker, or high temperature (95 °C). The endogenous peroxidase activity was quenched using 3% hydrogen peroxide; nonspecific protein binding was blocked using 2.5% normal horse serum (Vector Laboratories), and the endogenous biotin activity was quenched using the Avidin/Biotin Blocking Kit (Vector Laboratories). When using mouse primary antibodies on mouse tissue, Klear mouse blocking reagent (Diagomics) was used to block endogenous mouse immunoglobulins. After blocking, the tissue sections were incubated with the primary antibodies overnight at 4 °C. The following primary antibodies were used: rabbit anti-Ki-67 antibody (SP6; Thermo Fisher Scientific),

rabbit anti–P-S6RP antibody (D68F8; Cell Signaling Technology), mouse anti–P-AKT (Ser^{473}) antibody (587F11; Cell Signaling Technology), rabbit anti–P-AKT (Thr^{308}) antibody (C31E5E; Cell Signaling Technology), mouse anti-S6RP antibody (54E2; Cell Signaling Technology), chicken anti-GFP antibody (ab13970; Abcam), guinea pig anti-Nephrin antibody (GP-N2; Progen), rabbit anti-Podocin antibody (P0372; Sigma Aldrich), mouse anti-Nestin antibody (4D11, Novus Biologicals), mouse anti-synaptopodin antibody (G1D4, Progen), rat anti-CD44 antibiody (IM7, BD Biosciences), and mouse anti-WT1 antibody (6F-H2; DAKO). The corresponding secondary antibodies, including anti-mouse IgG (Thermo Fisher Scientific), anti-rabbit IgG (Thermo Fisher Scientific), anti-guinea pig IgG (Thermo Fisher Scientific), anti-chicken IgG (Abcam), biotinylated anti-rabbit IgG (Vector Laboratories), and biotinylated anti-mouse IgG (Vector Laboratories) were applied. For the Avidin/Biotin detection, an R.T.U. Vectastain Kit (Vector Laboratories) or streptavidin (Thermo Fisher Scientific) was used. The immunoreactive antigen sites were detected with hydrogen peroxide and diaminobenzidine. For alkaline phosphatase detection, alkaline phosphatase conjugate streptavidin (Thermo Fisher Scientific) and BCIP/NBT Substrate Kit (Vectpr Laboratories) were used. For frozen immunofluorescent staining, frozen tissue sections (4 μm) were briefly air dried and fixed in 50% methanol/50% acetone. Then, sections were blocked with 2.5% normal horse serum and incubated overnight at 4 °C with the following primary antibodies: guinea pig anti-Nephrin (GP-N2; Progen), rabbit anti-Ki-67 (SP6; Thermo Fisher Scientific), chicken anti-GFP antibody (ab13970; Abcam), rat anti-PDGFRβ (APB5, eBioscience™), rat anti-CD3 (17A2, eBioscience™), or rat anti-F4/80 (A3-1, Bio-Rad Laboratories), or mouse anti–P-AKT (Ser^{473}) antibody (587F11; Cell Signaling Technology). The sections were then incubated at room temperature for 40 min with the following secondary antibodies: fluorescein isothiocyanate–conjugated (FITC) anti-mouse IgG (Sigma Aldrich), FITC anti-mouse IgM (BD Biosciences), FITC anti-mouse C3 (Abcam) or the corresponding secondary antibodies for the primary antibodies including anti-mouse IgG (Thermo Fisher Scientific), anti-rabbit IgG (Thermo Fisher Scientific), anti-guinea pig IgG (Thermo Fisher Scientific), biotinylated anti-rabbit IgG (Vector Laboratories), and anti-chicken IgG.

Electron microscopy analysis

The fixed samples were washed 3 times in PBS. Samples were postfixed in 1% osmium tetroxide 0.1M (Electron Microscopy Science, UK) in 0.1 M Phosphate Buffer (PB) (pH 7.4). Samples were washed 3 times in H_2O. Samples were dehydrated in alcohol grades :70% Ethanol 10 min, 90% Ethanol 10min, 100% Ethanol 3x15min, 100% Propylene oxide (Electron Microscopy Science, UK) 2X5min. Epikote 812 was prepared with

these proportions: 12.5 mL Embed 812, 7.5 mL Araldite 502, 27 mL DDSA, 1.3 mL DMP30 (kit (Electron Microscopy Science, UK). Resin infiltration was performed as following: mix 1:1 Epikote 812 : propylene oxide 30 min followed by mix 1:2 Epikote 812 : propylene oxide overnight room temperature. Samples were washed in 100% Epikote 812 then embedded in 4mm gelatine capsules in 100% Epikote 812, and Polymerised in 60ºC oven for 24hours. Ultrathin sections were cut at 90 nm with a Leica UFC7 ultramicrotom (Leica Microsystems GmbH, Germany) and deposed on Gilder grids 200 mesh (Electron Microscopy Science, UK), They were counterstained with uranyl acetate 7% (LFG, France) and Reynold's lead citrate (LFG, France).

cDNA synthesis and quantitative RT-PCR analysis

Total RNA in the kidney cortex was extracted using NucleoSpin RNA (Macherey Nagel). Complementary DNA was reverse-transcribed using a TaqMan high-capacity cDNA RT kit (Thermo Fisher Scientific). qPCR was performed with iTaq universal SYBR Green Supermix (Bio-Rad Laboratories) using a CFX Connect real-time system (Bio-Rad Laboratories). The expression levels were analyzed using the delta-delta Ct method. *Hypoxanthine phosphoribosyltransferase* (*Hprt*) was used as the normalization control.

Western blotting

Protein extracts in RIPA buffer from the kidney cortex were separated by SDS–PAGE, transferred onto the membrane and incubated with antibodies, and followed by the appropriate peroxidase-conjugated secondary antibody incubation. The following primary antibodies were used: rabbit anti–P-S6RP antibody (D68F8; Cell Signaling Technology), rabbit anti–P-AKT (Ser^{473}) antibody (D9E; Cell Signaling Technology), rabbit anti–P-AKT (Thr^{308}) antibody (C31E5E; Cell Signaling Technology), mouse anti-S6RP antibody (54E2; Cell Signaling Technology), mouse anti-Akt (pan) antibody (40D4, Cell Signaling Technology), chicken anti-GFP antibody (ab13970; Abcam), mouse anti-alpha-tubulin antibody (B-5-1-2; Sigma Aldrich), rabbit anti-Nephrin antibody (29070; IBL), and rabbit anti-Podocin antibody (P0372; Sigma Aldrich). The chemiluminescence was acquired using ChemiDoc MP (Bio-Rad Laboratories) and densitometry was performed using Image Lab software (Bio-Rad Laboratories, version 6.0.1).

Flow cytometry

For lymphocyte, granulocyte and monocyte lineage analysis, anti-CD3a antibody (500A2; Becton Dickinson), anti-CD4 antibody (L3T4; Becton Dickinson), anti-CD8a antibody (63-6.72; Becton Dickinson), anti-B220

antibody (RA3-6B2; Becton Dickinson), anti-CD11b antibody (M1/70; Becton Dickinson), anti-Ly6G antibody (RB6-8C5; Becton Dickinson), and CD16/CD32 (2.4G2; Becton Dickinson) were used. Cells were resuspended in 0.2 to 1 mL of PBS +2%FCS + 2 µL of 7-aminoactinomycine D (7-AAD, Thermo Fischer Scientific).

Imaging flow cytometry (Amnis ImageStream)

Following the single-cell suspension procedure, flow cytometry cell preparation was conducted by essentially following the Flow Cytometry Protocol by Cell Signaling Technology. Briefly, cell samples were fixed with 4% PFA for 15 min, permeabilized with methanol on ice, and resuspended in PBS after several washes. The samples were labeled with rabbit anti-P-S6RP-AF647 antibody (D68F8; Cell Signaling Technology) and/or rabbit anti-P-Akt^{Thr308}-PE antibody (D25E6; Cell Signaling Technology). Rabbit IgG isotype controls (AF647 and PE; Cell Signaling Technology) were used. Resuspended cells were run on an ImageStream ISX mkII (Amnis) that combines flow cytometry with detailed cell imaging. Magnification (40×) was used for all acquisitions. Data were acquired with INSPIRE software (Amnis) and analyzed with IDEAS software (v.6.2, Amnis).

Single-cell RNA-seq barcoding and cDNA synthesis

The scRNA-seq libraries were generated using a Chromium Single Cell 3’ Library & Gel Bead Kit v.3 (10x Genomics) according to the manufacturer’s protocol. Briefly, suspended single-cells were counted, diluted at 1,000 cells/µL in PBS + 0.04% BSA, and 20,000 cells were loaded in the 10x Chromium Controller to generate single-cell gel-beads in an emulsion. After reverse transcription, the gel-beads in the emulsion were disrupted. Then, barcoded complementary DNA was isolated and amplified by PCR. Following fragmentation, end repair and A-tailing, sample indexes were added during index PCR. An equimolar pool of the 4 individual 10X Genomics Single Cell Expression 3' V3 libraries was prepared by the Imagine Genomic Core Facility and sequenced on an S2 FlowCell using the NovaSeq6000, Illumina (sequencing mode Paired-End 100+100 bases + indexes). A total of 2 billion reads were targeted for this pool of 4 libraries (500 million reads per library).

Single-cell RNA-seq pre-processing

Sequencing data quality analysis was performed using FastQC v0.11.2 on 4 samples. For read alignment and quantification of unique molecular identifiers (UMI), CellRanger software v3.0.2 was used on Mus Musculus genome mm10 with default parameters and gene annotation from Ensembl 100 (plus eGFP and tdTomato). The 4 expression matrices containing the UMI counts were merged, and only the genes with UMI ≥ 1 in at least one cell were kept. The following filters were applied to generate a global matrix used in further analysis: cells with UMI ≥ 2000, number of detected genes ≥ 600, and cells with UMI in mitochondrial genes ≤ 60%. For the normalization of UMIs, Seurat 3.1.1 was used(1) and a global-scaling normalization method was applied with a scale factor of 10,000 and log-transformation of data. This was followed by a scaling linear transformation step to avoid highly-expressed genes with a higher weight in downstream analysis.

Clustering and marker genes

PCA was performed on the scaled data with a Jackstraw plot to choose how many PCs to retain as an input for the Seurat clustering step. The clustering step was performed using default parameters, the Louvain algorithm as the clustering method, and 15 PC and a resolution parameter defining the cluster granularity set to 0.5. Marker genes defining each cluster were found via differential expression testing with a Wilcoxon rank sum test and a log fold change threshold of 1.

Trajectory analysis

The Monocle single-cell trajectory was constructed using M3Drop(2) and Monocle 2.10.1(3). Input genes for the Monocle trajectory construction were selected using an unsupervised approach via the M3Drop result, which identifies differentially expressed genes based on a Michaelis-Menten function for the relationship between mean expression and dropout rate with the relevant genes being the ones shifting above a fitted curve. The default Monocle workflow was then performed to generate the trajectories.

Enrichment analysis

An analysis for enriched KEGG pathways and Gene Ontology (GO) terms was performed using the WebGestaltR package(3) on databases from the Mus Musculus organism. GO terms and pathways were considered enriched if fold enrichment was ≥ 2.0, the uncorrected p-value was ≤ 0.05 and a minimum number of regulated genes in pathway/term ≥ 2.0 was achieved.

LC-MS analysis of alpelisib

Each tissue type was homogenized in 100% cold methanol with a tissue-to-solvent ratio of 1-mg tissue to 5-μl methanol. After sonication for 20 s, the tissue extract was centrifuged at 13,000 × g for 30 min and then injected onto a Phenomenex Kinetex XB-C18 HPLC column (100 mm by 2.1 mm) at 45 °C. Alpelisib was analyzed by reverse-phase HPLC (Shimadzu LC-MS system 8040 interfaced with LabSolutions software). Twenty micrograms of tissue extracts were injected onto a column, and the mobile phase used for the separation consisted of two eluents as follows: Solvent A was 0.1% formic acid in ddH2O, and solvent B was acetonitrile with 0.1% of formic acid. The compounds were separated using the following discontinuous gradient at a flow rate of 0.6 ml/min: the initial concentration of 20% in solvent B increased to 70% over 6 min, and this was followed by a decrease to 20% over the next minute and the initial conditions were then maintained for 14 min. The alpelisib was monitored spectrophotometrically by absorbance (photodiode detector) from 205 to 600 nm and by tandem mass detection. The mass measurement was implemented in positive ion mode using multiple reaction monitoring (MRM) with an electrospray ionization source. Three MRM transitions for alpelisib are used as follows: 442.1 > 328.0, 442.1 > 288.0, and 442.1 > 115.1. Quantification was conducted by integration of the peak absorbance area using a calibration curve established with various known concentrations of alpelisib.

Spatial transcriptomics

Briefly, 4 μm of AFA-fixed paraffin-embedded human samples were baked overnight at 37 °C and 1 hour at 65 °C, and then they were processed on a Leica automation platform with a protocol including three major steps as follows: 1) slide baking, 2) antigen retrieval for 20min at 100 °C, and 3) 1.0ug/ml proteinase K treatment for 15min. After taking the slide off of the Leica, they were incubated with a GeoMx WTA assay probe cocktail overnight. The following day, the slides were washed and applied with morphology marker incubation before loading onto the GeoMx machine. On the GeoMx machine, slides were fluorescently scanned and finished ROI selection. Barcodes were collected and subsequent barcoding readings were performed on an Illumina NGS platform. Individual counts were normalized against the 75th percentile of the signal from their own ROI (Q3 Normalization).

Human PBMC flow cytometry experiment and supernatant cytokine measurement

PBMC were sorted for CD3+ T cells and CD19+ B cells with anti-CD3 (clone UCHT1, Sony Biotechnology), anti-CD19 (clone HIB19, Sony Biotechnology), and 7-AAD using BD FACS Aria II SORP. CD3+ T cells or CD19+ B cells were incubated in RPMI 1640 media (Thermo Fischer Scientific) containing 10% FBS, 2mM glutamine, 100 U/ml penicillin and streptomycin (Thermo Fischer Scientific). To stimulate T cells, Dynabeads™ Human T-Activator CD3/CD28 (Thermo Fischer Scientific, ref. 11161D) and 1000 U/mL recombinant human interleukin-2 (IL-2) (Proteintech, ref. 200-02) were used. To stimulate B cells, 2.5 ug/ml resiquimod (Sigma Aldrich, ref. SML0196) and 1000 U/mL recombinant human IL-2 were used. For in vitro experiments, alpelisib was dissolved in DMSO (5mM) as a stock solution and T cells or B cells were pre-treated with the indicated concentration of alpelisib for 30 min before starting the stimulation or left unstimulated. CD19+ B cells were collected 16hr after stimulation and treatment, while CD3+ T cells were collected either 16hr or 7 days after stimulation and treatment. Upon cell collection, the supernatants were also collected. Cells were fixed and permeabilized using BD Cytofix (BD Biosciences, ref. 554655) and Perm Buffer III (BD Biosciences, ref. 558050), followed by intracellular staining with P-S6RP (lone D68F8, Cell Signaling Technologies). CD69 (clone FN50, Sony Biotechnology) was used as activation marker. The flow cytometry analysis was performed with a Sony ID7000™ spectral cell analyzer. Data were analyzed with FlowJo software (TreeStar). Supernatant cytokines were analyzed with a V-PLEX® proinflammatory panel 1 human kit (Meso Scale Discovery, MSD).

Supplemental references

1. Butler A, Hoffman P, Smibert P, Papalexi E, and Satija R. Integrating single-cell transcriptomic data across different conditions, technologies, and species. *Nature biotechnology.* 2018;36(5):411-20.
2. Andrews TS, and Hemberg M. M3Drop: dropout-based feature selection for scRNASeq. *Bioinformatics.* 2019;35(16):2865-7.
3. Qiu X, Mao Q, Tang Y, Wang L, Chawla R, Pliner HA, et al. Reversed graph embedding resolves complex single-cell trajectories. *Nature methods.* 2017;14(10):979-82.

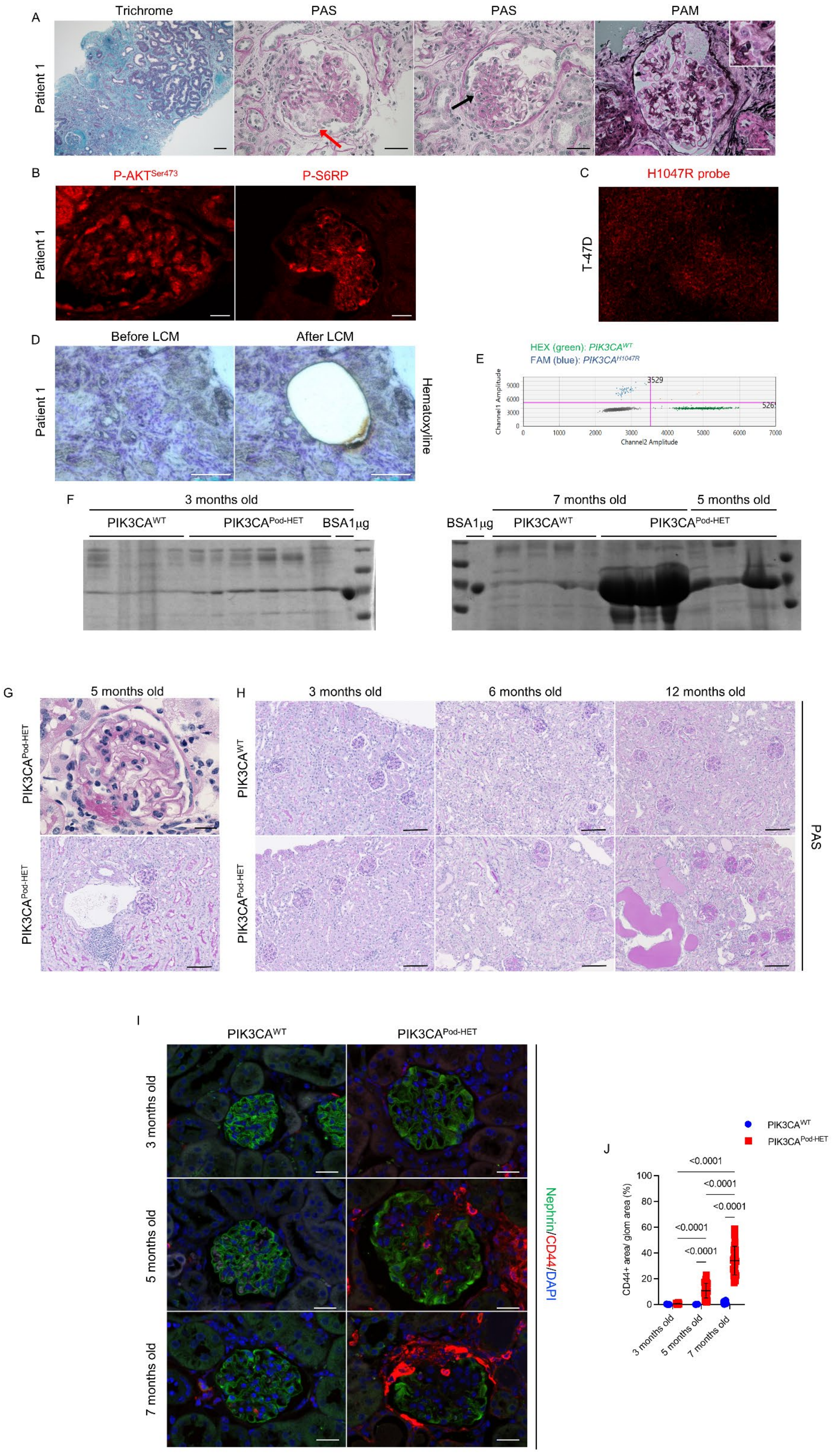

Supplemental Figure 1

Supplemental Figure 1: *PIK3CA* gain-of-function mutation in podocytes leads to severe glomerular disease. (**A**) Trichrome, PAS, and silver staining (PAM) of the kidney biopsy of patient 1. Scale bar 100 μm for Trichrome and 40 μm for PAS and PAM. Black arrow showing hypercellularity, and red arrow showing hypertrophy and hyperplasia of the overlying podocytes. (**B**) P-AKT^{Ser473} and P-S6RP immunofluorescence staining of the kidney biopsy of patient 1. Scale bar 20 μm. (**C**) *In situ H1047* hybridization of the kidney biopsy of T-47D. (**D**) Hematoxylin staining for glomeruli laser capture microdissection of the kidney biopsy from patient 1. Left: before cutting and right: after cutting. Scale bar 100 μm. (**E**) Droplet digital PCR result of the glomeruli from the kidney biopsy of patient 1. Channel 1: *H1047R* Mutant probe and Channel 2: *Wild Type* (WT) probe. (**F**) Representative Coomassie blue staining of 3-, 5-, and 7-month-old $PIK3CA^{WT}$ and $PIK3CA^{Pod\text{-}HET}$ mice. Bovine serum albumin (BSA) is shown as a positive control. (**G**) Another PAS staining of 5 months old $PIK3CA^{Pod\text{-}HET}$ mice. Scale bar 25 μm for upper panel, 100 μm for lower panel. (**H**) Representative PAS staining of 3-, 6-, and 12-month-old $PIK3CA^{WT}$ and $PIK3CA^{Pod\text{-}HET}$ mice. Scale bar 100 μm. (**I**) Representative immunofluorescence staining of Nephrin and CD44 in kidneys from $PIK3CA^{WT}$ and $PIK3CA^{Pod\text{-}HET}$ mice, at 3-, 5- and 7-months old. Scale bar 20 μm. (**J**). CD44+/ glomerulus area (%) quantification (3-m.o.-$PIK3CA^{WT}$, n=4, 3-m.o.-$PIK3CA^{Pod\text{-}HET}$, n=5, 5-m.o.-$PIK3CA^{WT}$, n=5, 5-m.o.-$PIK3CA^{Pod\text{-}HET}$, n=5, 7-m.o.-$PIK3CA^{WT}$, n=4, 7-m.o.-$PIK3CA^{Pod\text{-}HET}$, n=5). Data are means ± SD. P values are calculated using two-way ANOVA with Tukey's post hoc test.

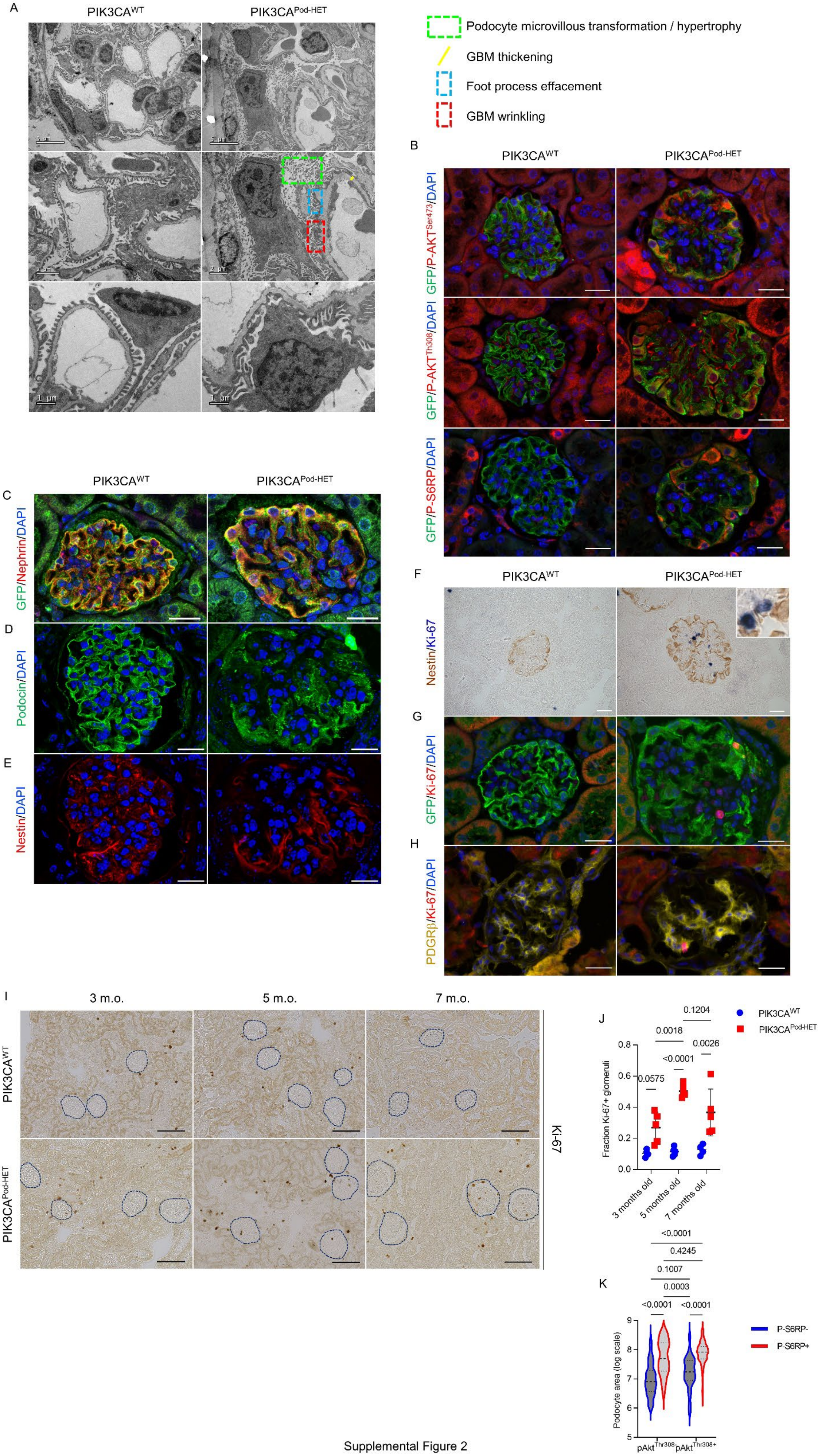

Supplemental Figure 2

Supplemental Figure 2: *PIK3CA$^{Pod-HET}$* mice characterization. (**A**) Representative images of transmission electron microscopy of kidneys from *PIK3CA^{WT}* and *PIK3CA$^{Pod-HET}$* mice at 5 months old. (**B**) Representative coimmunofluorescence staining of P-AKT^{Ser473}, P-AKT^{Th308}, P-S6RP and GFP in kidneys from 5-month-old *PIK3CA^{WT}* and *PIK3CA$^{Pod-HET}$* mice. Scale bar 20 μm. (**C**) Representative coimmunofluorescence staining of GFP and Nephrin in kidneys from *PIK3CA^{WT}* and *PIK3CA$^{Pod-HET}$* mice. Scale bar 20 μm. (**D**) and (**E**) Representative coimmunofluorescence staining of Podocin and Nestin in kidneys from 5-month-old *PIK3CA^{WT}* and *PIK3CA$^{Pod-HET}$* mice. Scale bar 20 μm. (**F-G**) Representative coimmunostaining of Nestin and Ki-67 (**F**), GFP and Ki-67 (**G**), PDGFRβ and Ki-67 (**H**) in kidneys from 5-month-old *PIK3CA^{WT}* and *PIK3CA$^{Pod-HET}$* mice. Scale bar 20 μm. (**I**) Representative Ki-67 immunostaining of kidneys from *PIK3CA^{WT}* and *PIK3CA$^{Pod-HET}$* mice at the age of 3, 5 and 7 months. Scale bar 100 μm. and (**J**) Quantification of fraction Ki-67+ glomeruli from *PIK3CA^{WT}* and *PIK3CA$^{Pod-HET}$* mice (3-m.o.-*PIK3CA^{WT}*, n=4, 3-m.o.- *PIK3CA$^{Pod-HET}$*, n=5, 5-m.o.-*PIK3CA^{WT}*, n=5, 5-m.o.-*PIK3CA$^{Pod-HET}$*, n=5, 7-m.o.-*PIK3CA^{WT}*, n=4, 7-m.o.-*PIK3CA$^{Pod-HET}$*, n=5). (**K**) Amnis ImageStream analysis of isolated podocytes from 4-month-old *PIK3CA^{WT}* and *PIK3CA$^{Pod-HET}$* mice (n=2 mice per group). GFP+ podocytes were labelled with P-AKT^{Thr308} and P-S6RP. Data are means ± SD. P values are calculated using two-way ANOVA with Tukey's post hoc test (**J**, **K**).

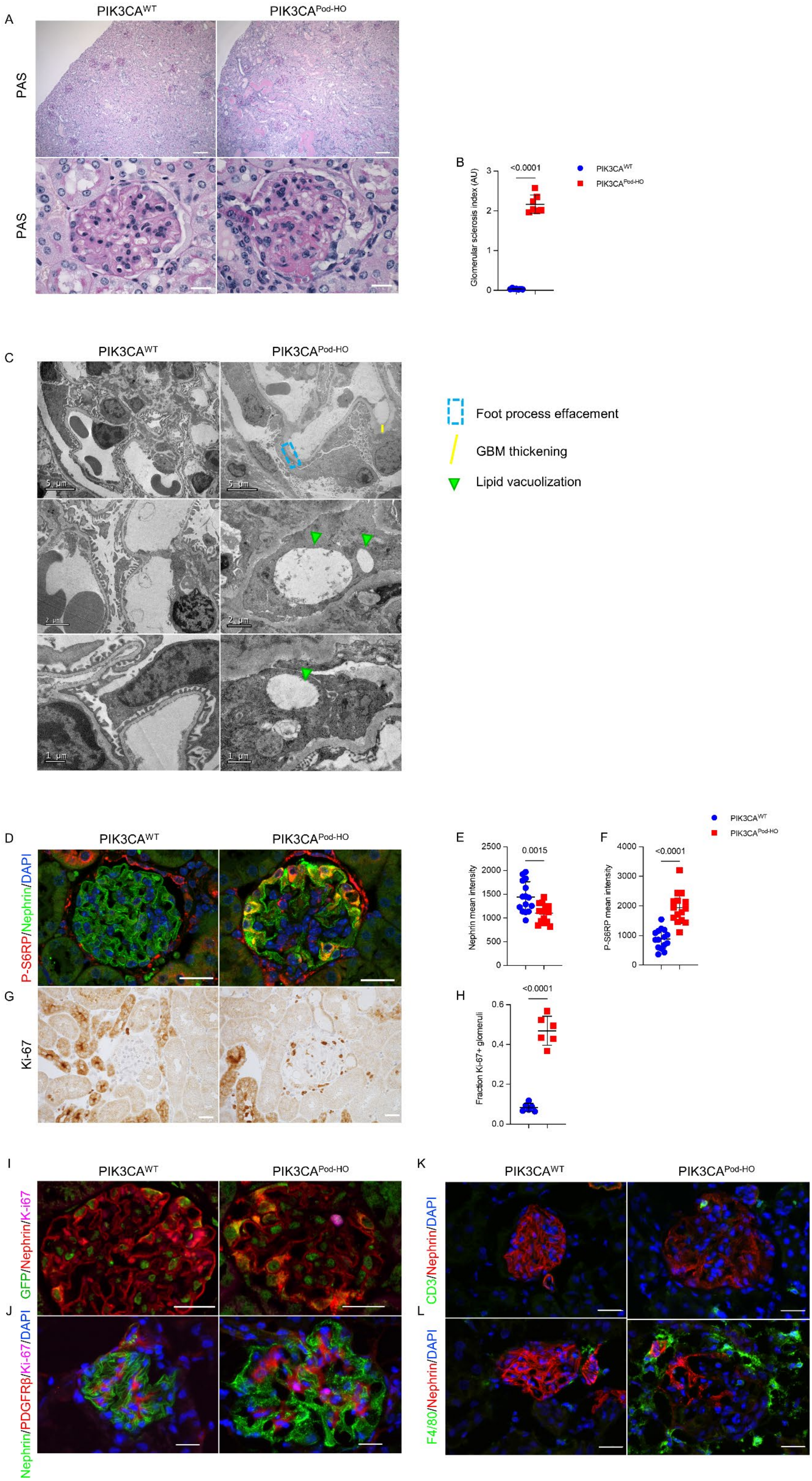

Supplemental Figure 3

Supplemental Figure 3: *PIK3CA$^{Pod\text{-}HO}$* mice characterization. (**A**) Representative PAS staining and (**B**) GS index quantification from 3-month-old *PIK3CA^{WT}* and *PIK3CA$^{Pod\text{-}HO}$* mice kidneys (n=6 per group). (**C**) Representative images of transmission electron microscopy of kidneys from *PIK3CA^{WT}* and *PIK3CA$^{Pod\text{-}HO}$* mice at 4 months old. (**D**-**L**) 3-month-old *PIK3CA^{WT}* and *PIK3CA$^{Pod\text{-}HO}$* mice kidneys (n=6 per group). (**D**) P-S6RP and Nephrin coimmunofluorescence staining, and quantification in glomeruli (**E**, **F**). (**G**) Ki-67 immunostaining and (**H**) quantification. (**I**-**L**) Coimmunofluorescence stainings in kidneys from 2-month-old *PIK3CA^{WT}* and *PIK3CA$^{Pod\text{-}HO}$* mice. (**I**) Representative coimmunofluorescence staining of GFP, Nephrin and Ki-67, (**J**) Representative coimmunofluorescence staining of Nephrin, PDGFRβ and Ki-67, (**K**) Representative coimmunofluorescence staining of CD3 and Nephrin**,** (**L**) Representative coimmunofluorescence staining of F4/80 and Nephrin. Values are means ± SD. *P* values calculated using two-tailed Mann-Whitney U test (**E**, **F**); or two-tailed *t* test (**B**, **H**). Scale bars: 130 μm (**A** upper), 32 μm (**A** lower), 20 μm (**D**, **I**-**L**), 32.2 μm (**G**).

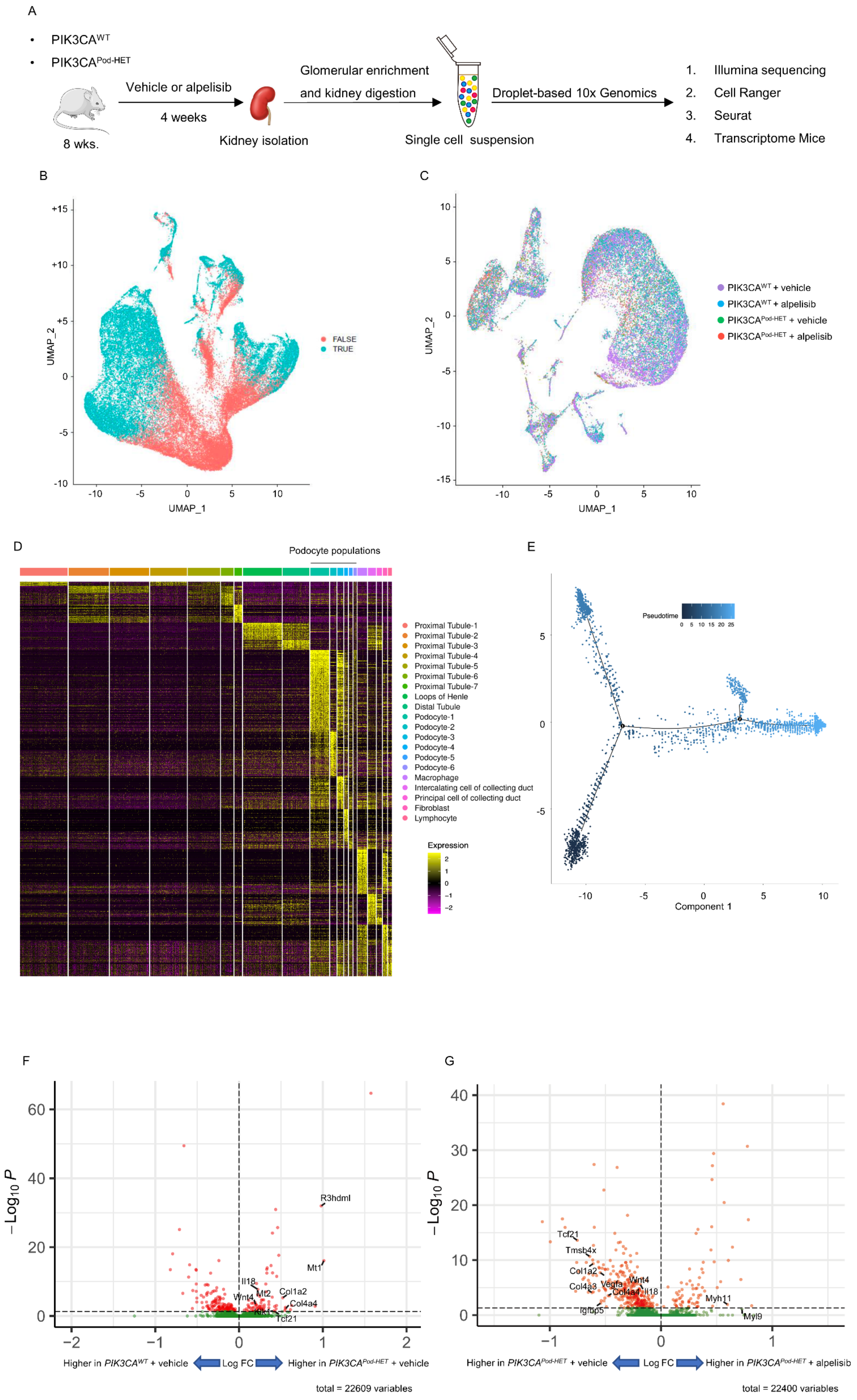

Supplemental Figure 4

Supplemental Figure 4: *PIK3CA* gain-of-function mutation in podocytes is associated with changes in cell fate determination. (**A**) Schematic of single-cell RNA sequencing. Eight-week-old $PIK3CA^{WT}$ and $PIK3CA^{Pod\text{-}HET}$ mice were treated for 4 weeks with either the vehicle or alpelisib (n=2 mice per group). (**B**) UMAP plot showing all cells from glomerular enriched kidneys before filtering from 8 mice. Cells with unique molecular identifiers (UMIs) over 2000, with genes over 600, and a mitochondrial gene expression percentage (MT%) less than 60 were chosen as filtering criteria. The cells that passed this filtering criteria are colored in green (TRUE), and those did not pass the criteria are colored in red (FALSE). (**C**) UMAP plot showing all of the cell distributions after filtering. No batch effect was observed. (**D**) Heatmap of strongly expressed genes in each cluster. Each column represents a cell and each row represents a gene. The color scheme is based on the expression level of the gene. (**E**) Cell trajectory map of podocyte clusters (1-6) showing the pseudo-time. (**F**) Volcano plots showing the differentially expressed genes between two samples ($PIK3CA^{Pod\text{-}WT}$-vehicle vs. $PIK3CA^{Pod\text{-}HET}$-vehicle). Red dots are the genes that are differentially expressed ($-\log_{10}$ adjusted *p*-value > 1.3). (**G**) Volcano plots showing the differentially expressed genes between two samples ($PIK3CA^{Pod\text{-}HET}$-vehicle vs. $PIK3CA^{Pod\text{-}HET}$-alpelisib). Red dots are the genes that are differentially expressed ($-\log_{10}$ adjusted *p*-value > 1.3).

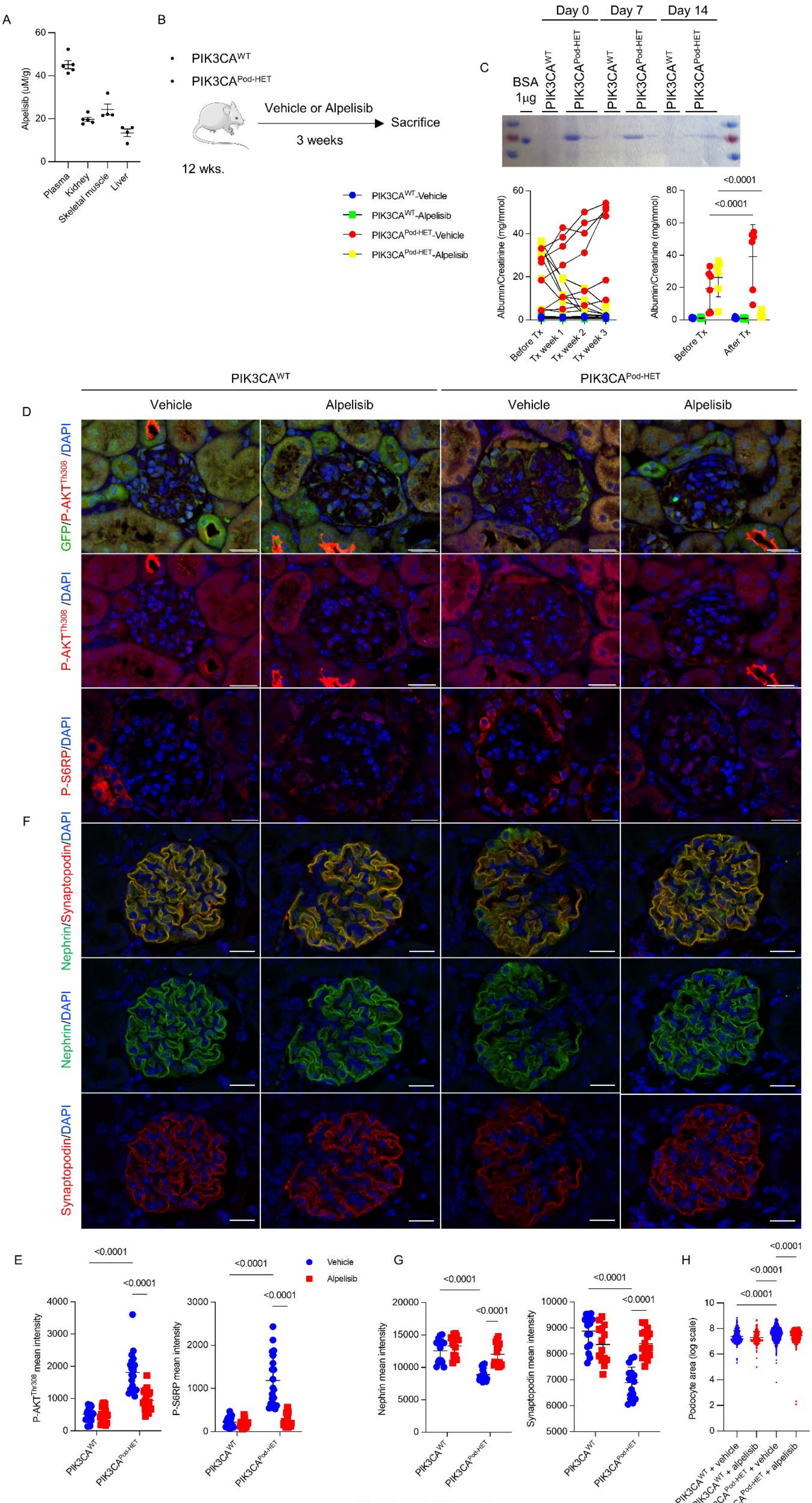

Supplemental Figure 5

Supplemental Figure 5: *PIK3CA* gain-of-function mutation in podocytes leads to progressive glomerular disease. (**A**) Alpelisib concentrations (μM/g) in mice plasma, kidneys, skeletal muscle, and liver were measured by LC-MS analysis (n=5 mice for plasma and kidney, n=4 for skeletal muscle and liver). (**B**) Experimental protocol design. (**C**) Representative Coomassie blue staining of $PIK3CA^{WT}$ and $PIK3CA^{Pod\text{-}HET}$ mice during treatment with either the vehicle or alpelisib and below, quantification (n=6 per group). Bovine serum albumin (BSA) is shown as a positive control. The trajectory of each mouse urinary albumin/creatinine is shown in the below left graph. Below right graph are means ± SD. P values are calculated using two-way ANOVA with Tukey's post hoc test. The values for Before Tx and After Tx (= Tx week 3) are shared between the 2 graphs. (**D**) Upper two panels show representative coimmunofluorescence staining between GFP and P-AKT^{Thr308} in the kidneys from $PIK3CA^{WT}$ and $PIK3CA^{Pod\text{-}HET}$ mice treated with either the vehicle or alpelisib. Note the second panel is the split channel panel of the top panel. The lowest panel shows the representative immunofluorescence staining of P-S6RP in kidneys from $PIK3CA^{WT}$ and $PIK3CA^{Pod\text{-}HET}$ mice treated with either the vehicle or alpelisib. Scale bar 20 μm**.** (**E**) Glomerular P-AKT^{Thr308} and P-S6RP mean intensity quantification (n=6 mice per group). (**F**) Upper panel shows representative coimmunofluorescence staining between Nephrin and Synaptopodin in the kidneys from $PIK3CA^{WT}$ and $PIK3CA^{Pod\text{-}HET}$ mice treated with either the vehicle or alpelisib. The lowest two panels show representative immunofluorescence staining of Nephrin or Synaptopodin in kidneys from $PIK3CA^{WT}$ and $PIK3CA^{Pod\text{-}HET}$ mice treated with either the vehicle or alpelisib. The lowest two panels are split channel images of the top panel images. Scale bar 20 μm**.** (**G**) Glomerular Nephrin and Synaptopodin mean intensity quantification (n= 6 mice per group). (**H**) Amnis ImageStream analysis of isolated podocytes of $PIK3CA^{WT}$ and $PIK3CA^{Pod\text{-}HET}$ mice treated with either the vehicle or alpelisib (n=3 mice per group). Data are represented as means ± SD. P values calculated using two-way ANOVA with Bonferroni's post hoc test (**C** right), and two-way ANOVA with Tukey's post hoc test (**E**, **G**, and **H**).

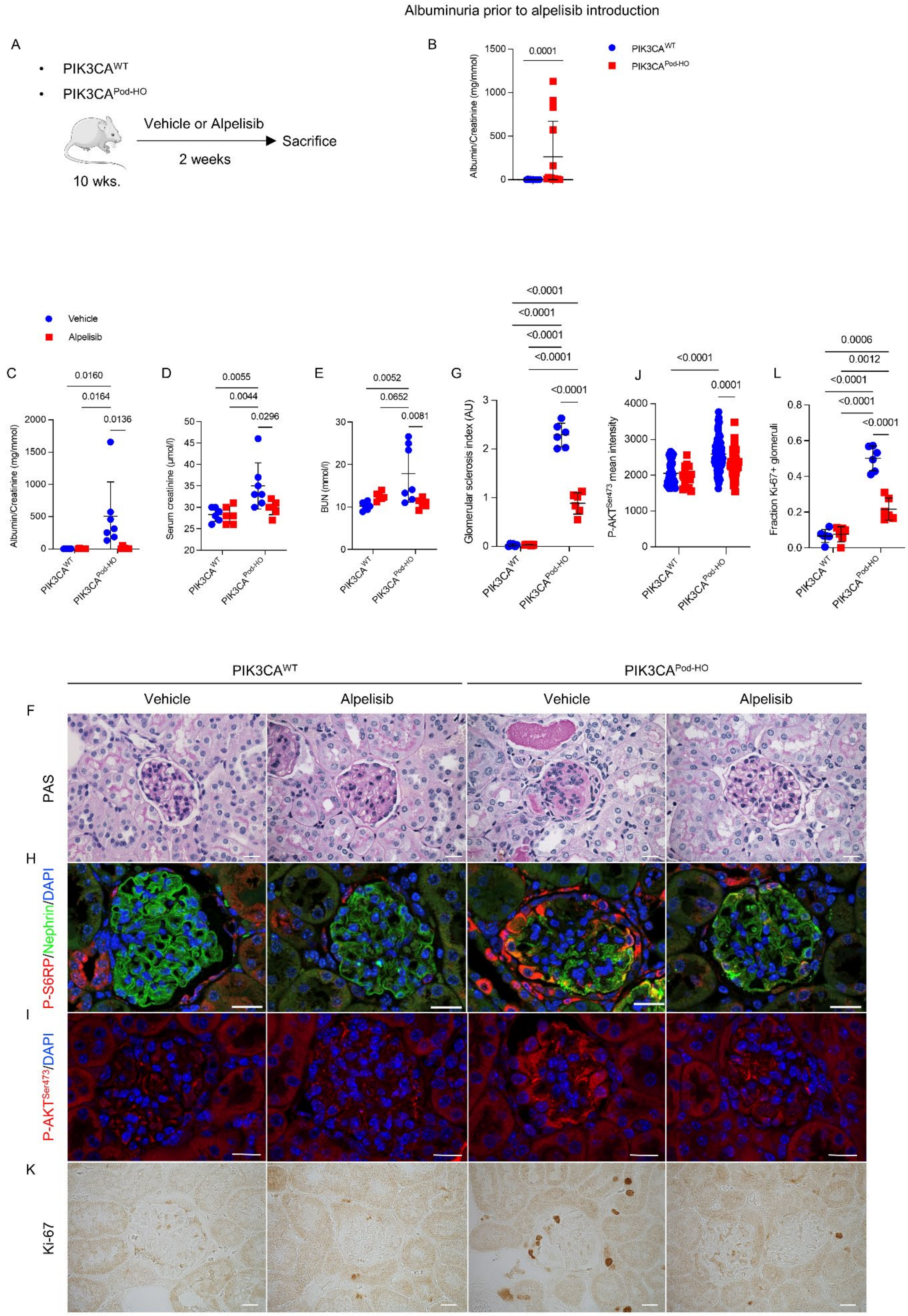

Supplemental Figure 6

Supplemental Figure 6: Allele dose effect of *PIK3CA* gain-of-function mutation in podocytes and sensitivity to alpelisib. (**A**) Design of the experimental protocol. (**B**) Urinary albumin to creatinine ratio of *PIK3CA*^{WT} and *PIK3CA*$^{Pod\text{-}HO}$ mice before the start of treatment. (n=11 for *PIK3CA*^{WT}, n=14 for *PIK3CA*$^{Pod\text{-}HO}$ mice). **C**-**L** are of *PIK3CA*^{WT} and *PIK3CA*$^{Pod\text{-}HO}$ mice at the time of sacrifice (2 weeks after treatment with either the vehicle or alpelisib) (n=6 for *PIK3CA*^{WT}-Vehicle and *PIK3CA*^{WT}-Alpelisib, n=7 for *PIK3CA*^{HO}-Vehicle and *PIK3CA*^{HO}-Alpelisib, except otherwise stated). (**C**) Urinary albumin to creatinine ratio, (**D**) Serum creatinine, (**E**) BUN, (**F**) Representative PAS staining of kidneys (Scale

bar 32.2 μm), (**G**) GS index quantification (n=6 per group) (**H**) Representative coimmunofluorescence staining between P-S6RP and Nephrin in kidneys (Scale bar 20 μm), (**I**) Representative P-AKT^{Ser473} immunofluorescence staining in kidneys (Scale bar 20 μm). (**J**) Quantification of mean P-AKT^{Ser473} intensity in glomeruli, (**K**) Representative Ki-67 immunostaining (Scale bar 32.2 μm), (**L**) Quantification of Ki-67+ fraction (n=6 per group). Values are the means ± SD and representative of 3 independent experiments (**B**, **C**, **D**, **E**, **G**, **J**, and **L**). P values calculated using two-way ANOVA with Tukey's post hoc test (**C**, **D**, **E**, **G**, **J**, and **L**); two-tailed Mann-Whitney U test (**B**).

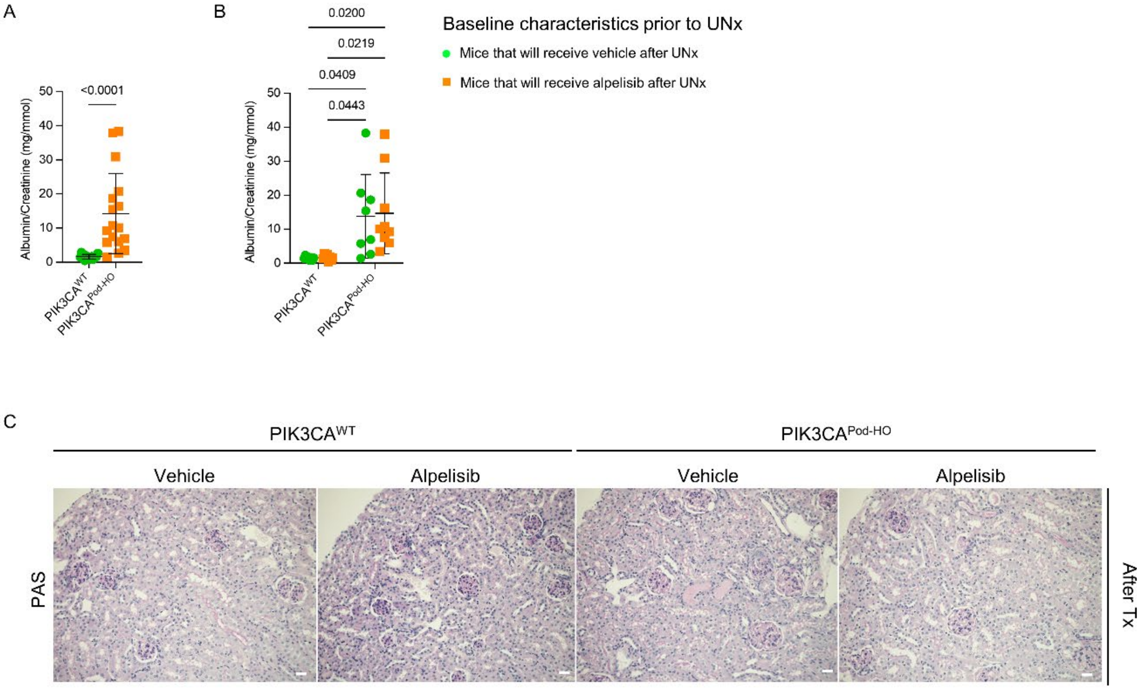

Supplemental Figure 7

Supplemental Figure 7: Alpelisib improves kidney lesions in uninephrectomized *PIK3CA$^{Pod\text{-}HO}$* mice. UNx: Uninephrectomy. (**A**) Urinary albumin/creatinine of *PIK3CA^{WT}* (n=16) and *PIK3CA$^{Pod\text{-}HO}$* (n=17) mice prior to UNx. (**B**) Urinary albumin/creatinine of *PIK3CA^{WT}* and *PIK3CA$^{Pod\text{-}HO}$* mice prior to UNx that will be treated with either vehicle or alpelisib. n=8 for *PIK3CA^{WT}*-Vehicle, *PIK3CA^{WT}*-Alpelisib, *PIK3CA^{HO}*-Vehicle, and n=9 for *PIK3CA^{HO}*-Alpelisib. (**C**) PAS staining of kidneys from *PIK3CA^{WT}* and *PIK3CA$^{Pod\text{-}HO}$* mice at sacrifice. Data are represented as means ± SD from 3 independent experiments for (**A**) and (**B**). P values are calculated using two-tailed Mann-Whitney U test (**A**), two-way ANOVA with Tukey's post hoc test (**B**). Scale bars: 32.2 μm (**C**). Some data are identical between (**A**), (**B**) and Figure **4L**.

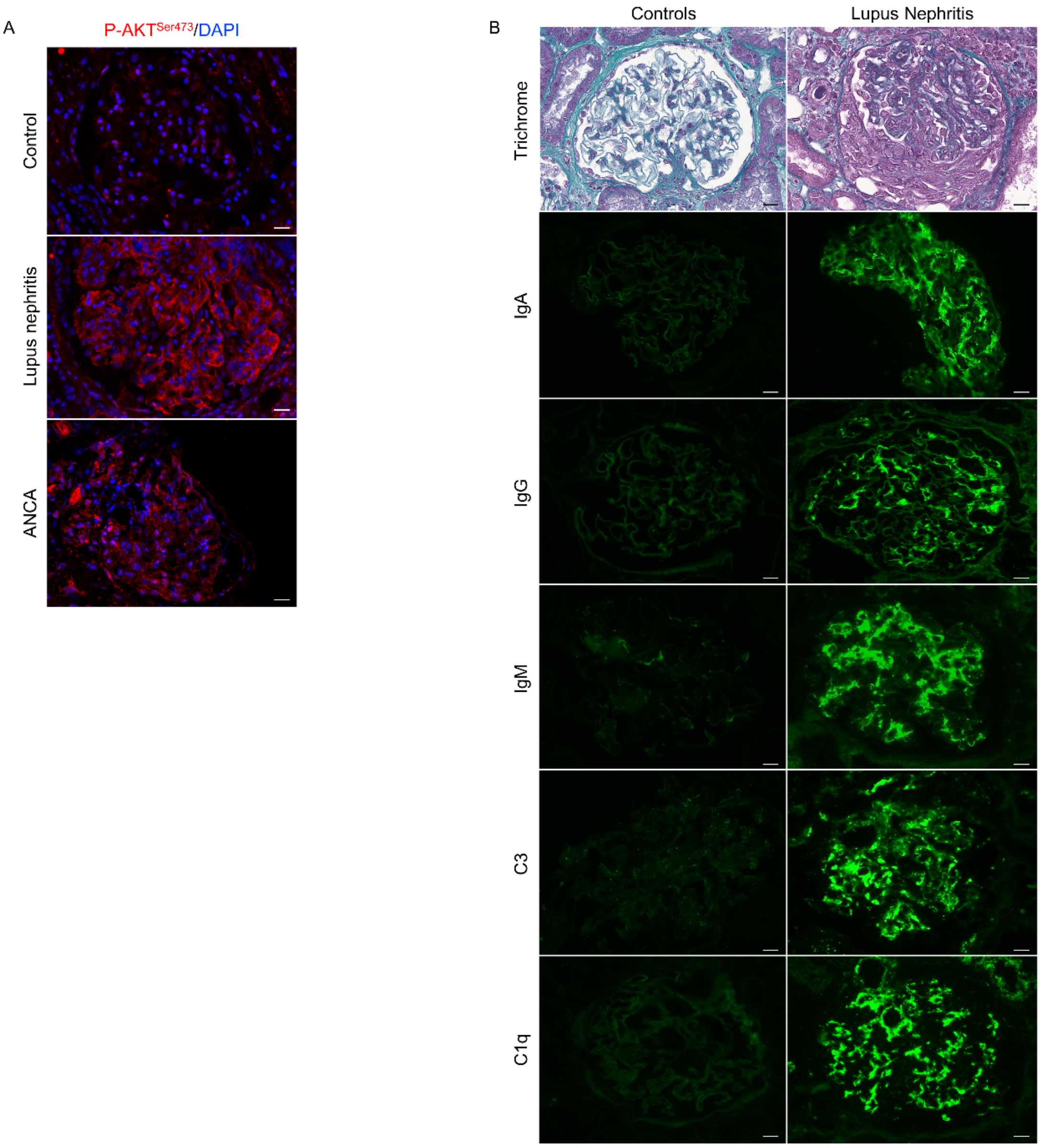

C

		Glom. ROI No.	Reference No.	Age (yrs.)	Sex	Morphology	Lupus classification (2003)	Chronicity (%)	NIH classification activity	NIH classification chronicity	GS	IFTA (%)	Crescent lesion (n)	Crescent lesion (%)	IF
Slide A	Control A2	1-2	21NA01839	19	M	Normal					0	0	0	0	Neg
	Control A1	3-6	21NA05988	36	F	Normal					0	0	0	0	Neg
	LN A2	15-23	19NA08977	21	M	Lupus Nephritis	4	6	12/24	1/12	0/15	5	7/15	47%	Full house
	LN A1	7-14	19NA00135	18	F	Lupus Nephritis	4	22	14/24	4/12	2/30	15	15/30	50%	Full house
Slide B	LN B2	18-26	21NA03559	33	F	Lupus Nephritis	4	0	15/24	0/12	0/21	0	12/21	57%	Full house
	Control B2	1-4	21NA03758	25	F	Normal					0	0	0	0	Neg
	Control B1	5-10	21NA02670	32	F	Normal					0	0	0	0	Neg
	LN B1	11-17	22NA00744	41	F	Lupus Nephritis	4	0	14/24	0/12	0/19	0	17/19	89%	Full house

GS: Glomerulosclerosis over the total number of glomeruli; IFTA: Interstitial fibrosis and tubular atrophy; IF: Immunofluorescence.

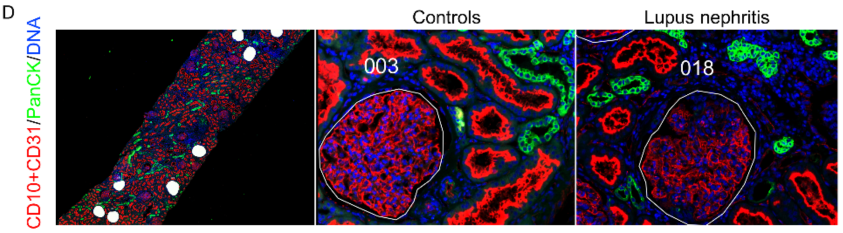

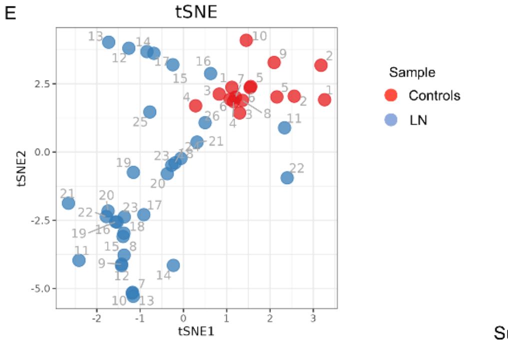

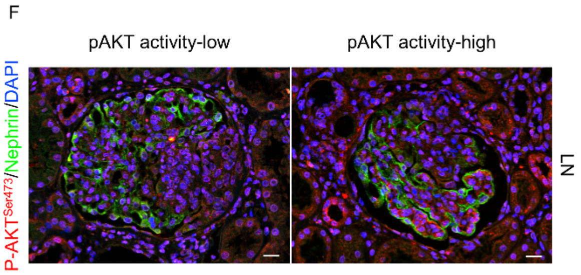

Supplemental Figure 8

Supplemental Figure 8: Human kidney biopsies used for glomerular spatial transcriptomics. (**A**) Representative immunofluorescence staining of P-AKT^{Ser473} (frozen section) in kidney biopsies from patients with lupus nephritis (LN), ANCA vasculitis or controls (n=4 patients per group). Note the images are the split channel images of Figure 4A. (**B**) Representative kidney staining (Trichrome staining and immunofluorescence of IgA, IgG, IgM, C3, and C1q) for controls and LN patients. (**C**) Patient biopsy information and NIH classification for LN to demonstrate the activity of the disease. A total of 49 ROI distributions among each sample are shown. (**D**) Schematic of the spatial transcriptomics workflow. Two slides were analyzed. On each slide (slide A and B), 2 LN kidney biopsies and 2 control kidney biopsies were assigned to avoid batch effects between the slides. CD10 + CD31, PanCK, and DNA were used to visualize the kidney structure to enable selection of the region of interest (ROI). Representative ROIs for controls and LN are shown. (**E**) T-distributed neighbor embedding (t-SNE) plot showing the distribution of LN (in blue) and controls (in red) glomeruli. (**F**) Representative Nephrin/P-AKT^{Ser473} immunofluorescence staining for LN kidneys. With mean intensity quantification, the LN ROIs were classified either as pAKT activity-low or pAKT activity-high. Scaler bars: 20 μm (**A, B, F**).

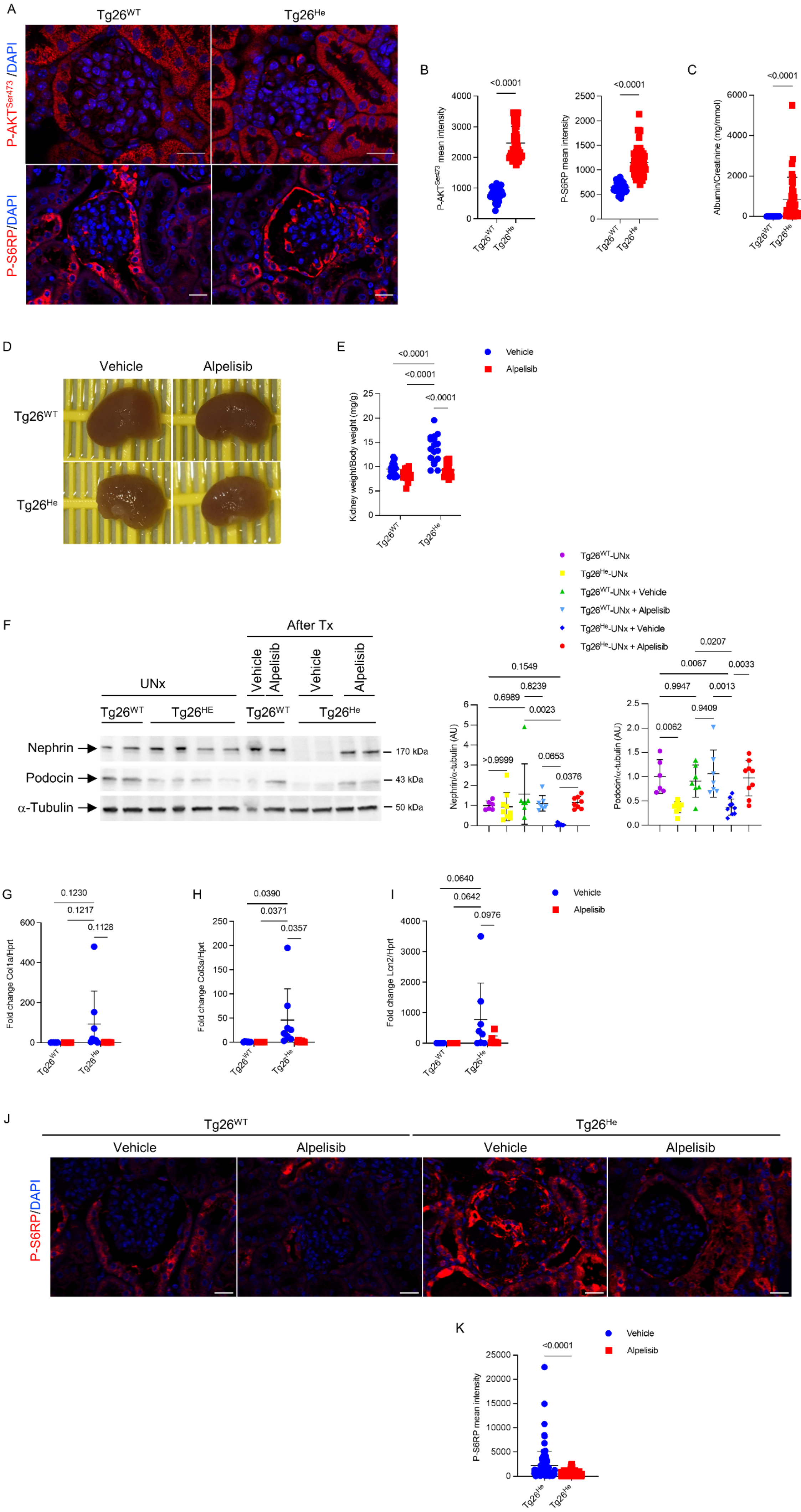

Supplemental Figure 9

Supplemental Figure 9: Alpelisib improves kidney lesions in a mouse model of collapsing glomerulopathy. (**A**) Representative P-AKT^{Ser473} and P-S6RP immunofluorescence staining in kidneys from 4-week-old *Tg26*^{WT} and *Tg26*^{He} mice. Scale bar 20 μm. (**B**) Quantification of mean P-AKT^{Ser473} intensity and P-S6RP mean intensity of glomeruli from 4-week-old *Tg26*^{WT} and *Tg26*^{He} mice. (n=6 mice per group). (**C**) Urinary albumin to creatinine ratio of 4-week-old *Tg26*^{WT} and *Tg26*^{He} mice before UNx (n= 31 for *Tg26*^{WT}, and n= 37 for *Tg26*^{He} mice). Note that this data is identical to the before-UNx data in Figure 5Q. (**D**) Representative kidney photos of the *Tg26*^{WT} and *Tg26*^{He} mice at the time of sacrifice (4 weeks following UNx and treated either with vehicle or alpelisib). (**E**) kidney-to-body weight ratio of *Tg26*^{WT} and *Tg26*^{He} mice at the time of sacrifice (n= 16 for *Tg26*^{WT}-Vehicle, n= 15 for *Tg26*^{WT}-Alpelisib, n=18 for *Tg26*^{He}-Vehicle, n=19 for *Tg26*^{He}-Alpelisib). (**F**) Western blot and quantification of Nephrin, Podocin, and α-tubulin in kidney cortex from *Tg26*^{WT} and *Tg26*^{He} mice either at UNx or at sacrifice (n=6 for *Tg26*^{WT} UNx kidney, n=9 for *Tg26*^{He} UNx kidney, n=7 for *Tg26*^{WT}-Vehicle and *Tg26*^{WT}-Alpelisib, n=8 for *Tg26*^{He}-Vehicle and *Tg26*^{He}-Alpelisib). (**G**) *Col1a*, (**H**) *Col3a*, and (**I**) *Lcn2* quantification of qRT-PCR analysis in kidney cortex from *Tg26*^{WT} and *Tg26*^{He} mice at sacrifice (n=8 for *Tg26*^{WT}-Vehicle, *Tg26*^{WT}-Alpelisib, *Tg26*^{He}-Vehicle, and n=9 for *Tg26*^{He}-Alpelisib). (**J**) Representative P-S6RP immunofluorescence and their glomerular quantification (**K**) from *Tg26*^{WT} and *Tg26*^{He} mice kidney at sacrifice (n= 6 mice per group). Data are represented as means ± SD are from 6 independent experiments (**E**), or are means ± SD and representative of 3 independent experiments (**B**, **C**, **F**-**K**). P values calculated using two-tailed Mann-Whitney U test (**B**, **C**, and **K**), two-way ANOVA with Tukey's post hoc test (**E**, **G**-**I**); one-way ANOVA with Tukey's post hoc test (**F**).

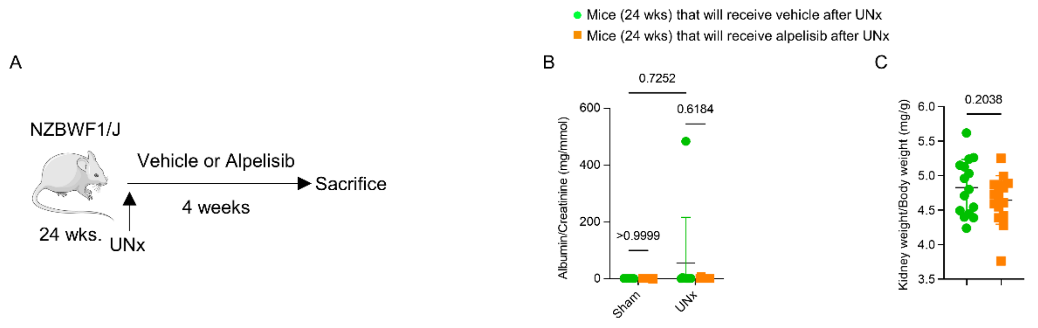

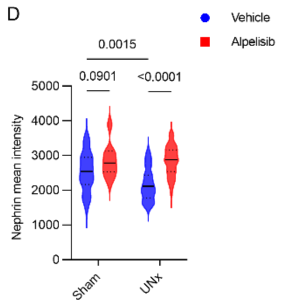

E

NZBWF1/J mice (Uninephrectomy. Tx for 4 weeks with vehicle/alpelisib)

	Vehicle	Alpelisib	P value
Hb (g/dL)*	7.33 ± 2.59	9.52 ± 2.22	0.014
Hct (%)	22.85 ± 8.59	28.88 ± 7.13	0.214
WBC (K/uL)*	1.50 ± 0.66	0.82 ± 0.16	0.034
Neut (K/uL)	0.87 ± 0.49	0.57 ± 0.31	0.224
Lymph (K/uL)*	0.50 ± 0.15	0.3 ± 0.08	0.015
Mono (K/uL)*	0.11 ± 0.07	0.03 ± 0.02	0.025
Eos (K/uL)	0.01 ± 0.01	0.02 ± 0.02	0.292
Plt (K/uL)*	588.67 ± 367.13	1104.00 ± 330.40	0.028

*:p<0.05. mean ± SD. Vehicle. n=6. Alpelisib. n=6.

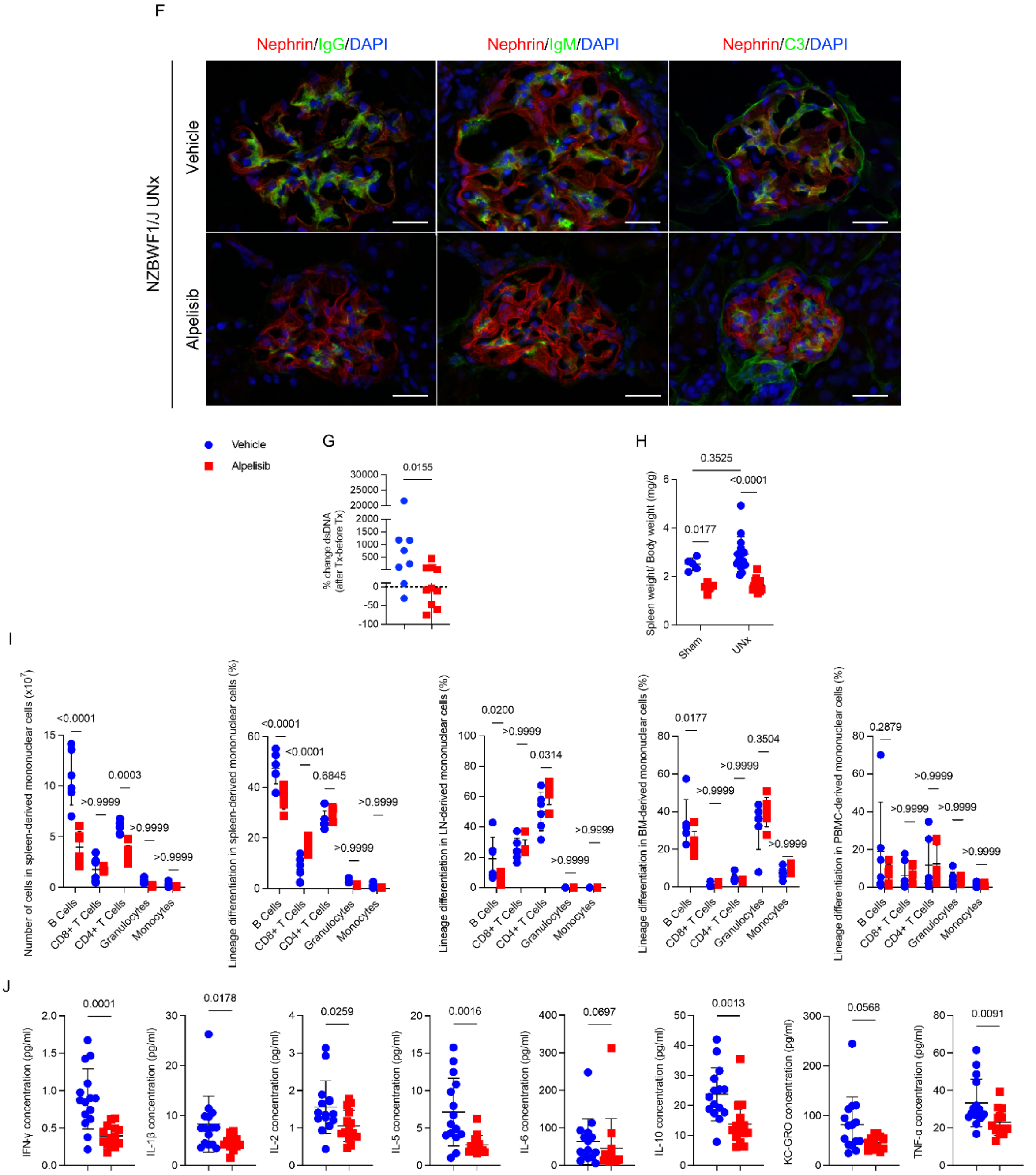

Supplemental Figure 10

Supplemental Figure 10: Alpelisib improves kidney lesions in *NZBWF1/J* Lupus Nephritis models. (**A**) Design of the experimental protocol. (**B**) Urinary albumin to creatinine ratio. (n= 5 for *NZBWF1/J*-Sham-Vehicle and *NZBWF1/J*-Sham-Alpelisib, n= 9 for *NZBWF1/J*-UNx-Vehicle and *NZBWF1/J*-UNx-Alpelisib mice). (**C**) Kidney-to-body weight ratio of *NZBWF1/J* mice UNx kidney (n=15 per group). **D**-**J** are of *NZBWF1/J* mice at the time of sacrifice (4 weeks after either sham operation or UNx followed by treatment with either the vehicle or alpelisib). (**D**) Nephrin mean intensity quantification of glomeruli at sacrifice (n= 6 mice per group). Related to Figure 7F. (**E**) Peripheral blood counts at sacrifice (n= 6 mice per group). (**F**) Representative IgG/Nephrin, IgM/Nephrin, and C3/Nephrin coimmunofluorescence staining at sacrifice (n=6 mice per group). Scale bar 20 μm. (**G**) dsDNA titer change ratio in the serum (after treatment at sacrifice to before UNx) of *NZBWF1/J* mice treated with either the vehicle or alpelisib (n=8 for *NZBWF1/J*-UNx-Vehicle, n=10 for *NZBWF1/J*-UNx-Alpelisib). (**H**) Spleen to body weight ratio at sacrifice (n=5 for *NZBWF1/J*-Sham-Vehicle and *NZBWF1/J*-Sham-Alpelisib, n=15 for *NZBWF1/J*-UNx-Vehicle and *NZBWF1/J*-UNx-Alpelisib mice). (**I**) Percentage of peripheral blood mononuclear cell (PBMCs), spleen, lymph nodes, and bone marrow cell population at sacrifice determined by flow cytometry analysis (n=6 per group). CD4, CD8, B220, Mac1, and Gr1 are used as lineage markers. For the spleen, the absolute cell numbers are also shown. (**J**) Serum cytokines at sacrifice (IFN-γ, IL-1β, IL-2, IL-5, IL-6, IL-10, KC-GRO, and TNF-α) measurements (n=16 mice for *NZBWF1/J*-UNx-Vehicle, n=15 mice for *NZBWF1/J*-UNx-Alpelisib). Values are the means ± SD and representative of 3 independent experiments (**B**, **C**, **D**, **G**, **H**, **I**, and **J**). P values calculated using two-way ANOVA with Tukey's post hoc test (**B**, **D**, and **H**); two-way ANOVA with Bonferroni's post hoc test (**I**), two-tailed *t* test (**C**, **E**), and two-tailed Mann-Whitney U test (**G** and **J**).

A

FVB/NJ mice (Tx for 4 weeks with vehicle/alpelisib)

	Vehicle	Alpelisib	P value
Hb (g/dL)	11.32 ± 4.77	13.77. ± 2.33	0.2935
Hct (%)	37.26 ± 17.62	47.03 ± 8.18	0.2536
WBC (K/uL)	3.98 ± 1.87	4.66 ± 2.05	0.5861
Neut (K/uL)	0.74 ± 0.39	1.25 ± 0.91	0.2764
Lymph (K/uL)	3.17 ± 1.55	3.31 ± 1.43	0.8793
Mono (K/uL)	0.02 ± 0.02	0.04 ± 0.03	0.5213
Eos (K/uL)	0.04 ± 0.03	0.07 ± 0.03	0.2594
Plt (K/uL)	1106 ± 545	1116 ± 174	0.9681

mean ± SD. Vehicle, n=5. Alpelisib, n=6.

B

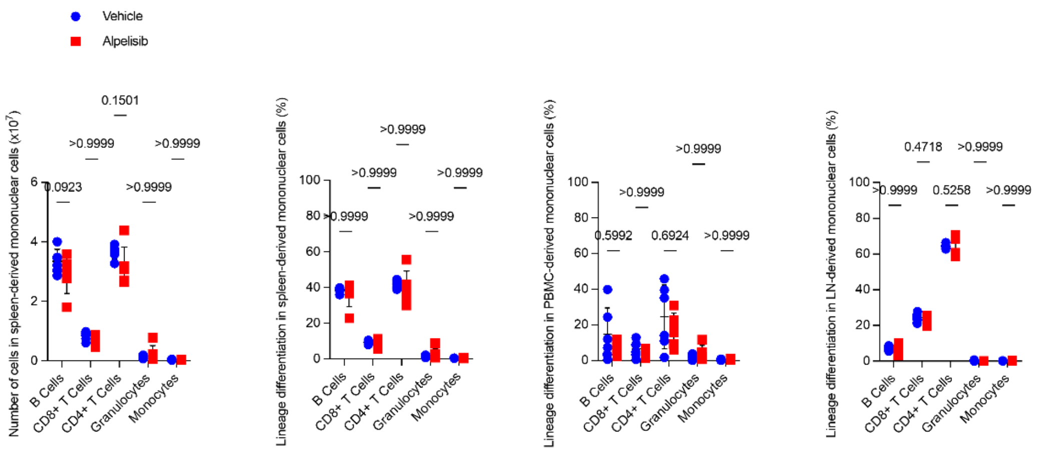

Supplemental Figure 11

Supplemental Figure 11: Alpelisib does not affect the lymphocyte population in wild-type mice. (**A**) Peripheral blood counts of FVB/N wild-type mice treated with either the vehicle or alpelisib for 4 weeks (n=5 mice for vehicle and n=6 mice for alpelisib). (**B**) Percentage of spleen, peripheral blood mononuclear cell (PBMCs), and lymph node cell population determined by flow cytometry analysis in FVB/N mice at the time of sacrifice and 4 weeks after treatment with either the vehicle or alpelisib (n=6 mice per group). For the spleen, the absolute cell numbers are also shown. CD4, CD8, B220, Mac1, Gr1 are used as lineage markers. Values are the means ± SD. P values calculated using two-tailed *t* test (**A**), and two-way ANOVA with Bonferroni's post hoc test (**B**).

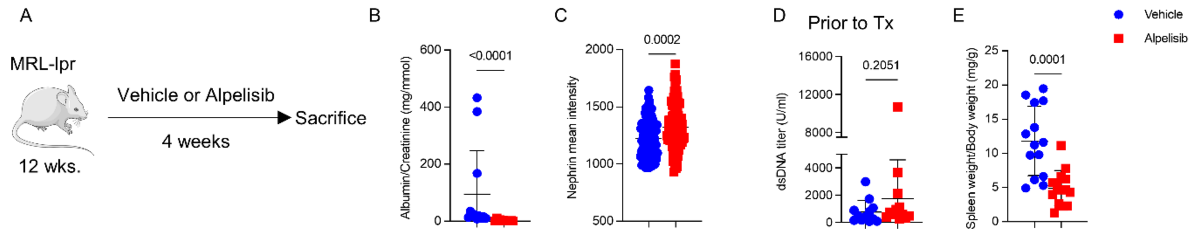

F

	Vehicle	Alpelisib	P value
Hb (g/dL)*	12.05 ± 1.85	14.39 ± 1.78	0.017
Hct (%)	38.44 ± 7.26	44.98 ± 6.28	0.064
WBC (K/uL)*	7.16 ±5.02	2.55 ± 1.58	0.016
Neut (K/uL)	1.74 ± 1.16	1.46 ± 1.20	0.556
Lymph (K/uL)*	4.7 ± 3.59	0.66 ± 0.39	0.004
Mono (K/uL)	0.53 ± 0.50	0.23 ± 0.30	0.162
Eos (K/uL)	0.17 ± 0.15	0.07 ± 0.07	0.072
Plt (K/uL)*	607.75 ± 164.56	911.44 ± 222.06	0.006

MRL/Lpr mice (Tx for 4 weeks with vehicle/alpelisib). *:p<0.05. mean ± SD. Vehicle. n=8. Alpelisib. n=9.

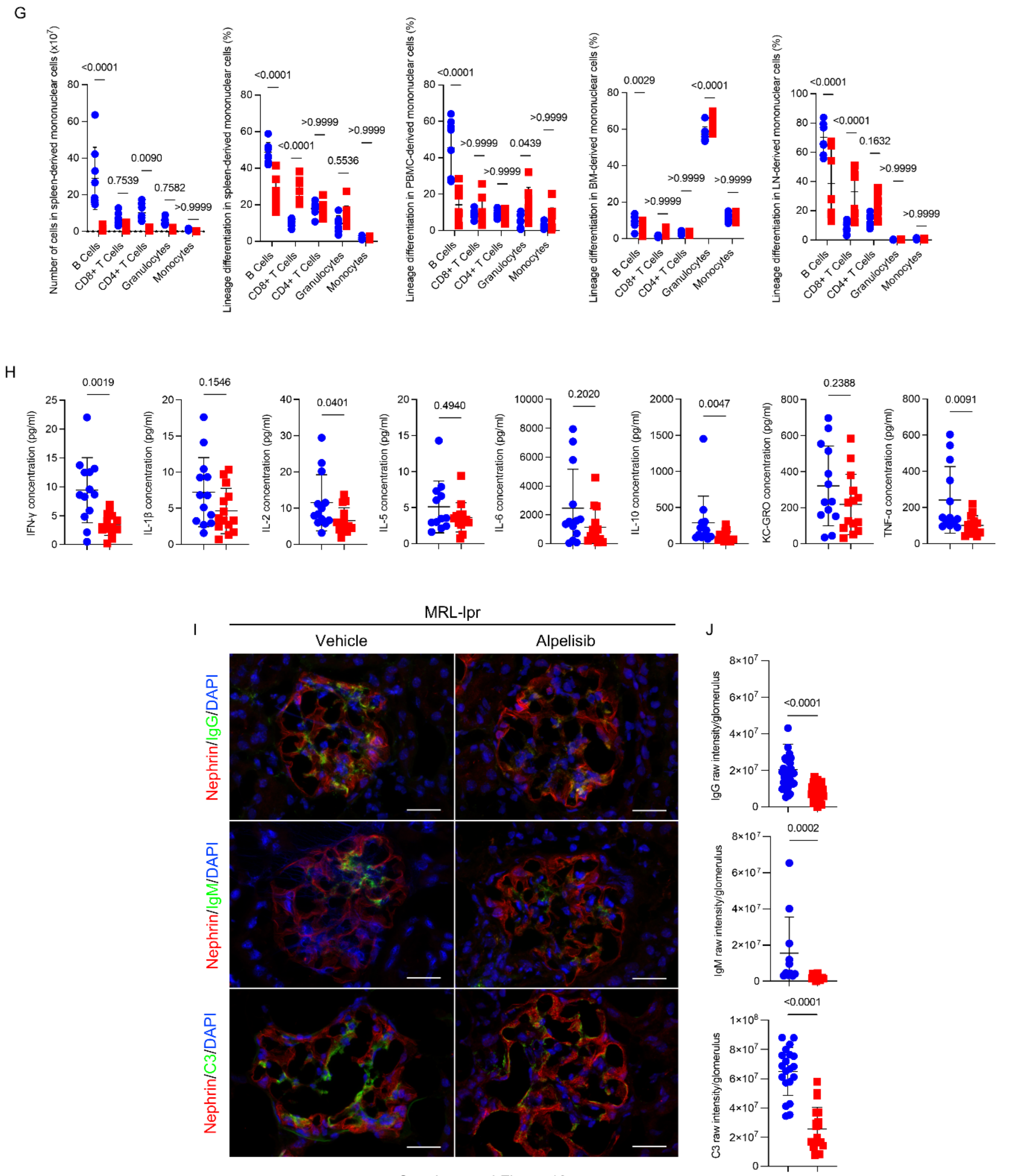

Supplemental Figure 12

Supplemental Figure 12: Alpelisib improves kidney lesions in *MRL-lpr* mice. (**A**) Design of the experimental protocol. **B**-**C**, and **E**-**J** are of *MRL-lpr* mice at the time of sacrifice (4 weeks after treatment with either the vehicle or alpelisib). (**B**) Urinary albumin to creatinine ratio at sacrifice (n=12 mice per group). (**C**) Nephrin mean intensity quantification from the kidney glomeruli at sacrifice (n=4 mice for vehicle, n=6 mice for alpelisib). (**D**) dsDNA titer in the serum of the *MRL-lpr* mice prior to treatment initiation at 12 weeks old (n=14 mice for vehicle, n=15 mice for alpelisib). (**E**) Spleen to body weight ratio at sacrifice (n=14 mice per group). (**F**) Peripheral blood cell counts at sacrifice (n=8 for vehicle, n=9 for alpelisib). (**G**) Percentage of spleen, peripheral blood mononuclear cell (PBMCs), bone marrow, and lymph node cell population at sacrifice determined by flow cytometry analysis (n=8 mice for vehicle, n=9 mice for alpelisib). For the spleen, absolute cell numbers are also shown. CD4, CD8, B220, Mac1, and Gr1 are used as lineage markers. (**H**) Serum cytokines (IFN-γ, IL-1β, IL-2, IL-5, IL-6, IL-10, KC-GRO, and TNF-α) at sacrifice (n=13 for vehicle, n=14 for alpelisib). Note that for IL-5, 1 vehicle mouse was below detection range that n=12 for vehicle in IL-5 measurement. (**I**) Representative IgG/Nephrin, IgM/Nephrin, C3/Nephrin coimmunofluorescence staining of kidneys. Scale bar 20 μm. (**J**) Quantification of the IgG, IgM, and C3 raw intensity per glomerulus (n=6 mice per group). Values are the means ± SD. P values calculated using two-way ANOVA with Bonferroni's post hoc test (**G**), two-tailed *t* test (**C**, **F**), and two-tailed Mann-Whitney U test (**B**, **D**, **E**, **H**, and **J**).

A

Patient (LN)	Treatments			
1	2022<br>• Steroids<br>• Mycophenolate mofetil<br>• Obinutuzumab			
2	2013<br>• Hydroxychloroquine<br>• Steroids<br>• Cyclophosphamide	2014<br>• Steroids<br>• Mycophenolate mofetil	2018<br>• Steroids<br>• Mycophenolate mofetil<br>• Rituximab	2022<br>• Steroids<br>• Mycophenolate mofetil<br>• Cyclophosphamide
3	2012<br>• Steroids<br>• Mycophenolate mofetil<br>• IVIG	2019<br>• Steroids<br>• Mycophenolate mofetil<br>• Cyclophosphamide	2022<br>• Steroids<br>• Mycophenolate mofetil<br>• Cyclophosphamide	
4	2014<br>• Hydroxychloroquine	2016<br>• Rituximab	2020<br>• Rituximab<br>• Mycophenolate mofetil	2022<br>• Steroids<br>• Cyclophosphamide<br>• Mycophenolate mofetil

B

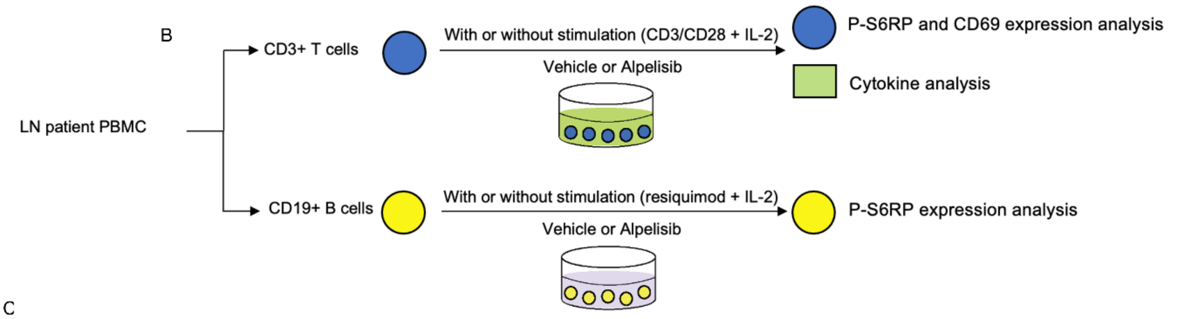

C

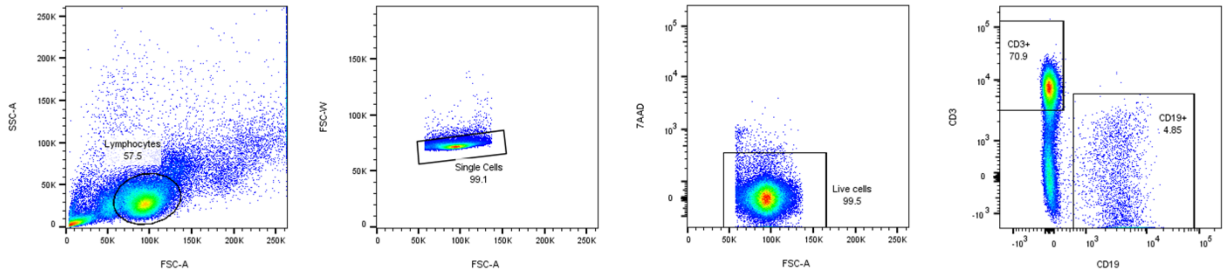

D

Patients (LN)	Age (yrs.)	Sex	PBMC sorting results			Serum cytokine results (pg/ml)									
			Lymphocytes (%PBMC)	CD3+ T cells (% PBMC)	CD19+ B cells (% PBMC)	IFN-γ	IL-1β	IL-2	IL-4	IL-6	IL-8	IL-10	IL-12	IL-13	TNF-α
1	35	F	11.5	9.6	0.047	1.73	0.58	0.23	0.05	1.01	124.68	0.94	0.22	0.79	4.77
2	21	F	57.2	40.4	2.8	1.04	0.43	N/A	0.04	0.75	24.23	0.85	0.12	3.49	3.83
3	26	F	14.6	12.6	1	20.52	2.29	0.36	0.06	3.85	853.13	0.66	0.35	4.17	3.98
4	22	F	27	22.9	1.7	4.08	1.40	0.18	0.03	0.46	1.90	1.70	NaN	NaN	1.17

From MSD data:

	IFN-γ	IL-1β	IL-2	IL-4	IL-6	IL-8	IL-10	IL-12	IL-13	TNF-α
Normal samples median (pg/ml)	3.77	0.16	0.52	ND	0.47	9.61	0.2	0.29	1.65	0.36
Range (pg/ml)	0.64-14.4	0.11-24.3	0.22-2.68	NA	0.16-27.2	1.48-1720	0.06-3.08	0.26-0.38	0.60-2.78	0.10-1.75

ND: Not detectable, NA: Not appropriate

E

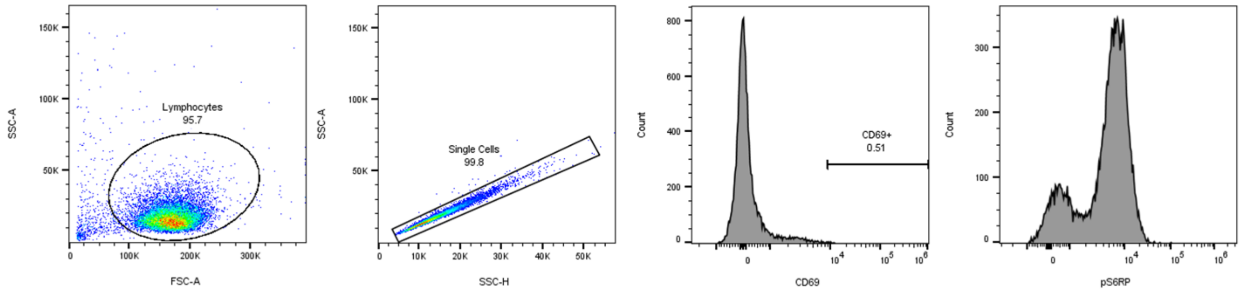

Supplemental Figure 13

Supplemental Figure 13: Alpelisib impairs the activation of lymphocytes from patients with Lupus Nephritis. (**A**) Immunosuppressant medical history of the 4 lupus patients (LN1-LN4) whose PBMCs underwent analysis. (**B**) Scheme of the study. (**C**) CD3+ T cell and CD19+ B cell sorting scheme from the PBMCs of lupus patients. (**D**) Lupus patients' PBMC sorting results for lymphocytes, including CD3+ T cells and CD19+ B cells (% PBMC), and serum cytokine results. The data of normal human serum tested by MSD® for this Proinflammatory Panel 1 (human) kit is shown as a reference. (**E**) The gating scheme of the phosphoflow analysis of CD3+ T cells 1 day after stimulation and treatment of the cells.

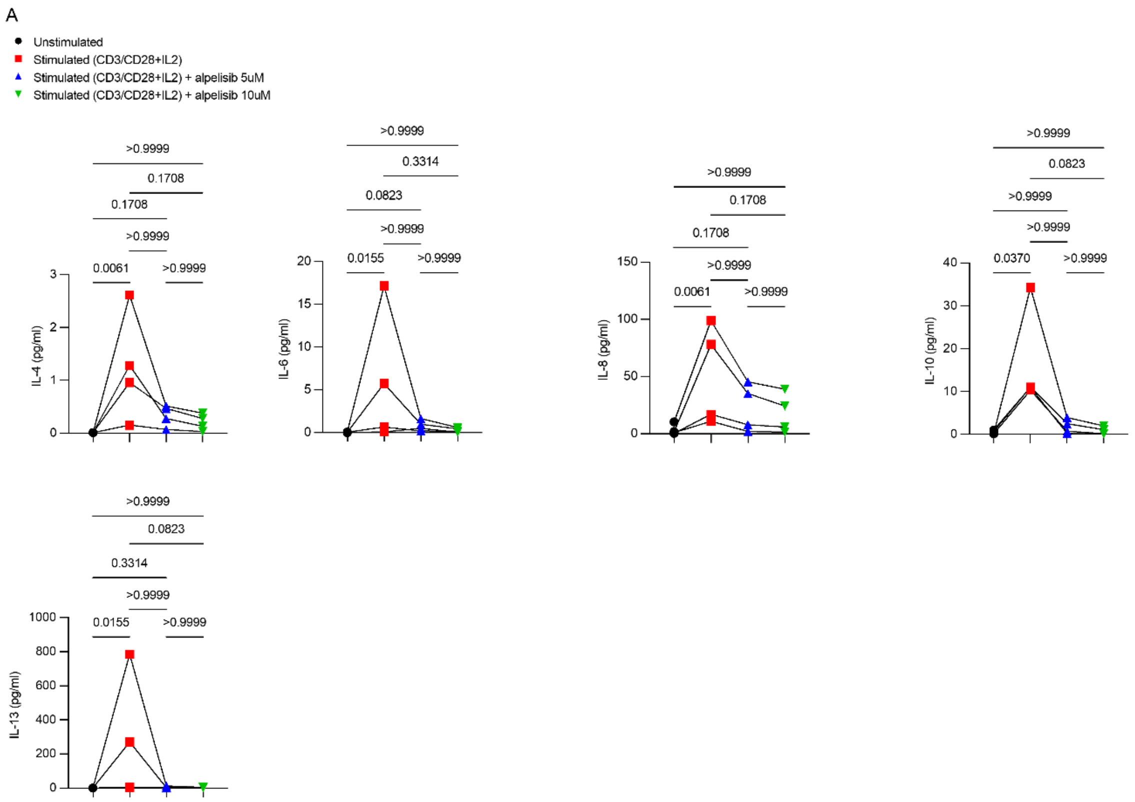

Supplemental Figure 14

Supplemental Figure 14: Alpelisib impaired, in vitro, B and T cell activation from lupus patients. (**A**) Cytokine measurements at day 7 in supernatant from non-stimulated or stimulated CD3+ T cells, treated with the vehicle or 5μM or 10μM alpelisib. Patients LN1-4 are included in the analysis. IL-4, IL-6, IL-8, IL-10 and IL-13 are shown here. Means ± SD. P values calculated using one-way ANOVA with Friedman test with Dunn's multiple comparisons test.

Supplemental Table 1. Representative highly expressed gene lists of 20 clusters

**p_val**	p-value
**avg_logFC**	Average fold change in expression between the compared groups
**pct.1**	Percentage of cells where the gene is detected in the first group (the cluster)
**pct.2**	Percentage of cells where the gene is detected in the second group (other cells)
**p_val_adj**	Adjusted p-value based on bonferroni correction using all genes in the dataset
**cluster**	Cluster identifier
**gene**	Gene identified as a marker

p_val	avg_logFC	pct.1	pct.2	p_val_adj	cluster	gene
0.00E+00	1.54	0.976	0.375	0.00E+00	Proximal Tubule-1	Spp2
0.00E+00	1.44	0.981	0.251	0.00E+00	Proximal Tubule-1	Slc5a2
0.00E+00	1.36	0.987	0.443	0.00E+00	Proximal Tubule-1	Gatm
0.00E+00	1.30	0.878	0.274	0.00E+00	Proximal Tubule-1	Cyp24a1
0.00E+00	1.21	1	0.985	0.00E+00	Proximal Tubule-1	Gpx3
0.00E+00	1.16	0.965	0.352	0.00E+00	Proximal Tubule-1	Slc6a19
0.00E+00	1.16	0.952	0.203	0.00E+00	Proximal Tubule-1	Slc5a12
0.00E+00	1.15	0.975	0.48	0.00E+00	Proximal Tubule-1	Nox4
0.00E+00	1.08	0.996	0.692	0.00E+00	Proximal Tubule-1	Slc4a4
0.00E+00	0.97	0.992	0.596	0.00E+00	Proximal Tubule-1	Maf
0.00E+00	0.97	0.747	0.162	0.00E+00	Proximal Tubule-1	Apob
0.00E+00	0.95	0.889	0.303	0.00E+00	Proximal Tubule-1	Cyp2d26
0.00E+00	0.92	0.934	0.367	0.00E+00	Proximal Tubule-1	Gldc
0.00E+00	0.91	0.748	0.142	0.00E+00	Proximal Tubule-1	Snhg11
0.00E+00	0.87	0.927	0.399	0.00E+00	Proximal Tubule-1	Slc7a7
0.00E+00	0.86	0.902	0.316	0.00E+00	Proximal Tubule-1	Slc7a8
0.00E+00	0.83	0.841	0.283	0.00E+00	Proximal Tubule-1	Clec2h
0.00E+00	0.82	0.988	0.769	0.00E+00	Proximal Tubule-1	Lgmn
0.00E+00	0.79	0.934	0.42	0.00E+00	Proximal Tubule-1	Slc2a2
0.00E+00	0.79	0.968	0.547	0.00E+00	Proximal Tubule-1	Fut9
0.00E+00	0.78	0.939	0.501	0.00E+00	Proximal Tubule-1	Enpp2
0.00E+00	0.78	0.962	0.504	0.00E+00	Proximal Tubule-1	Alpl
0.00E+00	0.76	0.999	0.882	0.00E+00	Proximal Tubule-1	Ndrg1
0.00E+00	0.76	0.985	0.627	0.00E+00	Proximal Tubule-1	Dab2
0.00E+00	0.75	0.967	0.581	0.00E+00	Proximal Tubule-1	Idh1
0.00E+00	0.73	0.943	0.537	0.00E+00	Proximal Tubule-1	G6pc
0.00E+00	0.70	0.991	0.794	0.00E+00	Proximal Tubule-1	Pdzk1ip1
0.00E+00	0.69	0.893	0.344	0.00E+00	Proximal Tubule-1	Acss1
0.00E+00	0.69	0.951	0.513	0.00E+00	Proximal Tubule-1	Lipa
0.00E+00	0.66	0.994	0.755	0.00E+00	Proximal Tubule-1	Cltrn
0.00E+00	0.66	1	0.809	0.00E+00	Proximal Tubule-1	Slc34a1
0.00E+00	0.66	0.723	0.081	0.00E+00	Proximal Tubule-1	Slc34a3
0.00E+00	0.65	0.983	0.681	0.00E+00	Proximal Tubule-1	Igfbp4
0.00E+00	0.64	0.95	0.575	0.00E+00	Proximal Tubule-1	Gsta2
0.00E+00	0.63	0.956	0.613	0.00E+00	Proximal Tubule-1	Asl
0.00E+00	0.63	0.995	0.802	0.00E+00	Proximal Tubule-1	Spink1
0.00E+00	0.62	0.899	0.481	0.00E+00	Proximal Tubule-1	Slc43a2
0.00E+00	0.62	0.611	0.064	0.00E+00	Proximal Tubule-1	630023F18Ri
0.00E+00	0.61	0.885	0.505	0.00E+00	Proximal Tubule-1	Creg1
0.00E+00	0.60	0.863	0.343	0.00E+00	Proximal Tubule-1	Folh1
0.00E+00	0.59	0.974	0.609	0.00E+00	Proximal Tubule-1	Slc13a1
0.00E+00	0.58	0.824	0.287	0.00E+00	Proximal Tubule-1	Prodh2
0.00E+00	0.56	0.755	0.227	0.00E+00	Proximal Tubule-1	Dpep1
0.00E+00	0.55	0.7	0.314	0.00E+00	Proximal Tubule-1	Rcan1
0.00E+00	0.54	0.75	0.276	0.00E+00	Proximal Tubule-1	Slc16a1
0.00E+00	0.53	0.942	0.55	0.00E+00	Proximal Tubule-1	Dnase1
0.00E+00	0.53	0.91	0.466	0.00E+00	Proximal Tubule-1	Cldn2
0.00E+00	0.53	0.947	0.534	0.00E+00	Proximal Tubule-1	Apoe
0.00E+00	0.52	0.991	0.743	0.00E+00	Proximal Tubule-1	Errfi1
0.00E+00	0.51	0.984	0.875	0.00E+00	Proximal Tubule-1	Mif
0.00E+00	0.51	0.946	0.578	0.00E+00	Proximal Tubule-1	Prodh
0.00E+00	0.51	0.874	0.485	0.00E+00	Proximal Tubule-1	Slc6a8

0.00E+00	0.50	0.727	0.176	0.00E+00	Proximal Tubule-1	Npl
0.00E+00	0.50	0.902	0.546	0.00E+00	Proximal Tubule-1	Nus1
0.00E+00	0.50	0.942	0.67	0.00E+00	Proximal Tubule-1	Slc3a2
0.00E+00	1.66	1	0.989	0.00E+00	Proximal Tubule-2	Kap
0.00E+00	1.29	0.954	0.216	0.00E+00	Proximal Tubule-2	Slc7a13
0.00E+00	1.26	0.994	0.568	0.00E+00	Proximal Tubule-2	Cyp2e1
0.00E+00	1.18	0.885	0.246	0.00E+00	Proximal Tubule-2	Cyp2a4
0.00E+00	1.18	0.995	0.459	0.00E+00	Proximal Tubule-2	Slc22a6
0.00E+00	1.16	0.855	0.129	0.00E+00	Proximal Tubule-2	Slco1a1
0.00E+00	1.16	1	0.775	0.00E+00	Proximal Tubule-2	Cyp4b1
0.00E+00	1.14	0.956	0.345	0.00E+00	Proximal Tubule-2	Cyp2d9
0.00E+00	1.10	0.991	0.582	0.00E+00	Proximal Tubule-2	Cndp2
0.00E+00	1.09	0.994	0.596	0.00E+00	Proximal Tubule-2	Acy3
0.00E+00	1.08	0.994	0.524	0.00E+00	Proximal Tubule-2	Mep1a
0.00E+00	1.07	0.965	0.398	0.00E+00	Proximal Tubule-2	Nat8
0.00E+00	1.02	1	0.748	0.00E+00	Proximal Tubule-2	Slc27a2
0.00E+00	1.01	0.99	0.573	0.00E+00	Proximal Tubule-2	Inmt
0.00E+00	1.01	0.967	0.365	0.00E+00	Proximal Tubule-2	Tmigd1
0.00E+00	1.00	0.985	0.396	0.00E+00	Proximal Tubule-2	Ces1f
0.00E+00	0.98	0.978	0.538	0.00E+00	Proximal Tubule-2	Nat8f1
0.00E+00	0.97	0.958	0.404	0.00E+00	Proximal Tubule-2	Ces2c
0.00E+00	0.97	0.998	0.652	0.00E+00	Proximal Tubule-2	Acaa1b
0.00E+00	0.96	0.986	0.557	0.00E+00	Proximal Tubule-2	Ugt2b38
0.00E+00	0.96	0.859	0.173	0.00E+00	Proximal Tubule-2	Acsm3
0.00E+00	0.95	0.989	0.635	0.00E+00	Proximal Tubule-2	Nudt19
0.00E+00	0.91	0.997	0.667	0.00E+00	Proximal Tubule-2	Cyp4a10
0.00E+00	0.89	1	0.762	0.00E+00	Proximal Tubule-2	Acsm2
0.00E+00	0.86	0.992	0.653	0.00E+00	Proximal Tubule-2	Ehhadh
0.00E+00	0.86	0.779	0.283	0.00E+00	Proximal Tubule-2	Cyp2a5
0.00E+00	0.86	1	0.906	0.00E+00	Proximal Tubule-2	Chpt1
0.00E+00	0.86	0.999	0.691	0.00E+00	Proximal Tubule-2	Akr1c21
0.00E+00	0.86	0.991	0.692	0.00E+00	Proximal Tubule-2	Ggt1
0.00E+00	0.85	0.994	0.544	0.00E+00	Proximal Tubule-2	Napsa
0.00E+00	0.85	0.999	0.632	0.00E+00	Proximal Tubule-2	Keg1
0.00E+00	0.85	0.993	0.607	0.00E+00	Proximal Tubule-2	Slc22a12
0.00E+00	0.85	0.826	0.169	0.00E+00	Proximal Tubule-2	Azgp1
0.00E+00	0.84	0.903	0.26	0.00E+00	Proximal Tubule-2	Cd36
0.00E+00	0.83	0.97	0.541	0.00E+00	Proximal Tubule-2	Ldhd
0.00E+00	0.82	0.987	0.58	0.00E+00	Proximal Tubule-2	Tmem252
0.00E+00	0.82	0.963	0.437	0.00E+00	Proximal Tubule-2	Slc17a3
0.00E+00	0.82	0.994	0.796	0.00E+00	Proximal Tubule-2	Acox1
0.00E+00	0.80	0.901	0.231	0.00E+00	Proximal Tubule-2	Slc5a8
0.00E+00	0.80	0.651	0.118	0.00E+00	Proximal Tubule-2	Ugt2b37
0.00E+00	0.79	0.953	0.447	0.00E+00	Proximal Tubule-2	Eci3
0.00E+00	0.79	0.987	0.598	0.00E+00	Proximal Tubule-2	Cyp4a14
0.00E+00	0.78	0.995	0.624	0.00E+00	Proximal Tubule-2	Cyp2j5
0.00E+00	0.78	0.942	0.363	0.00E+00	Proximal Tubule-2	Slc22a2
0.00E+00	0.77	0.931	0.273	0.00E+00	Proximal Tubule-2	Slc6a18
0.00E+00	0.77	0.973	0.679	0.00E+00	Proximal Tubule-2	Tmem205
0.00E+00	0.76	0.831	0.206	0.00E+00	Proximal Tubule-2	Mogat1
0.00E+00	0.76	0.986	0.549	0.00E+00	Proximal Tubule-2	Ugt3a2
0.00E+00	0.75	0.999	0.902	0.00E+00	Proximal Tubule-2	Cyb5a
0.00E+00	0.75	0.949	0.574	0.00E+00	Proximal Tubule-2	Retsat
0.00E+00	0.75	0.978	0.608	0.00E+00	Proximal Tubule-2	Guca2b
0.00E+00	0.75	0.994	0.791	0.00E+00	Proximal Tubule-2	Ttc36
0.00E+00	0.74	0.984	0.557	0.00E+00	Proximal Tubule-2	Slc17a1
0.00E+00	0.74	0.97	0.606	0.00E+00	Proximal Tubule-2	Pank1
0.00E+00	0.74	0.959	0.561	0.00E+00	Proximal Tubule-2	Crot
0.00E+00	0.74	0.982	0.565	0.00E+00	Proximal Tubule-2	Cyp4a31
0.00E+00	0.74	0.958	0.5	0.00E+00	Proximal Tubule-2	Me1
0.00E+00	0.73	0.967	0.491	0.00E+00	Proximal Tubule-2	Slc13a3
0.00E+00	0.72	0.948	0.402	0.00E+00	Proximal Tubule-2	Ugt3a1
0.00E+00	0.72	0.877	0.254	0.00E+00	Proximal Tubule-2	Slc22a30
0.00E+00	0.72	0.994	0.849	0.00E+00	Proximal Tubule-2	Tmem176a

0.00E+00	0.72	0.746	0.215	0.00E+00	Proximal Tubule-2	Hpd
0.00E+00	0.71	0.99	0.632	0.00E+00	Proximal Tubule-2	Cda
0.00E+00	0.70	0.895	0.331	0.00E+00	Proximal Tubule-2	630029K05Ri
0.00E+00	0.70	0.893	0.34	0.00E+00	Proximal Tubule-2	Atp11a
0.00E+00	0.69	0.983	0.59	0.00E+00	Proximal Tubule-2	Kcnj15
0.00E+00	0.69	0.918	0.481	0.00E+00	Proximal Tubule-2	Hao2
0.00E+00	0.68	0.973	0.543	0.00E+00	Proximal Tubule-2	Mfsd4b5
0.00E+00	0.68	0.992	0.676	0.00E+00	Proximal Tubule-2	Hmgcs2
0.00E+00	0.68	0.977	0.602	0.00E+00	Proximal Tubule-2	Ephx2
0.00E+00	0.66	0.914	0.407	0.00E+00	Proximal Tubule-2	Car4
0.00E+00	0.66	0.893	0.398	0.00E+00	Proximal Tubule-2	Mettl7b
0.00E+00	0.65	0.999	0.85	0.00E+00	Proximal Tubule-2	Rida
0.00E+00	0.64	0.915	0.444	0.00E+00	Proximal Tubule-2	Csad
0.00E+00	0.64	0.962	0.573	0.00E+00	Proximal Tubule-2	Pecr
0.00E+00	0.64	1	0.814	0.00E+00	Proximal Tubule-2	Slc34a1
0.00E+00	0.64	0.89	0.369	0.00E+00	Proximal Tubule-2	Adh1
0.00E+00	0.63	0.975	0.639	0.00E+00	Proximal Tubule-2	Lap3
0.00E+00	0.63	0.983	0.693	0.00E+00	Proximal Tubule-2	Gclm
0.00E+00	0.62	0.918	0.397	0.00E+00	Proximal Tubule-2	Adtrp
0.00E+00	0.61	0.927	0.493	0.00E+00	Proximal Tubule-2	Papss2
0.00E+00	0.60	0.916	0.422	0.00E+00	Proximal Tubule-2	Acot12
0.00E+00	0.60	0.956	0.562	0.00E+00	Proximal Tubule-2	Slc22a18
0.00E+00	0.60	0.876	0.291	0.00E+00	Proximal Tubule-2	Slc5a10
0.00E+00	0.60	0.953	0.504	0.00E+00	Proximal Tubule-2	Slc3a1
0.00E+00	0.59	1	0.963	0.00E+00	Proximal Tubule-2	Dbi
0.00E+00	0.59	0.967	0.548	0.00E+00	Proximal Tubule-2	Gm19950
0.00E+00	0.59	0.954	0.531	0.00E+00	Proximal Tubule-2	Cmbl
0.00E+00	0.59	0.958	0.6	0.00E+00	Proximal Tubule-2	Ghr
0.00E+00	0.59	0.778	0.35	0.00E+00	Proximal Tubule-2	Defb29
0.00E+00	0.59	1	0.955	0.00E+00	Proximal Tubule-2	Gpx1
0.00E+00	0.59	0.683	0.226	0.00E+00	Proximal Tubule-2	Gm45792
0.00E+00	0.58	0.979	0.664	0.00E+00	Proximal Tubule-2	Lpl
0.00E+00	0.58	0.906	0.559	0.00E+00	Proximal Tubule-2	Tmem37
0.00E+00	0.57	0.955	0.5	0.00E+00	Proximal Tubule-2	Slc47a1
0.00E+00	0.57	0.902	0.421	0.00E+00	Proximal Tubule-2	Vnn1
0.00E+00	0.57	0.942	0.536	0.00E+00	Proximal Tubule-2	Cryzl2
0.00E+00	0.57	0.974	0.7	0.00E+00	Proximal Tubule-2	Ccdc107
0.00E+00	0.57	0.974	0.587	0.00E+00	Proximal Tubule-2	Fmo2
0.00E+00	0.56	0.989	0.65	0.00E+00	Proximal Tubule-2	Lrp2
0.00E+00	0.56	0.869	0.394	0.00E+00	Proximal Tubule-2	Cgref1
0.00E+00	0.56	0.97	0.66	0.00E+00	Proximal Tubule-2	Acot1
0.00E+00	0.56	0.842	0.329	0.00E+00	Proximal Tubule-2	Serpinf2
0.00E+00	0.56	0.971	0.649	0.00E+00	Proximal Tubule-2	Aldh9a1
0.00E+00	0.56	0.99	0.759	0.00E+00	Proximal Tubule-2	Cat
0.00E+00	0.56	0.994	0.873	0.00E+00	Proximal Tubule-2	Tmem176b
0.00E+00	0.56	0.878	0.51	0.00E+00	Proximal Tubule-2	Hsd17b11
0.00E+00	0.56	0.962	0.549	0.00E+00	Proximal Tubule-2	Aass
0.00E+00	0.56	0.944	0.63	0.00E+00	Proximal Tubule-2	Dhrs4
0.00E+00	0.55	0.878	0.329	0.00E+00	Proximal Tubule-2	Rdh16f2
0.00E+00	0.55	0.99	0.653	0.00E+00	Proximal Tubule-2	Glyat
0.00E+00	0.54	0.789	0.229	0.00E+00	Proximal Tubule-2	Bhmt2
0.00E+00	0.54	0.852	0.346	0.00E+00	Proximal Tubule-2	Proc
0.00E+00	0.54	0.954	0.702	0.00E+00	Proximal Tubule-2	Msrb1
0.00E+00	0.54	0.964	0.651	0.00E+00	Proximal Tubule-2	Bphl
0.00E+00	0.54	0.858	0.415	0.00E+00	Proximal Tubule-2	Amacr
0.00E+00	0.53	0.968	0.603	0.00E+00	Proximal Tubule-2	Calml4
0.00E+00	0.53	0.744	0.186	0.00E+00	Proximal Tubule-2	Cyp2j13
0.00E+00	0.53	0.962	0.587	0.00E+00	Proximal Tubule-2	Ddah1
0.00E+00	0.52	0.97	0.691	0.00E+00	Proximal Tubule-2	Gstt2
0.00E+00	0.52	0.916	0.558	0.00E+00	Proximal Tubule-2	Dnajc12
0.00E+00	0.52	0.965	0.544	0.00E+00	Proximal Tubule-2	Tmem174
0.00E+00	0.52	0.796	0.408	0.00E+00	Proximal Tubule-2	G0s2
0.00E+00	0.52	0.968	0.711	0.00E+00	Proximal Tubule-2	Mettl26
0.00E+00	0.52	0.974	0.606	0.00E+00	Proximal Tubule-2	Folr1

0.00E+00	0.52	0.869	0.436	0.00E+00	Proximal Tubule-2	Coasy
0.00E+00	0.52	0.963	0.579	0.00E+00	Proximal Tubule-2	Gk
0.00E+00	0.51	0.895	0.498	0.00E+00	Proximal Tubule-2	Hyi
0.00E+00	0.51	0.968	0.567	0.00E+00	Proximal Tubule-2	AI314278
0.00E+00	0.51	0.944	0.586	0.00E+00	Proximal Tubule-2	Galnt11
0.00E+00	0.51	0.932	0.56	0.00E+00	Proximal Tubule-2	Fuca2
0.00E+00	0.51	0.92	0.484	0.00E+00	Proximal Tubule-2	Aqp1
0.00E+00	0.51	0.831	0.346	0.00E+00	Proximal Tubule-2	Hykk
0.00E+00	0.51	0.992	0.83	0.00E+00	Proximal Tubule-2	Scp2
0.00E+00	0.51	0.819	0.366	0.00E+00	Proximal Tubule-2	Fitm1
0.00E+00	0.50	0.884	0.439	0.00E+00	Proximal Tubule-2	Gas2
0.00E+00	0.50	0.998	0.89	0.00E+00	Proximal Tubule-2	Selenop
0.00E+00	0.50	0.875	0.382	0.00E+00	Proximal Tubule-2	Slco1a6
1.75E-52	0.53	0.764	0.603	5.41E-48	Proximal Tubule-2	Lars2
0.00E+00	1.09	1	0.99	0.00E+00	Proximal Tubule-3	Kap
0.00E+00	1.00	0.959	0.577	0.00E+00	Proximal Tubule-3	Inmt
0.00E+00	0.97	0.93	0.591	0.00E+00	Proximal Tubule-3	Cndp2
0.00E+00	0.94	0.988	0.654	0.00E+00	Proximal Tubule-3	Acaa1b
0.00E+00	0.92	0.998	0.85	0.00E+00	Proximal Tubule-3	Rida
0.00E+00	0.91	0.992	0.693	0.00E+00	Proximal Tubule-3	Akr1c21
0.00E+00	0.91	0.993	0.791	0.00E+00	Proximal Tubule-3	Ttc36
0.00E+00	0.90	0.98	0.678	0.00E+00	Proximal Tubule-3	Hmgcs2
0.00E+00	0.89	0.95	0.602	0.00E+00	Proximal Tubule-3	Acy3
0.00E+00	0.85	0.881	0.458	0.00E+00	Proximal Tubule-3	Eci3
0.00E+00	0.84	0.992	0.903	0.00E+00	Proximal Tubule-3	Cyb5a
0.00E+00	0.82	0.968	0.636	0.00E+00	Proximal Tubule-3	Cda
0.00E+00	0.80	0.966	0.573	0.00E+00	Proximal Tubule-3	Cyp2e1
0.00E+00	0.77	0.998	0.799	0.00E+00	Proximal Tubule-3	Ass1
0.00E+00	0.77	0.899	0.581	0.00E+00	Proximal Tubule-3	Pecr
0.00E+00	0.76	0.966	0.637	0.00E+00	Proximal Tubule-3	Keg1
0.00E+00	0.76	0.996	0.757	0.00E+00	Proximal Tubule-3	Miox
0.00E+00	0.72	0.884	0.551	0.00E+00	Proximal Tubule-3	Nat8f1
0.00E+00	0.71	0.896	0.67	0.00E+00	Proximal Tubule-3	Acot1
0.00E+00	0.71	0.908	0.646	0.00E+00	Proximal Tubule-3	Nudt19
0.00E+00	0.70	1	0.859	0.00E+00	Proximal Tubule-3	Aldob
0.00E+00	0.70	0.912	0.611	0.00E+00	Proximal Tubule-3	Ephx2
0.00E+00	0.69	0.981	0.702	0.00E+00	Proximal Tubule-3	Fbp1
0.00E+00	0.69	0.916	0.47	0.00E+00	Proximal Tubule-3	Slc22a6
0.00E+00	0.68	0.937	0.614	0.00E+00	Proximal Tubule-3	Guca2b
0.00E+00	0.68	0.999	0.955	0.00E+00	Proximal Tubule-3	Gpx1
0.00E+00	0.68	0.987	0.777	0.00E+00	Proximal Tubule-3	Cyp4b1
0.00E+00	0.67	0.954	0.697	0.00E+00	Proximal Tubule-3	Ggt1
0.00E+00	0.67	0.891	0.641	0.00E+00	Proximal Tubule-3	Grhpr
0.00E+00	0.67	0.843	0.549	0.00E+00	Proximal Tubule-3	Cryzl2
0.00E+00	0.67	0.833	0.383	0.00E+00	Proximal Tubule-3	Tmigd1
0.00E+00	0.66	0.859	0.641	0.00E+00	Proximal Tubule-3	Dhrs4
0.00E+00	0.66	0.892	0.711	0.00E+00	Proximal Tubule-3	Msrb1
0.00E+00	0.65	0.715	0.35	0.00E+00	Proximal Tubule-3	Rdh16f2
0.00E+00	0.65	0.89	0.651	0.00E+00	Proximal Tubule-3	Lap3
0.00E+00	0.65	0.85	0.545	0.00E+00	Proximal Tubule-3	Cmbl
0.00E+00	0.65	0.886	0.661	0.00E+00	Proximal Tubule-3	Bphl
0.00E+00	0.65	1	0.963	0.00E+00	Proximal Tubule-3	Dbi
0.00E+00	0.64	0.815	0.418	0.00E+00	Proximal Tubule-3	Nat8
0.00E+00	0.63	0.798	0.517	0.00E+00	Proximal Tubule-3	Tst
0.00E+00	0.63	0.95	0.706	0.00E+00	Proximal Tubule-3	Khk
0.00E+00	0.62	0.997	0.929	0.00E+00	Proximal Tubule-3	Akr1a1
0.00E+00	0.62	0.961	0.658	0.00E+00	Proximal Tubule-3	Glyat
0.00E+00	0.61	0.981	0.892	0.00E+00	Proximal Tubule-3	Etfb
0.00E+00	0.60	0.842	0.558	0.00E+00	Proximal Tubule-3	Ldhd
0.00E+00	0.60	0.753	0.372	0.00E+00	Proximal Tubule-3	Cyp2d9
0.00E+00	0.60	0.709	0.393	0.00E+00	Proximal Tubule-3	Adh1
0.00E+00	0.60	0.799	0.497	0.00E+00	Proximal Tubule-3	Hao2
0.00E+00	0.59	0.865	0.617	0.00E+00	Proximal Tubule-3	Msra
0.00E+00	0.59	0.975	0.893	0.00E+00	Proximal Tubule-3	Ech1

0.00E+00	0.59	0.831	0.417	0.00E+00	Proximal Tubule-3	Ces1f
0.00E+00	0.58	0.696	0.314	0.00E+00	Proximal Tubule-3	Slc5a10
0.00E+00	0.58	0.962	0.834	0.00E+00	Proximal Tubule-3	Scp2
0.00E+00	0.58	0.997	0.906	0.00E+00	Proximal Tubule-3	Chpt1
0.00E+00	0.57	0.888	0.619	0.00E+00	Proximal Tubule-3	Fah
0.00E+00	0.57	0.873	0.572	0.00E+00	Proximal Tubule-3	Ugt2b38
0.00E+00	0.56	0.938	0.615	0.00E+00	Proximal Tubule-3	Slc22a12
0.00E+00	0.56	0.834	0.619	0.00E+00	Proximal Tubule-3	Mcrip2
0.00E+00	0.55	0.876	0.692	0.00E+00	Proximal Tubule-3	Eci1
0.00E+00	0.54	0.931	0.742	0.00E+00	Proximal Tubule-3	Stard10
0.00E+00	0.54	0.871	0.683	0.00E+00	Proximal Tubule-3	Csrp2
0.00E+00	0.53	0.992	0.972	0.00E+00	Proximal Tubule-3	Gapdh
0.00E+00	0.53	0.985	0.804	0.00E+00	Proximal Tubule-3	Sord
0.00E+00	0.53	0.78	0.428	0.00E+00	Proximal Tubule-3	Ces2c
0.00E+00	0.53	0.871	0.58	0.00E+00	Proximal Tubule-3	AI314278
0.00E+00	0.53	0.992	0.75	0.00E+00	Proximal Tubule-3	Slc27a2
0.00E+00	0.53	0.731	0.431	0.00E+00	Proximal Tubule-3	Car4
0.00E+00	0.53	0.956	0.658	0.00E+00	Proximal Tubule-3	Ehhadh
0.00E+00	0.53	0.91	0.718	0.00E+00	Proximal Tubule-3	Akr7a5
0.00E+00	0.53	0.94	0.831	0.00E+00	Proximal Tubule-3	Aldh2
0.00E+00	0.53	0.807	0.52	0.00E+00	Proximal Tubule-3	Me1
0.00E+00	0.52	0.958	0.868	0.00E+00	Proximal Tubule-3	Txn1
0.00E+00	0.51	0.677	0.36	0.00E+00	Proximal Tubule-3	630029K05Ri
0.00E+00	0.51	0.931	0.803	0.00E+00	Proximal Tubule-3	Ddt
0.00E+00	0.51	0.978	0.815	0.00E+00	Proximal Tubule-3	Cyba
0.00E+00	0.50	0.94	0.632	0.00E+00	Proximal Tubule-3	Cyp2j5
3.04E-305	0.55	0.938	0.808	9.43E-301	Proximal Tubule-3	Hibadh
7.79E-305	0.52	0.885	0.614	2.41E-300	Proximal Tubule-3	Calml4
9.73E-302	0.50	0.849	0.562	3.01E-297	Proximal Tubule-3	Bdh2
1.47E-299	0.56	0.805	0.573	4.55E-295	Proximal Tubule-3	Tmem37
9.81E-295	0.55	0.925	0.767	3.04E-290	Proximal Tubule-3	Selenbp1
3.79E-282	0.51	0.892	0.705	1.17E-277	Proximal Tubule-3	Gclm
1.11E-276	0.50	0.84	0.631	3.45E-272	Proximal Tubule-3	Gcdh
1.00E-267	0.56	0.805	0.573	3.10E-263	Proximal Tubule-3	Dnajc12
7.07E-264	0.51	0.865	0.666	2.19E-259	Proximal Tubule-3	Cryz
1.64E-257	0.57	0.693	0.434	5.07E-253	Proximal Tubule-3	Apom
0.00E+00	0.97	0.782	0.299	0.00E+00	Proximal Tubule-4	Slc5a2
0.00E+00	0.95	0.834	0.412	0.00E+00	Proximal Tubule-4	Spp2
0.00E+00	0.85	1	0.985	0.00E+00	Proximal Tubule-4	Gpx3
0.00E+00	0.84	0.915	0.471	0.00E+00	Proximal Tubule-4	Gatm
0.00E+00	0.77	0.913	0.63	0.00E+00	Proximal Tubule-4	Asl
0.00E+00	0.74	0.896	0.595	0.00E+00	Proximal Tubule-4	Gsta2
0.00E+00	0.71	0.706	0.319	0.00E+00	Proximal Tubule-4	Clec2h
0.00E+00	0.67	0.991	0.801	0.00E+00	Proximal Tubule-4	Ass1
0.00E+00	0.66	0.752	0.398	0.00E+00	Proximal Tubule-4	Slc6a19
0.00E+00	0.63	0.755	0.437	0.00E+00	Proximal Tubule-4	Slc7a7
0.00E+00	0.60	0.681	0.318	0.00E+00	Proximal Tubule-4	Cyp24a1
0.00E+00	0.59	0.734	0.255	0.00E+00	Proximal Tubule-4	Slc5a12
0.00E+00	0.57	0.841	0.513	0.00E+00	Proximal Tubule-4	Nox4
0.00E+00	0.55	0.93	0.784	0.00E+00	Proximal Tubule-4	Lgmn
0.00E+00	0.53	0.774	0.405	0.00E+00	Proximal Tubule-4	Gldc
6.88E-308	0.54	0.968	0.881	2.13E-303	Proximal Tubule-4	Mif
2.50E-306	0.50	1	1	7.75E-302	Proximal Tubule-4	mt-Nd2
1.47E-295	0.56	0.794	0.539	4.55E-291	Proximal Tubule-4	Alpl
9.44E-274	0.50	0.931	0.698	2.93E-269	Proximal Tubule-4	Igfbp4
9.93E-271	0.56	0.966	0.706	3.08E-266	Proximal Tubule-4	Fbp1
9.78E-257	0.64	0.617	0.315	3.03E-252	Proximal Tubule-4	Cyp2d12
4.64E-252	0.52	0.991	0.759	1.44E-247	Proximal Tubule-4	Miox
9.16E-251	0.54	0.644	0.352	2.84E-246	Proximal Tubule-4	Cyp2d26
2.30E-215	0.50	0.825	0.577	7.11E-211	Proximal Tubule-4	Dnase1
1.70E-206	0.51	0.73	0.504	5.27E-202	Proximal Tubule-4	Cela1
0.00E+00	1.23	0.972	0.609	0.00E+00	Proximal Tubule-5	Cyp4a14
0.00E+00	0.84	0.998	0.794	0.00E+00	Proximal Tubule-5	Pck1
0.00E+00	0.83	0.95	0.54	0.00E+00	Proximal Tubule-5	Slc22a8

0.00E+00	0.81	0.982	0.676	0.00E+00	Proximal Tubule-5	Cyp4a10
0.00E+00	0.73	0.93	0.506	0.00E+00	Proximal Tubule-5	Slc13a3
0.00E+00	0.70	0.964	0.664	0.00E+00	Proximal Tubule-5	Ehhadh
0.00E+00	0.68	0.978	0.685	0.00E+00	Proximal Tubule-5	Hmgcs2
0.00E+00	0.68	0.979	0.626	0.00E+00	Proximal Tubule-5	Slc13a1
0.00E+00	0.65	0.967	0.767	0.00E+00	Proximal Tubule-5	Cat
0.00E+00	0.62	0.99	0.801	0.00E+00	Proximal Tubule-5	Acox1
0.00E+00	0.59	0.923	0.563	0.00E+00	Proximal Tubule-5	Aass
0.00E+00	0.59	0.682	0.238	0.00E+00	Proximal Tubule-5	Gm45792
0.00E+00	0.59	0.954	0.578	0.00E+00	Proximal Tubule-5	Cyp4a31
0.00E+00	0.58	0.963	0.582	0.00E+00	Proximal Tubule-5	Cyp2e1
0.00E+00	0.56	0.991	0.707	0.00E+00	Proximal Tubule-5	Slc4a4
0.00E+00	0.55	0.71	0.322	0.00E+00	Proximal Tubule-5	Slc5a10
0.00E+00	0.55	0.985	0.662	0.00E+00	Proximal Tubule-5	Acaa1b
0.00E+00	0.54	0.981	0.66	0.00E+00	Proximal Tubule-5	Lrp2
0.00E+00	0.52	0.988	0.755	0.00E+00	Proximal Tubule-5	Errfi1
0.00E+00	0.51	0.997	0.755	0.00E+00	Proximal Tubule-5	Slc27a2
0.00E+00	0.50	0.967	0.619	0.00E+00	Proximal Tubule-5	Slc22a12
9.61E-305	0.51	0.739	0.356	2.98E-300	Proximal Tubule-5	Rdh16f2
3.73E-279	0.50	0.994	0.762	1.16E-274	Proximal Tubule-5	Miox
1.48E-211	0.50	0.891	0.695	4.59E-207	Proximal Tubule-5	Abcd3
4.98E-204	0.52	0.856	0.589	1.54E-199	Proximal Tubule-5	Plin2
1.56E-95	0.54	0.698	0.543	4.82E-91	Proximal Tubule-5	Cbr1
0.00E+00	1.60	0.999	0.766	0.00E+00	Proximal Tubule-6	Jun
0.00E+00	1.57	0.903	0.313	0.00E+00	Proximal Tubule-6	Osgin1
0.00E+00	1.47	0.981	0.605	0.00E+00	Proximal Tubule-6	Dusp1
0.00E+00	1.44	0.608	0.156	0.00E+00	Proximal Tubule-6	Gm15441
0.00E+00	1.38	0.971	0.456	0.00E+00	Proximal Tubule-6	Angptl4
0.00E+00	1.34	0.935	0.254	0.00E+00	Proximal Tubule-6	Gm45792
0.00E+00	1.32	0.97	0.53	0.00E+00	Proximal Tubule-6	Slc25a25
0.00E+00	1.24	0.985	0.605	0.00E+00	Proximal Tubule-6	Brd2
0.00E+00	1.22	0.981	0.613	0.00E+00	Proximal Tubule-6	Tmem252
0.00E+00	1.15	0.992	0.745	0.00E+00	Proximal Tubule-6	Mat2a
0.00E+00	1.09	0.886	0.359	0.00E+00	Proximal Tubule-6	Pdk4
0.00E+00	1.07	0.987	0.718	0.00E+00	Proximal Tubule-6	Pim3
0.00E+00	1.06	0.991	0.797	0.00E+00	Proximal Tubule-6	Rsrp1
0.00E+00	1.06	0.971	0.564	0.00E+00	Proximal Tubule-6	Tob1
0.00E+00	1.04	0.998	0.768	0.00E+00	Proximal Tubule-6	Errfi1
0.00E+00	1.03	0.996	0.68	0.00E+00	Proximal Tubule-6	Acaa1b
0.00E+00	1.01	0.891	0.333	0.00E+00	Proximal Tubule-6	Ppp1r10
0.00E+00	1.01	0.991	0.694	0.00E+00	Proximal Tubule-6	Srsf2
0.00E+00	0.94	0.838	0.28	0.00E+00	Proximal Tubule-6	Slc7a13
0.00E+00	0.91	0.876	0.305	0.00E+00	Proximal Tubule-6	Slc22a30
0.00E+00	0.89	0.991	0.502	0.00E+00	Proximal Tubule-6	Slc22a6
0.00E+00	0.86	0.837	0.321	0.00E+00	Proximal Tubule-6	Gm43323
0.00E+00	0.86	0.785	0.214	0.00E+00	Proximal Tubule-6	Nat8f6
0.00E+00	0.85	0.999	0.975	0.00E+00	Proximal Tubule-6	Ubc
0.00E+00	0.84	0.789	0.252	0.00E+00	Proximal Tubule-6	031425E22Ri
0.00E+00	0.82	0.931	0.451	0.00E+00	Proximal Tubule-6	Thoc2l
0.00E+00	0.62	0.841	0.29	0.00E+00	Proximal Tubule-6	Trim7
2.20E-302	1.09	0.999	0.735	6.81E-298	Proximal Tubule-6	Neat1
5.17E-301	1.35	0.955	0.61	1.60E-296	Proximal Tubule-6	Cited2
5.35E-301	0.78	0.957	0.48	1.66E-296	Proximal Tubule-6	Slc17a3
5.12E-300	0.82	0.998	0.804	1.59E-295	Proximal Tubule-6	Zbtb20
2.98E-298	1.15	0.866	0.398	9.22E-294	Proximal Tubule-6	Hbegf
1.06E-297	1.29	0.855	0.423	3.28E-293	Proximal Tubule-6	Ccn1
6.71E-293	0.57	0.758	0.255	2.08E-288	Proximal Tubule-6	Pde4d
7.19E-293	0.89	0.997	0.848	2.23E-288	Proximal Tubule-6	Jund
7.03E-290	0.85	0.989	0.598	2.18E-285	Proximal Tubule-6	Cyp4a31
1.31E-287	0.83	0.999	0.769	4.06E-283	Proximal Tubule-6	Slc27a2
3.05E-287	0.78	0.909	0.463	9.45E-283	Proximal Tubule-6	Alas1
1.35E-285	0.63	0.857	0.337	4.19E-281	Proximal Tubule-6	Snx29
3.90E-284	0.84	0.961	0.539	1.21E-279	Proximal Tubule-6	Srsf7
6.24E-284	0.54	0.751	0.263	1.93E-279	Proximal Tubule-6	Slc25a42

9.39E-283	0.64	0.875	0.328	2.91E-278	Proximal Tubule-6	Slc6a18
1.26E-282	0.97	0.824	0.3	3.90E-278	Proximal Tubule-6	Cyp2a4
8.16E-280	0.59	0.843	0.288	2.53E-275	Proximal Tubule-6	Slc5a8
1.68E-279	0.58	0.801	0.292	5.20E-275	Proximal Tubule-6	632427E13Ri
1.56E-275	0.75	0.982	0.582	4.84E-271	Proximal Tubule-6	Gm19950
4.36E-270	0.88	0.951	0.515	1.35E-265	Proximal Tubule-6	Hao2
3.18E-269	0.83	0.951	0.611	9.85E-265	Proximal Tubule-6	Tubb4b
1.45E-265	0.70	0.999	0.903	4.49E-261	Proximal Tubule-6	Ddx5
5.34E-263	0.84	0.988	0.713	1.65E-258	Proximal Tubule-6	Gstt2
5.89E-263	0.91	1	0.995	1.83E-258	Proximal Tubule-6	Malat1
1.11E-262	1.52	0.917	0.547	3.45E-258	Proximal Tubule-6	Gadd45g
2.64E-260	0.53	0.766	0.259	8.16E-256	Proximal Tubule-6	Mogat1
4.25E-260	0.76	0.884	0.4	1.32E-255	Proximal Tubule-6	Rhob
6.09E-260	0.81	0.97	0.663	1.89E-255	Proximal Tubule-6	Sephs2
1.33E-258	0.59	0.872	0.352	4.13E-254	Proximal Tubule-6	Mlxipl
2.53E-256	0.60	0.92	0.411	7.83E-252	Proximal Tubule-6	Slc22a2
2.16E-253	0.73	0.992	0.639	6.68E-249	Proximal Tubule-6	Slc22a12
2.38E-252	0.61	0.67	0.232	7.37E-248	Proximal Tubule-6	Nr1d1
1.11E-251	0.59	0.87	0.402	3.45E-247	Proximal Tubule-6	Pex11a
2.84E-251	0.72	0.897	0.457	8.78E-247	Proximal Tubule-6	Aspa
2.13E-250	0.60	0.972	0.581	6.59E-246	Proximal Tubule-6	Napsa
2.51E-250	0.50	0.711	0.226	7.76E-246	Proximal Tubule-6	Azgp1
3.59E-248	0.68	0.892	0.438	1.11E-243	Proximal Tubule-6	Mettl7b
9.84E-248	0.86	0.994	0.874	3.05E-243	Proximal Tubule-6	Hsp90aa1
4.71E-246	0.66	0.959	0.537	1.46E-241	Proximal Tubule-6	Slc47a1
9.63E-243	0.61	0.889	0.373	2.98E-238	Proximal Tubule-6	Rdh16f2
1.32E-242	0.68	0.962	0.52	4.10E-238	Proximal Tubule-6	Abcc2
2.28E-235	0.80	0.966	0.665	7.07E-231	Proximal Tubule-6	Nudt19
8.34E-235	0.66	0.948	0.445	2.58E-230	Proximal Tubule-6	Ces1f
2.35E-234	0.74	0.992	0.694	7.28E-230	Proximal Tubule-6	Cyp4a10
3.57E-233	0.71	0.818	0.377	1.11E-228	Proximal Tubule-6	Arrdc3
5.43E-230	0.54	0.82	0.344	1.68E-225	Proximal Tubule-6	BC024386
1.85E-228	0.80	0.944	0.655	5.73E-224	Proximal Tubule-6	BC005537
2.43E-226	0.64	0.917	0.445	7.54E-222	Proximal Tubule-6	Nat8
3.04E-224	0.75	0.965	0.629	9.43E-220	Proximal Tubule-6	Acy3
3.68E-224	0.52	0.885	0.422	1.14E-219	Proximal Tubule-6	Slc6a20b
5.90E-224	0.67	0.965	0.604	1.83E-219	Proximal Tubule-6	Pecr
1.74E-222	0.69	0.991	0.63	5.38E-218	Proximal Tubule-6	Cyp4a14
4.24E-221	0.70	0.958	0.575	1.31E-216	Proximal Tubule-6	Nat8f1
4.81E-221	0.86	1	0.99	1.49E-216	Proximal Tubule-6	Kap
6.56E-221	0.59	0.918	0.45	2.03E-216	Proximal Tubule-6	Ces2c
7.38E-221	0.61	0.934	0.529	2.29E-216	Proximal Tubule-6	Hyi
1.21E-219	0.61	0.997	0.914	3.76E-215	Proximal Tubule-6	Chpt1
5.46E-219	0.63	0.978	0.592	1.69E-214	Proximal Tubule-6	Slc17a1
6.49E-219	0.71	0.986	0.717	2.01E-214	Proximal Tubule-6	Akr1c21
1.87E-218	0.72	0.955	0.617	5.79E-214	Proximal Tubule-6	Cndp2
4.09E-218	0.56	0.991	0.848	1.27E-213	Proximal Tubule-6	Eif5
1.22E-217	0.54	0.909	0.457	3.79E-213	Proximal Tubule-6	Xylb
2.67E-217	0.76	0.857	0.453	8.27E-213	Proximal Tubule-6	Hmgb2
3.39E-217	0.54	0.903	0.44	1.05E-212	Proximal Tubule-6	Adtrp
2.26E-216	0.67	0.944	0.615	7.00E-212	Proximal Tubule-6	Galnt11
1.53E-215	0.71	0.97	0.744	4.73E-211	Proximal Tubule-6	Dnaja1
3.75E-215	0.70	0.984	0.662	1.16E-210	Proximal Tubule-6	Keg1
2.78E-214	0.54	0.757	0.309	8.60E-210	Proximal Tubule-6	Kcnk5
4.63E-213	0.64	0.971	0.763	1.43E-208	Proximal Tubule-6	Sqstm1
1.80E-212	0.60	0.968	0.676	5.56E-208	Proximal Tubule-6	Aldh9a1
2.00E-212	0.67	0.916	0.542	6.19E-208	Proximal Tubule-6	Hnrnph1
1.94E-211	0.55	0.882	0.462	6.00E-207	Proximal Tubule-6	Tprkb
1.21E-210	0.56	0.826	0.408	3.75E-206	Proximal Tubule-6	Slc25a20
1.47E-210	0.65	0.943	0.578	4.56E-206	Proximal Tubule-6	Clk1
9.13E-210	0.53	0.843	0.379	2.83E-205	Proximal Tubule-6	630029K05Ri
1.14E-206	0.85	0.971	0.763	3.52E-202	Proximal Tubule-6	Tsc22d1
1.49E-206	0.58	0.928	0.584	4.63E-202	Proximal Tubule-6	Psmd7
1.71E-206	0.60	0.88	0.458	5.29E-202	Proximal Tubule-6	Taf1d

2.88E-206	0.69	0.98	0.751	8.93E-202	Proximal Tubule-6	Herpud1
4.09E-206	0.59	0.904	0.416	1.27E-201	Proximal Tubule-6	Tmigd1
6.76E-205	0.52	0.923	0.447	2.09E-200	Proximal Tubule-6	Ugt3a1
1.70E-204	0.61	0.965	0.563	5.26E-200	Proximal Tubule-6	Mep1a
1.79E-204	0.58	0.89	0.483	5.55E-200	Proximal Tubule-6	Csad
3.30E-203	0.58	0.962	0.655	1.02E-198	Proximal Tubule-6	Dhrs4
1.29E-201	0.64	0.997	0.959	4.00E-197	Proximal Tubule-6	Gpx1
2.24E-201	0.52	0.925	0.521	6.94E-197	Proximal Tubule-6	Hoga1
6.47E-199	0.59	0.967	0.586	2.00E-194	Proximal Tubule-6	Ugt3a2
8.39E-199	0.57	0.847	0.424	2.60E-194	Proximal Tubule-6	Nufip2
1.79E-198	0.55	0.891	0.504	5.55E-194	Proximal Tubule-6	Vegfa
2.30E-198	0.59	0.862	0.469	7.12E-194	Proximal Tubule-6	Snhg12
3.08E-198	0.69	0.963	0.607	9.53E-194	Proximal Tubule-6	Inmt
1.27E-197	0.71	1	0.806	3.94E-193	Proximal Tubule-6	Pck1
1.57E-197	0.56	0.734	0.287	4.86E-193	Proximal Tubule-6	Ier5
2.45E-197	0.55	0.892	0.46	7.60E-193	Proximal Tubule-6	Vnn1
3.36E-197	0.61	0.971	0.685	1.04E-192	Proximal Tubule-6	Acot1
1.18E-196	0.59	0.932	0.581	3.64E-192	Proximal Tubule-6	Atf4
7.82E-195	0.55	0.971	0.583	2.42E-190	Proximal Tubule-6	Aldh8a1
4.40E-194	0.59	0.942	0.575	1.36E-189	Proximal Tubule-6	Tra2b
6.04E-194	0.60	0.865	0.398	1.87E-189	Proximal Tubule-6	Cyp2d9
6.94E-192	0.57	0.905	0.516	2.15E-187	Proximal Tubule-6	Rsrc2
3.22E-190	0.56	0.959	0.636	9.99E-186	Proximal Tubule-6	Pank1
3.28E-190	0.66	0.633	0.242	1.02E-185	Proximal Tubule-6	Rn7sk
1.81E-188	0.62	0.968	0.715	5.59E-184	Proximal Tubule-6	Pnrc1
5.91E-188	0.53	0.984	0.806	1.83E-183	Proximal Tubule-6	Hspd1
1.95E-187	0.56	0.855	0.452	6.04E-183	Proximal Tubule-6	Amacr
2.79E-187	0.54	0.945	0.569	8.63E-183	Proximal Tubule-6	Cryzl2
4.78E-187	0.57	0.969	0.529	1.48E-182	Proximal Tubule-6	Slc13a3
9.27E-186	0.54	0.93	0.564	2.87E-181	Proximal Tubule-6	Cebpb
6.08E-185	0.52	0.934	0.489	1.88E-180	Proximal Tubule-6	Eci3
6.95E-184	0.53	0.874	0.474	2.15E-179	Proximal Tubule-6	Mafg
2.83E-183	0.54	0.919	0.553	8.75E-179	Proximal Tubule-6	Pbld1
3.13E-183	0.53	0.867	0.438	9.69E-179	Proximal Tubule-6	Gm47283
6.56E-182	0.54	0.835	0.446	2.03E-177	Proximal Tubule-6	Hexim1
1.57E-181	0.60	0.919	0.566	4.86E-177	Proximal Tubule-6	Vmp1
1.75E-181	0.72	0.952	0.709	5.43E-177	Proximal Tubule-6	Dynll1
2.72E-180	0.53	0.8	0.377	8.44E-176	Proximal Tubule-6	Polr3e
6.22E-180	0.55	0.946	0.577	1.93E-175	Proximal Tubule-6	Ldhd
1.72E-179	0.61	0.965	0.638	5.34E-175	Proximal Tubule-6	Guca2b
1.78E-179	0.54	0.955	0.694	5.51E-175	Proximal Tubule-6	Csrp2
3.35E-178	0.59	0.931	0.595	1.04E-173	Proximal Tubule-6	Crot
1.25E-177	0.51	1	0.997	3.89E-173	Proximal Tubule-6	Ftl1
7.10E-175	0.60	0.803	0.404	2.20E-170	Proximal Tubule-6	Nabp1
1.06E-174	0.54	0.989	0.91	3.29E-170	Proximal Tubule-6	Cyb5a
2.01E-174	0.83	0.837	0.438	6.22E-170	Proximal Tubule-6	G0s2
1.86E-173	0.59	0.855	0.479	5.75E-169	Proximal Tubule-6	Tob2
3.47E-172	0.53	0.961	0.579	1.07E-167	Proximal Tubule-6	Mfsd4b5
9.12E-172	0.90	0.703	0.302	2.83E-167	Proximal Tubule-6	Gdf15
9.11E-170	0.59	0.994	0.794	2.82E-165	Proximal Tubule-6	Cyp4b1
1.92E-169	0.61	0.952	0.631	5.94E-165	Proximal Tubule-6	Slc25a30
8.03E-169	0.78	0.872	0.471	2.49E-164	Proximal Tubule-6	Btg2
1.50E-168	0.87	0.891	0.539	4.65E-164	Proximal Tubule-6	Sgk1
4.85E-168	0.59	0.902	0.575	1.50E-163	Proximal Tubule-6	Zfand5
3.02E-166	0.54	0.996	0.812	9.36E-162	Proximal Tubule-6	Acox1
2.24E-165	0.51	0.959	0.679	6.95E-161	Proximal Tubule-6	Srsf5
4.13E-165	0.51	0.933	0.615	1.28E-160	Proximal Tubule-6	Srsf3
5.85E-165	0.53	0.726	0.345	1.81E-160	Proximal Tubule-6	Dnajb4
7.49E-165	0.53	0.97	0.662	2.32E-160	Proximal Tubule-6	Cda
1.18E-164	0.51	0.969	0.717	3.65E-160	Proximal Tubule-6	Gclm
1.33E-162	0.65	0.812	0.459	4.11E-158	Proximal Tubule-6	Dnajb9
2.17E-162	0.52	0.984	0.703	6.71E-158	Proximal Tubule-6	Neu1
6.47E-161	0.52	0.992	0.716	2.00E-156	Proximal Tubule-6	Ggt1
3.48E-158	0.54	0.992	0.807	1.08E-153	Proximal Tubule-6	Ttc36

6.91E-158	0.53	0.974	0.604	2.14E-153	Proximal Tubule-6	Cyp2e1
1.00E-157	0.62	0.81	0.445	3.10E-153	Proximal Tubule-6	Med21
2.11E-152	0.67	0.717	0.326	6.53E-148	Proximal Tubule-6	Cyp2a5
4.26E-150	0.50	0.998	0.862	1.32E-145	Proximal Tubule-6	Rida
1.34E-149	0.54	1	0.893	4.16E-145	Proximal Tubule-6	Timp3
2.00E-148	0.65	0.932	0.601	6.21E-144	Proximal Tubule-6	Plin2
2.21E-148	0.50	0.952	0.663	6.85E-144	Proximal Tubule-6	Rbm47
5.11E-147	0.80	0.933	0.58	1.58E-142	Proximal Tubule-6	G6pc
8.43E-138	0.63	0.746	0.388	2.61E-133	Proximal Tubule-6	Hmox1
7.86E-134	0.51	0.987	0.702	2.43E-129	Proximal Tubule-6	Hmgcs2
1.12E-133	0.54	0.919	0.636	3.47E-129	Proximal Tubule-6	Glul
1.10E-130	0.52	1	0.829	3.41E-126	Proximal Tubule-6	Slc34a1
5.13E-129	0.54	0.996	0.878	1.59E-124	Proximal Tubule-6	Id2
2.82E-119	0.51	0.985	0.646	8.74E-115	Proximal Tubule-6	Slc13a1
2.69E-99	0.52	0.748	0.416	8.33E-95	Proximal Tubule-6	Resf1
1.89E-91	0.58	0.728	0.435	5.86E-87	Proximal Tubule-6	Id1
1.24E-87	0.62	0.634	0.356	3.86E-83	Proximal Tubule-6	Rcan1
9.83E-64	0.53	0.884	0.695	3.04E-59	Proximal Tubule-6	Txnip
0.00E+00	2.91	1	0.586	0.00E+00	Proximal Tubule-7	Napsa
0.00E+00	2.28	0.943	0.422	0.00E+00	Proximal Tubule-7	Aadat
0.00E+00	2.07	0.953	0.388	0.00E+00	Proximal Tubule-7	Mpv17l
0.00E+00	1.85	0.83	0.05	0.00E+00	Proximal Tubule-7	Scd1
0.00E+00	1.84	0.884	0.007	0.00E+00	Proximal Tubule-7	Serpina1f
0.00E+00	1.79	0.874	0.043	0.00E+00	Proximal Tubule-7	Slc22a7
0.00E+00	1.62	0.984	0.568	0.00E+00	Proximal Tubule-7	Mep1a
0.00E+00	1.62	0.99	0.852	0.00E+00	Proximal Tubule-7	Acadm
0.00E+00	1.62	0.978	0.633	0.00E+00	Proximal Tubule-7	Ghr
0.00E+00	1.56	0.926	0.305	0.00E+00	Proximal Tubule-7	Cyp2a4
0.00E+00	1.55	0.955	0.334	0.00E+00	Proximal Tubule-7	Slc6a18
0.00E+00	1.52	0.905	0.026	0.00E+00	Proximal Tubule-7	Slc22a13
0.00E+00	1.51	0.645	0.013	0.00E+00	Proximal Tubule-7	Serpina1d
0.00E+00	1.44	0.875	0.047	0.00E+00	Proximal Tubule-7	Mep1b
0.00E+00	1.43	0.934	0.24	0.00E+00	Proximal Tubule-7	Cyp51
0.00E+00	1.40	0.817	0.014	0.00E+00	Proximal Tubule-7	Mettl7a2
0.00E+00	1.39	0.922	0.369	0.00E+00	Proximal Tubule-7	Slc23a1
0.00E+00	1.27	0.849	0.025	0.00E+00	Proximal Tubule-7	Slc22a19
0.00E+00	1.26	0.737	0.159	0.00E+00	Proximal Tubule-7	Kcnk1
0.00E+00	1.26	0.875	0.178	0.00E+00	Proximal Tubule-7	Ppic
0.00E+00	1.21	0.792	0.105	0.00E+00	Proximal Tubule-7	Apoc3
0.00E+00	1.19	0.841	0.152	0.00E+00	Proximal Tubule-7	Akr1c14
0.00E+00	1.04	0.602	0.027	0.00E+00	Proximal Tubule-7	Cp
0.00E+00	1.02	0.825	0.178	0.00E+00	Proximal Tubule-7	Gpm6a
0.00E+00	1.01	0.723	0.005	0.00E+00	Proximal Tubule-7	Methig1
0.00E+00	0.98	0.849	0.186	0.00E+00	Proximal Tubule-7	Bdh1
0.00E+00	0.95	0.832	0.232	0.00E+00	Proximal Tubule-7	Mme
0.00E+00	0.91	0.815	0.158	0.00E+00	Proximal Tubule-7	Agt
0.00E+00	0.82	0.782	0.15	0.00E+00	Proximal Tubule-7	Ace
0.00E+00	0.81	0.715	0.134	0.00E+00	Proximal Tubule-7	Slc10a2
0.00E+00	0.76	0.734	0.088	0.00E+00	Proximal Tubule-7	Slc39a8
0.00E+00	0.67	0.732	0.166	0.00E+00	Proximal Tubule-7	BC035947
0.00E+00	0.58	0.652	0.119	0.00E+00	Proximal Tubule-7	Slc38a3
6.64E-303	1.34	0.825	0.234	2.06E-298	Proximal Tubule-7	Cryab
2.21E-291	1.26	0.915	0.391	6.85E-287	Proximal Tubule-7	Atp11a
1.95E-290	0.86	0.782	0.218	6.04E-286	Proximal Tubule-7	Kyat3
8.09E-290	1.07	0.898	0.294	2.51E-285	Proximal Tubule-7	Slc5a8
3.33E-284	0.75	0.77	0.202	1.03E-279	Proximal Tubule-7	Id4
2.06E-283	1.16	0.965	0.545	6.39E-279	Proximal Tubule-7	Slc3a1
1.25E-280	1.34	0.99	0.76	3.88E-276	Proximal Tubule-7	Gstm1
5.88E-276	1.09	1	0.915	1.82E-271	Proximal Tubule-7	Chpt1
3.08E-275	1.33	0.967	0.598	9.53E-271	Proximal Tubule-7	Crot
8.40E-275	0.99	0.886	0.309	2.60E-270	Proximal Tubule-7	Cdo1
1.74E-272	1.21	0.95	0.453	5.38E-268	Proximal Tubule-7	Ugt3a1
7.84E-267	1.15	0.939	0.488	2.43E-262	Proximal Tubule-7	Csad
2.03E-253	1.17	0.995	0.772	6.28E-249	Proximal Tubule-7	Slc27a2

1.91E-247	0.91	0.735	0.227	5.91E-243	Proximal Tubule-7	Gramd1b
4.27E-247	0.74	0.765	0.24	1.32E-242	Proximal Tubule-7	Slc6a13
7.02E-246	1.09	0.981	0.721	2.18E-241	Proximal Tubule-7	Gclm
4.06E-245	1.15	0.978	0.615	1.26E-240	Proximal Tubule-7	Gk
2.14E-237	0.82	0.801	0.229	6.62E-233	Proximal Tubule-7	Apob
2.39E-231	0.91	0.884	0.399	7.40E-227	Proximal Tubule-7	Cth
2.82E-229	0.69	0.773	0.246	8.72E-225	Proximal Tubule-7	Acox2
3.44E-222	1.17	0.986	0.633	1.07E-217	Proximal Tubule-7	Acy3
8.12E-219	1.10	0.978	0.668	2.52E-214	Proximal Tubule-7	Nudt19
8.59E-218	0.79	0.822	0.333	2.66E-213	Proximal Tubule-7	Ugdh
8.02E-216	0.89	0.882	0.388	2.48E-211	Proximal Tubule-7	Defb29
1.36E-214	0.77	0.761	0.272	4.22E-210	Proximal Tubule-7	Slc16a9
1.78E-213	1.18	0.974	0.642	5.52E-209	Proximal Tubule-7	Guca2b
4.15E-212	0.91	0.894	0.345	1.29E-207	Proximal Tubule-7	Slc5a10
6.83E-204	0.99	0.822	0.357	2.12E-199	Proximal Tubule-7	Fgf1
1.92E-202	1.09	0.898	0.423	5.95E-198	Proximal Tubule-7	Tmigd1
1.27E-201	0.71	0.995	0.948	3.93E-197	Proximal Tubule-7	Tmbim6
1.02E-200	0.67	0.676	0.205	3.15E-196	Proximal Tubule-7	Slc16a4
2.44E-200	0.83	0.837	0.322	7.54E-196	Proximal Tubule-7	Cd36
6.97E-200	0.97	0.952	0.641	2.16E-195	Proximal Tubule-7	Pank1
1.96E-199	0.97	0.905	0.522	6.08E-195	Proximal Tubule-7	Nceh1
5.66E-194	0.67	0.798	0.309	1.75E-189	Proximal Tubule-7	Cyp2j11
7.54E-190	0.62	0.804	0.281	2.34E-185	Proximal Tubule-7	Bhmt2
2.08E-188	0.77	0.836	0.396	6.45E-184	Proximal Tubule-7	Osbpl8
3.64E-188	0.80	0.898	0.418	1.13E-183	Proximal Tubule-7	Slc22a2
2.87E-185	0.89	0.922	0.524	8.89E-181	Proximal Tubule-7	Aqp1
3.92E-184	0.90	0.988	0.667	1.22E-179	Proximal Tubule-7	Keg1
1.03E-182	0.90	0.998	0.814	3.19E-178	Proximal Tubule-7	Sult1d1
3.16E-181	0.66	0.787	0.318	9.79E-177	Proximal Tubule-7	Agps
5.20E-181	0.86	0.965	0.671	1.61E-176	Proximal Tubule-7	Lap3
1.13E-178	0.81	0.917	0.552	3.49E-174	Proximal Tubule-7	Entpd5
2.32E-176	0.79	0.894	0.535	7.19E-172	Proximal Tubule-7	Lypla1
1.43E-170	1.13	0.9	0.522	4.44E-166	Proximal Tubule-7	Hao2
4.37E-170	0.59	0.753	0.291	1.35E-165	Proximal Tubule-7	Slc15a2
3.12E-165	0.84	0.896	0.519	9.68E-161	Proximal Tubule-7	Gcnt1
7.50E-163	1.53	0.682	0.291	2.32E-158	Proximal Tubule-7	Slc7a13
1.07E-162	0.64	0.656	0.223	3.32E-158	Proximal Tubule-7	Acy1
1.28E-161	0.67	0.76	0.318	3.97E-157	Proximal Tubule-7	Cbs
2.34E-158	0.67	0.846	0.393	7.24E-154	Proximal Tubule-7	Proc
3.01E-158	0.78	0.974	0.72	9.33E-154	Proximal Tubule-7	Ggt1
3.00E-156	1.13	0.924	0.617	9.29E-152	Proximal Tubule-7	Gclc
2.53E-155	0.82	0.924	0.55	7.82E-151	Proximal Tubule-7	Pter
2.76E-154	0.72	0.875	0.497	8.55E-150	Proximal Tubule-7	Gss
3.99E-153	0.61	0.817	0.416	1.24E-148	Proximal Tubule-7	Tmem19
1.68E-150	0.62	0.78	0.335	5.19E-146	Proximal Tubule-7	Nqo1
7.08E-149	0.77	0.998	0.864	2.19E-144	Proximal Tubule-7	Rida
1.30E-148	0.73	0.948	0.591	4.03E-144	Proximal Tubule-7	Ugt3a2
8.20E-148	0.65	0.863	0.479	2.54E-143	Proximal Tubule-7	Tmem189
6.48E-147	0.58	0.829	0.395	2.01E-142	Proximal Tubule-7	Tpmt
6.96E-146	0.95	0.988	0.698	2.16E-141	Proximal Tubule-7	Cyp4a10
7.26E-144	0.81	0.933	0.638	2.25E-139	Proximal Tubule-7	Calml4
1.28E-142	0.72	0.991	0.862	3.97E-138	Proximal Tubule-7	Tmem176a
2.95E-140	1.10	0.623	0.243	9.14E-136	Proximal Tubule-7	Acsm3
1.12E-139	0.66	0.945	0.589	3.45E-135	Proximal Tubule-7	Aldh8a1
7.47E-139	1.27	0.998	0.99	2.31E-134	Proximal Tubule-7	Kap
7.89E-139	0.67	0.685	0.275	2.44E-134	Proximal Tubule-7	Reep6
2.84E-137	0.67	0.952	0.723	8.80E-133	Proximal Tubule-7	Lrpap1
1.87E-132	0.73	0.865	0.493	5.79E-128	Proximal Tubule-7	Slc1a1
3.34E-132	0.62	0.858	0.445	1.03E-127	Proximal Tubule-7	Mettl7b
1.76E-131	0.53	0.68	0.285	5.44E-127	Proximal Tubule-7	Glb1l2
9.19E-131	0.62	0.753	0.353	2.85E-126	Proximal Tubule-7	Gchfr
6.14E-130	0.73	0.957	0.604	1.90E-125	Proximal Tubule-7	Cyp4a31
2.97E-126	0.81	0.824	0.405	9.19E-122	Proximal Tubule-7	Cyp2d9
7.31E-122	0.63	0.946	0.676	2.27E-117	Proximal Tubule-7	Hmgcs1

8.90E-122	0.58	0.701	0.309	2.76E-117	Proximal Tubule-7	Lyplal1
1.06E-120	0.55	0.843	0.477	3.29E-116	Proximal Tubule-7	Coasy
1.13E-119	0.59	0.801	0.386	3.50E-115	Proximal Tubule-7	630029K05Ri
1.75E-118	0.60	0.912	0.544	5.43E-114	Proximal Tubule-7	Me1
7.25E-118	0.68	0.9	0.583	2.24E-113	Proximal Tubule-7	Ldhd
4.34E-117	0.52	0.862	0.447	1.34E-112	Proximal Tubule-7	Adtrp
1.28E-116	0.56	0.787	0.416	3.96E-112	Proximal Tubule-7	Acss2
2.03E-116	0.72	0.979	0.81	6.30E-112	Proximal Tubule-7	Ttc36
2.00E-115	0.62	0.946	0.68	6.21E-111	Proximal Tubule-7	Bphl
7.86E-114	0.63	0.862	0.536	2.43E-109	Proximal Tubule-7	Hyi
8.83E-113	0.60	0.889	0.596	2.74E-108	Proximal Tubule-7	Fuca2
6.51E-112	0.64	0.772	0.399	2.02E-107	Proximal Tubule-7	Mpst
1.80E-109	0.55	0.879	0.488	5.58E-105	Proximal Tubule-7	Slc17a3
1.60E-108	0.89	0.964	0.683	4.96E-104	Proximal Tubule-7	Lrp2
4.16E-108	0.55	0.86	0.486	1.29E-103	Proximal Tubule-7	Sod3
5.52E-107	0.59	0.976	0.884	1.71E-102	Proximal Tubule-7	Tmem176b
1.79E-106	0.56	0.903	0.611	5.54E-102	Proximal Tubule-7	Lactb2
2.17E-106	0.60	0.905	0.64	6.72E-102	Proximal Tubule-7	Glul
1.03E-105	0.58	0.913	0.575	3.18E-101	Proximal Tubule-7	Cryzl2
2.03E-105	0.59	0.898	0.621	6.29E-101	Proximal Tubule-7	Galnt11
1.19E-104	0.65	0.817	0.462	3.69E-100	Proximal Tubule-7	H1f2
2.84E-103	0.79	0.907	0.61	8.80E-99	Proximal Tubule-7	Retsat
4.17E-102	0.50	0.974	0.874	1.29E-97	Proximal Tubule-7	Oxct1
4.93E-102	0.56	0.995	0.911	1.53E-97	Proximal Tubule-7	Cyb5a
1.83E-101	0.58	0.879	0.496	5.65E-97	Proximal Tubule-7	Eci3
1.59E-100	0.56	0.971	0.921	4.93E-96	Proximal Tubule-7	Psap
2.23E-99	0.55	0.799	0.382	6.90E-95	Proximal Tubule-7	Rdh16f2
2.68E-99	0.52	0.789	0.45	8.30E-95	Proximal Tubule-7	Far1
1.07E-98	0.56	0.815	0.462	3.31E-94	Proximal Tubule-7	Tnfrsf21
1.73E-98	0.52	0.943	0.773	5.37E-94	Proximal Tubule-7	Slc25a39
4.98E-96	0.52	0.881	0.516	1.54E-91	Proximal Tubule-7	Cubn
5.74E-94	0.58	0.952	0.66	1.78E-89	Proximal Tubule-7	Cyp2j5
8.98E-94	0.75	0.72	0.37	2.78E-89	Proximal Tubule-7	Pdk4
1.14E-93	0.58	0.836	0.55	3.52E-89	Proximal Tubule-7	Por
1.31E-93	0.58	0.92	0.6	4.06E-89	Proximal Tubule-7	Slc22a18
9.08E-91	0.50	0.756	0.41	2.81E-86	Proximal Tubule-7	Ar
9.71E-90	0.52	0.742	0.39	3.01E-85	Proximal Tubule-7	Itgb8
1.01E-88	0.51	0.974	0.846	3.12E-84	Proximal Tubule-7	Scp2
3.50E-88	0.55	0.9	0.617	1.09E-83	Proximal Tubule-7	Gm2a
2.86E-87	0.54	0.903	0.604	8.86E-83	Proximal Tubule-7	Pipox
3.65E-85	0.52	0.967	0.728	1.13E-80	Proximal Tubule-7	Khk
4.29E-85	0.51	0.799	0.44	1.33E-80	Proximal Tubule-7	Cgref1
4.11E-84	0.58	0.971	0.9	1.27E-79	Proximal Tubule-7	Ndufb8
2.69E-83	0.51	0.957	0.644	8.32E-79	Proximal Tubule-7	Slc22a12
7.93E-81	0.51	0.962	0.876	2.46E-76	Proximal Tubule-7	Txn1
1.96E-78	0.66	0.964	0.923	6.07E-74	Proximal Tubule-7	Gpx4
5.70E-77	0.51	0.915	0.681	1.76E-72	Proximal Tubule-7	Mettl7a1
1.37E-73	0.79	0.884	0.751	4.25E-69	Proximal Tubule-7	Mat2a
6.78E-70	0.59	0.898	0.663	2.10E-65	Proximal Tubule-7	Slc6a6
1.21E-69	0.50	0.936	0.765	3.74E-65	Proximal Tubule-7	Sod1
1.88E-26	0.91	0.708	0.553	5.82E-22	Proximal Tubule-7	Cbr1
4.29E-21	0.66	0.612	0.434	1.33E-16	Proximal Tubule-7	Slco1a6
0.00E+00	3.60	0.954	0.104	0.00E+00	Loops of Henle	Slc12a1
0.00E+00	3.41	0.995	0.529	0.00E+00	Loops of Henle	Umod
0.00E+00	2.94	0.937	0.248	0.00E+00	Loops of Henle	Egf
0.00E+00	2.47	0.98	0.258	0.00E+00	Loops of Henle	Wfdc15b
0.00E+00	2.04	0.989	0.218	0.00E+00	Loops of Henle	Ppp1r1a
0.00E+00	1.80	0.858	0.198	0.00E+00	Loops of Henle	Slc5a3
0.00E+00	1.70	0.855	0.101	0.00E+00	Loops of Henle	Sostdc1
0.00E+00	1.69	0.988	0.615	0.00E+00	Loops of Henle	Nudt4
0.00E+00	1.67	0.934	0.216	0.00E+00	Loops of Henle	Ckb
0.00E+00	1.66	0.944	0.288	0.00E+00	Loops of Henle	Kng2
0.00E+00	1.65	0.998	0.715	0.00E+00	Loops of Henle	Mt1
0.00E+00	1.54	0.94	0.379	0.00E+00	Loops of Henle	Mrps6

0.00E+00	1.47	0.868	0.241	0.00E+00	Loops of Henle	Mt2
0.00E+00	1.44	0.739	0.27	0.00E+00	Loops of Henle	Hes1
0.00E+00	1.42	0.885	0.319	0.00E+00	Loops of Henle	Wfdc2
0.00E+00	1.41	0.882	0.342	0.00E+00	Loops of Henle	Cyfip2
0.00E+00	1.38	0.947	0.252	0.00E+00	Loops of Henle	Mal
0.00E+00	1.36	0.983	0.62	0.00E+00	Loops of Henle	Ly6a
0.00E+00	1.28	0.939	0.239	0.00E+00	Loops of Henle	Cd24a
0.00E+00	1.28	0.991	0.891	0.00E+00	Loops of Henle	Atp1b1
0.00E+00	1.25	0.802	0.115	0.00E+00	Loops of Henle	Mfsd4a
0.00E+00	1.24	0.809	0.103	0.00E+00	Loops of Henle	Tmem72
0.00E+00	1.24	0.851	0.147	0.00E+00	Loops of Henle	Epcam
0.00E+00	1.23	0.719	0.027	0.00E+00	Loops of Henle	Ppp1r1b
0.00E+00	1.21	0.882	0.147	0.00E+00	Loops of Henle	Kcnj1
0.00E+00	1.18	0.886	0.38	0.00E+00	Loops of Henle	Paqr5
0.00E+00	1.15	0.707	0.054	0.00E+00	Loops of Henle	Gpx6
0.00E+00	1.13	0.836	0.184	0.00E+00	Loops of Henle	Efhd1
0.00E+00	1.11	0.77	0.1	0.00E+00	Loops of Henle	Tfap2b
0.00E+00	1.11	0.732	0.159	0.00E+00	Loops of Henle	Clcnkb
0.00E+00	1.09	0.968	0.711	0.00E+00	Loops of Henle	Spp1
0.00E+00	1.09	0.718	0.058	0.00E+00	Loops of Henle	Clcnka
0.00E+00	1.06	0.994	0.96	0.00E+00	Loops of Henle	Atp1a1
0.00E+00	1.05	0.805	0.445	0.00E+00	Loops of Henle	Etnk1
0.00E+00	1.04	0.832	0.529	0.00E+00	Loops of Henle	Dst
0.00E+00	1.03	0.8	0.135	0.00E+00	Loops of Henle	Krt7
0.00E+00	0.97	0.667	0.06	0.00E+00	Loops of Henle	Gm47708
0.00E+00	0.97	0.732	0.145	0.00E+00	Loops of Henle	Rap1gap
0.00E+00	0.96	0.762	0.128	0.00E+00	Loops of Henle	Hk1
0.00E+00	0.96	0.752	0.216	0.00E+00	Loops of Henle	Pde1a
0.00E+00	0.96	0.658	0.291	0.00E+00	Loops of Henle	Atf3
0.00E+00	0.95	0.66	0.394	0.00E+00	Loops of Henle	Bhlhe40
0.00E+00	0.93	0.814	0.243	0.00E+00	Loops of Henle	Aif1l
0.00E+00	0.92	0.901	0.287	0.00E+00	Loops of Henle	Pkm
0.00E+00	0.92	0.804	0.433	0.00E+00	Loops of Henle	Ppargc1a
0.00E+00	0.90	0.829	0.484	0.00E+00	Loops of Henle	Adgrg1
0.00E+00	0.90	0.762	0.13	0.00E+00	Loops of Henle	Slc16a7
0.00E+00	0.89	0.862	0.634	0.00E+00	Loops of Henle	Pkp4
0.00E+00	0.89	0.626	0.017	0.00E+00	Loops of Henle	Cldn19
0.00E+00	0.88	0.78	0.449	0.00E+00	Loops of Henle	Btg2
0.00E+00	0.88	0.804	0.562	0.00E+00	Loops of Henle	Fos
0.00E+00	0.87	0.823	0.677	0.00E+00	Loops of Henle	Zfp36l1
0.00E+00	0.86	0.855	0.616	0.00E+00	Loops of Henle	Cgnl1
0.00E+00	0.84	0.699	0.305	0.00E+00	Loops of Henle	Irf2bpl
0.00E+00	0.83	0.693	0.108	0.00E+00	Loops of Henle	Kcnk1
0.00E+00	0.81	0.72	0.328	0.00E+00	Loops of Henle	Sorl1
0.00E+00	0.80	0.705	0.177	0.00E+00	Loops of Henle	Pou3f3
0.00E+00	0.79	0.719	0.101	0.00E+00	Loops of Henle	Abca13
0.00E+00	0.79	0.777	0.394	0.00E+00	Loops of Henle	Aktip
0.00E+00	0.79	0.609	0.169	0.00E+00	Loops of Henle	Sox4
0.00E+00	0.78	0.964	0.826	0.00E+00	Loops of Henle	Idh2
0.00E+00	0.78	0.77	0.455	0.00E+00	Loops of Henle	Egr1
0.00E+00	0.77	0.703	0.13	0.00E+00	Loops of Henle	Scnn1a
0.00E+00	0.76	0.759	0.512	0.00E+00	Loops of Henle	Gls
0.00E+00	0.76	0.944	0.312	0.00E+00	Loops of Henle	Defb1
0.00E+00	0.75	0.669	0.132	0.00E+00	Loops of Henle	Mecom
0.00E+00	0.74	0.756	0.42	0.00E+00	Loops of Henle	Acadsb
0.00E+00	0.74	0.891	0.687	0.00E+00	Loops of Henle	Ivns1abp
0.00E+00	0.73	0.633	0.089	0.00E+00	Loops of Henle	Emb
0.00E+00	0.72	0.676	0.187	0.00E+00	Loops of Henle	Cd82
0.00E+00	0.72	0.933	0.782	0.00E+00	Loops of Henle	Bag1
0.00E+00	0.71	0.754	0.379	0.00E+00	Loops of Henle	Nfe2l1
0.00E+00	0.71	0.607	0.212	0.00E+00	Loops of Henle	Slc9a3
0.00E+00	0.71	0.854	0.524	0.00E+00	Loops of Henle	Gadd45g
0.00E+00	0.70	0.659	0.247	0.00E+00	Loops of Henle	Baiap2l2
0.00E+00	0.70	0.81	0.418	0.00E+00	Loops of Henle	Fabp3

0.00E+00	0.69	0.807	0.506	0.00E+00	Loops of Henle	ccdc198
0.00E+00	0.69	0.694	0.241	0.00E+00	Loops of Henle	Hoxd8
0.00E+00	0.68	0.647	0.229	0.00E+00	Loops of Henle	Stard8
0.00E+00	0.68	0.694	0.26	0.00E+00	Loops of Henle	Wnk4
0.00E+00	0.67	0.928	0.586	0.00E+00	Loops of Henle	Klk1
0.00E+00	0.66	0.709	0.433	0.00E+00	Loops of Henle	Syne2
0.00E+00	0.66	0.671	0.363	0.00E+00	Loops of Henle	Itgb6
0.00E+00	0.66	0.803	0.593	0.00E+00	Loops of Henle	Tfcp2l1
0.00E+00	0.65	0.633	0.325	0.00E+00	Loops of Henle	Lamb1
0.00E+00	0.64	0.653	0.219	0.00E+00	Loops of Henle	Cdkl1
0.00E+00	0.64	0.752	0.145	0.00E+00	Loops of Henle	Tspan8
0.00E+00	0.64	0.689	0.405	0.00E+00	Loops of Henle	Usp2
0.00E+00	0.64	0.657	0.24	0.00E+00	Loops of Henle	Tacc1
0.00E+00	0.62	0.924	0.675	0.00E+00	Loops of Henle	Txnip
0.00E+00	0.62	0.668	0.245	0.00E+00	Loops of Henle	Cdh1
0.00E+00	0.61	0.849	0.657	0.00E+00	Loops of Henle	Spint2
0.00E+00	0.61	0.877	0.723	0.00E+00	Loops of Henle	Arhgap5
0.00E+00	0.60	0.861	0.694	0.00E+00	Loops of Henle	Hadha
0.00E+00	0.59	0.817	0.559	0.00E+00	Loops of Henle	Wwc1
0.00E+00	0.58	0.689	0.36	0.00E+00	Loops of Henle	P3h2
0.00E+00	0.58	0.833	0.651	0.00E+00	Loops of Henle	Camk2n1
0.00E+00	0.57	0.761	0.541	0.00E+00	Loops of Henle	Bicc1
0.00E+00	0.57	0.836	0.661	0.00E+00	Loops of Henle	Fmc1
0.00E+00	0.57	0.627	0.292	0.00E+00	Loops of Henle	Shmt1
0.00E+00	0.56	0.872	0.719	0.00E+00	Loops of Henle	Mgst3
0.00E+00	0.56	0.604	0.269	0.00E+00	Loops of Henle	Tanc1
0.00E+00	0.56	0.978	0.901	0.00E+00	Loops of Henle	Atp5g3
0.00E+00	0.55	0.897	0.802	0.00E+00	Loops of Henle	Sdc4
0.00E+00	0.54	0.762	0.454	0.00E+00	Loops of Henle	Ndrg2
0.00E+00	0.54	0.869	0.726	0.00E+00	Loops of Henle	Cdh16
0.00E+00	0.54	0.992	0.965	0.00E+00	Loops of Henle	Uqcrh
0.00E+00	0.53	0.992	0.99	0.00E+00	Loops of Henle	mt-Nd3
0.00E+00	0.51	0.982	0.924	0.00E+00	Loops of Henle	Aldoa
0.00E+00	0.51	0.987	0.936	0.00E+00	Loops of Henle	Cox7b
0.00E+00	0.50	0.983	0.926	0.00E+00	Loops of Henle	Atp5g1
1.55E-303	0.93	0.738	0.574	4.81E-299	Loops of Henle	Zfp36
1.11E-288	0.78	0.761	0.627	3.44E-284	Loops of Henle	Slc25a30
1.67E-278	0.80	0.663	0.468	5.16E-274	Loops of Henle	Itga6
8.87E-268	0.90	0.767	0.671	2.75E-263	Loops of Henle	Cldn10
1.13E-195	0.59	0.854	0.76	3.49E-191	Loops of Henle	Tsc22d1
0.00E+00	3.72	0.906	0.141	0.00E+00	Distal Tubule	Slc12a3
0.00E+00	3.04	0.876	0.188	0.00E+00	Distal Tubule	Calb1
0.00E+00	2.18	0.969	0.333	0.00E+00	Distal Tubule	Defb1
0.00E+00	2.10	0.952	0.553	0.00E+00	Distal Tubule	Wnk1
0.00E+00	1.98	0.897	0.092	0.00E+00	Distal Tubule	Pgam2
0.00E+00	1.96	0.932	0.599	0.00E+00	Distal Tubule	Klk1
0.00E+00	1.90	0.948	0.152	0.00E+00	Distal Tubule	Tmem52b
0.00E+00	1.87	0.846	0.189	0.00E+00	Distal Tubule	Clu
0.00E+00	1.70	0.919	0.225	0.00E+00	Distal Tubule	Tmem213
0.00E+00	1.65	0.727	0.123	0.00E+00	Distal Tubule	Abca13
0.00E+00	1.49	0.91	0.315	0.00E+00	Distal Tubule	Kng2
0.00E+00	1.48	0.931	0.251	0.00E+00	Distal Tubule	Ppp1r1a
0.00E+00	1.44	0.671	0.072	0.00E+00	Distal Tubule	Sfrp1
0.00E+00	1.38	0.987	0.726	0.00E+00	Distal Tubule	Mt1
0.00E+00	1.36	0.906	0.309	0.00E+00	Distal Tubule	Pkm
0.00E+00	1.35	0.995	0.961	0.00E+00	Distal Tubule	Atp1a1
0.00E+00	1.28	0.82	0.179	0.00E+00	Distal Tubule	Kcnj1
0.00E+00	1.27	0.837	0.172	0.00E+00	Distal Tubule	Clcnkb
0.00E+00	1.21	0.698	0.161	0.00E+00	Distal Tubule	Klhl3
0.00E+00	1.16	0.792	0.25	0.00E+00	Distal Tubule	Hoxd8
0.00E+00	1.16	0.729	0.156	0.00E+00	Distal Tubule	Slc16a7
0.00E+00	1.14	0.804	0.299	0.00E+00	Distal Tubule	Wfdc15b
0.00E+00	1.11	0.916	0.28	0.00E+00	Distal Tubule	Mal
0.00E+00	1.10	0.706	0.148	0.00E+00	Distal Tubule	Emx1

0.00E+00	1.08	0.77	0.272	0.00E+00	Distal Tubule	Mt2
0.00E+00	1.07	0.862	0.342	0.00E+00	Distal Tubule	Wfdc2
0.00E+00	1.06	0.841	0.43	0.00E+00	Distal Tubule	Fabp3
0.00E+00	1.04	0.665	0.364	0.00E+00	Distal Tubule	Sgms2
0.00E+00	1.03	0.946	0.81	0.00E+00	Distal Tubule	S100a1
0.00E+00	1.00	0.704	0.275	0.00E+00	Distal Tubule	Wnk4
0.00E+00	0.97	0.773	0.39	0.00E+00	Distal Tubule	Ier3
0.00E+00	0.93	0.642	0.266	0.00E+00	Distal Tubule	Ckb
0.00E+00	0.88	0.999	0.976	0.00E+00	Distal Tubule	Fxyd2
0.00E+00	0.86	0.674	0.153	0.00E+00	Distal Tubule	Scnn1a
0.00E+00	0.86	0.64	0.133	0.00E+00	Distal Tubule	Pcolce
0.00E+00	0.86	0.612	0.107	0.00E+00	Distal Tubule	Cldn8
0.00E+00	0.86	0.858	0.391	0.00E+00	Distal Tubule	Sh3bgrl3
0.00E+00	0.86	0.713	0.267	0.00E+00	Distal Tubule	Gsta4
0.00E+00	0.83	0.77	0.287	0.00E+00	Distal Tubule	Egf
0.00E+00	0.82	0.657	0.287	0.00E+00	Distal Tubule	Papss1
0.00E+00	0.81	0.95	0.832	0.00E+00	Distal Tubule	Idh2
0.00E+00	0.80	0.929	0.852	0.00E+00	Distal Tubule	Gabarapl1
0.00E+00	0.79	0.916	0.634	0.00E+00	Distal Tubule	Nudt4
0.00E+00	0.78	0.652	0.142	0.00E+00	Distal Tubule	Tmem72
0.00E+00	0.77	0.98	0.964	0.00E+00	Distal Tubule	Atp5e
0.00E+00	0.76	0.984	0.938	0.00E+00	Distal Tubule	Cox7b
0.00E+00	0.75	0.968	0.905	0.00E+00	Distal Tubule	Atp5k
0.00E+00	0.73	0.993	0.927	0.00E+00	Distal Tubule	Ldhb
0.00E+00	0.72	0.994	0.98	0.00E+00	Distal Tubule	Cox6c
0.00E+00	0.71	0.73	0.361	0.00E+00	Distal Tubule	Cd9
0.00E+00	0.71	0.947	0.876	0.00E+00	Distal Tubule	Ndufa1
0.00E+00	0.70	0.969	0.896	0.00E+00	Distal Tubule	Atp1b1
0.00E+00	0.67	0.974	0.929	0.00E+00	Distal Tubule	Atp5g1
0.00E+00	0.65	0.749	0.409	0.00E+00	Distal Tubule	Paqr5
0.00E+00	0.64	0.949	0.877	0.00E+00	Distal Tubule	Ndufc1
0.00E+00	0.63	0.981	0.961	0.00E+00	Distal Tubule	Atp5h
0.00E+00	0.62	0.984	0.96	0.00E+00	Distal Tubule	Cox5b
0.00E+00	0.60	0.973	0.939	0.00E+00	Distal Tubule	Uqcrq
0.00E+00	0.60	0.978	0.956	0.00E+00	Distal Tubule	Cox6b1
0.00E+00	0.60	0.894	0.555	0.00E+00	Distal Tubule	Umod
0.00E+00	0.59	0.977	0.954	0.00E+00	Distal Tubule	Atp5j
0.00E+00	0.58	0.998	0.987	0.00E+00	Distal Tubule	Cox8a
0.00E+00	0.57	0.641	0.223	0.00E+00	Distal Tubule	Efhd1
0.00E+00	0.53	0.647	0.19	0.00E+00	Distal Tubule	Epcam
1.19E-304	0.54	0.975	0.94	3.68E-300	Distal Tubule	Uqcr11
1.84E-297	0.50	0.659	0.277	5.69E-293	Distal Tubule	Aif1l
1.19E-292	0.51	0.977	0.965	3.68E-288	Distal Tubule	Cox7a2
3.47E-290	0.56	0.748	0.415	1.07E-285	Distal Tubule	Mrps6
5.00E-288	0.55	0.959	0.906	1.55E-283	Distal Tubule	Atp5g3
1.78E-269	0.68	0.616	0.297	5.53E-265	Distal Tubule	Cdo1
1.37E-267	0.52	0.964	0.928	4.23E-263	Distal Tubule	Aldoa
2.32E-259	0.50	0.968	0.95	7.19E-255	Distal Tubule	Atp5j2
7.43E-259	0.66	0.763	0.521	2.30E-254	Distal Tubule	ccdc198
1.74E-258	2.45	0.882	0.814	5.40E-254	Distal Tubule	S100g
2.04E-255	0.60	0.713	0.394	6.32E-251	Distal Tubule	Acss1
6.80E-246	0.53	0.949	0.91	2.11E-241	Distal Tubule	Ndufb11
4.28E-231	0.59	0.884	0.791	1.33E-226	Distal Tubule	Bag1
3.50E-229	0.60	0.674	0.439	1.09E-224	Distal Tubule	Acadsb
1.79E-224	0.54	0.892	0.827	5.55E-220	Distal Tubule	Ndufa11
1.56E-223	0.80	0.646	0.457	4.83E-219	Distal Tubule	Trpm7
9.93E-222	0.59	0.937	0.871	3.08E-217	Distal Tubule	Oxct1
6.02E-218	0.58	0.881	0.807	1.86E-213	Distal Tubule	Sdc4
8.62E-214	0.51	0.928	0.875	2.67E-209	Distal Tubule	Tle5
6.51E-203	0.57	0.837	0.728	2.02E-198	Distal Tubule	Mgst3
5.76E-201	0.50	0.946	0.909	1.78E-196	Distal Tubule	Atp5o.1
1.40E-198	0.51	0.85	0.784	4.33E-194	Distal Tubule	Atp6v0e
8.22E-187	0.50	0.891	0.823	2.55E-182	Distal Tubule	Prdx2
1.44E-186	0.54	0.865	0.689	4.47E-182	Distal Tubule	Txnip

6.95E-182	0.68	0.918	0.725	2.15E-177	Distal Tubule	Spp1
3.07E-171	0.50	0.772	0.67	9.52E-167	Distal Tubule	Spint2
3.67E-165	0.51	0.7	0.514	1.14E-160	Distal Tubule	Gstt1
2.48E-129	0.85	0.67	0.581	7.67E-125	Distal Tubule	Kl
6.49E-76	0.80	0.657	0.583	2.01E-71	Distal Tubule	Fos
0.00E+00	3.74	0.967	0.128	0.00E+00	Podocyte-1	Plat
0.00E+00	3.53	0.995	0.059	0.00E+00	Podocyte-1	Emcn
0.00E+00	3.28	0.996	0.067	0.00E+00	Podocyte-1	Plpp1
0.00E+00	3.18	0.925	0.063	0.00E+00	Podocyte-1	Ehd3
0.00E+00	3.09	0.978	0.04	0.00E+00	Podocyte-1	Kdr
0.00E+00	2.81	0.98	0.224	0.00E+00	Podocyte-1	Plpp3
0.00E+00	2.71	0.984	0.16	0.00E+00	Podocyte-1	Ly6c1
0.00E+00	2.65	0.964	0.061	0.00E+00	Podocyte-1	Egfl7
0.00E+00	2.54	0.814	0.29	0.00E+00	Podocyte-1	Igfbp5
0.00E+00	2.54	0.96	0.268	0.00E+00	Podocyte-1	Nrp1
0.00E+00	2.47	0.805	0.045	0.00E+00	Podocyte-1	Fabp4
0.00E+00	2.46	0.914	0.036	0.00E+00	Podocyte-1	Ctla2a
0.00E+00	2.43	0.952	0.044	0.00E+00	Podocyte-1	Eng
0.00E+00	2.37	0.93	0.02	0.00E+00	Podocyte-1	Cd300lg
0.00E+00	2.33	0.932	0.039	0.00E+00	Podocyte-1	Flt1
0.00E+00	2.28	0.961	0.076	0.00E+00	Podocyte-1	Meis2
0.00E+00	2.27	0.964	0.132	0.00E+00	Podocyte-1	Slc9a3r2
0.00E+00	2.17	0.911	0.039	0.00E+00	Podocyte-1	Gimap4
0.00E+00	2.14	0.935	0.04	0.00E+00	Podocyte-1	Ptprb
0.00E+00	2.12	0.911	0.098	0.00E+00	Podocyte-1	Ramp2
0.00E+00	2.08	0.738	0.014	0.00E+00	Podocyte-1	Gpihbp1
0.00E+00	2.06	0.94	0.081	0.00E+00	Podocyte-1	S100a6
0.00E+00	2.05	0.981	0.212	0.00E+00	Podocyte-1	Klf2
0.00E+00	2.05	0.961	0.093	0.00E+00	Podocyte-1	Srgn
0.00E+00	2.01	0.78	0.031	0.00E+00	Podocyte-1	Pi16
0.00E+00	2.01	0.954	0.158	0.00E+00	Podocyte-1	Sparc
0.00E+00	2.00	0.848	0.048	0.00E+00	Podocyte-1	Dlc1
0.00E+00	1.96	0.915	0.119	0.00E+00	Podocyte-1	Cd200
0.00E+00	1.96	0.917	0.043	0.00E+00	Podocyte-1	Pecam1
0.00E+00	1.95	0.95	0.106	0.00E+00	Podocyte-1	Ifitm3
0.00E+00	1.93	0.896	0.059	0.00E+00	Podocyte-1	Adgrf5
0.00E+00	1.92	0.888	0.04	0.00E+00	Podocyte-1	Gimap6
0.00E+00	1.91	0.95	0.113	0.00E+00	Podocyte-1	Tm4sf1
0.00E+00	1.90	0.858	0.133	0.00E+00	Podocyte-1	Hspb1
0.00E+00	1.87	0.897	0.068	0.00E+00	Podocyte-1	Esam
0.00E+00	1.87	0.928	0.201	0.00E+00	Podocyte-1	Epas1
0.00E+00	1.87	0.87	0.088	0.00E+00	Podocyte-1	Gng11
0.00E+00	1.86	0.988	0.675	0.00E+00	Podocyte-1	Serinc3
0.00E+00	1.86	0.837	0.027	0.00E+00	Podocyte-1	AU021092
0.00E+00	1.80	0.821	0.027	0.00E+00	Podocyte-1	Gimap5
0.00E+00	1.77	0.788	0.124	0.00E+00	Podocyte-1	Tcim
0.00E+00	1.73	0.926	0.197	0.00E+00	Podocyte-1	Crip2
0.00E+00	1.73	0.841	0.025	0.00E+00	Podocyte-1	Cyyr1
0.00E+00	1.73	0.851	0.029	0.00E+00	Podocyte-1	Cdh5
0.00E+00	1.73	0.685	0.03	0.00E+00	Podocyte-1	Ednrb
0.00E+00	1.66	0.875	0.42	0.00E+00	Podocyte-1	Pbx1
0.00E+00	1.64	0.734	0.029	0.00E+00	Podocyte-1	Dkk2
0.00E+00	1.64	0.898	0.147	0.00E+00	Podocyte-1	Podxl
0.00E+00	1.64	0.652	0.02	0.00E+00	Podocyte-1	Cyp26b1
0.00E+00	1.64	0.767	0.024	0.00E+00	Podocyte-1	Tmem204
0.00E+00	1.63	0.964	0.623	0.00E+00	Podocyte-1	Gnai2
0.00E+00	1.63	0.917	0.281	0.00E+00	Podocyte-1	Cd24a
0.00E+00	1.63	0.966	0.609	0.00E+00	Podocyte-1	H2-D1
0.00E+00	1.61	0.928	0.53	0.00E+00	Podocyte-1	Sgk1
0.00E+00	1.61	0.807	0.03	0.00E+00	Podocyte-1	Icam2
0.00E+00	1.61	0.963	0.444	0.00E+00	Podocyte-1	Ly6e
0.00E+00	1.59	0.755	0.032	0.00E+00	Podocyte-1	Slfn5
0.00E+00	1.59	0.819	0.033	0.00E+00	Podocyte-1	Adgrl4
0.00E+00	1.55	0.794	0.03	0.00E+00	Podocyte-1	Mmrn2

0.00E+00	1.55	0.953	0.394	0.00E+00	Podocyte-1	Ifitm2
0.00E+00	1.53	0.791	0.253	0.00E+00	Podocyte-1	Cdkn1a
0.00E+00	1.51	0.698	0.008	0.00E+00	Podocyte-1	Fam167b
0.00E+00	1.51	0.822	0.293	0.00E+00	Podocyte-1	Snrk
0.00E+00	1.50	0.793	0.091	0.00E+00	Podocyte-1	Ehd4
0.00E+00	1.50	0.867	0.482	0.00E+00	Podocyte-1	Clic4
0.00E+00	1.49	0.774	0.053	0.00E+00	Podocyte-1	Gimap1
0.00E+00	1.47	0.745	0.014	0.00E+00	Podocyte-1	Exoc3l2
0.00E+00	1.47	0.977	0.654	0.00E+00	Podocyte-1	B2m
0.00E+00	1.47	0.762	0.119	0.00E+00	Podocyte-1	Klf7
0.00E+00	1.47	0.771	0.048	0.00E+00	Podocyte-1	Gngt2
0.00E+00	1.46	0.92	0.581	0.00E+00	Podocyte-1	Fkbp1a
0.00E+00	1.44	0.688	0.02	0.00E+00	Podocyte-1	Hecw2
0.00E+00	1.42	0.694	0.037	0.00E+00	Podocyte-1	Tshz2
0.00E+00	1.42	0.719	0.048	0.00E+00	Podocyte-1	Efnb2
0.00E+00	1.41	0.832	0.344	0.00E+00	Podocyte-1	Rbms1
0.00E+00	1.41	0.748	0.026	0.00E+00	Podocyte-1	Slc43a3
0.00E+00	1.41	0.698	0.022	0.00E+00	Podocyte-1	Rasgrp3
0.00E+00	1.40	0.866	0.422	0.00E+00	Podocyte-1	Id1
0.00E+00	1.39	0.718	0.028	0.00E+00	Podocyte-1	F2r
0.00E+00	1.39	0.711	0.23	0.00E+00	Podocyte-1	Klf4
0.00E+00	1.39	0.708	0.027	0.00E+00	Podocyte-1	Clec1a
0.00E+00	1.38	0.932	0.6	0.00E+00	Podocyte-1	Id3
0.00E+00	1.37	0.874	0.398	0.00E+00	Podocyte-1	S100a13
0.00E+00	1.36	0.953	0.727	0.00E+00	Podocyte-1	Laptm4a
0.00E+00	1.36	0.795	0.269	0.00E+00	Podocyte-1	Ece1
0.00E+00	1.36	0.783	0.069	0.00E+00	Podocyte-1	Anxa3
0.00E+00	1.36	0.73	0.041	0.00E+00	Podocyte-1	Cavin3
0.00E+00	1.35	0.787	0.283	0.00E+00	Podocyte-1	Tcf4
0.00E+00	1.34	0.957	0.756	0.00E+00	Podocyte-1	Sptbn1
0.00E+00	1.33	0.847	0.406	0.00E+00	Podocyte-1	Mxd4
0.00E+00	1.32	0.772	0.323	0.00E+00	Podocyte-1	Dusp3
0.00E+00	1.32	0.79	0.281	0.00E+00	Podocyte-1	Ccdc85b
0.00E+00	1.31	0.718	0.024	0.00E+00	Podocyte-1	Cd34
0.00E+00	1.31	0.839	0.393	0.00E+00	Podocyte-1	Rhob
0.00E+00	1.30	0.706	0.143	0.00E+00	Podocyte-1	Cmtm8
0.00E+00	1.29	0.647	0.163	0.00E+00	Podocyte-1	Cavin2
0.00E+00	1.29	0.69	0.151	0.00E+00	Podocyte-1	Tspan7
0.00E+00	1.29	0.811	0.339	0.00E+00	Podocyte-1	S100a16
0.00E+00	1.29	0.714	0.106	0.00E+00	Podocyte-1	Syne1
0.00E+00	1.28	0.716	0.095	0.00E+00	Podocyte-1	Fas
0.00E+00	1.28	0.854	0.519	0.00E+00	Podocyte-1	Cd47
0.00E+00	1.27	0.728	0.157	0.00E+00	Podocyte-1	Tinagl1
0.00E+00	1.27	0.736	0.287	0.00E+00	Podocyte-1	Lifr
0.00E+00	1.26	0.732	0.049	0.00E+00	Podocyte-1	Arhgap31
0.00E+00	1.26	0.682	0.041	0.00E+00	Podocyte-1	Cbfa2t3
0.00E+00	1.25	0.8	0.448	0.00E+00	Podocyte-1	Ppp1r2
0.00E+00	1.25	0.726	0.235	0.00E+00	Podocyte-1	Myo1c
0.00E+00	1.24	0.676	0.028	0.00E+00	Podocyte-1	Bvht
0.00E+00	1.24	0.627	0.016	0.00E+00	Podocyte-1	Sox18
0.00E+00	1.24	0.876	0.541	0.00E+00	Podocyte-1	Tagln2
0.00E+00	1.23	0.645	0.102	0.00E+00	Podocyte-1	Slco2a1
0.00E+00	1.23	0.787	0.08	0.00E+00	Podocyte-1	Fxyd5
0.00E+00	1.23	0.674	0.034	0.00E+00	Podocyte-1	Heg1
0.00E+00	1.22	0.951	0.684	0.00E+00	Podocyte-1	H2-K1
0.00E+00	1.22	0.743	0.179	0.00E+00	Podocyte-1	Eva1b
0.00E+00	1.21	0.984	0.874	0.00E+00	Podocyte-1	Cd81
0.00E+00	1.21	0.724	0.178	0.00E+00	Podocyte-1	Fam117b
0.00E+00	1.21	0.688	0.242	0.00E+00	Podocyte-1	Ntn4
0.00E+00	1.21	0.872	0.69	0.00E+00	Podocyte-1	Lpl
0.00E+00	1.20	0.687	0.096	0.00E+00	Podocyte-1	Adgre5
0.00E+00	1.20	0.748	0.255	0.00E+00	Podocyte-1	Tpm4
0.00E+00	1.20	0.654	0.023	0.00E+00	Podocyte-1	Myct1
0.00E+00	1.20	0.775	0.146	0.00E+00	Podocyte-1	Anxa2

0.00E+00	1.20	0.659	0.021	0.00E+00	Podocyte-1	Tek
0.00E+00	1.19	0.738	0.315	0.00E+00	Podocyte-1	Col4a1
0.00E+00	1.19	0.732	0.199	0.00E+00	Podocyte-1	Vamp5
0.00E+00	1.19	0.95	0.805	0.00E+00	Podocyte-1	Calm1
0.00E+00	1.18	0.666	0.066	0.00E+00	Podocyte-1	Notch1
0.00E+00	1.17	0.642	0.023	0.00E+00	Podocyte-1	Tie1
0.00E+00	1.16	0.681	0.058	0.00E+00	Podocyte-1	Cald1
0.00E+00	1.16	0.701	0.27	0.00E+00	Podocyte-1	Clic5
0.00E+00	1.16	0.762	0.34	0.00E+00	Podocyte-1	Msn
0.00E+00	1.16	0.608	0.025	0.00E+00	Podocyte-1	Tbx3
0.00E+00	1.16	0.649	0.029	0.00E+00	Podocyte-1	S1pr1
0.00E+00	1.16	0.627	0.012	0.00E+00	Podocyte-1	Cd38
0.00E+00	1.15	0.653	0.024	0.00E+00	Podocyte-1	Rasip1
0.00E+00	1.15	0.887	0.577	0.00E+00	Podocyte-1	Clic1
0.00E+00	1.15	0.69	0.182	0.00E+00	Podocyte-1	Bnip2
0.00E+00	1.15	0.694	0.112	0.00E+00	Podocyte-1	Bst2
0.00E+00	1.14	0.73	0.192	0.00E+00	Podocyte-1	Rras
0.00E+00	1.14	0.631	0.056	0.00E+00	Podocyte-1	Lmcd1
0.00E+00	1.14	0.789	0.499	0.00E+00	Podocyte-1	Qk
0.00E+00	1.13	0.636	0.072	0.00E+00	Podocyte-1	Smad6
0.00E+00	1.13	0.666	0.112	0.00E+00	Podocyte-1	Ddah2
0.00E+00	1.13	0.905	0.707	0.00E+00	Podocyte-1	Dynll1
0.00E+00	1.12	0.654	0.054	0.00E+00	Podocyte-1	Kank3
0.00E+00	1.12	0.823	0.52	0.00E+00	Podocyte-1	Itgb1
0.00E+00	1.12	0.641	0.046	0.00E+00	Podocyte-1	Elk3
0.00E+00	1.11	0.661	0.073	0.00E+00	Podocyte-1	Tgfb1
0.00E+00	1.11	0.629	0.034	0.00E+00	Podocyte-1	Grasp
0.00E+00	1.11	0.676	0.085	0.00E+00	Podocyte-1	Tspan15
0.00E+00	1.10	0.853	0.63	0.00E+00	Podocyte-1	Drap1
0.00E+00	1.08	0.617	0.027	0.00E+00	Podocyte-1	Lrrc8c
0.00E+00	1.08	0.84	0.709	0.00E+00	Podocyte-1	Rdx
0.00E+00	1.08	0.66	0.208	0.00E+00	Podocyte-1	Limch1
0.00E+00	1.08	0.613	0.025	0.00E+00	Podocyte-1	Cldn15
0.00E+00	1.07	0.627	0.073	0.00E+00	Podocyte-1	Tuba1a
0.00E+00	1.07	0.654	0.062	0.00E+00	Podocyte-1	Mef2c
0.00E+00	1.05	0.705	0.31	0.00E+00	Podocyte-1	Bmpr2
0.00E+00	1.05	0.665	0.239	0.00E+00	Podocyte-1	Inpp5k
0.00E+00	1.05	0.695	0.305	0.00E+00	Podocyte-1	Mef2a
0.00E+00	1.04	0.72	0.279	0.00E+00	Podocyte-1	Tnfrsf1a
0.00E+00	1.04	0.876	0.506	0.00E+00	Podocyte-1	Nfkbia
0.00E+00	1.03	0.602	0.022	0.00E+00	Podocyte-1	Arap3
0.00E+00	1.03	0.67	0.242	0.00E+00	Podocyte-1	Plscr1
0.00E+00	1.03	0.738	0.321	0.00E+00	Podocyte-1	Aopep
0.00E+00	1.02	0.719	0.436	0.00E+00	Podocyte-1	Hipk2
0.00E+00	1.02	0.638	0.215	0.00E+00	Podocyte-1	Ptprg
0.00E+00	1.02	0.704	0.306	0.00E+00	Podocyte-1	Klf13
0.00E+00	1.01	0.945	0.688	0.00E+00	Podocyte-1	Txnip
0.00E+00	1.01	0.976	0.931	0.00E+00	Podocyte-1	Gnas
0.00E+00	1.01	0.629	0.188	0.00E+00	Podocyte-1	Adgrl2
0.00E+00	1.00	0.782	0.51	0.00E+00	Podocyte-1	Fermt2
0.00E+00	0.99	0.649	0.253	0.00E+00	Podocyte-1	Clec2d
0.00E+00	0.99	0.623	0.16	0.00E+00	Podocyte-1	931406P16Ri
0.00E+00	0.99	0.772	0.368	0.00E+00	Podocyte-1	Cd9
0.00E+00	0.98	0.74	0.423	0.00E+00	Podocyte-1	Macf1
0.00E+00	0.98	0.98	0.508	0.00E+00	Podocyte-1	Tmsb4x
0.00E+00	0.97	0.866	0.64	0.00E+00	Podocyte-1	Myl12a
0.00E+00	0.97	0.994	0.969	0.00E+00	Podocyte-1	Ptma
0.00E+00	0.96	0.696	0.313	0.00E+00	Podocyte-1	Lims2
0.00E+00	0.96	0.648	0.204	0.00E+00	Podocyte-1	Ets2
0.00E+00	0.96	0.754	0.302	0.00E+00	Podocyte-1	Arpc1b
0.00E+00	0.96	0.989	0.964	0.00E+00	Podocyte-1	Actb
0.00E+00	0.95	0.617	0.079	0.00E+00	Podocyte-1	Plekho1
0.00E+00	0.95	0.776	0.424	0.00E+00	Podocyte-1	Tmem50a
0.00E+00	0.95	0.671	0.298	0.00E+00	Podocyte-1	Serpinh1

0.00E+00	0.95	0.616	0.247	0.00E+00	Podocyte-1	Col4a2
0.00E+00	0.94	0.603	0.149	0.00E+00	Podocyte-1	Arl4d
0.00E+00	0.94	0.64	0.146	0.00E+00	Podocyte-1	Tnfaip2
0.00E+00	0.93	0.621	0.174	0.00E+00	Podocyte-1	Tgfbr2
0.00E+00	0.93	0.681	0.318	0.00E+00	Podocyte-1	H2-T23
0.00E+00	0.93	0.778	0.53	0.00E+00	Podocyte-1	Gnb1
0.00E+00	0.92	0.687	0.183	0.00E+00	Podocyte-1	Bcam
0.00E+00	0.92	0.674	0.28	0.00E+00	Podocyte-1	Luzp1
0.00E+00	0.91	0.613	0.215	0.00E+00	Podocyte-1	810025M15Ri
0.00E+00	0.91	0.634	0.149	0.00E+00	Podocyte-1	Mfge8
0.00E+00	0.91	0.78	0.539	0.00E+00	Podocyte-1	Rap1b
0.00E+00	0.90	0.609	0.167	0.00E+00	Podocyte-1	Asap1
0.00E+00	0.89	0.871	0.71	0.00E+00	Podocyte-1	Calm2
0.00E+00	0.88	0.826	0.678	0.00E+00	Podocyte-1	Mtch1
0.00E+00	0.88	0.883	0.745	0.00E+00	Podocyte-1	Gnb2
0.00E+00	0.87	0.871	0.71	0.00E+00	Podocyte-1	Arpc3
0.00E+00	0.87	0.874	0.604	0.00E+00	Podocyte-1	Dusp1
0.00E+00	0.86	0.913	0.752	0.00E+00	Podocyte-1	S100a10
0.00E+00	0.81	0.626	0.25	0.00E+00	Podocyte-1	Serpinb6a
0.00E+00	0.80	0.617	0.234	0.00E+00	Podocyte-1	Leprot
0.00E+00	0.74	0.959	0.857	0.00E+00	Podocyte-1	App
0.00E+00	0.71	0.967	0.918	0.00E+00	Podocyte-1	H3f3a
0.00E+00	0.70	0.967	0.935	0.00E+00	Podocyte-1	Myl6
0.00E+00	0.70	0.851	0.403	0.00E+00	Podocyte-1	Sh3bgrl3
0.00E+00	0.66	0.979	0.957	0.00E+00	Podocyte-1	Rps3
0.00E+00	0.66	0.681	0.162	0.00E+00	Podocyte-1	Crip1
0.00E+00	0.59	0.99	0.962	0.00E+00	Podocyte-1	Rpl9
0.00E+00	0.58	0.995	0.984	0.00E+00	Podocyte-1	Eif1
2.20E-307	0.88	0.708	0.385	6.83E-303	Podocyte-1	Septin7
1.63E-304	0.85	0.977	0.893	5.06E-300	Podocyte-1	Timp3
1.90E-302	0.93	0.7	0.399	5.89E-298	Podocyte-1	Jup
1.90E-299	0.91	0.617	0.272	5.88E-295	Podocyte-1	Hyal2
2.45E-299	0.92	0.728	0.433	7.59E-295	Podocyte-1	Tpm3
3.39E-293	0.90	0.774	0.504	1.05E-288	Podocyte-1	Selenom
1.28E-291	0.55	0.981	0.956	3.97E-287	Podocyte-1	Rps4x
1.38E-290	0.73	0.844	0.744	4.29E-286	Podocyte-1	Selenok
4.93E-289	0.73	0.883	0.817	1.53E-284	Podocyte-1	Rhoa
1.34E-281	0.95	0.739	0.478	4.16E-277	Podocyte-1	Mgll
1.51E-280	0.77	0.851	0.755	4.67E-276	Podocyte-1	Arpc2
5.72E-280	0.54	0.984	0.945	1.77E-275	Podocyte-1	Rpl21
1.72E-276	0.65	0.962	0.931	5.34E-272	Podocyte-1	H3f3b
1.81E-276	0.85	0.65	0.335	5.62E-272	Podocyte-1	Wasf2
2.12E-272	0.81	0.601	0.271	6.55E-268	Podocyte-1	Arhgap29
3.04E-272	0.70	0.885	0.795	9.40E-268	Podocyte-1	Cfl1
8.65E-272	0.81	0.61	0.292	2.68E-267	Podocyte-1	Apbb2
4.49E-270	0.78	0.737	0.416	1.39E-265	Podocyte-1	Anxa5
2.73E-269	0.84	0.89	0.82	8.47E-265	Podocyte-1	Plscr2
1.62E-263	0.76	0.636	0.284	5.02E-259	Podocyte-1	Ier5
6.48E-262	0.81	0.671	0.369	2.01E-257	Podocyte-1	Cd151
9.69E-260	0.55	0.991	0.976	3.00E-255	Podocyte-1	Rps27
4.20E-259	0.76	0.63	0.287	1.30E-254	Podocyte-1	Tsc22d3
2.74E-255	0.83	0.735	0.514	8.49E-251	Podocyte-1	Tprgl
7.95E-253	0.80	0.644	0.344	2.46E-248	Podocyte-1	Akap13
2.97E-249	0.65	0.879	0.8	9.20E-245	Podocyte-1	Rbm39
3.31E-249	0.90	0.687	0.436	1.03E-244	Podocyte-1	Sh3bp5
7.38E-249	0.67	0.863	0.76	2.29E-244	Podocyte-1	Son
3.42E-247	0.76	0.78	0.655	1.06E-242	Podocyte-1	Rac1
1.09E-244	0.52	0.994	0.985	3.38E-240	Podocyte-1	Rps8
1.53E-242	0.57	0.954	0.903	4.73E-238	Podocyte-1	Ddx5
6.92E-240	0.75	0.777	0.644	2.15E-235	Podocyte-1	Sh3glb1
7.43E-239	0.91	0.76	0.547	2.30E-234	Podocyte-1	Rhoc
1.06E-238	0.70	0.745	0.443	3.29E-234	Podocyte-1	Selenow
1.82E-236	0.55	0.949	0.914	5.65E-232	Podocyte-1	Rpl3
9.18E-235	0.73	0.607	0.293	2.84E-230	Podocyte-1	Smco4

1.12E-229	0.72	0.793	0.708	3.46E-225	Podocyte-1	Csnk1a1
7.87E-229	0.76	0.757	0.622	2.44E-224	Podocyte-1	Ywhab
2.38E-225	0.74	0.623	0.335	7.38E-221	Podocyte-1	Mapk3
1.13E-222	0.80	0.607	0.337	3.49E-218	Podocyte-1	Elf1
7.22E-222	0.77	0.602	0.325	2.24E-217	Podocyte-1	Xiap
9.56E-222	0.51	0.978	0.981	2.96E-217	Podocyte-1	Hsp90ab1
2.06E-216	0.52	0.97	0.943	6.40E-212	Podocyte-1	Rpl34
3.82E-211	0.76	0.691	0.456	1.18E-206	Podocyte-1	F11r
1.56E-210	0.70	0.924	0.645	4.84E-206	Podocyte-1	Ly6a
1.29E-208	0.75	0.669	0.449	3.99E-204	Podocyte-1	Gna11
9.28E-208	0.80	0.638	0.4	2.87E-203	Podocyte-1	Pitpnc1
6.10E-198	0.61	0.834	0.771	1.89E-193	Podocyte-1	Hmgb1
2.66E-196	0.78	0.697	0.49	8.25E-192	Podocyte-1	Arglu1
3.57E-194	0.75	0.607	0.355	1.11E-189	Podocyte-1	Sgms1
4.12E-189	0.78	0.681	0.515	1.28E-184	Podocyte-1	Cyb5r3
2.72E-187	0.65	0.795	0.714	8.41E-183	Podocyte-1	Pomp
1.44E-186	0.66	0.75	0.64	4.46E-182	Podocyte-1	Prkar1a
1.54E-185	0.59	0.809	0.733	4.78E-181	Podocyte-1	Cdc42
1.60E-184	0.52	0.871	0.805	4.96E-180	Podocyte-1	Rabac1
2.55E-183	0.62	0.757	0.634	7.90E-179	Podocyte-1	Arhgdia
8.85E-181	0.83	0.671	0.423	2.74E-176	Podocyte-1	Ier2
8.39E-179	0.52	0.872	0.8	2.60E-174	Podocyte-1	Rsrp1
2.57E-178	0.71	0.682	0.515	7.95E-174	Podocyte-1	Sptan1
5.34E-178	0.63	0.753	0.622	1.65E-173	Podocyte-1	810037I17Ri
4.25E-175	0.58	0.789	0.7	1.32E-170	Podocyte-1	Ctnnb1
9.71E-174	0.61	0.828	0.724	3.01E-169	Podocyte-1	Tspo
1.24E-173	0.56	0.812	0.759	3.84E-169	Podocyte-1	Cnbp
1.58E-173	0.82	0.647	0.478	4.90E-169	Podocyte-1	Mapt
4.58E-172	0.71	0.647	0.461	1.42E-167	Podocyte-1	Ppp3ca
1.43E-170	0.66	0.694	0.528	4.44E-166	Podocyte-1	Ostf1
3.64E-170	0.75	0.729	0.538	1.13E-165	Podocyte-1	Ccnd1
3.76E-170	0.66	0.804	0.785	1.16E-165	Podocyte-1	H2az1
5.01E-167	0.71	0.602	0.391	1.55E-162	Podocyte-1	Ctdspl
7.52E-166	0.67	0.652	0.428	2.33E-161	Podocyte-1	Shisa5
3.08E-163	0.65	0.701	0.454	9.53E-159	Podocyte-1	Ucp2
3.58E-160	0.59	0.748	0.62	1.11E-155	Podocyte-1	Srsf3
4.46E-160	0.69	0.647	0.464	1.38E-155	Podocyte-1	Nfib
6.28E-160	0.64	0.641	0.432	1.95E-155	Podocyte-1	Myl12b
5.32E-158	0.67	0.636	0.455	1.65E-153	Podocyte-1	Pitpna
3.88E-157	0.54	0.805	0.718	1.20E-152	Podocyte-1	Aplp2
3.17E-154	0.61	0.745	0.676	9.82E-150	Podocyte-1	Kif5b
1.48E-153	0.59	0.727	0.625	4.59E-149	Podocyte-1	Snrpb
5.77E-152	0.63	0.641	0.457	1.79E-147	Podocyte-1	Tln1
2.84E-149	0.60	0.675	0.529	8.79E-145	Podocyte-1	Mbd2
7.14E-149	0.51	0.957	0.878	2.21E-144	Podocyte-1	Ctsl
5.01E-145	0.68	0.67	0.572	1.55E-140	Podocyte-1	Arhgef12
9.38E-145	0.51	0.789	0.775	2.91E-140	Podocyte-1	Morf4l1
3.30E-144	0.51	0.787	0.742	1.02E-139	Podocyte-1	Rbm3
4.58E-143	0.69	0.865	0.769	1.42E-138	Podocyte-1	Jun
8.69E-143	0.62	0.633	0.468	2.69E-138	Podocyte-1	Sbds
2.00E-142	0.54	0.756	0.704	6.20E-138	Podocyte-1	Arf5
2.12E-142	0.59	0.699	0.599	6.56E-138	Podocyte-1	Pura
6.53E-141	0.54	0.71	0.508	2.02E-136	Podocyte-1	S100a11
1.63E-140	0.66	0.674	0.519	5.05E-136	Podocyte-1	Atrx
4.52E-132	0.68	0.675	0.547	1.40E-127	Podocyte-1	Srsf7
7.23E-131	0.59	0.606	0.433	2.24E-126	Podocyte-1	230219D22Ri
5.80E-130	0.92	0.706	0.546	1.80E-125	Podocyte-1	Junb
2.33E-129	0.55	0.649	0.497	7.23E-125	Podocyte-1	Syf2
4.58E-128	0.61	0.642	0.489	1.42E-123	Podocyte-1	Lmna
4.17E-127	0.58	0.636	0.489	1.29E-122	Podocyte-1	Rap1a
8.82E-127	0.56	0.733	0.658	2.73E-122	Podocyte-1	Pttg1ip
1.50E-124	0.58	0.67	0.552	4.65E-120	Podocyte-1	Kmt2e
7.68E-119	0.59	0.666	0.576	2.38E-114	Podocyte-1	Purb
1.48E-116	0.53	0.713	0.664	4.59E-112	Podocyte-1	Rrbp1

4.21E-115	0.56	0.647	0.519	1.30E-110	Podocyte-1	Hras
2.08E-113	0.55	0.617	0.475	6.46E-109	Podocyte-1	Sfr1
1.23E-112	0.55	0.722	0.662	3.80E-108	Podocyte-1	Fus
1.41E-112	0.56	0.705	0.635	4.37E-108	Podocyte-1	Mcl1
6.80E-112	0.52	0.823	0.767	2.11E-107	Podocyte-1	Tsc22d1
1.43E-111	0.57	0.603	0.451	4.42E-107	Podocyte-1	Nucb1
1.08E-110	0.55	0.634	0.512	3.34E-106	Podocyte-1	Rock1
5.34E-110	0.52	0.707	0.635	1.65E-105	Podocyte-1	Sf3b1
2.57E-109	0.57	0.842	0.854	7.97E-105	Podocyte-1	Jund
4.13E-108	0.56	0.615	0.438	1.28E-103	Podocyte-1	Slc38a2
2.22E-104	0.50	0.709	0.662	6.87E-100	Podocyte-1	Tmem234
1.33E-103	0.54	0.627	0.492	4.13E-99	Podocyte-1	Mbnl2
9.94E-103	0.52	0.619	0.492	3.08E-98	Podocyte-1	Ccdc12
4.59E-97	0.50	0.635	0.527	1.42E-92	Podocyte-1	Tmed9
1.09E-86	0.51	0.637	0.57	3.38E-82	Podocyte-1	Lrrc58
3.63E-83	0.53	0.602	0.515	1.12E-78	Podocyte-1	Reep3
6.64E-56	0.53	0.664	0.584	2.06E-51	Podocyte-1	Fos
0.00E+00	3.29	0.911	0.127	0.00E+00	Podocyte-2	Cdkn1c
0.00E+00	3.04	0.926	0.079	0.00E+00	Podocyte-2	Nphs2
0.00E+00	2.97	0.869	0.13	0.00E+00	Podocyte-2	H2-Q7
0.00E+00	2.96	0.873	0.252	0.00E+00	Podocyte-2	EGFP
0.00E+00	2.93	0.941	0.173	0.00E+00	Podocyte-2	Podxl
0.00E+00	2.75	0.837	0.047	0.00E+00	Podocyte-2	Clic3
0.00E+00	2.73	0.903	0.103	0.00E+00	Podocyte-2	Nupr1
0.00E+00	2.67	0.883	0.12	0.00E+00	Podocyte-2	H2-Q6
0.00E+00	2.50	0.847	0.03	0.00E+00	Podocyte-2	Nphs1
0.00E+00	2.50	0.894	0.188	0.00E+00	Podocyte-2	Sparc
0.00E+00	2.29	0.811	0.088	0.00E+00	Podocyte-2	Rasl11a
0.00E+00	2.27	0.816	0.048	0.00E+00	Podocyte-2	Thsd7a
0.00E+00	2.25	0.79	0.047	0.00E+00	Podocyte-2	Cldn5
0.00E+00	2.21	0.803	0.064	0.00E+00	Podocyte-2	Sema3g
0.00E+00	2.12	0.856	0.094	0.00E+00	Podocyte-2	Synpo
0.00E+00	2.08	0.854	0.177	0.00E+00	Podocyte-2	Mafb
0.00E+00	2.08	0.801	0.167	0.00E+00	Podocyte-2	Gsn
0.00E+00	2.05	0.85	0.206	0.00E+00	Podocyte-2	Dpp4
0.00E+00	2.00	0.847	0.031	0.00E+00	Podocyte-2	Wt1
0.00E+00	1.94	0.765	0.132	0.00E+00	Podocyte-2	Magi2
0.00E+00	1.92	0.725	0.034	0.00E+00	Podocyte-2	Ptpro
0.00E+00	1.89	0.873	0.23	0.00E+00	Podocyte-2	Tmsb10
0.00E+00	1.89	0.712	0.023	0.00E+00	Podocyte-2	Rab3b
0.00E+00	1.88	0.847	0.145	0.00E+00	Podocyte-2	Tm4sf1
0.00E+00	1.86	0.758	0.071	0.00E+00	Podocyte-2	Angptl2
0.00E+00	1.79	0.729	0.096	0.00E+00	Podocyte-2	Cmtm7
0.00E+00	1.76	0.674	0.017	0.00E+00	Podocyte-2	Tcf21
0.00E+00	1.71	0.733	0.076	0.00E+00	Podocyte-2	Nes
0.00E+00	1.69	0.78	0.168	0.00E+00	Podocyte-2	Anxa2
0.00E+00	1.68	0.739	0.123	0.00E+00	Podocyte-2	Golim4
0.00E+00	1.68	0.659	0.024	0.00E+00	Podocyte-2	Mmp23
0.00E+00	1.66	0.689	0.031	0.00E+00	Podocyte-2	Hs3st6
0.00E+00	1.65	0.695	0.036	0.00E+00	Podocyte-2	Srgap1
0.00E+00	1.64	0.731	0.067	0.00E+00	Podocyte-2	Rhpn1
0.00E+00	1.61	0.716	0.033	0.00E+00	Podocyte-2	Tmem150c
0.00E+00	1.51	0.678	0.06	0.00E+00	Podocyte-2	Gpsm3
0.00E+00	1.50	0.61	0.028	0.00E+00	Podocyte-2	Aplp1
0.00E+00	1.41	0.729	0.101	0.00E+00	Podocyte-2	Vim
0.00E+00	1.38	0.64	0.035	0.00E+00	Podocyte-2	Myom2
0.00E+00	1.37	0.612	0.015	0.00E+00	Podocyte-2	Ddn
0.00E+00	1.36	0.617	0.027	0.00E+00	Podocyte-2	Shisa3
0.00E+00	1.36	0.669	0.107	0.00E+00	Podocyte-2	Tspan15
0.00E+00	1.33	0.627	0.05	0.00E+00	Podocyte-2	Npr1
0.00E+00	1.31	0.667	0.097	0.00E+00	Podocyte-2	Anxa3
0.00E+00	1.25	0.85	0.16	0.00E+00	Podocyte-2	Plat
0.00E+00	1.24	0.631	0.094	0.00E+00	Podocyte-2	Marveld1
1.49E-305	2.12	0.792	0.2	4.62E-301	Podocyte-2	Bcam

4.42E-293	1.36	0.712	0.157	1.37E-288	Podocyte-2	Lrrfip1
3.26E-281	2.29	0.831	0.273	1.01E-276	Podocyte-2	Enpep
6.35E-270	1.87	0.809	0.249	1.97E-265	Podocyte-2	Cd59a
8.15E-269	2.03	0.742	0.196	2.52E-264	Podocyte-2	Aebp1
4.11E-266	2.63	0.987	0.525	1.27E-261	Podocyte-2	Tmsb4x
1.11E-260	1.46	0.744	0.204	3.43E-256	Podocyte-2	Tmod3
4.11E-259	1.13	0.744	0.166	1.27E-254	Podocyte-2	Slc9a3r2
2.27E-244	1.61	0.682	0.176	7.05E-240	Podocyte-2	Metrnl
5.22E-243	1.58	0.784	0.237	1.62E-238	Podocyte-2	Cryab
2.10E-239	1.30	0.682	0.177	6.52E-235	Podocyte-2	Efnb1
3.63E-227	1.33	0.695	0.195	1.12E-222	Podocyte-2	Ripor1
1.45E-221	1.78	0.903	0.51	4.49E-217	Podocyte-2	Vegfa
3.34E-219	1.94	0.858	0.396	1.03E-214	Podocyte-2	Itgb5
1.64E-215	1.25	0.627	0.161	5.08E-211	Podocyte-2	Mgat5
3.19E-213	1.16	0.614	0.151	9.90E-209	Podocyte-2	Pdlim2
4.47E-205	1.46	0.735	0.25	1.38E-200	Podocyte-2	Gas1
2.02E-196	1.74	0.867	0.415	6.26E-192	Podocyte-2	Ifitm2
1.67E-195	1.64	0.807	0.351	5.17E-191	Podocyte-2	Npnt
1.48E-189	1.33	0.633	0.189	4.60E-185	Podocyte-2	Plod2
2.82E-186	1.57	0.784	0.335	8.74E-182	Podocyte-2	Gadd45a
7.73E-186	1.36	0.733	0.276	2.39E-181	Podocyte-2	Septin11
8.10E-184	1.72	0.809	0.392	2.51E-179	Podocyte-2	Col4a3
8.95E-183	2.08	0.992	0.922	2.77E-178	Podocyte-2	Igfbp7
8.59E-182	1.15	0.636	0.189	2.66E-177	Podocyte-2	St3gal6
7.87E-173	1.39	0.731	0.285	2.44E-168	Podocyte-2	Clic5
8.98E-169	1.33	0.775	0.335	2.78E-164	Podocyte-2	Aopep
8.77E-168	1.38	0.731	0.314	2.72E-163	Podocyte-2	Mpp5
4.58E-163	1.42	0.703	0.274	1.42E-158	Podocyte-2	Ifngr1
2.25E-162	1.44	0.966	0.81	6.98E-158	Podocyte-2	Sdc4
5.65E-161	1.76	0.994	0.99	1.75E-156	Podocyte-2	Itm2b
6.31E-156	1.23	0.941	0.798	1.96E-151	Podocyte-2	Pth1r
1.22E-154	1.55	0.873	0.654	3.77E-150	Podocyte-2	Eif3m
3.30E-152	1.11	0.64	0.228	1.02E-147	Podocyte-2	Mob2
1.01E-151	1.57	0.807	0.47	3.14E-147	Podocyte-2	Htra1
2.35E-148	1.59	0.898	0.715	7.27E-144	Podocyte-2	Calm2
3.53E-148	1.62	0.833	0.513	1.09E-143	Podocyte-2	S100a11
3.66E-148	1.16	0.966	0.936	1.13E-143	Podocyte-2	Myl6
1.92E-145	1.45	0.731	0.356	5.95E-141	Podocyte-2	Man1a2
1.38E-141	1.02	0.621	0.227	4.28E-137	Podocyte-2	Pak1
4.92E-140	1.66	0.939	0.879	1.52E-135	Podocyte-2	Cd81
3.27E-137	1.56	0.9	0.736	1.01E-132	Podocyte-2	Laptm4a
5.05E-135	1.48	0.858	0.648	1.56E-130	Podocyte-2	Myl12a
8.99E-134	1.25	0.799	0.515	2.79E-129	Podocyte-2	Wsb2
2.77E-129	1.16	0.672	0.305	8.59E-125	Podocyte-2	Iqgap2
2.99E-126	1.51	0.716	0.38	9.26E-122	Podocyte-2	Cd151
3.87E-124	1.08	0.731	0.364	1.20E-119	Podocyte-2	Pmepa1
2.81E-118	1.01	0.619	0.263	8.71E-114	Podocyte-2	Nsf
4.16E-118	1.30	0.79	0.524	1.29E-113	Podocyte-2	Col4a4
1.41E-115	1.10	0.78	0.481	4.37E-111	Podocyte-2	Mapt
4.07E-115	1.16	0.686	0.348	1.26E-110	Podocyte-2	Ildr2
9.55E-114	1.14	0.727	0.426	2.96E-109	Podocyte-2	Arhgap24
1.84E-109	0.93	0.665	0.317	5.69E-105	Podocyte-2	Mylk
1.93E-108	1.32	0.773	0.531	5.96E-104	Podocyte-2	Itgb1
2.99E-108	0.90	0.689	0.298	9.25E-104	Podocyte-2	Aif1l
1.82E-105	0.99	0.956	0.92	5.65E-101	Podocyte-2	H3f3a
5.78E-104	1.10	0.636	0.296	1.79E-99	Podocyte-2	Col18a1
3.40E-102	1.06	0.661	0.356	1.05E-97	Podocyte-2	Parva
1.27E-101	1.13	0.644	0.299	3.94E-97	Podocyte-2	Tsc22d3
5.10E-99	1.17	0.68	0.385	1.58E-94	Podocyte-2	Dag1
3.82E-96	0.91	0.818	0.66	1.18E-91	Podocyte-2	Actn4
9.79E-92	1.04	0.862	0.832	3.03E-87	Podocyte-2	Ndfip1
1.11E-91	1.16	0.672	0.412	3.44E-87	Podocyte-2	Cpq
7.37E-91	1.06	0.934	0.881	2.28E-86	Podocyte-2	Ctsl
8.18E-90	0.96	0.758	0.462	2.53E-85	Podocyte-2	Ucp2

4.02E-89	1.08	0.797	0.683	1.25E-84	Podocyte-2	Mtch1
1.86E-88	0.93	0.617	0.295	5.76E-84	Podocyte-2	Gsta4
1.11E-86	0.85	0.833	0.71	3.45E-82	Podocyte-2	Csnk1a1
3.78E-85	0.95	0.742	0.51	1.17E-80	Podocyte-2	Qk
6.65E-85	1.02	0.646	0.362	2.06E-80	Podocyte-2	Palld
5.40E-81	1.13	0.646	0.368	1.67E-76	Podocyte-2	Kcnq1ot1
1.13E-79	0.93	0.608	0.323	3.49E-75	Podocyte-2	Tpm1
1.01E-75	0.85	0.881	0.846	3.13E-71	Podocyte-2	Tmem59
2.32E-74	1.00	0.807	0.625	7.19E-70	Podocyte-2	H2-D1
3.92E-73	0.68	0.941	0.933	1.22E-68	Podocyte-2	Gnas
2.01E-70	0.59	0.97	0.946	6.24E-66	Podocyte-2	Rpl21
6.94E-68	0.88	0.729	0.564	2.15E-63	Podocyte-2	Cd2ap
1.68E-66	0.99	0.623	0.391	5.21E-62	Podocyte-2	P3h2
2.55E-64	0.89	0.799	0.76	7.89E-60	Podocyte-2	Selenof
3.33E-64	0.79	0.97	0.965	1.03E-59	Podocyte-2	Actb
1.22E-62	0.77	0.833	0.823	3.79E-58	Podocyte-2	Vdac2
1.32E-59	0.83	0.657	0.439	4.09E-55	Podocyte-2	Tmem50a
1.59E-59	0.72	0.841	0.697	4.93E-55	Podocyte-2	Lpl
2.45E-59	0.79	0.646	0.447	7.59E-55	Podocyte-2	Mtss1
1.04E-58	0.69	0.809	0.787	3.23E-54	Podocyte-2	Dad1
7.28E-58	0.73	0.811	0.759	2.26E-53	Podocyte-2	Arpc2
1.15E-56	0.88	0.629	0.439	3.57E-52	Podocyte-2	Pdia6
2.02E-56	0.81	0.672	0.52	6.27E-52	Podocyte-2	Slc48a1
8.85E-56	0.67	0.875	0.862	2.74E-51	Podocyte-2	App
3.06E-52	0.70	0.809	0.788	9.49E-48	Podocyte-2	Atp6v0e
5.34E-52	0.59	0.907	0.877	1.65E-47	Podocyte-2	Ptms
9.69E-49	0.63	0.858	0.769	3.00E-44	Podocyte-2	Tsc22d1
1.19E-48	0.74	0.75	0.678	3.68E-44	Podocyte-2	Kif5b
3.56E-48	0.78	0.669	0.563	1.10E-43	Podocyte-2	Nap1l1
4.19E-48	0.85	0.703	0.627	1.30E-43	Podocyte-2	810037I17Ri
8.18E-45	0.71	0.805	0.809	2.53E-40	Podocyte-2	Rabac1
1.32E-44	0.76	0.659	0.558	4.08E-40	Podocyte-2	Degs1
1.28E-43	0.57	0.788	0.716	3.96E-39	Podocyte-2	Dynll1
3.20E-39	0.61	0.602	0.454	9.91E-35	Podocyte-2	Ptprd
4.17E-39	0.75	0.629	0.531	1.29E-34	Podocyte-2	Tmed9
5.75E-39	0.61	0.775	0.722	1.78E-34	Podocyte-2	Aplp2
6.28E-39	0.57	0.773	0.697	1.95E-34	Podocyte-2	H2-K1
6.18E-38	0.54	0.803	0.765	1.91E-33	Podocyte-2	Son
1.34E-36	0.65	0.703	0.661	4.16E-32	Podocyte-2	Pttg1ip
3.57E-36	0.62	0.657	0.592	1.11E-31	Podocyte-2	Emc7
4.83E-35	0.54	0.761	0.75	1.50E-30	Podocyte-2	Sumo2
3.54E-34	0.55	0.646	0.522	1.10E-29	Podocyte-2	Fermt2
4.51E-33	0.72	0.629	0.556	1.40E-28	Podocyte-2	Smim1
3.55E-32	0.75	0.617	0.522	1.10E-27	Podocyte-2	Cyb5r3
1.66E-31	0.57	0.674	0.645	5.14E-27	Podocyte-2	BC031181
2.23E-31	0.64	0.665	0.58	6.92E-27	Podocyte-2	Ctsh
3.66E-31	0.57	0.68	0.628	1.14E-26	Podocyte-2	Wls
1.75E-30	0.57	0.691	0.65	5.41E-26	Podocyte-2	Sh3glb1
1.96E-29	0.57	0.682	0.662	6.08E-25	Podocyte-2	Spcs1
8.62E-29	0.51	0.739	0.745	2.67E-24	Podocyte-2	Rbm3
3.07E-27	0.52	0.75	0.741	9.52E-23	Podocyte-2	Hsp90b1
3.30E-27	0.54	0.638	0.568	1.02E-22	Podocyte-2	Reep5
4.62E-26	0.50	0.693	0.68	1.43E-21	Podocyte-2	Tspan3
1.04E-22	0.53	0.655	0.64	3.21E-18	Podocyte-2	Arhgdia
5.42E-19	0.51	0.627	0.622	1.68E-14	Podocyte-2	Sap18
1.30E-17	0.51	0.604	0.577	4.03E-13	Podocyte-2	Arhgef12
0.00E+00	3.13	0.677	0.023	0.00E+00	Podocyte-3	Mgp
0.00E+00	2.80	0.973	0.098	0.00E+00	Podocyte-3	Vim
0.00E+00	2.77	1	0.143	0.00E+00	Podocyte-3	Tm4sf1
0.00E+00	2.56	0.975	0.176	0.00E+00	Podocyte-3	Crip1
0.00E+00	2.52	0.993	0.19	0.00E+00	Podocyte-3	Ly6c1
0.00E+00	2.51	0.998	0.24	0.00E+00	Podocyte-3	Klf2
0.00E+00	2.51	0.941	0.244	0.00E+00	Podocyte-3	Klf4
0.00E+00	2.51	0.977	0.112	0.00E+00	Podocyte-3	S100a6

0.00E+00	2.51	0.81	0.201	0.00E+00	Podocyte-3	Tspan8
0.00E+00	2.37	0.991	0.136	0.00E+00	Podocyte-3	Ifitm3
0.00E+00	2.12	0.923	0.271	0.00E+00	Podocyte-3	Cdkn1a
0.00E+00	2.00	0.901	0.159	0.00E+00	Podocyte-3	Hspb1
0.00E+00	1.96	0.862	0.017	0.00E+00	Podocyte-3	Fbln5
0.00E+00	1.96	0.98	0.162	0.00E+00	Podocyte-3	Slc9a3r2
0.00E+00	1.94	0.928	0.06	0.00E+00	Podocyte-3	Adgrl4
0.00E+00	1.92	0.788	0.022	0.00E+00	Podocyte-3	S100a4
0.00E+00	1.92	0.914	0.031	0.00E+00	Podocyte-3	Sox17
0.00E+00	1.90	0.919	0.115	0.00E+00	Podocyte-3	Ehd4
0.00E+00	1.90	0.86	0.033	0.00E+00	Podocyte-3	Cd93
0.00E+00	1.89	0.937	0.148	0.00E+00	Podocyte-3	Cd200
0.00E+00	1.88	0.926	0.128	0.00E+00	Podocyte-3	Ramp2
0.00E+00	1.85	0.95	0.228	0.00E+00	Podocyte-3	Epas1
0.00E+00	1.83	0.953	0.223	0.00E+00	Podocyte-3	Crip2
0.00E+00	1.80	0.93	0.175	0.00E+00	Podocyte-3	Tinagl1
0.00E+00	1.80	0.901	0.073	0.00E+00	Podocyte-3	Ptprb
0.00E+00	1.79	0.878	0.049	0.00E+00	Podocyte-3	Ebf1
0.00E+00	1.72	0.919	0.075	0.00E+00	Podocyte-3	Pecam1
0.00E+00	1.69	0.709	0.028	0.00E+00	Podocyte-3	Edn1
0.00E+00	1.69	0.923	0.089	0.00E+00	Podocyte-3	Adgrf5
0.00E+00	1.63	0.912	0.109	0.00E+00	Podocyte-3	Meis2
0.00E+00	1.59	0.957	0.094	0.00E+00	Podocyte-3	Egfl7
0.00E+00	1.57	0.749	0.042	0.00E+00	Podocyte-3	Ltbp4
0.00E+00	1.56	0.808	0.04	0.00E+00	Podocyte-3	Thbd
0.00E+00	1.53	0.912	0.104	0.00E+00	Podocyte-3	Fxyd5
0.00E+00	1.51	0.926	0.098	0.00E+00	Podocyte-3	Esam
0.00E+00	1.48	0.745	0.005	0.00E+00	Podocyte-3	Fbln2
0.00E+00	1.46	0.837	0.048	0.00E+00	Podocyte-3	S1pr1
0.00E+00	1.43	0.792	0.058	0.00E+00	Podocyte-3	Ccnd2
0.00E+00	1.43	0.919	0.188	0.00E+00	Podocyte-3	Sparc
0.00E+00	1.42	0.959	0.125	0.00E+00	Podocyte-3	Srgn
0.00E+00	1.38	0.815	0.06	0.00E+00	Podocyte-3	Cdh5
0.00E+00	1.37	0.842	0.058	0.00E+00	Podocyte-3	Icam2
0.00E+00	1.37	0.856	0.065	0.00E+00	Podocyte-3	Cavin3
0.00E+00	1.36	0.758	0.043	0.00E+00	Podocyte-3	Pltp
0.00E+00	1.36	0.761	0.04	0.00E+00	Podocyte-3	Clec14a
0.00E+00	1.35	0.849	0.074	0.00E+00	Podocyte-3	Flt1
0.00E+00	1.35	0.804	0.056	0.00E+00	Podocyte-3	Acvrl1
0.00E+00	1.35	0.677	0.044	0.00E+00	Podocyte-3	Rsad2
0.00E+00	1.32	0.795	0.052	0.00E+00	Podocyte-3	Lmo2
0.00E+00	1.28	0.817	0.065	0.00E+00	Podocyte-3	Sema3g
0.00E+00	1.28	0.79	0.039	0.00E+00	Podocyte-3	Ecscr
0.00E+00	1.25	0.792	0.059	0.00E+00	Podocyte-3	Pi16
0.00E+00	1.24	0.67	0.081	0.00E+00	Podocyte-3	Jag1
0.00E+00	1.24	0.914	0.174	0.00E+00	Podocyte-3	Podxl
0.00E+00	1.23	0.783	0.083	0.00E+00	Podocyte-3	Emp3
0.00E+00	1.23	0.758	0.056	0.00E+00	Podocyte-3	Heg1
0.00E+00	1.23	0.867	0.079	0.00E+00	Podocyte-3	Eng
0.00E+00	1.22	0.79	0.096	0.00E+00	Podocyte-3	Anxa3
0.00E+00	1.21	0.817	0.073	0.00E+00	Podocyte-3	Gimap6
0.00E+00	1.21	0.727	0.013	0.00E+00	Podocyte-3	Jam2
0.00E+00	1.21	0.668	0.027	0.00E+00	Podocyte-3	Apold1
0.00E+00	1.20	0.659	0.042	0.00E+00	Podocyte-3	Calcrl
0.00E+00	1.20	0.684	0.029	0.00E+00	Podocyte-3	Lrrc32
0.00E+00	1.18	0.749	0.164	0.00E+00	Podocyte-3	Adam15
0.00E+00	1.17	0.795	0.143	0.00E+00	Podocyte-3	Klf7
0.00E+00	1.17	0.756	0.091	0.00E+00	Podocyte-3	Cavin1
0.00E+00	1.16	0.781	0.074	0.00E+00	Podocyte-3	Arhgap31
0.00E+00	1.16	0.743	0.059	0.00E+00	Podocyte-3	Mmrn2
0.00E+00	1.15	0.824	0.168	0.00E+00	Podocyte-3	Anxa2
0.00E+00	1.13	0.747	0.133	0.00E+00	Podocyte-3	Bst2
0.00E+00	1.13	0.659	0.093	0.00E+00	Podocyte-3	Tuba1a
0.00E+00	1.12	0.673	0.056	0.00E+00	Podocyte-3	Gm8995

0.00E+00	1.11	0.774	0.08	0.00E+00	Podocyte-3	Cald1
0.00E+00	1.10	0.731	0.045	0.00E+00	Podocyte-3	Myct1
0.00E+00	1.10	0.666	0.036	0.00E+00	Podocyte-3	Palmd
0.00E+00	1.09	0.698	0.055	0.00E+00	Podocyte-3	Grasp
0.00E+00	1.09	0.625	0.025	0.00E+00	Podocyte-3	Gja4
0.00E+00	1.04	0.725	0.044	0.00E+00	Podocyte-3	Tie1
0.00E+00	1.04	0.713	0.052	0.00E+00	Podocyte-3	Clec1a
0.00E+00	1.03	0.639	0.014	0.00E+00	Podocyte-3	Ptprr
0.00E+00	1.03	0.718	0.043	0.00E+00	Podocyte-3	Sncg
0.00E+00	1.03	0.7	0.058	0.00E+00	Podocyte-3	Ehd2
0.00E+00	0.98	0.657	0.049	0.00E+00	Podocyte-3	Pmp22
0.00E+00	0.97	0.7	0.05	0.00E+00	Podocyte-3	Cd34
0.00E+00	0.97	0.698	0.065	0.00E+00	Podocyte-3	Cav1
0.00E+00	0.96	0.752	0.058	0.00E+00	Podocyte-3	AU021092
0.00E+00	0.96	0.664	0.088	0.00E+00	Podocyte-3	Notch1
0.00E+00	0.96	0.682	0.102	0.00E+00	Podocyte-3	Kitl
0.00E+00	0.95	0.711	0.106	0.00E+00	Podocyte-3	Psmb8
0.00E+00	0.95	0.693	0.046	0.00E+00	Podocyte-3	Rasip1
0.00E+00	0.95	0.682	0.065	0.00E+00	Podocyte-3	Cbfa2t3
0.00E+00	0.94	0.684	0.045	0.00E+00	Podocyte-3	Cldn15
0.00E+00	0.93	0.731	0.081	0.00E+00	Podocyte-3	Gimap1
0.00E+00	0.92	0.661	0.039	0.00E+00	Podocyte-3	Fgd5
0.00E+00	0.92	0.628	0.041	0.00E+00	Podocyte-3	Jcad
0.00E+00	0.92	0.842	0.096	0.00E+00	Podocyte-3	Emcn
0.00E+00	0.91	0.605	0.064	0.00E+00	Podocyte-3	Rasd1
0.00E+00	0.90	0.67	0.097	0.00E+00	Podocyte-3	Mpzl1
0.00E+00	0.90	0.621	0.035	0.00E+00	Podocyte-3	Samd12
0.00E+00	0.89	0.609	0.078	0.00E+00	Podocyte-3	Lmcd1
0.00E+00	0.89	0.603	0.039	0.00E+00	Podocyte-3	Sparcl1
0.00E+00	0.88	0.709	0.083	0.00E+00	Podocyte-3	Mef2c
0.00E+00	0.88	0.666	0.046	0.00E+00	Podocyte-3	Rapgef5
0.00E+00	0.88	0.896	0.103	0.00E+00	Podocyte-3	Plpp1
0.00E+00	0.87	0.659	0.068	0.00E+00	Podocyte-3	Ets1
0.00E+00	0.86	0.673	0.06	0.00E+00	Podocyte-3	Slfn5
0.00E+00	0.86	0.614	0.033	0.00E+00	Podocyte-3	Atp2a3
0.00E+00	0.85	0.657	0.048	0.00E+00	Podocyte-3	Lrrc8c
0.00E+00	0.84	0.637	0.059	0.00E+00	Podocyte-3	Cyyr1
0.00E+00	0.84	0.619	0.073	0.00E+00	Podocyte-3	Dnm3
0.00E+00	0.82	0.625	0.077	0.00E+00	Podocyte-3	Kank3
0.00E+00	0.82	0.65	0.045	0.00E+00	Podocyte-3	Tek
0.00E+00	0.81	0.619	0.054	0.00E+00	Podocyte-3	Amotl1
0.00E+00	0.80	0.734	0.119	0.00E+00	Podocyte-3	Gng11
0.00E+00	0.79	0.628	0.077	0.00E+00	Podocyte-3	Gngt2
0.00E+00	0.75	0.63	0.074	0.00E+00	Podocyte-3	Efnb2
6.07E-306	1.25	0.686	0.121	1.88E-301	Podocyte-3	Slco2a1
1.69E-299	1.24	0.621	0.098	5.25E-295	Podocyte-3	Gm26532
2.21E-291	0.75	0.65	0.11	6.85E-287	Podocyte-3	Kctd10
1.26E-289	0.78	0.643	0.108	3.89E-285	Podocyte-3	Sorbs2
5.39E-289	1.34	0.783	0.178	1.67E-284	Podocyte-3	Cavin2
1.62E-285	0.78	0.661	0.115	5.01E-281	Podocyte-3	Lamb2
5.31E-284	1.04	0.686	0.128	1.64E-279	Podocyte-3	Emp1
4.66E-278	1.14	0.745	0.17	1.44E-273	Podocyte-3	Tspan7
2.47E-275	0.72	0.616	0.095	7.64E-271	Podocyte-3	Tgfb1
1.72E-272	0.86	0.691	0.132	5.33E-268	Podocyte-3	Ddah2
1.74E-272	0.78	0.621	0.109	5.39E-268	Podocyte-3	Cyth3
6.60E-272	0.82	0.643	0.113	2.05E-267	Podocyte-3	H2-Q4
8.56E-272	0.93	0.691	0.138	2.65E-267	Podocyte-3	Utrn
1.52E-266	1.07	0.736	0.175	4.71E-262	Podocyte-3	931406P16Ri
2.16E-266	1.16	0.804	0.211	6.68E-262	Podocyte-3	Rras
6.65E-264	0.78	0.65	0.119	2.06E-259	Podocyte-3	Fas
1.58E-262	1.48	0.98	0.414	4.88E-258	Podocyte-3	Ifitm2
9.04E-261	1.62	0.984	0.463	2.80E-256	Podocyte-3	Ly6e
3.94E-260	0.78	0.657	0.123	1.22E-255	Podocyte-3	Pdgfb
1.13E-257	0.76	0.648	0.119	3.51E-253	Podocyte-3	Adgre5

2.19E-257	1.31	0.901	0.316	6.77E-253	Podocyte-3	Arpc1b
1.38E-252	1.35	0.84	0.285	4.28E-248	Podocyte-3	Foxp1
3.33E-251	1.31	0.842	0.265	1.03E-246	Podocyte-3	Ahnak
1.05E-244	1.04	0.603	0.118	3.27E-240	Podocyte-3	Plac8
4.19E-244	1.40	0.926	0.415	1.30E-239	Podocyte-3	S100a13
1.94E-237	2.12	0.991	0.835	6.02E-233	Podocyte-3	Cst3
1.82E-235	1.50	0.991	0.81	5.63E-231	Podocyte-3	Calm1
2.87E-233	0.95	0.781	0.2	8.90E-229	Podocyte-3	Bcam
5.45E-232	0.88	0.718	0.163	1.69E-227	Podocyte-3	Tnfaip2
3.11E-229	0.78	0.605	0.125	9.63E-225	Podocyte-3	Swap70
4.32E-229	1.35	0.932	0.408	1.34E-224	Podocyte-3	Rhob
1.86E-226	1.29	1	0.525	5.76E-222	Podocyte-3	Tmsb4x
6.07E-225	1.60	0.995	0.654	1.88E-220	Podocyte-3	Ly6a
4.79E-224	1.17	0.74	0.22	1.48E-219	Podocyte-3	Plec
9.57E-222	1.23	0.869	0.356	2.96E-217	Podocyte-3	S100a16
2.31E-221	0.77	0.634	0.13	7.14E-217	Podocyte-3	Syne1
1.97E-220	1.46	1	0.895	6.10E-216	Podocyte-3	Timp3
8.64E-220	1.60	0.989	0.622	2.68E-215	Podocyte-3	H2-D1
5.60E-216	0.88	0.652	0.161	1.74E-211	Podocyte-3	Plscr4
4.96E-214	1.29	0.989	0.735	1.54E-209	Podocyte-3	Laptm4a
1.66E-210	1.14	0.822	0.301	5.14E-206	Podocyte-3	Tcf4
3.90E-209	0.61	0.648	0.135	1.21E-204	Podocyte-3	H2-Q7
2.06E-206	1.34	0.959	0.636	6.39E-202	Podocyte-3	Gnai2
2.47E-205	0.68	0.614	0.125	7.66E-201	Podocyte-3	Zfhx3
6.00E-202	1.23	0.966	0.725	1.86E-197	Podocyte-3	Tspo
2.05E-199	1.25	0.788	0.284	6.36E-195	Podocyte-3	Clic5
3.22E-199	1.02	1	0.97	9.96E-195	Podocyte-3	Ptma
4.34E-198	0.84	0.641	0.168	1.34E-193	Podocyte-3	Dab2ip
1.81E-196	1.08	0.826	0.296	5.62E-192	Podocyte-3	Tsc22d3
3.42E-194	0.64	0.682	0.157	1.06E-189	Podocyte-3	Csrp1
1.03E-192	1.08	0.905	0.425	3.18E-188	Podocyte-3	Anxa5
1.05E-191	1.39	0.883	0.44	3.25E-187	Podocyte-3	Slc38a2
3.30E-191	0.89	0.998	0.976	1.02E-186	Podocyte-3	Rps14
3.35E-191	1.32	0.982	0.693	1.04E-186	Podocyte-3	H2-K1
1.18E-188	1.39	0.937	0.519	3.65E-184	Podocyte-3	Nfkbia
2.46E-188	1.59	0.935	0.664	7.63E-184	Podocyte-3	Slc6a6
2.38E-187	0.84	0.637	0.162	7.38E-183	Podocyte-3	Pcdh17
3.20E-186	0.52	0.603	0.126	9.92E-182	Podocyte-3	H2-Q6
3.23E-186	0.82	0.702	0.205	1.00E-181	Podocyte-3	Tmod3
1.01E-183	1.04	0.63	0.172	3.14E-179	Podocyte-3	Rflnb
2.36E-182	0.92	0.731	0.236	7.30E-178	Podocyte-3	Slc50a1
5.21E-182	0.78	0.619	0.152	1.61E-177	Podocyte-3	Ddit4
3.26E-181	0.83	0.7	0.201	1.01E-176	Podocyte-3	Bnip2
5.18E-180	0.83	0.998	0.963	1.60E-175	Podocyte-3	Rpl9
3.08E-178	0.89	0.675	0.199	9.54E-174	Podocyte-3	Fam117b
4.11E-178	1.04	0.898	0.38	1.27E-173	Podocyte-3	Cd9
7.66E-176	0.98	0.772	0.293	2.37E-171	Podocyte-3	Luzp1
7.93E-176	0.93	0.901	0.305	2.46E-171	Podocyte-3	Cd24a
1.42E-175	1.07	0.619	0.167	4.41E-171	Podocyte-3	Sbno2
6.74E-175	1.29	0.901	0.438	2.09E-170	Podocyte-3	Id1
8.11E-174	0.81	0.998	0.975	2.51E-169	Podocyte-3	Rps20
2.27E-172	0.81	1	0.979	7.04E-168	Podocyte-3	Rps24
3.19E-172	1.00	0.986	0.878	9.89E-168	Podocyte-3	Cd81
5.86E-172	0.71	1	0.996	1.82E-167	Podocyte-3	Tpt1
7.67E-171	1.14	0.962	0.666	2.38E-166	Podocyte-3	B2m
2.05E-170	0.84	1	0.976	6.36E-166	Podocyte-3	Rps27
7.53E-170	0.71	0.609	0.159	2.33E-165	Podocyte-3	Tjp1
2.87E-169	1.18	0.948	0.713	8.88E-165	Podocyte-3	Dynll1
4.54E-169	0.83	0.998	0.965	1.41E-164	Podocyte-3	Rpl18a
2.27E-168	0.78	0.995	0.974	7.02E-164	Podocyte-3	Rps11
5.08E-166	1.09	0.916	0.552	1.57E-161	Podocyte-3	Tagln2
8.85E-165	0.83	0.993	0.956	2.74E-160	Podocyte-3	Rpl13
1.10E-163	0.84	0.995	0.946	3.42E-159	Podocyte-3	Rpl21
1.48E-163	1.06	0.95	0.763	4.57E-159	Podocyte-3	Sptbn1

4.88E-163	1.15	0.767	0.295	1.51E-158	Podocyte-3	Ier5
2.31E-162	0.82	0.993	0.956	7.15E-158	Podocyte-3	Rps4x
1.35E-160	0.89	0.684	0.225	4.17E-156	Podocyte-3	Limch1
4.90E-160	0.79	0.991	0.954	1.52E-155	Podocyte-3	Rps23
1.16E-159	0.79	0.998	0.975	3.58E-155	Podocyte-3	Rps12
1.67E-159	0.73	1	0.985	5.18E-155	Podocyte-3	Eif1
5.51E-159	0.77	1	0.986	1.71E-154	Podocyte-3	Rps8
5.92E-159	1.76	0.758	0.311	1.83E-154	Podocyte-3	Hspa1a
7.96E-159	0.93	0.786	0.335	2.47E-154	Podocyte-3	Tubb5
1.13E-158	1.46	0.738	0.293	3.51E-154	Podocyte-3	Ppp1r15a
1.22E-158	0.81	0.991	0.961	3.77E-154	Podocyte-3	Rpl8
7.46E-157	0.80	0.993	0.963	2.31E-152	Podocyte-3	Rps5
1.13E-156	1.10	0.898	0.528	3.49E-152	Podocyte-3	Btg1
1.19E-153	0.78	0.993	0.957	3.69E-149	Podocyte-3	Rps3
4.11E-153	1.06	0.657	0.221	1.27E-148	Podocyte-3	Ier5l
2.17E-152	1.06	0.856	0.496	6.73E-148	Podocyte-3	Clic4
3.05E-152	0.73	0.998	0.972	9.43E-148	Podocyte-3	Rps3a1
2.06E-151	0.71	0.998	0.972	6.37E-147	Podocyte-3	Rps16
6.26E-151	1.05	0.824	0.442	1.94E-146	Podocyte-3	Tpm3
6.27E-151	1.04	0.777	0.336	1.94E-146	Podocyte-3	Aopep
3.54E-150	1.04	0.659	0.201	1.10E-145	Podocyte-3	Socs3
2.02E-149	0.94	0.761	0.32	6.27E-145	Podocyte-3	Klf13
7.30E-149	0.87	0.777	0.344	2.26E-144	Podocyte-3	Wasf2
8.08E-149	0.84	0.984	0.915	2.50E-144	Podocyte-3	Rpl3
2.59E-148	0.85	0.718	0.277	8.04E-144	Podocyte-3	Septin11
1.33E-147	1.02	0.765	0.353	4.12E-143	Podocyte-3	Akap13
3.51E-146	1.69	0.81	0.412	1.09E-141	Podocyte-3	Ier3
8.53E-146	0.76	0.993	0.943	2.64E-141	Podocyte-3	Rpl34
8.73E-146	0.67	0.993	0.973	2.71E-141	Podocyte-3	Rpl19
1.15E-145	0.97	0.783	0.355	3.56E-141	Podocyte-3	Msn
1.83E-145	0.94	0.824	0.422	5.66E-141	Podocyte-3	Mxd4
2.16E-145	0.88	0.691	0.257	6.70E-141	Podocyte-3	Plscr1
4.24E-144	0.88	0.763	0.343	1.31E-139	Podocyte-3	Mapk3
7.21E-142	0.72	0.609	0.187	2.23E-137	Podocyte-3	Rtl8a
7.03E-141	1.13	0.801	0.438	2.18E-136	Podocyte-3	Pbx1
2.17E-140	1.13	0.673	0.258	6.73E-136	Podocyte-3	Sertad1
2.60E-140	1.43	0.81	0.43	8.04E-136	Podocyte-3	Ier2
2.63E-140	0.67	0.605	0.183	8.13E-136	Podocyte-3	Npdc1
4.23E-138	0.70	0.657	0.219	1.31E-133	Podocyte-3	Trim47
2.38E-137	0.72	0.993	0.901	7.37E-133	Podocyte-3	Rpl18
4.59E-137	0.56	0.711	0.233	1.42E-132	Podocyte-3	Tmsb10
2.24E-135	0.86	0.718	0.314	6.94E-131	Podocyte-3	Arl2bp
2.64E-135	0.94	0.734	0.341	8.19E-131	Podocyte-3	Tnrc6c
3.45E-135	0.70	1	0.985	1.07E-130	Podocyte-3	Rps21
3.26E-134	0.90	0.725	0.325	1.01E-129	Podocyte-3	Klf3
4.53E-134	0.57	1	0.985	1.40E-129	Podocyte-3	Fau
4.76E-134	0.66	0.632	0.206	1.48E-129	Podocyte-3	Il10rb
4.76E-133	0.71	0.991	0.928	1.48E-128	Podocyte-3	Rps19
3.34E-132	0.83	0.747	0.35	1.04E-127	Podocyte-3	Card19
3.92E-132	0.72	0.98	0.9	1.21E-127	Podocyte-3	Rpl7
1.15E-131	0.88	0.67	0.231	3.56E-127	Podocyte-3	Cebpd
2.11E-130	0.87	0.98	0.932	6.55E-126	Podocyte-3	H3f3b
2.82E-130	1.61	0.86	0.549	8.72E-126	Podocyte-3	Junb
1.29E-129	0.83	0.894	0.588	3.99E-125	Podocyte-3	Clic1
6.68E-129	0.76	0.704	0.29	2.07E-124	Podocyte-3	Ece1
2.73E-127	0.72	0.98	0.907	8.45E-123	Podocyte-3	Rps7
5.24E-126	0.58	0.655	0.22	1.62E-121	Podocyte-3	Vamp5
5.86E-126	0.93	0.826	0.539	1.81E-121	Podocyte-3	Gnb1
1.59E-125	0.59	1	0.988	4.94E-121	Podocyte-3	Rplp1
5.95E-125	0.64	0.632	0.183	1.84E-120	Podocyte-3	Mecom
1.26E-123	0.61	0.7	0.252	3.89E-119	Podocyte-3	Cd59a
1.39E-123	0.72	0.702	0.282	4.30E-119	Podocyte-3	Arl6ip5
1.43E-123	0.68	0.998	0.98	4.42E-119	Podocyte-3	Hsp90ab1
1.53E-123	0.90	0.679	0.235	4.75E-119	Podocyte-3	Gadd45b

4.53E-123	0.56	1	0.997	1.40E-118	Podocyte-3	Eef1a1
8.57E-123	0.74	0.948	0.818	2.66E-118	Podocyte-3	Rhoa
2.56E-122	0.83	0.847	0.532	7.93E-118	Podocyte-3	Cd47
5.80E-122	1.07	0.939	0.852	1.80E-117	Podocyte-3	Jund
5.85E-122	0.65	0.986	0.946	1.81E-117	Podocyte-3	Rpl17
6.20E-121	0.61	0.612	0.198	1.92E-116	Podocyte-3	Pls3
2.68E-120	0.81	0.725	0.327	8.30E-116	Podocyte-3	Lims2
3.13E-120	0.76	0.939	0.798	9.68E-116	Podocyte-3	Cfl1
7.19E-118	0.95	0.912	0.613	2.23E-113	Podocyte-3	Id3
2.09E-117	0.84	0.901	0.72	6.48E-113	Podocyte-3	Aplp2
2.75E-117	0.80	0.844	0.453	8.53E-113	Podocyte-3	Selenow
2.79E-117	0.91	0.93	0.758	8.66E-113	Podocyte-3	S100a10
1.14E-116	0.65	0.993	0.939	3.53E-112	Podocyte-3	Rpl30
4.87E-116	0.85	0.885	0.713	1.51E-111	Podocyte-3	Rdx
1.39E-115	0.71	0.707	0.303	4.31E-111	Podocyte-3	Smco4
1.73E-115	0.67	0.695	0.274	5.36E-111	Podocyte-3	Tpm4
4.14E-115	0.93	0.901	0.613	1.28E-110	Podocyte-3	Dusp1
4.31E-115	0.54	0.995	0.976	1.34E-110	Podocyte-3	Rpl26
2.05E-114	0.59	0.88	0.419	6.36E-110	Podocyte-3	Sh3bgrl3
3.58E-114	0.62	0.98	0.961	1.11E-109	Podocyte-3	Rps15
7.97E-114	0.81	0.91	0.715	2.47E-109	Podocyte-3	Calm2
2.17E-113	1.25	0.754	0.376	6.74E-109	Podocyte-3	Fosb
5.00E-113	0.73	0.67	0.284	1.55E-108	Podocyte-3	Hyal2
1.67E-112	0.56	0.984	0.95	5.18E-108	Podocyte-3	Rpsa
2.09E-112	0.64	0.616	0.229	6.46E-108	Podocyte-3	Phc2
7.28E-112	0.63	0.962	0.896	2.25E-107	Podocyte-3	Rpl12
1.04E-111	0.56	0.989	0.967	3.22E-107	Podocyte-3	Rps9
1.49E-111	0.73	0.661	0.286	4.60E-107	Podocyte-3	Gltp
7.37E-110	0.82	0.736	0.391	2.28E-105	Podocyte-3	Arid5b
1.40E-109	0.74	0.652	0.277	4.33E-105	Podocyte-3	Btbd7
3.13E-109	0.65	0.67	0.263	9.68E-105	Podocyte-3	Serpinb6a
3.33E-109	0.72	0.907	0.688	1.03E-104	Podocyte-3	Serinc3
4.45E-109	0.72	0.763	0.396	1.38E-104	Podocyte-3	Septin7
1.14E-108	0.65	0.982	0.919	3.54E-104	Podocyte-3	H3f3a
8.16E-108	0.62	0.966	0.932	2.53E-103	Podocyte-3	Gnas
9.09E-108	1.09	0.837	0.642	2.82E-103	Podocyte-3	Glul
1.51E-107	0.78	0.81	0.319	4.68E-103	Podocyte-3	Mal
2.74E-107	1.11	0.937	0.771	8.49E-103	Podocyte-3	Jun
9.24E-107	0.70	0.763	0.364	2.86E-102	Podocyte-3	Iqgap1
1.69E-106	0.57	0.993	0.971	5.24E-102	Podocyte-3	Rpl35a
2.93E-106	1.46	0.84	0.584	9.09E-102	Podocyte-3	Fos
3.06E-105	0.54	0.995	0.975	9.47E-101	Podocyte-3	Rps27a
1.30E-103	0.69	0.856	0.595	4.02E-99	Podocyte-3	Fkbp1a
2.81E-102	0.90	0.853	0.635	8.72E-98	Podocyte-3	Mcl1
4.37E-102	0.85	0.946	0.698	1.35E-97	Podocyte-3	Txnip
5.41E-101	0.76	0.806	0.519	1.68E-96	Podocyte-3	Cyb5r3
1.94E-100	0.74	0.971	0.905	6.01E-96	Podocyte-3	Ddx5
6.04E-100	0.57	0.973	0.92	1.87E-95	Podocyte-3	Rpl29
6.09E-100	0.68	0.704	0.341	1.89E-95	Podocyte-3	Dusp3
1.09E-99	0.71	0.779	0.437	3.39E-95	Podocyte-3	Tmem50a
1.83E-99	0.61	0.63	0.248	5.68E-95	Podocyte-3	Leprot
2.63E-99	0.57	0.984	0.946	8.14E-95	Podocyte-3	Rpl14
5.70E-99	0.53	0.991	0.961	1.77E-94	Podocyte-3	Rplp2
5.96E-99	0.71	0.883	0.648	1.85E-94	Podocyte-3	Myl12a
2.74E-98	0.70	0.817	0.531	8.49E-94	Podocyte-3	Itgb1
3.99E-98	0.51	0.984	0.946	1.24E-93	Podocyte-3	Rps13
2.42E-97	0.59	0.971	0.934	7.49E-93	Podocyte-3	Rack1
2.81E-97	0.72	0.738	0.407	8.72E-93	Podocyte-3	Ywhah
4.36E-97	0.56	0.998	0.964	1.35E-92	Podocyte-3	Actb
1.70E-96	0.71	0.837	0.659	5.26E-92	Podocyte-3	Rac1
2.31E-96	0.59	0.679	0.302	7.16E-92	Podocyte-3	Ccdc85b
3.02E-96	1.02	0.637	0.285	9.36E-92	Podocyte-3	Hspa1b
5.35E-96	0.51	0.984	0.966	1.66E-91	Podocyte-3	Rps18
3.99E-94	0.64	0.623	0.273	1.24E-89	Podocyte-3	Nfix

6.01E-94	0.73	0.731	0.412	1.86E-89	Podocyte-3	Mprip
9.15E-94	0.66	0.878	0.774	2.83E-89	Podocyte-3	Rps6
2.20E-93	0.98	0.786	0.55	6.80E-89	Podocyte-3	Srsf7
5.27E-93	0.60	0.941	0.857	1.63E-88	Podocyte-3	Rpl27a
1.54E-92	0.98	0.756	0.492	4.78E-88	Podocyte-3	Lmna
1.12E-91	0.75	0.849	0.547	3.47E-87	Podocyte-3	Sgk1
1.85E-91	0.51	0.995	0.984	5.74E-87	Podocyte-3	Rpl37a
5.01E-91	0.63	0.652	0.298	1.55E-86	Podocyte-3	Itm2c
8.24E-91	0.57	0.953	0.867	2.55E-86	Podocyte-3	Rpl5
1.71E-90	0.62	0.896	0.75	5.31E-86	Podocyte-3	Gnb2
1.85E-89	0.53	0.993	0.981	5.73E-85	Podocyte-3	Rpl37
2.78E-89	0.63	0.639	0.268	8.62E-85	Podocyte-3	Clec2d
3.28E-89	0.62	0.894	0.745	1.02E-84	Podocyte-3	Eif3f
3.05E-88	0.67	0.826	0.648	9.45E-84	Podocyte-3	Sh3glb1
1.33E-87	0.61	0.605	0.265	4.11E-83	Podocyte-3	Mast4
8.39E-87	0.65	0.657	0.312	2.60E-82	Podocyte-3	Serpinh1
1.65E-86	0.68	0.754	0.464	5.11E-82	Podocyte-3	F11r
4.84E-85	0.65	0.815	0.513	1.50E-80	Podocyte-3	S100a11
1.83E-84	1.22	0.763	0.484	5.68E-80	Podocyte-3	Egr1
5.95E-84	0.56	0.876	0.747	1.84E-79	Podocyte-3	Selenok
7.31E-84	0.76	0.673	0.384	2.26E-79	Podocyte-3	Tulp4
2.10E-83	0.57	0.892	0.757	6.49E-79	Podocyte-3	Arpc2
2.58E-83	0.71	0.632	0.316	7.98E-79	Podocyte-3	Dennd5b
9.15E-83	0.62	0.652	0.32	2.83E-78	Podocyte-3	Mef2a
9.14E-82	0.73	0.646	0.352	2.83E-77	Podocyte-3	Tut7
1.34E-81	0.56	0.619	0.284	4.16E-77	Podocyte-3	Cr1l
1.73E-81	0.61	0.702	0.38	5.36E-77	Podocyte-3	Cd151
2.95E-81	0.68	0.788	0.58	9.13E-77	Podocyte-3	Cltb
2.70E-80	0.62	0.628	0.301	8.36E-76	Podocyte-3	Tuba1b
7.69E-80	0.61	0.74	0.434	2.38E-75	Podocyte-3	Shisa5
3.59E-79	0.61	0.743	0.462	1.11E-74	Podocyte-3	Ppp1r2
4.65E-79	0.66	0.668	0.349	1.44E-74	Podocyte-3	Nfat5
9.44E-79	0.71	0.851	0.75	2.92E-74	Podocyte-3	Dnaja1
1.57E-78	0.55	0.661	0.332	4.85E-74	Podocyte-3	Col4a1
4.38E-78	0.65	0.833	0.48	1.36E-73	Podocyte-3	Btg2
1.77E-76	0.98	0.664	0.325	5.50E-72	Podocyte-3	Atf3
2.99E-76	0.52	0.914	0.821	9.26E-72	Podocyte-3	Cox7a2l
4.91E-76	0.61	0.616	0.314	1.52E-71	Podocyte-3	Gnaq
5.13E-76	0.54	0.91	0.803	1.59E-71	Podocyte-3	Rbm39
2.64E-75	0.58	0.639	0.28	8.17E-71	Podocyte-3	Tacc1
5.33E-75	0.64	0.711	0.457	1.65E-70	Podocyte-3	Gna11
2.72E-74	0.62	0.738	0.472	8.43E-70	Podocyte-3	Mapre1
8.93E-74	0.61	0.664	0.332	2.77E-69	Podocyte-3	H2-T23
9.55E-74	0.57	0.639	0.327	2.96E-69	Podocyte-3	Nedd9
2.36E-73	0.60	0.673	0.388	7.32E-69	Podocyte-3	Nr3c1
3.81E-73	0.59	0.725	0.457	1.18E-68	Podocyte-3	Ralbp1
9.72E-73	0.67	0.767	0.548	3.01E-68	Podocyte-3	Rap1b
1.24E-72	0.58	0.639	0.326	3.84E-68	Podocyte-3	Bmpr2
2.71E-72	0.59	0.765	0.522	8.39E-68	Podocyte-3	Tprgl
1.32E-71	0.61	0.772	0.57	4.09E-67	Podocyte-3	Rab11a
5.48E-71	0.68	0.684	0.416	1.70E-66	Podocyte-3	Lima1
1.04E-70	0.54	0.883	0.768	3.21E-66	Podocyte-3	Rps10
2.53E-69	0.57	0.661	0.34	7.84E-65	Podocyte-3	Ifi27
3.54E-69	0.59	0.815	0.623	1.10E-64	Podocyte-3	Srsf3
5.88E-68	0.51	0.912	0.873	1.82E-63	Podocyte-3	Rpl4
6.10E-68	0.56	0.835	0.677	1.89E-63	Podocyte-3	Kif5b
4.72E-67	0.60	0.684	0.427	1.46E-62	Podocyte-3	Ybx3
5.48E-67	0.54	0.609	0.319	1.70E-62	Podocyte-3	Cast
6.43E-67	0.55	0.609	0.323	1.99E-62	Podocyte-3	Sertad2
7.51E-67	0.57	0.74	0.501	2.33E-62	Podocyte-3	Syf2
6.27E-66	0.64	0.792	0.556	1.94E-61	Podocyte-3	Gadd45g
5.43E-65	0.55	0.72	0.469	1.68E-60	Podocyte-3	Bcl2l1
1.13E-63	0.58	0.634	0.359	3.49E-59	Podocyte-3	Kras
3.20E-63	0.52	0.903	0.768	9.92E-59	Podocyte-3	Tsc22d1

4.19E-63	0.66	0.63	0.35	1.30E-58	Podocyte-3	Tubb2a
4.44E-63	0.57	0.619	0.326	1.38E-58	Podocyte-3	Kdm6b
3.48E-62	0.56	0.634	0.339	1.08E-57	Podocyte-3	Tapbp
1.27E-61	0.54	0.605	0.327	3.94E-57	Podocyte-3	Arl4a
1.61E-61	0.50	0.619	0.333	4.98E-57	Podocyte-3	Rock2
4.06E-61	0.63	0.679	0.436	1.26E-56	Podocyte-3	Macf1
4.93E-61	0.55	0.718	0.438	1.53E-56	Podocyte-3	Myl12b
7.48E-61	0.73	0.616	0.348	2.32E-56	Podocyte-3	Ppp1r10
1.45E-60	0.57	0.725	0.521	4.49E-56	Podocyte-3	Sptan1
4.09E-60	0.56	0.758	0.555	1.27E-55	Podocyte-3	Kmt2e
1.22E-59	0.51	0.822	0.702	3.78E-55	Podocyte-3	Ctnnb1
1.70E-59	0.63	0.878	0.802	5.27E-55	Podocyte-3	Rsrp1
3.56E-59	0.56	0.752	0.542	1.10E-54	Podocyte-3	Rtn4
4.05E-59	0.50	0.767	0.514	1.25E-54	Podocyte-3	Selenom
4.25E-59	0.53	0.639	0.381	1.32E-54	Podocyte-3	Zbtb7a
3.27E-58	0.59	0.698	0.452	1.01E-53	Podocyte-3	Tra2a
5.84E-58	0.51	0.72	0.445	1.81E-53	Podocyte-3	Psme1
4.82E-57	0.72	0.684	0.492	1.49E-52	Podocyte-3	Ccnl1
3.16E-56	0.57	0.632	0.37	9.78E-52	Podocyte-3	Ankrd12
4.10E-55	0.52	0.804	0.638	1.27E-50	Podocyte-3	Arhgdia
1.53E-54	0.61	0.749	0.588	4.75E-50	Podocyte-3	Clk1
2.41E-54	0.58	0.781	0.663	7.46E-50	Podocyte-3	Fus
5.51E-54	0.61	0.874	0.823	1.71E-49	Podocyte-3	Plscr2
7.54E-53	0.51	0.686	0.47	2.34E-48	Podocyte-3	Mbnl1
1.19E-52	0.64	0.752	0.616	3.69E-48	Podocyte-3	Brd2
1.75E-51	0.68	0.673	0.468	5.42E-47	Podocyte-3	Dnajb9
6.42E-51	0.57	0.684	0.479	1.99E-46	Podocyte-3	Sfr1
3.19E-50	0.56	0.727	0.578	9.87E-46	Podocyte-3	Purb
9.21E-49	0.65	0.725	0.591	2.85E-44	Podocyte-3	Atf4
2.00E-47	0.55	0.664	0.468	6.19E-43	Podocyte-3	Rfk
1.72E-46	0.54	0.664	0.488	5.32E-42	Podocyte-3	Nek7
3.93E-45	0.56	0.781	0.703	1.22E-40	Podocyte-3	Srsf2
8.98E-44	0.51	0.679	0.503	2.78E-39	Podocyte-3	Nop53
1.06E-42	0.51	0.643	0.424	3.29E-38	Podocyte-3	Resf1
4.45E-41	0.55	0.738	0.589	1.38E-36	Podocyte-3	Zfp36
1.23E-40	0.57	0.736	0.621	3.82E-36	Podocyte-3	Tubb4b
2.32E-39	0.64	0.856	0.879	7.19E-35	Podocyte-3	Hsp90aa1
0.00E+00	4.33	0.969	0.028	0.00E+00	Podocyte-4	Acta2
0.00E+00	3.92	0.986	0.017	0.00E+00	Podocyte-4	Rgs5
0.00E+00	3.91	0.969	0.018	0.00E+00	Podocyte-4	Tagln
0.00E+00	3.76	0.969	0.02	0.00E+00	Podocyte-4	Myl9
0.00E+00	3.34	0.917	0.024	0.00E+00	Podocyte-4	Tpm2
0.00E+00	3.05	0.969	0.006	0.00E+00	Podocyte-4	Myh11
0.00E+00	2.96	0.955	0.181	0.00E+00	Podocyte-4	Crip1
0.00E+00	2.95	0.955	0.039	0.00E+00	Podocyte-4	Sparcl1
0.00E+00	2.74	0.941	0.012	0.00E+00	Podocyte-4	Mustn1
0.00E+00	2.54	0.955	0.082	0.00E+00	Podocyte-4	Cald1
0.00E+00	2.22	0.824	0.018	0.00E+00	Podocyte-4	Gm13889
0.00E+00	2.12	0.866	0.104	0.00E+00	Podocyte-4	Vim
0.00E+00	2.08	0.921	0.09	0.00E+00	Podocyte-4	Flna
0.00E+00	1.95	0.893	0.031	0.00E+00	Podocyte-4	Pcp4l1
0.00E+00	1.89	0.797	0.002	0.00E+00	Podocyte-4	Rergl
0.00E+00	1.87	0.876	0.017	0.00E+00	Podocyte-4	Fxyd1
0.00E+00	1.87	0.903	0.085	0.00E+00	Podocyte-4	Mef2c
0.00E+00	1.86	0.931	0.149	0.00E+00	Podocyte-4	Tm4sf1
0.00E+00	1.83	0.641	0.027	0.00E+00	Podocyte-4	Mgp
0.00E+00	1.82	0.917	0.054	0.00E+00	Podocyte-4	Ebf1
0.00E+00	1.77	0.862	0.007	0.00E+00	Podocyte-4	Notch3
0.00E+00	1.76	0.89	0.125	0.00E+00	Podocyte-4	Zfhx3
0.00E+00	1.73	0.717	0.007	0.00E+00	Podocyte-4	Ndufa4l2
0.00E+00	1.72	0.703	0.005	0.00E+00	Podocyte-4	Pln
0.00E+00	1.68	0.828	0.109	0.00E+00	Podocyte-4	Sorbs2
0.00E+00	1.66	0.845	0.045	0.00E+00	Podocyte-4	Filip1l
0.00E+00	1.60	0.807	0.094	0.00E+00	Podocyte-4	Rasl11a

0.00E+00	1.58	0.817	0.024	0.00E+00	Podocyte-4	Hopx
0.00E+00	1.57	0.834	0.094	0.00E+00	Podocyte-4	Cavin1
0.00E+00	1.55	0.748	0.046	0.00E+00	Podocyte-4	Sncg
0.00E+00	1.55	0.707	0.005	0.00E+00	Podocyte-4	Ppp1r14a
0.00E+00	1.53	0.803	0.002	0.00E+00	Podocyte-4	Lmod1
0.00E+00	1.52	0.81	0.086	0.00E+00	Podocyte-4	Ptp4a3
0.00E+00	1.51	0.8	0.07	0.00E+00	Podocyte-4	Cavin3
0.00E+00	1.37	0.779	0.067	0.00E+00	Podocyte-4	Lbh
0.00E+00	1.37	0.783	0.115	0.00E+00	Podocyte-4	Actn1
0.00E+00	1.36	0.762	0.017	0.00E+00	Podocyte-4	Pde3a
0.00E+00	1.34	0.697	0.031	0.00E+00	Podocyte-4	Bgn
0.00E+00	1.32	0.848	0.116	0.00E+00	Podocyte-4	Lamb2
0.00E+00	1.29	0.766	0.022	0.00E+00	Podocyte-4	Atp1b2
0.00E+00	1.28	0.672	0.015	0.00E+00	Podocyte-4	Myom1
0.00E+00	1.27	0.748	0.024	0.00E+00	Podocyte-4	Cenpa
0.00E+00	1.27	0.721	0.012	0.00E+00	Podocyte-4	Nrip2
0.00E+00	1.26	0.721	0.011	0.00E+00	Podocyte-4	Pdlim3
0.00E+00	1.25	0.738	0.055	0.00E+00	Podocyte-4	Ppp1r12b
0.00E+00	1.24	0.714	0.002	0.00E+00	Podocyte-4	Rgs7bp
0.00E+00	1.24	0.745	0.039	0.00E+00	Podocyte-4	Mcam
0.00E+00	1.22	0.659	0.003	0.00E+00	Podocyte-4	Cox4i2
0.00E+00	1.21	0.631	0.006	0.00E+00	Podocyte-4	S1pr3
0.00E+00	1.19	0.752	0.067	0.00E+00	Podocyte-4	Fry
0.00E+00	1.13	0.672	0.003	0.00E+00	Podocyte-4	Gpc6
0.00E+00	1.13	0.655	0.002	0.00E+00	Podocyte-4	Atp1a2
0.00E+00	1.13	0.738	0.08	0.00E+00	Podocyte-4	Map3k20
0.00E+00	1.12	0.659	0.064	0.00E+00	Podocyte-4	Ccnd2
0.00E+00	1.07	0.603	0.001	0.00E+00	Podocyte-4	Olfr558
0.00E+00	1.06	0.607	0.021	0.00E+00	Podocyte-4	Nexn
0.00E+00	1.05	0.634	0.014	0.00E+00	Podocyte-4	Cfh
0.00E+00	1.04	0.659	0.037	0.00E+00	Podocyte-4	Rcan2
0.00E+00	1.01	0.645	0.021	0.00E+00	Podocyte-4	Ltbp1
0.00E+00	0.99	0.707	0.049	0.00E+00	Podocyte-4	Serpine2
0.00E+00	0.99	0.645	0.017	0.00E+00	Podocyte-4	Filip1
0.00E+00	0.96	0.628	0.015	0.00E+00	Podocyte-4	Serpini1
0.00E+00	0.95	0.617	0.066	0.00E+00	Podocyte-4	Cspg4
0.00E+00	0.93	0.607	0.001	0.00E+00	Podocyte-4	Kcna5
0.00E+00	0.93	0.603	0.031	0.00E+00	Podocyte-4	Gjc1
0.00E+00	0.93	0.672	0.033	0.00E+00	Podocyte-4	Gucy1a1
0.00E+00	0.93	0.628	0.004	0.00E+00	Podocyte-4	Mrvi1
0.00E+00	0.90	0.624	0.037	0.00E+00	Podocyte-4	Tgfb1i1
8.54E-307	1.74	0.879	0.162	2.64E-302	Podocyte-4	Wtip
3.40E-293	1.56	0.807	0.122	1.05E-288	Podocyte-4	Gng11
8.52E-290	1.04	0.717	0.096	2.64E-285	Podocyte-4	Marveld1
6.27E-285	1.69	0.879	0.167	1.94E-280	Podocyte-4	Mfge8
6.64E-280	1.82	0.866	0.158	2.06E-275	Podocyte-4	Csrp1
1.10E-276	0.82	0.631	0.075	3.41E-272	Podocyte-4	Atp2b4
3.20E-264	1.14	0.662	0.093	9.93E-260	Podocyte-4	Map3k7cl
3.89E-253	0.76	0.603	0.071	1.21E-248	Podocyte-4	Cnn2
4.89E-253	1.00	0.634	0.085	1.51E-248	Podocyte-4	Jag1
1.25E-244	1.30	0.841	0.143	3.88E-240	Podocyte-4	Ifitm3
4.21E-244	1.43	0.862	0.18	1.30E-239	Podocyte-4	Tinagl1
2.28E-239	0.86	0.655	0.097	7.07E-235	Podocyte-4	Arhgef17
1.75E-235	1.59	0.838	0.164	5.41E-231	Podocyte-4	Hspb1
4.09E-232	2.39	0.962	0.316	1.27E-227	Podocyte-4	Mylk
1.36E-224	2.40	0.948	0.321	4.22E-220	Podocyte-4	Tpm1
4.01E-219	1.31	0.872	0.203	1.24E-214	Podocyte-4	Bcam
1.72E-205	1.20	0.803	0.171	5.32E-201	Podocyte-4	Gsn
1.02E-190	1.00	0.7	0.143	3.17E-186	Podocyte-4	Crtc3
1.19E-187	1.77	0.903	0.325	3.70E-183	Podocyte-4	Ppp1r12a
2.57E-186	1.42	0.862	0.233	7.97E-182	Podocyte-4	Epas1
2.20E-184	1.06	0.697	0.141	6.82E-180	Podocyte-4	Utrn
8.56E-173	1.23	0.797	0.214	2.65E-168	Podocyte-4	Rras
4.43E-171	1.01	0.703	0.156	1.37E-166	Podocyte-4	930523C07Ri

2.03E-167	1.35	0.834	0.273	6.28E-163	Podocyte-4	Uba2
1.24E-163	1.08	0.772	0.212	3.84E-159	Podocyte-4	Ppp1r12c
3.24E-161	0.68	0.617	0.106	1.00E-156	Podocyte-4	Esam
7.04E-154	1.00	0.683	0.146	2.18E-149	Podocyte-4	Rgs2
1.53E-151	1.86	0.997	0.936	4.74E-147	Podocyte-4	Myl6
1.85E-147	0.86	0.621	0.138	5.72E-143	Podocyte-4	Ttc28
2.83E-144	1.16	0.776	0.25	8.76E-140	Podocyte-4	Sorbs1
1.79E-142	0.77	0.783	0.194	5.55E-138	Podocyte-4	Sparc
8.42E-139	1.03	0.676	0.155	2.61E-134	Podocyte-4	Cd200
1.18E-127	1.41	0.9	0.514	3.67E-123	Podocyte-4	Rock1
1.40E-127	0.87	0.703	0.2	4.35E-123	Podocyte-4	Pls3
1.95E-123	1.34	0.834	0.362	6.03E-119	Podocyte-4	Palld
5.89E-122	1.06	0.766	0.23	1.83E-117	Podocyte-4	Crip2
1.43E-121	0.83	0.638	0.175	4.41E-117	Podocyte-4	Lgalsl
1.13E-120	1.34	0.9	0.446	3.51E-116	Podocyte-4	Lgals1
1.74E-116	0.82	0.693	0.204	5.38E-112	Podocyte-4	Bnip2
9.87E-112	1.20	0.803	0.333	3.06E-107	Podocyte-4	Pbxip1
1.70E-111	1.33	0.886	0.441	5.25E-107	Podocyte-4	Id1
1.28E-107	1.42	0.959	0.614	3.96E-103	Podocyte-4	Id3
2.50E-106	1.22	0.997	0.964	7.73E-102	Podocyte-4	Actb
2.36E-105	1.49	0.614	0.174	7.30E-101	Podocyte-4	Nr4a1
4.24E-105	1.52	0.955	0.821	1.31E-100	Podocyte-4	Dstn
8.81E-103	0.85	0.676	0.23	2.73E-98	Podocyte-4	Pdgfa
1.75E-101	1.00	0.786	0.364	5.43E-97	Podocyte-4	Crim1
2.25E-101	1.12	0.855	0.455	6.97E-97	Podocyte-4	Selenow
3.45E-101	0.92	0.676	0.231	1.07E-96	Podocyte-4	Mob2
1.53E-99	0.99	0.772	0.293	4.75E-95	Podocyte-4	Rarres2
4.39E-98	1.14	0.928	0.716	1.36E-93	Podocyte-4	Calm2
3.15E-95	1.04	0.945	0.677	9.75E-91	Podocyte-4	Rbpms
4.40E-93	0.73	0.776	0.312	1.36E-88	Podocyte-4	Igfbp5
7.79E-90	0.90	0.693	0.241	2.41E-85	Podocyte-4	Cpe
3.46E-89	0.74	0.676	0.247	1.07E-84	Podocyte-4	Rsu1
1.72E-86	0.85	0.869	0.52	5.32E-82	Podocyte-4	Fermt2
7.23E-86	0.83	0.734	0.333	2.24E-81	Podocyte-4	Rock2
4.19E-85	1.33	0.686	0.248	1.30E-80	Podocyte-4	Klf2
6.93E-85	0.86	0.731	0.298	2.15E-80	Podocyte-4	Col18a1
3.41E-84	0.59	0.7	0.242	1.06E-79	Podocyte-4	Cryab
1.03E-82	0.90	0.817	0.384	3.18E-78	Podocyte-4	Cd9
1.11E-80	0.91	0.841	0.532	3.45E-76	Podocyte-4	Itgb1
3.74E-77	1.44	0.679	0.278	1.16E-72	Podocyte-4	Cdkn1a
3.21E-76	0.96	0.821	0.524	9.94E-72	Podocyte-4	Ppp1cb
1.60E-75	0.72	0.662	0.245	4.95E-71	Podocyte-4	Tbx2
4.96E-73	0.94	0.745	0.398	1.54E-68	Podocyte-4	Septin7
2.36E-72	0.82	0.683	0.304	7.30E-68	Podocyte-4	Sh3bgrl
1.11E-71	0.69	0.697	0.304	3.44E-67	Podocyte-4	Ccdc85b
2.22E-68	0.85	0.852	0.53	6.87E-64	Podocyte-4	Btg1
3.71E-68	0.61	0.983	0.985	1.15E-63	Podocyte-4	Eif1
7.21E-68	0.76	0.7	0.354	2.23E-63	Podocyte-4	Pdcl3
4.59E-67	0.66	0.648	0.273	1.42E-62	Podocyte-4	Cbx6
5.43E-67	0.73	0.934	0.764	1.68E-62	Podocyte-4	Son
1.02E-66	0.63	0.759	0.289	3.16E-62	Podocyte-4	Ckb
2.29E-66	0.73	0.955	0.933	7.08E-62	Podocyte-4	Gnas
2.71E-66	0.93	0.952	0.836	8.40E-62	Podocyte-4	Cst3
7.59E-64	0.77	0.907	0.715	2.35E-59	Podocyte-4	Dynll1
1.28E-63	0.67	0.983	0.946	3.96E-59	Podocyte-4	Rpl21
5.90E-63	0.87	0.776	0.494	1.83E-58	Podocyte-4	Lmna
2.48E-62	0.85	0.79	0.515	7.67E-58	Podocyte-4	Selenom
8.99E-61	0.68	0.714	0.389	2.79E-56	Podocyte-4	Limd1
1.98E-60	0.72	0.845	0.643	6.13E-56	Podocyte-4	Prkar1a
2.53E-59	0.82	0.914	0.769	7.84E-55	Podocyte-4	Tsc22d1
5.41E-59	0.64	0.693	0.346	1.68E-54	Podocyte-4	Thra
1.44E-58	0.68	0.934	0.829	4.46E-54	Podocyte-4	Pfn1
1.87E-58	0.77	0.852	0.639	5.79E-54	Podocyte-4	Gnai2
4.52E-58	0.65	0.607	0.268	1.40E-53	Podocyte-4	Lims1

8.66E-58	0.69	0.941	0.869	2.68E-53	Podocyte-4	Slc25a4
7.88E-57	0.62	0.983	0.97	2.44E-52	Podocyte-4	Ptma
8.72E-57	0.57	0.666	0.314	2.70E-52	Podocyte-4	Serpinh1
2.44E-55	1.33	0.617	0.297	7.56E-51	Podocyte-4	Ppp1r15a
2.29E-53	0.59	0.731	0.367	7.11E-49	Podocyte-4	Iqgap1
2.62E-53	0.57	0.983	0.905	8.13E-49	Podocyte-4	Ddx5
1.53E-52	0.70	0.69	0.394	4.73E-48	Podocyte-4	Arid5b
4.92E-52	0.61	0.79	0.496	1.52E-47	Podocyte-4	Mbnl2
6.46E-52	0.68	0.783	0.519	2.00E-47	Podocyte-4	Lpp
6.61E-51	0.62	0.714	0.417	2.05E-46	Podocyte-4	Ktn1
2.46E-50	0.64	0.645	0.334	7.63E-46	Podocyte-4	Col4a1
3.25E-50	0.52	0.641	0.282	1.01E-45	Podocyte-4	Tacc1
1.31E-49	0.68	0.676	0.317	4.07E-45	Podocyte-4	Hes1
1.67E-49	0.54	0.966	0.933	5.16E-45	Podocyte-4	H3f3b
2.65E-49	0.58	0.752	0.42	8.21E-45	Podocyte-4	Ifitm2
1.97E-48	0.84	0.731	0.445	6.10E-44	Podocyte-4	Slc38a2
3.36E-48	0.59	0.669	0.34	1.04E-43	Podocyte-4	Aopep
6.74E-48	0.63	0.759	0.517	2.09E-43	Podocyte-4	Atp2b1
3.02E-47	0.55	0.862	0.76	9.35E-43	Podocyte-4	Cnbp
4.20E-47	0.66	0.641	0.323	1.30E-42	Podocyte-4	Arpc1b
6.51E-47	0.62	0.869	0.72	2.02E-42	Podocyte-4	Map1lc3a
2.66E-46	0.61	0.834	0.646	8.23E-42	Podocyte-4	Ywhaq
3.33E-46	0.59	0.741	0.494	1.03E-41	Podocyte-4	Rap1a
4.38E-46	0.66	0.714	0.464	1.36E-41	Podocyte-4	Tln1
1.56E-45	0.59	0.821	0.669	4.82E-41	Podocyte-4	Nedd4
1.58E-44	0.58	0.717	0.44	4.90E-40	Podocyte-4	Nptn
4.39E-44	0.55	0.862	0.738	1.36E-39	Podocyte-4	Laptm4a
5.16E-44	0.66	0.603	0.315	1.60E-39	Podocyte-4	Zyx
3.81E-41	0.57	0.697	0.458	1.18E-36	Podocyte-4	Gna11
5.10E-41	1.26	0.769	0.552	1.58E-36	Podocyte-4	Junb
6.19E-41	0.56	0.69	0.417	1.92E-36	Podocyte-4	Blmh
1.46E-40	0.56	0.652	0.371	4.51E-36	Podocyte-4	Anxa11
2.94E-40	0.58	0.876	0.702	9.11E-36	Podocyte-4	Atp2a2
1.33E-39	0.59	0.659	0.415	4.13E-35	Podocyte-4	Mprip
2.52E-39	0.56	0.772	0.586	7.80E-35	Podocyte-4	Nenf
2.78E-37	0.66	0.876	0.812	8.61E-33	Podocyte-4	Calm1
7.04E-37	0.82	0.9	0.853	2.18E-32	Podocyte-4	Jund
1.55E-36	0.55	0.679	0.438	4.81E-32	Podocyte-4	Macf1
1.95E-36	0.51	0.821	0.678	6.04E-32	Podocyte-4	Kif5b
3.12E-36	0.60	0.69	0.414	9.67E-32	Podocyte-4	Rhob
3.50E-36	0.52	0.669	0.439	1.09E-31	Podocyte-4	230219D22Ri
5.63E-36	0.56	0.659	0.403	1.74E-31	Podocyte-4	Nbeal1
3.59E-35	0.58	0.714	0.486	1.11E-30	Podocyte-4	Cirbp
9.25E-35	0.59	0.69	0.469	2.87E-30	Podocyte-4	Nr2f2
1.11E-34	0.51	0.779	0.662	3.43E-30	Podocyte-4	Actn4
2.97E-34	0.56	0.669	0.439	9.21E-30	Podocyte-4	Itga1
9.75E-34	1.13	0.766	0.575	3.02E-29	Podocyte-4	Cebpb
1.44E-33	1.01	0.79	0.586	4.47E-29	Podocyte-4	Fos
8.52E-33	0.52	0.707	0.524	2.64E-28	Podocyte-4	Tprgl
1.14E-31	0.52	0.621	0.382	3.53E-27	Podocyte-4	Slmap
1.78E-31	0.51	0.717	0.541	5.51E-27	Podocyte-4	Gnb1
2.78E-29	0.72	0.741	0.483	8.60E-25	Podocyte-4	Btg2
1.85E-28	0.76	0.666	0.493	5.73E-24	Podocyte-4	Ccnl1
2.05E-28	0.56	0.666	0.454	6.34E-24	Podocyte-4	Tra2a
2.83E-28	0.53	0.721	0.579	8.76E-24	Podocyte-4	Purb
3.97E-28	0.85	0.628	0.434	1.23E-23	Podocyte-4	Ier2
1.55E-27	0.56	0.755	0.64	4.81E-23	Podocyte-4	Drap1
5.88E-26	0.53	0.603	0.37	1.82E-21	Podocyte-4	Kcnq1ot1
7.79E-24	0.98	0.652	0.487	2.41E-19	Podocyte-4	Egr1
1.41E-23	0.52	0.648	0.501	4.38E-19	Podocyte-4	Clic4
9.99E-23	0.72	0.628	0.491	3.09E-18	Podocyte-4	Tob2
7.01E-22	0.57	0.693	0.524	2.17E-17	Podocyte-4	Nfkbia
4.12E-19	0.52	0.676	0.557	1.28E-14	Podocyte-4	Tagln2
0.00E+00	1.67	0.836	0.084	0.00E+00	Podocyte-5	Cald1

0.00E+00	1.65	0.804	0.031	0.00E+00	Podocyte-5	Cp
0.00E+00	1.46	0.644	0.034	0.00E+00	Podocyte-5	Akap12
0.00E+00	1.31	0.764	0.087	0.00E+00	Podocyte-5	Timp2
1.14E-307	0.98	0.655	0.066	3.54E-303	Podocyte-5	Tshz2
7.68E-208	2.53	0.876	0.24	2.38E-203	Podocyte-5	Cryab
3.33E-204	1.51	0.924	0.234	1.03E-199	Podocyte-5	Tmsb10
7.59E-202	1.27	0.68	0.112	2.35E-197	Podocyte-5	Nupr1
9.05E-197	0.84	0.636	0.098	2.80E-192	Podocyte-5	Scin
9.28E-197	1.00	0.618	0.096	2.88E-192	Podocyte-5	Thbs1
2.67E-164	0.94	0.724	0.157	8.28E-160	Podocyte-5	930523C07Ri
2.59E-163	1.00	0.825	0.204	8.03E-159	Podocyte-5	Bcam
4.14E-163	0.85	0.625	0.117	1.28E-158	Podocyte-5	Actn1
1.17E-150	0.62	0.633	0.12	3.62E-146	Podocyte-5	Capg
1.16E-142	0.87	0.756	0.173	3.61E-138	Podocyte-5	Anxa2
5.61E-140	1.16	0.716	0.166	1.74E-135	Podocyte-5	Hspb1
1.29E-138	1.24	0.72	0.182	4.00E-134	Podocyte-5	Tinagl1
3.25E-131	1.38	0.938	0.514	1.01E-126	Podocyte-5	S100a11
7.03E-122	1.07	0.833	0.323	2.18E-117	Podocyte-5	Tpm1
9.70E-119	0.65	0.647	0.148	3.00E-114	Podocyte-5	Klf7
2.42E-113	0.84	0.676	0.173	7.50E-109	Podocyte-5	Gsn
7.33E-113	0.89	0.756	0.247	2.27E-108	Podocyte-5	Litaf
7.82E-112	1.01	0.789	0.273	2.42E-107	Podocyte-5	Tns3
1.35E-107	0.74	0.669	0.203	4.17E-103	Podocyte-5	Ptpn13
5.03E-107	1.14	0.767	0.298	1.56E-102	Podocyte-5	Col18a1
7.73E-105	0.88	0.844	0.34	2.39E-100	Podocyte-5	Ifi27
1.39E-104	1.08	0.811	0.332	4.31E-100	Podocyte-5	Col4a1
2.99E-103	1.35	0.713	0.244	9.28E-99	Podocyte-5	Adamts1
6.72E-103	1.14	1	0.932	2.08E-98	Podocyte-5	H3f3b
2.91E-102	1.14	0.695	0.212	9.00E-98	Podocyte-5	Sox4
3.18E-100	1.03	0.833	0.386	9.85E-96	Podocyte-5	Dag1
1.12E-98	1.01	0.942	0.649	3.46E-94	Podocyte-5	Myl12a
2.70E-98	0.75	0.615	0.174	8.37E-94	Podocyte-5	Hoxb9
1.43E-97	0.86	0.996	0.97	4.43E-93	Podocyte-5	Ptma
6.79E-96	1.39	0.996	0.922	2.10E-91	Podocyte-5	Igfbp7
1.00E-93	0.77	0.996	0.92	3.11E-89	Podocyte-5	H3f3a
2.16E-91	0.67	0.745	0.264	6.69E-87	Podocyte-5	Serpinb6a
1.29E-88	0.79	0.662	0.191	3.98E-84	Podocyte-5	Lgals3
1.06E-86	0.78	0.676	0.207	3.29E-82	Podocyte-5	Tspan8
1.33E-86	0.94	0.644	0.196	4.11E-82	Podocyte-5	Sparc
5.96E-86	0.73	0.673	0.233	1.85E-81	Podocyte-5	Vcl
3.27E-85	0.76	0.618	0.167	1.01E-80	Podocyte-5	Kcnk1
3.63E-85	0.94	0.84	0.429	1.12E-80	Podocyte-5	Anxa5
3.34E-84	1.24	0.895	0.666	1.03E-79	Podocyte-5	Slc6a6
2.94E-83	1.21	0.618	0.17	9.12E-79	Podocyte-5	Mfge8
3.04E-83	0.69	0.618	0.201	9.43E-79	Podocyte-5	Mid1ip1
6.21E-83	0.87	0.909	0.532	1.92E-78	Podocyte-5	Itgb1
1.53E-82	1.15	0.825	0.562	4.75E-78	Podocyte-5	Bicc1
2.43E-82	1.28	0.662	0.219	7.52E-78	Podocyte-5	Epcam
3.90E-82	0.55	0.618	0.182	1.21E-77	Podocyte-5	Sema5a
5.16E-82	0.66	0.771	0.299	1.60E-77	Podocyte-5	Itm2c
8.53E-80	0.64	0.698	0.235	2.64E-75	Podocyte-5	Cd82
5.33E-79	0.66	0.996	0.936	1.65E-74	Podocyte-5	Myl6
9.10E-79	0.69	1	0.946	2.82E-74	Podocyte-5	Rpl21
5.91E-78	0.86	0.942	0.715	1.83E-73	Podocyte-5	Dynll1
3.62E-77	0.58	0.993	0.972	1.12E-72	Podocyte-5	Rps16
3.22E-76	0.77	0.633	0.231	9.98E-72	Podocyte-5	Pdgfa
1.26E-74	0.91	0.727	0.298	3.91E-70	Podocyte-5	Ier5
1.31E-74	0.67	0.793	0.358	4.07E-70	Podocyte-5	Msn
6.23E-73	1.11	0.913	0.615	1.93E-68	Podocyte-5	Id3
1.04E-72	0.88	0.993	0.965	3.22E-68	Podocyte-5	Actb
1.50E-72	0.51	0.611	0.212	4.65E-68	Podocyte-5	Smtn
2.90E-72	0.83	0.72	0.338	9.00E-68	Podocyte-5	Pax8
3.98E-72	1.23	0.949	0.763	1.23E-67	Podocyte-5	Gstm1
5.95E-70	0.73	0.687	0.28	1.84E-65	Podocyte-5	Bex3

6.50E-70	1.14	0.92	0.759	2.01E-65	Podocyte-5	S100a10
1.86E-69	0.59	0.705	0.288	5.75E-65	Podocyte-5	Anxa4
3.41E-69	0.70	0.935	0.625	1.06E-64	Podocyte-5	H2-D1
2.11E-68	0.62	0.705	0.277	6.53E-64	Podocyte-5	Tpm4
4.94E-68	0.68	0.651	0.248	1.53E-63	Podocyte-5	Smchd1
6.71E-68	0.91	0.924	0.696	2.08E-63	Podocyte-5	H2-K1
6.55E-67	0.52	1	0.99	2.03E-62	Podocyte-5	Itm2b
8.93E-67	1.01	0.658	0.271	2.77E-62	Podocyte-5	Ahnak
1.93E-66	1.06	0.935	0.853	5.97E-62	Podocyte-5	Jund
6.69E-66	0.62	0.935	0.638	2.07E-61	Podocyte-5	Gnai2
1.07E-65	0.61	0.993	0.915	3.30E-61	Podocyte-5	Rpl3
4.44E-65	0.70	0.924	0.678	1.38E-60	Podocyte-5	Rbpms
3.59E-64	0.68	0.676	0.238	1.11E-59	Podocyte-5	Gadd45b
3.74E-63	0.64	0.742	0.35	1.16E-58	Podocyte-5	Nfat5
3.80E-63	0.66	0.738	0.346	1.18E-58	Podocyte-5	Thra
4.62E-62	0.76	0.767	0.438	1.43E-57	Podocyte-5	Itga1
4.96E-62	0.60	0.964	0.811	1.54E-57	Podocyte-5	Calm1
1.23E-61	1.52	0.687	0.327	3.80E-57	Podocyte-5	Atf3
1.79E-61	0.78	0.782	0.413	5.56E-57	Podocyte-5	Tceal9
1.95E-61	0.51	0.993	0.964	6.05E-57	Podocyte-5	Rpl9
3.30E-61	0.53	0.996	0.973	1.02E-56	Podocyte-5	Rps3a1
4.69E-61	0.70	0.873	0.626	1.45E-56	Podocyte-5	810037I17Ri
1.28E-60	0.62	0.873	0.455	3.97E-56	Podocyte-5	Selenow
3.77E-60	1.12	0.782	0.494	1.17E-55	Podocyte-5	Lmna
6.86E-60	0.62	0.935	0.737	2.13E-55	Podocyte-5	Laptm4a
7.56E-60	0.66	0.775	0.413	2.34E-55	Podocyte-5	Myh9
2.73E-59	0.60	0.935	0.749	8.45E-55	Podocyte-5	Sumo2
2.84E-58	0.76	0.815	0.542	8.81E-54	Podocyte-5	Rtn4
4.68E-58	1.19	0.836	0.482	1.45E-53	Podocyte-5	Btg2
9.83E-58	0.62	0.731	0.353	3.05E-53	Podocyte-5	Atp1b3
1.41E-57	0.62	0.891	0.722	4.36E-53	Podocyte-5	Hmgn1
1.95E-57	0.65	0.615	0.262	6.03E-53	Podocyte-5	Spns2
2.87E-56	0.71	0.804	0.499	8.88E-52	Podocyte-5	Clic4
4.35E-56	0.64	0.625	0.263	1.35E-51	Podocyte-5	Col4a2
7.73E-56	0.58	0.964	0.862	2.39E-51	Podocyte-5	App
8.08E-56	0.78	0.84	0.676	2.50E-51	Podocyte-5	Spint2
9.50E-56	0.88	0.924	0.769	2.94E-51	Podocyte-5	Tsc22d1
1.40E-54	0.93	0.698	0.355	4.35E-50	Podocyte-5	Npnt
3.70E-54	0.68	0.844	0.591	1.15E-49	Podocyte-5	Clic1
4.25E-54	0.60	0.895	0.645	1.32E-49	Podocyte-5	Ywhaq
5.11E-54	0.58	0.862	0.585	1.58E-49	Podocyte-5	Nenf
7.69E-54	0.70	0.76	0.46	2.38E-49	Podocyte-5	Syne2
2.75E-53	0.54	0.847	0.54	8.51E-49	Podocyte-5	Gnb1
3.96E-53	0.57	0.873	0.52	1.23E-48	Podocyte-5	Fermt2
4.24E-53	0.53	0.749	0.366	1.31E-48	Podocyte-5	Rbms1
6.07E-53	0.61	0.709	0.334	1.88E-48	Podocyte-5	Rock2
6.68E-53	0.74	0.996	0.963	2.07E-48	Podocyte-5	Actg1
6.88E-53	1.03	0.847	0.551	2.13E-48	Podocyte-5	Junb
1.84E-51	0.61	0.775	0.398	5.69E-47	Podocyte-5	Septin7
2.38E-51	0.68	0.738	0.399	7.36E-47	Podocyte-5	Ctdspl
3.03E-51	1.12	0.665	0.356	9.38E-47	Podocyte-5	Ifrd1
3.91E-51	0.52	0.625	0.252	1.21E-46	Podocyte-5	Klf4
5.10E-51	0.56	0.753	0.402	1.58E-46	Podocyte-5	Nbeal1
5.83E-51	0.60	0.887	0.669	1.81E-46	Podocyte-5	Nedd4
9.97E-51	0.56	0.938	0.798	3.09E-46	Podocyte-5	Cfl1
4.74E-50	1.13	0.778	0.486	1.47E-45	Podocyte-5	Egr1
5.06E-50	0.67	0.665	0.346	1.57E-45	Podocyte-5	Midn
3.27E-49	1.13	0.804	0.558	1.01E-44	Podocyte-5	Klf6
1.28E-48	0.62	0.695	0.365	3.97E-44	Podocyte-5	Crim1
1.60E-48	0.57	0.833	0.531	4.95E-44	Podocyte-5	Btg1
9.30E-48	0.82	0.887	0.812	2.88E-43	Podocyte-5	Sdc4
1.82E-47	0.77	0.807	0.556	5.64E-43	Podocyte-5	Tagln2
4.61E-47	1.37	0.658	0.379	1.43E-42	Podocyte-5	Fosb
5.12E-47	0.50	0.724	0.317	1.59E-42	Podocyte-5	Hes1

7.05E-47	0.63	0.76	0.413	2.18E-42	Podocyte-5	Rhob
1.82E-46	0.64	0.655	0.297	5.63E-42	Podocyte-5	Ppp1r15a
6.05E-46	0.52	0.811	0.496	1.87E-41	Podocyte-5	Mbnl2
6.33E-46	0.77	0.876	0.691	1.96E-41	Podocyte-5	Zfp36l1
1.20E-45	0.84	0.804	0.575	3.72E-41	Podocyte-5	Cebpb
2.47E-45	1.55	0.891	0.738	7.64E-41	Podocyte-5	Spp1
3.60E-45	0.51	0.764	0.439	1.12E-40	Podocyte-5	230219D22Ri
9.67E-45	0.51	0.793	0.439	2.99E-40	Podocyte-5	Tmem50a
3.66E-44	0.72	0.84	0.53	1.13E-39	Podocyte-5	Tmsb4x
4.33E-44	0.56	0.742	0.446	1.34E-39	Podocyte-5	Tpm3
2.97E-43	0.50	0.982	0.905	9.21E-39	Podocyte-5	Ddx5
3.21E-43	0.51	0.96	0.807	9.94E-39	Podocyte-5	Rabac1
3.71E-43	0.54	0.913	0.669	1.15E-38	Podocyte-5	B2m
7.35E-43	1.59	0.658	0.411	2.28E-38	Podocyte-5	Hbegf
2.89E-42	0.50	0.938	0.764	8.95E-38	Podocyte-5	Son
6.35E-42	0.50	0.691	0.369	1.97E-37	Podocyte-5	Eid1
6.35E-42	0.68	0.673	0.372	1.97E-37	Podocyte-5	Lmo7
2.94E-41	0.55	0.778	0.444	9.10E-37	Podocyte-5	Slc38a2
8.90E-41	0.78	0.753	0.442	2.76E-36	Podocyte-5	Id1
3.87E-40	0.51	0.793	0.53	1.20E-35	Podocyte-5	Hnrnpa1
1.19E-39	0.50	0.662	0.375	3.69E-35	Podocyte-5	Chd9
1.35E-39	0.54	0.658	0.356	4.17E-35	Podocyte-5	Lamb1
1.97E-39	0.69	0.785	0.523	6.12E-35	Podocyte-5	Nfkbia
4.40E-38	0.80	0.753	0.468	1.36E-33	Podocyte-5	Nr2f2
9.69E-38	0.56	0.655	0.378	3.00E-33	Podocyte-5	Eif1a
2.46E-37	0.55	0.764	0.52	7.61E-33	Podocyte-5	Adgrg1
3.47E-37	0.96	0.702	0.415	1.08E-32	Podocyte-5	Ier3
1.39E-36	0.63	0.615	0.32	4.30E-32	Podocyte-5	Mylk
2.44E-36	0.59	0.618	0.328	7.56E-32	Podocyte-5	Kdm6b
7.21E-36	0.51	0.669	0.372	2.23E-31	Podocyte-5	Ankrd12
8.89E-36	0.62	0.851	0.722	2.76E-31	Podocyte-5	Pnrc1
2.43E-35	0.50	0.825	0.637	7.52E-31	Podocyte-5	Mcl1
1.04E-34	1.01	0.785	0.586	3.23E-30	Podocyte-5	Fos
1.16E-34	0.52	0.749	0.426	3.59E-30	Podocyte-5	Mxd4
2.37E-33	0.73	0.782	0.585	7.34E-29	Podocyte-5	Wwc1
5.93E-33	0.89	0.756	0.641	1.84E-28	Podocyte-5	Cstb
3.39E-32	0.50	0.655	0.37	1.05E-27	Podocyte-5	Kcnq1ot1
3.41E-31	0.55	0.793	0.584	1.06E-26	Podocyte-5	Zfand5
2.83E-29	0.63	0.942	0.886	8.75E-25	Podocyte-5	Tmem176b
1.22E-26	0.53	0.625	0.312	3.78E-22	Podocyte-5	Cd24a
1.37E-26	0.69	0.669	0.435	4.24E-22	Podocyte-5	Ccn1
1.70E-26	0.54	0.76	0.521	5.27E-22	Podocyte-5	Cyb5r3
8.40E-25	1.25	0.665	0.529	2.60E-20	Podocyte-5	Krt8
2.57E-22	0.68	0.953	0.901	7.96E-18	Podocyte-5	Ndufb8
5.70E-20	0.71	0.684	0.539	1.76E-15	Podocyte-5	Chka
2.68E-18	0.69	0.709	0.59	8.31E-14	Podocyte-5	Zfp36
6.95E-18	0.67	0.647	0.467	2.15E-13	Podocyte-5	H1f2
6.07E-14	0.54	0.607	0.449	1.88E-09	Podocyte-5	Lgals1
8.03E-12	0.51	0.633	0.378	2.49E-07	Podocyte-5	Wfdc2
1.21E-274	0.62	0.755	0.083	3.75E-270	Podocyte-6	Kdr
1.32E-178	0.57	0.678	0.103	4.09E-174	Podocyte-6	Ehd3
3.14E-169	0.51	0.727	0.117	9.73E-165	Podocyte-6	Meis2
4.19E-156	0.66	0.796	0.167	1.30E-151	Podocyte-6	Plat
0.00E+00	4.56	0.993	0.183	0.00E+00	Macrophage	Cd74
0.00E+00	4.25	0.788	0.025	0.00E+00	Macrophage	Ccl4
0.00E+00	4.17	0.987	0.076	0.00E+00	Macrophage	H2-Eb1
0.00E+00	4.05	0.986	0.076	0.00E+00	Macrophage	H2-Aa
0.00E+00	3.82	0.983	0.154	0.00E+00	Macrophage	H2-Ab1
0.00E+00	3.65	0.793	0.013	0.00E+00	Macrophage	Ccl3
0.00E+00	3.48	0.957	0.007	0.00E+00	Macrophage	C1qa
0.00E+00	3.48	0.968	0.01	0.00E+00	Macrophage	C1qb
0.00E+00	3.17	0.61	0.028	0.00E+00	Macrophage	Cxcl2
0.00E+00	3.17	0.818	0.023	0.00E+00	Macrophage	Il1b
0.00E+00	2.97	0.935	0.005	0.00E+00	Macrophage	C1qc

0.00E+00	2.93	0.964	0.023	0.00E+00	Macrophage	Ctss
0.00E+00	2.76	0.973	0.031	0.00E+00	Macrophage	Fcer1g
0.00E+00	2.73	0.93	0.028	0.00E+00	Macrophage	Lyz2
0.00E+00	2.59	0.783	0.013	0.00E+00	Macrophage	Rgs1
0.00E+00	2.49	0.966	0.024	0.00E+00	Macrophage	Tyrobp
0.00E+00	2.48	0.871	0.041	0.00E+00	Macrophage	Cd14
0.00E+00	2.43	0.792	0.026	0.00E+00	Macrophage	Cd83
0.00E+00	2.33	0.958	0.032	0.00E+00	Macrophage	Cd52
0.00E+00	2.12	0.999	0.521	0.00E+00	Macrophage	Tmsb4x
0.00E+00	2.11	0.689	0.013	0.00E+00	Macrophage	Tnf
0.00E+00	2.06	0.993	0.833	0.00E+00	Macrophage	Cst3
0.00E+00	2.01	0.877	0.121	0.00E+00	Macrophage	H2-DMb1
0.00E+00	1.96	0.881	0.316	0.00E+00	Macrophage	Atf3
0.00E+00	1.95	0.791	0.173	0.00E+00	Macrophage	Mafb
0.00E+00	1.91	0.739	0.013	0.00E+00	Macrophage	Bcl2a1b
0.00E+00	1.90	0.854	0.009	0.00E+00	Macrophage	Fcgr3
0.00E+00	1.88	0.885	0.132	0.00E+00	Macrophage	Ctsc
0.00E+00	1.87	0.858	0.288	0.00E+00	Macrophage	Ier5
0.00E+00	1.86	0.89	0.025	0.00E+00	Macrophage	Laptm5
0.00E+00	1.83	0.713	0.002	0.00E+00	Macrophage	Ms4a7
0.00E+00	1.81	0.808	0.019	0.00E+00	Macrophage	Fyb
0.00E+00	1.80	0.831	0.028	0.00E+00	Macrophage	Rgs10
0.00E+00	1.78	0.855	0.061	0.00E+00	Macrophage	H2-DMa
0.00E+00	1.77	0.9	0.096	0.00E+00	Macrophage	Fxyd5
0.00E+00	1.77	0.937	0.515	0.00E+00	Macrophage	Nfkbia
0.00E+00	1.74	0.868	0.09	0.00E+00	Macrophage	Unc93b1
0.00E+00	1.74	0.74	0.169	0.00E+00	Macrophage	Pim1
0.00E+00	1.72	0.976	0.619	0.00E+00	Macrophage	H2-D1
0.00E+00	1.69	0.699	0.011	0.00E+00	Macrophage	Basp1
0.00E+00	1.68	0.7	0.012	0.00E+00	Macrophage	Wfdc17
0.00E+00	1.66	0.803	0.096	0.00E+00	Macrophage	Trf
0.00E+00	1.64	0.73	0.034	0.00E+00	Macrophage	Ifi27l2a
0.00E+00	1.64	0.801	0.045	0.00E+00	Macrophage	Csf1r
0.00E+00	1.64	0.855	0.217	0.00E+00	Macrophage	Cxcl16
0.00E+00	1.60	0.892	0.119	0.00E+00	Macrophage	Srgn
0.00E+00	1.60	0.782	0.01	0.00E+00	Macrophage	Cd300c2
0.00E+00	1.59	0.786	0.014	0.00E+00	Macrophage	Cybb
0.00E+00	1.54	0.815	0.026	0.00E+00	Macrophage	Coro1a
0.00E+00	1.50	0.719	0.007	0.00E+00	Macrophage	Slamf9
0.00E+00	1.50	0.735	0.011	0.00E+00	Macrophage	Ly86
0.00E+00	1.47	0.813	0.296	0.00E+00	Macrophage	Nfkbiz
0.00E+00	1.46	0.617	0.02	0.00E+00	Macrophage	Stap1
0.00E+00	1.44	0.66	0.023	0.00E+00	Macrophage	Ifi207
0.00E+00	1.44	0.723	0.024	0.00E+00	Macrophage	Alox5ap
0.00E+00	1.44	0.66	0.009	0.00E+00	Macrophage	Lilr4b
0.00E+00	1.42	0.638	0.006	0.00E+00	Macrophage	Gm34084
0.00E+00	1.42	0.68	0.012	0.00E+00	Macrophage	Pou2f2
0.00E+00	1.40	0.689	0.015	0.00E+00	Macrophage	Tgfbi
0.00E+00	1.38	0.765	0.033	0.00E+00	Macrophage	Ptprc
0.00E+00	1.38	0.773	0.164	0.00E+00	Macrophage	Fcgr2b
0.00E+00	1.38	0.663	0.012	0.00E+00	Macrophage	Cd86
0.00E+00	1.36	0.704	0.026	0.00E+00	Macrophage	Cd68
0.00E+00	1.36	0.753	0.074	0.00E+00	Macrophage	Lcp1
0.00E+00	1.35	0.644	0.036	0.00E+00	Macrophage	Neurl3
0.00E+00	1.34	0.676	0.002	0.00E+00	Macrophage	C3ar1
0.00E+00	1.33	0.775	0.128	0.00E+00	Macrophage	Ptpn18
0.00E+00	1.32	0.68	0.013	0.00E+00	Macrophage	Pld4
0.00E+00	1.30	0.646	0.006	0.00E+00	Macrophage	Aif1
0.00E+00	1.29	0.677	0.017	0.00E+00	Macrophage	Lst1
0.00E+00	1.27	0.71	0.182	0.00E+00	Macrophage	Lgals3
0.00E+00	1.26	0.683	0.013	0.00E+00	Macrophage	Mpeg1
0.00E+00	1.25	0.666	0.004	0.00E+00	Macrophage	Adgre1
0.00E+00	1.23	0.633	0.07	0.00E+00	Macrophage	Rel
0.00E+00	1.23	0.677	0.055	0.00E+00	Macrophage	Marcks

0.00E+00	1.22	0.74	0.181	0.00E+00	Macrophage	Efhd2
0.00E+00	1.21	0.677	0.03	0.00E+00	Macrophage	Tm6sf1
0.00E+00	1.21	0.611	0.007	0.00E+00	Macrophage	Slamf7
0.00E+00	1.21	0.651	0.015	0.00E+00	Macrophage	Spi1
0.00E+00	1.20	0.661	0.089	0.00E+00	Macrophage	Stk17b
0.00E+00	1.12	1	0.985	0.00E+00	Macrophage	Fau
0.00E+00	1.08	0.61	0.02	0.00E+00	Macrophage	Ncf2
0.00E+00	1.03	0.67	0.101	0.00E+00	Macrophage	Psmb8
0.00E+00	0.99	0.617	0.09	0.00E+00	Macrophage	Tgfb1
0.00E+00	0.97	0.61	0.049	0.00E+00	Macrophage	Arhgdib
0.00E+00	0.95	0.608	0.124	0.00E+00	Macrophage	Fam49b
5.18E-308	1.58	0.971	0.691	1.61E-303	Macrophage	H2-K1
8.69E-307	1.20	0.999	0.966	2.69E-302	Macrophage	Rps9
1.05E-300	0.98	0.615	0.152	3.24E-296	Macrophage	Irf8
1.14E-299	1.24	0.709	0.203	3.52E-295	Macrophage	Kctd12
6.43E-298	1.34	0.974	0.663	1.99E-293	Macrophage	B2m
1.11E-292	1.05	1	0.979	3.44E-288	Macrophage	Rps24
1.20E-288	1.30	0.945	0.662	3.71E-284	Macrophage	Npc2
1.50E-282	1.31	0.927	0.572	4.65E-278	Macrophage	Ctsh
7.72E-281	1.25	0.923	0.455	2.39E-276	Macrophage	Ucp2
2.48E-279	1.12	0.994	0.921	7.69E-275	Macrophage	Psap
7.34E-279	1.36	0.887	0.479	2.27E-274	Macrophage	Hexb
6.54E-278	1.12	0.696	0.229	2.03E-273	Macrophage	Tgfbr1
3.93E-275	1.82	0.94	0.609	1.22E-270	Macrophage	Dusp1
4.80E-273	1.60	0.981	0.932	1.49E-268	Macrophage	H3f3b
4.55E-270	1.14	0.914	0.413	1.41E-265	Macrophage	Sh3bgrl3
2.02E-269	1.06	0.996	0.956	6.27E-265	Macrophage	Rps4x
4.17E-265	0.93	0.994	0.974	1.29E-260	Macrophage	Rps11
1.37E-261	1.24	0.722	0.266	4.26E-257	Macrophage	Skil
2.61E-259	1.06	0.802	0.313	8.07E-255	Macrophage	Arpc1b
2.27E-258	0.96	0.994	0.964	7.03E-254	Macrophage	Rpl18a
3.44E-258	1.64	0.918	0.544	1.07E-253	Macrophage	Junb
1.05E-257	1.30	0.773	0.319	3.25E-253	Macrophage	Kdm6b
3.06E-251	0.90	0.996	0.974	9.48E-247	Macrophage	Rps12
6.06E-250	0.93	0.999	0.963	1.88E-245	Macrophage	Rps5
2.05E-249	1.57	0.737	0.24	6.35E-245	Macrophage	Klf2
7.83E-244	0.84	0.997	0.977	2.42E-239	Macrophage	Rpl32
1.98E-242	0.79	1	0.988	6.15E-238	Macrophage	Rplp1
8.87E-239	1.21	0.928	0.658	2.75E-234	Macrophage	410006H16Ri
2.57E-238	1.15	0.904	0.583	7.95E-234	Macrophage	Ctsz
2.74E-237	0.90	0.996	0.959	8.48E-233	Macrophage	Rpl10
1.05E-235	0.90	0.989	0.946	3.27E-231	Macrophage	Rps13
1.36E-235	1.05	1	0.964	4.23E-231	Macrophage	Actb
2.00E-232	0.94	0.994	0.949	6.19E-228	Macrophage	Rpsa
2.43E-232	0.98	0.983	0.895	7.52E-228	Macrophage	Rpl12
5.00E-229	1.24	0.746	0.289	1.55E-224	Macrophage	Ppp1r15a
1.76E-226	1.33	0.9	0.632	5.46E-222	Macrophage	Mcl1
1.86E-224	1.18	0.733	0.294	5.77E-220	Macrophage	Tsc22d3
1.08E-222	0.76	0.999	0.972	3.35E-218	Macrophage	Rps16
1.30E-222	0.81	0.999	0.97	4.01E-218	Macrophage	Rpl39
1.73E-221	1.19	0.925	0.474	5.36E-217	Macrophage	Btg2
8.93E-221	0.95	0.664	0.242	2.77E-216	Macrophage	Hexa
1.96E-219	1.27	0.917	0.551	6.07E-215	Macrophage	Klf6
3.09E-218	2.76	0.862	0.582	9.57E-214	Macrophage	Apoe
7.55E-215	0.73	1	0.99	2.34E-210	Macrophage	Rps29
3.77E-210	0.83	0.991	0.943	1.17E-205	Macrophage	Rpl34
2.23E-205	1.13	0.891	0.524	6.89E-201	Macrophage	Btg1
1.37E-204	0.85	0.951	0.684	4.26E-200	Macrophage	Serinc3
1.37E-200	0.74	0.999	0.976	4.24E-196	Macrophage	Rps14
8.87E-200	1.14	0.98	0.85	2.75E-195	Macrophage	Jund
2.07E-199	0.76	0.996	0.975	6.41E-195	Macrophage	Rps27a
3.05E-194	0.96	0.681	0.286	9.45E-190	Macrophage	Zeb2
1.14E-191	0.65	1	0.984	3.53E-187	Macrophage	Rpl37a
1.35E-189	1.20	0.881	0.584	4.18E-185	Macrophage	Zfp36

1.91E-188	0.62	1	0.989	5.92E-184	Macrophage	Rpl23
1.98E-188	1.31	0.726	0.349	6.14E-184	Macrophage	Ifrd1
9.29E-188	1.11	0.732	0.357	2.88E-183	Macrophage	Dleu2
1.47E-186	0.65	1	0.976	4.57E-182	Macrophage	Rps27
3.59E-185	0.70	0.996	0.961	1.11E-180	Macrophage	Rplp2
4.30E-182	0.72	0.993	0.956	1.33E-177	Macrophage	Rpl13
5.30E-182	1.00	0.957	0.845	1.64E-177	Macrophage	Ctsb
1.07E-176	0.65	0.994	0.973	3.31E-172	Macrophage	Rpl19
7.46E-176	0.84	0.656	0.271	2.31E-171	Macrophage	Ifngr1
1.01E-175	0.65	0.993	0.965	3.14E-171	Macrophage	Rps18
1.42E-174	0.68	0.991	0.952	4.40E-170	Macrophage	Rps15a
4.14E-174	0.98	0.749	0.36	1.28E-169	Macrophage	Iqgap1
1.52E-172	0.91	0.862	0.586	4.71E-168	Macrophage	Clic1
2.95E-171	0.82	0.974	0.915	9.14E-167	Macrophage	Rpl3
2.66E-170	0.70	0.993	0.945	8.25E-166	Macrophage	Rpl17
9.82E-170	1.12	0.862	0.479	3.04E-165	Macrophage	Egr1
4.26E-166	0.66	0.99	0.957	1.32E-161	Macrophage	Rps3
2.52E-164	0.77	0.892	0.634	7.81E-160	Macrophage	Gnai2
3.13E-164	0.94	0.765	0.472	9.69E-160	Macrophage	Snx5
3.11E-163	0.69	0.994	0.946	9.64E-159	Macrophage	Rpl21
2.01E-161	0.62	0.994	0.972	6.22E-157	Macrophage	Rpl11
3.35E-161	0.61	0.999	0.984	1.04E-156	Macrophage	Eif1
4.19E-160	0.90	0.614	0.256	1.30E-155	Macrophage	Jpt1
2.74E-158	0.88	0.648	0.245	8.50E-154	Macrophage	Klf4
4.79E-156	0.62	0.986	0.954	1.48E-151	Macrophage	Rps23
7.37E-155	0.62	0.996	0.975	2.28E-150	Macrophage	Rps20
1.11E-152	0.68	0.978	0.933	3.45E-148	Macrophage	Rack1
3.14E-152	0.96	0.911	0.783	9.72E-148	Macrophage	H2az1
3.82E-151	0.60	0.997	0.963	1.18E-146	Macrophage	Rpl9
4.65E-149	0.57	0.996	0.976	1.44E-144	Macrophage	Rpl26
4.14E-148	0.59	0.996	0.972	1.28E-143	Macrophage	Rps3a1
1.60E-147	0.69	0.968	0.901	4.94E-143	Macrophage	Rpl18
1.33E-146	1.05	0.712	0.423	4.12E-142	Macrophage	Ifi30
1.10E-144	0.70	0.943	0.856	3.42E-140	Macrophage	Rpl27a
2.54E-143	0.55	0.996	0.985	7.86E-139	Macrophage	Rps21
4.69E-140	0.75	0.967	0.904	1.45E-135	Macrophage	Ddx5
1.78E-139	0.79	0.618	0.232	5.50E-135	Macrophage	Gadd45b
2.34E-136	0.56	0.607	0.231	7.25E-132	Macrophage	Tmsb10
9.46E-136	0.65	0.977	0.884	2.93E-131	Macrophage	Tmem176b
1.32E-134	0.58	0.984	0.939	4.08E-130	Macrophage	Rpl30
1.33E-134	1.11	0.795	0.571	4.11E-130	Macrophage	Cebpb
8.14E-134	0.64	0.976	0.907	2.52E-129	Macrophage	Rps7
1.56E-133	0.91	0.634	0.272	4.82E-129	Macrophage	Cdkn1a
1.38E-131	1.13	0.884	0.58	4.27E-127	Macrophage	Fos
9.07E-131	0.80	0.74	0.479	2.81E-126	Macrophage	Actr3
3.38E-129	1.13	0.747	0.428	1.05E-124	Macrophage	Ier2
9.26E-129	0.81	0.627	0.329	2.87E-124	Macrophage	Pold4
1.85E-128	1.00	0.758	0.501	5.73E-124	Macrophage	Zfp36l2
5.86E-128	0.97	0.907	0.767	1.81E-123	Macrophage	Hspa5
7.10E-128	0.57	1	0.997	2.20E-123	Macrophage	Ftl1
8.12E-128	0.61	0.986	0.946	2.52E-123	Macrophage	Rps26
8.31E-126	0.55	0.991	0.965	2.58E-121	Macrophage	Rpl6
1.50E-125	0.58	0.986	0.928	4.65E-121	Macrophage	Rps19
9.54E-124	0.51	0.99	0.97	2.96E-119	Macrophage	Rpl35a
1.84E-123	1.90	0.64	0.31	5.70E-119	Macrophage	Hspa1a
1.26E-120	0.86	0.729	0.373	3.91E-116	Macrophage	Fosb
3.94E-120	0.57	0.977	0.939	1.22E-115	Macrophage	Rpl22
7.78E-120	0.58	0.991	0.961	2.41E-115	Macrophage	Rplp0
3.55E-119	0.78	0.879	0.719	1.10E-114	Macrophage	Pnrc1
3.84E-118	0.60	0.961	0.913	1.19E-113	Macrophage	Rpl10a
9.39E-116	0.90	0.785	0.591	2.91E-111	Macrophage	Nfe2l2
1.51E-111	0.61	0.94	0.858	4.68E-107	Macrophage	Eef1b2
9.11E-111	0.74	0.759	0.547	2.82E-106	Macrophage	Rap1b
4.80E-109	0.77	0.736	0.529	1.49E-104	Macrophage	Man2b1

1.50E-107	0.64	0.857	0.734	4.66E-103	Macrophage	Cdc42
1.07E-105	0.65	0.9	0.797	3.30E-101	Macrophage	Cfl1
2.18E-105	0.51	0.977	0.937	6.74E-101	Macrophage	Rps28
4.29E-105	0.62	0.838	0.715	1.33E-100	Macrophage	Arpc3
9.96E-104	0.74	0.73	0.489	3.09E-99	Macrophage	Ccnl1
1.96E-103	0.61	0.907	0.81	6.08E-99	Macrophage	Calm1
4.83E-103	0.64	0.857	0.745	1.50E-98	Macrophage	Eif3f
3.67E-101	0.52	0.964	0.919	1.14E-96	Macrophage	Rpl29
1.19E-100	0.71	0.608	0.33	3.69E-96	Macrophage	H2-T23
1.16E-99	0.79	0.974	0.976	3.59E-95	Macrophage	Ubc
3.74E-97	0.53	0.935	0.878	1.16E-92	Macrophage	Cd81
5.69E-95	0.67	0.749	0.54	1.76E-90	Macrophage	Rtn4
3.64E-87	0.81	0.692	0.513	1.13E-82	Macrophage	Sdcbp
2.22E-86	0.54	0.869	0.757	6.89E-82	Macrophage	Arpc2
2.76E-86	0.98	0.669	0.41	8.56E-82	Macrophage	Rhob
1.48E-85	0.62	0.783	0.636	4.58E-81	Macrophage	Ctsa
1.15E-81	0.62	0.758	0.62	3.56E-77	Macrophage	Picalm
3.44E-78	0.69	0.742	0.582	1.07E-73	Macrophage	Zfand5
1.97E-77	0.83	0.656	0.462	6.11E-73	Macrophage	Hmgb2
9.64E-77	0.59	0.674	0.426	2.99E-72	Macrophage	Anxa5
1.12E-76	0.60	0.657	0.443	3.48E-72	Macrophage	Tpm3
6.09E-75	0.58	0.74	0.621	1.89E-70	Macrophage	Erp29
2.72E-72	0.51	0.888	0.82	8.42E-68	Macrophage	Clta
5.43E-68	0.84	0.758	0.619	1.68E-63	Macrophage	Cited2
3.93E-66	0.57	0.752	0.664	1.22E-61	Macrophage	Rrbp1
5.62E-65	0.90	0.895	0.771	1.74E-60	Macrophage	Jun
1.12E-61	0.65	0.818	0.769	3.45E-57	Macrophage	Sqstm1
2.41E-60	0.54	0.846	0.816	7.48E-56	Macrophage	Eif4a1
5.07E-59	0.51	0.716	0.55	1.57E-54	Macrophage	Cotl1
1.01E-55	0.55	0.66	0.538	3.12E-51	Macrophage	Tpd52
5.43E-52	0.52	0.648	0.516	1.68E-47	Macrophage	Atp2b1
9.07E-52	0.55	0.878	0.74	2.81E-47	Macrophage	Neat1
1.67E-42	0.57	0.875	0.826	5.17E-38	Macrophage	Sat1
5.05E-33	0.82	0.865	0.879	1.56E-28	Macrophage	Hsp90aa1
1.73E-09	0.59	0.686	0.753	5.36E-05	Macrophage	Dnaja1
0.00E+00	3.24	0.98	0.058	0.00E+00	Intercalating cell of collecting duct	Atp6v1g3
0.00E+00	2.51	0.746	0.061	0.00E+00	Intercalating cell of collecting duct	Hsd11b2
0.00E+00	2.02	0.927	0.029	0.00E+00	Intercalating cell of collecting duct	Slc4a9
0.00E+00	1.90	0.693	0.009	0.00E+00	Intercalating cell of collecting duct	Aqp6
0.00E+00	1.87	0.822	0.043	0.00E+00	Intercalating cell of collecting duct	Cldn4
0.00E+00	1.82	0.613	0.04	0.00E+00	Intercalating cell of collecting duct	Aqp2
0.00E+00	1.78	0.958	0.261	0.00E+00	Intercalating cell of collecting duct	Tmem213
0.00E+00	1.77	0.899	0.007	0.00E+00	Intercalating cell of collecting duct	Foxi1
0.00E+00	1.76	0.874	0.169	0.00E+00	Intercalating cell of collecting duct	Trib1
0.00E+00	1.73	0.93	0.018	0.00E+00	Intercalating cell of collecting duct	Atp6v0d2
0.00E+00	1.71	0.94	0.355	0.00E+00	Intercalating cell of collecting duct	Pam
0.00E+00	1.68	0.884	0.036	0.00E+00	Intercalating cell of collecting duct	Rhbg
0.00E+00	1.64	0.837	0.181	0.00E+00	Intercalating cell of collecting duct	Aqp3
0.00E+00	1.63	0.917	0.071	0.00E+00	Intercalating cell of collecting duct	Rhcg
0.00E+00	1.63	0.985	0.761	0.00E+00	Intercalating cell of collecting duct	Atp6v1a
0.00E+00	1.57	0.862	0.02	0.00E+00	Intercalating cell of collecting duct	Atp6v1c2
0.00E+00	1.54	0.967	0.608	0.00E+00	Intercalating cell of collecting duct	Tfcp2l1
0.00E+00	1.50	0.804	0.026	0.00E+00	Intercalating cell of collecting duct	Spink8
0.00E+00	1.50	0.86	0.224	0.00E+00	Intercalating cell of collecting duct	Clu
0.00E+00	1.42	0.849	0.224	0.00E+00	Intercalating cell of collecting duct	Cebpd
0.00E+00	1.39	0.636	0.005	0.00E+00	Intercalating cell of collecting duct	Slc4a1
0.00E+00	1.37	0.749	0.101	0.00E+00	Intercalating cell of collecting duct	Slc8a1
0.00E+00	1.35	0.94	0.205	0.00E+00	Intercalating cell of collecting duct	Clcnkb
0.00E+00	1.30	0.817	0.078	0.00E+00	Intercalating cell of collecting duct	Krt18
0.00E+00	1.30	0.89	0.128	0.00E+00	Intercalating cell of collecting duct	Cldn8
0.00E+00	1.30	0.92	0.175	0.00E+00	Intercalating cell of collecting duct	Scnn1a
0.00E+00	1.29	0.899	0.339	0.00E+00	Intercalating cell of collecting duct	Car12
0.00E+00	1.29	0.892	0.127	0.00E+00	Intercalating cell of collecting duct	Cldn7
0.00E+00	1.28	0.927	0.239	0.00E+00	Intercalating cell of collecting duct	Efhd1

0.00E+00	1.28	0.832	0.198	0.00E+00	Intercalating cell of collecting duct	Tmem52b
0.00E+00	1.28	0.708	0.103	0.00E+00	Intercalating cell of collecting duct	Tacstd2
0.00E+00	1.26	0.953	0.313	0.00E+00	Intercalating cell of collecting duct	Mal
0.00E+00	1.26	0.925	0.291	0.00E+00	Intercalating cell of collecting duct	Aif1l
0.00E+00	1.26	0.784	0.042	0.00E+00	Intercalating cell of collecting duct	Scnn1b
0.00E+00	1.25	0.689	0.109	0.00E+00	Intercalating cell of collecting duct	Gm44120
0.00E+00	1.25	0.819	0.063	0.00E+00	Intercalating cell of collecting duct	Plet1
0.00E+00	1.22	0.797	0.032	0.00E+00	Intercalating cell of collecting duct	Oxgr1
0.00E+00	1.20	0.864	0.09	0.00E+00	Intercalating cell of collecting duct	Atp6v1b1
0.00E+00	1.20	0.817	0.164	0.00E+00	Intercalating cell of collecting duct	Nr4a1
0.00E+00	1.18	0.817	0.032	0.00E+00	Intercalating cell of collecting duct	Serpinb6b
0.00E+00	1.18	0.799	0.1	0.00E+00	Intercalating cell of collecting duct	Scd2
0.00E+00	1.18	0.744	0.121	0.00E+00	Intercalating cell of collecting duct	Maff
0.00E+00	1.13	0.885	0.278	0.00E+00	Intercalating cell of collecting duct	Cdh1
0.00E+00	1.10	0.822	0.182	0.00E+00	Intercalating cell of collecting duct	Lgals3
0.00E+00	1.10	0.782	0.048	0.00E+00	Intercalating cell of collecting duct	Ociad2
0.00E+00	1.10	0.922	0.208	0.00E+00	Intercalating cell of collecting duct	Epcam
0.00E+00	1.09	0.882	0.279	0.00E+00	Intercalating cell of collecting duct	Anxa4
0.00E+00	1.07	0.832	0.15	0.00E+00	Intercalating cell of collecting duct	Csrp1
0.00E+00	1.07	0.751	0.101	0.00E+00	Intercalating cell of collecting duct	Phlda1
0.00E+00	1.07	0.899	0.338	0.00E+00	Intercalating cell of collecting duct	Itpr2
0.00E+00	1.02	0.741	0.009	0.00E+00	Intercalating cell of collecting duct	Hepacam2
0.00E+00	1.00	0.889	0.192	0.00E+00	Intercalating cell of collecting duct	Krt7
0.00E+00	0.99	0.663	0.025	0.00E+00	Intercalating cell of collecting duct	Scnn1g
0.00E+00	0.98	0.781	0.052	0.00E+00	Intercalating cell of collecting duct	Gata3
0.00E+00	0.97	0.766	0.03	0.00E+00	Intercalating cell of collecting duct	Tmem117
0.00E+00	0.96	0.791	0.077	0.00E+00	Intercalating cell of collecting duct	Blnk
0.00E+00	0.95	0.787	0.179	0.00E+00	Intercalating cell of collecting duct	Cxadr
0.00E+00	0.94	0.714	0.037	0.00E+00	Intercalating cell of collecting duct	Ptges
0.00E+00	0.94	0.794	0.12	0.00E+00	Intercalating cell of collecting duct	Ckmt1
0.00E+00	0.92	0.709	0.044	0.00E+00	Intercalating cell of collecting duct	Olfm1
0.00E+00	0.92	0.741	0.077	0.00E+00	Intercalating cell of collecting duct	Mal2
0.00E+00	0.92	0.867	0.289	0.00E+00	Intercalating cell of collecting duct	Col18a1
0.00E+00	0.92	0.628	0.011	0.00E+00	Intercalating cell of collecting duct	Kit
0.00E+00	0.91	0.704	0.016	0.00E+00	Intercalating cell of collecting duct	Rhou
0.00E+00	0.90	0.698	0.135	0.00E+00	Intercalating cell of collecting duct	Emb
0.00E+00	0.88	0.744	0.053	0.00E+00	Intercalating cell of collecting duct	Serpinb9
0.00E+00	0.87	0.841	0.262	0.00E+00	Intercalating cell of collecting duct	Rnf152
0.00E+00	0.86	0.734	0.093	0.00E+00	Intercalating cell of collecting duct	Foxq1
0.00E+00	0.85	0.807	0.151	0.00E+00	Intercalating cell of collecting duct	Tmem50b
0.00E+00	0.85	0.814	0.106	0.00E+00	Intercalating cell of collecting duct	St14
0.00E+00	0.85	0.693	0.09	0.00E+00	Intercalating cell of collecting duct	Adgrf5
0.00E+00	0.84	0.719	0.112	0.00E+00	Intercalating cell of collecting duct	Elf3
0.00E+00	0.84	0.731	0.028	0.00E+00	Intercalating cell of collecting duct	Rcan2
0.00E+00	0.84	0.744	0.084	0.00E+00	Intercalating cell of collecting duct	Cav2
0.00E+00	0.83	0.767	0.16	0.00E+00	Intercalating cell of collecting duct	C77080
0.00E+00	0.82	0.703	0.069	0.00E+00	Intercalating cell of collecting duct	Slc35g1
0.00E+00	0.82	0.625	0.015	0.00E+00	Intercalating cell of collecting duct	Malrd1
0.00E+00	0.82	0.779	0.122	0.00E+00	Intercalating cell of collecting duct	Casz1
0.00E+00	0.80	0.826	0.176	0.00E+00	Intercalating cell of collecting duct	Mecom
0.00E+00	0.80	0.716	0.154	0.00E+00	Intercalating cell of collecting duct	Tdrp
0.00E+00	0.79	0.65	0.056	0.00E+00	Intercalating cell of collecting duct	Gata2
0.00E+00	0.79	0.673	0.05	0.00E+00	Intercalating cell of collecting duct	Sptbn2
0.00E+00	0.78	0.719	0.105	0.00E+00	Intercalating cell of collecting duct	Sowahc
0.00E+00	0.76	0.653	0.06	0.00E+00	Intercalating cell of collecting duct	Ehf
0.00E+00	0.76	0.784	0.199	0.00E+00	Intercalating cell of collecting duct	Cpeb2
0.00E+00	0.75	0.719	0.105	0.00E+00	Intercalating cell of collecting duct	Alcam
0.00E+00	0.74	0.746	0.114	0.00E+00	Intercalating cell of collecting duct	Smim5
0.00E+00	0.74	0.842	0.184	0.00E+00	Intercalating cell of collecting duct	Slc16a7
0.00E+00	0.72	0.603	0.018	0.00E+00	Intercalating cell of collecting duct	Tmem61
0.00E+00	0.71	0.65	0.006	0.00E+00	Intercalating cell of collecting duct	Avpr1a
0.00E+00	0.71	0.646	0.002	0.00E+00	Intercalating cell of collecting duct	Klkb1
0.00E+00	0.70	0.781	0.151	0.00E+00	Intercalating cell of collecting duct	Cds1
0.00E+00	0.70	0.716	0.115	0.00E+00	Intercalating cell of collecting duct	Tspan1

0.00E+00	0.70	0.661	0.036	0.00E+00	Intercalating cell of collecting duct	Plcg2
0.00E+00	0.68	0.724	0.071	0.00E+00	Intercalating cell of collecting duct	Kcnq1
0.00E+00	0.67	0.729	0.161	0.00E+00	Intercalating cell of collecting duct	Ermp1
0.00E+00	0.67	0.646	0.113	0.00E+00	Intercalating cell of collecting duct	Kcnj10
0.00E+00	0.65	0.625	0.016	0.00E+00	Intercalating cell of collecting duct	Syn2
0.00E+00	0.65	0.716	0.095	0.00E+00	Intercalating cell of collecting duct	Etl4
0.00E+00	0.63	0.618	0.089	0.00E+00	Intercalating cell of collecting duct	Thbs1
0.00E+00	0.63	0.762	0.181	0.00E+00	Intercalating cell of collecting duct	Twsg1
0.00E+00	0.63	0.623	0.059	0.00E+00	Intercalating cell of collecting duct	Dsp
0.00E+00	0.63	0.661	0.054	0.00E+00	Intercalating cell of collecting duct	Muc1
0.00E+00	0.60	0.606	0.015	0.00E+00	Intercalating cell of collecting duct	Parm1
0.00E+00	0.60	0.688	0.132	0.00E+00	Intercalating cell of collecting duct	Abr
0.00E+00	0.58	0.663	0.094	0.00E+00	Intercalating cell of collecting duct	Bsnd
0.00E+00	0.58	0.658	0.083	0.00E+00	Intercalating cell of collecting duct	Sort1
0.00E+00	0.56	0.734	0.12	0.00E+00	Intercalating cell of collecting duct	Zfhx3
0.00E+00	0.56	0.676	0.117	0.00E+00	Intercalating cell of collecting duct	Slit2
0.00E+00	0.56	0.603	0.09	0.00E+00	Intercalating cell of collecting duct	Serpinb1a
0.00E+00	0.50	0.618	0.092	0.00E+00	Intercalating cell of collecting duct	Mfsd6
4.21E-307	0.64	0.699	0.15	1.30E-302	Intercalating cell of collecting duct	Slc2a1
7.52E-303	0.84	0.869	0.261	2.33E-298	Intercalating cell of collecting duct	Pde1a
1.62E-301	1.51	0.927	0.369	5.01E-297	Intercalating cell of collecting duct	Defb1
8.96E-300	0.75	0.669	0.133	2.77E-295	Intercalating cell of collecting duct	Gstm2
1.78E-296	0.99	0.887	0.346	5.53E-292	Intercalating cell of collecting duct	Tmprss2
2.04E-296	0.86	0.857	0.3	6.31E-292	Intercalating cell of collecting duct	Rragd
1.10E-295	0.64	0.663	0.147	3.41E-291	Intercalating cell of collecting duct	Klhl21
1.63E-291	0.67	0.696	0.14	5.05E-287	Intercalating cell of collecting duct	Pgam2
3.80E-288	1.35	0.973	0.644	1.18E-283	Intercalating cell of collecting duct	Car2
1.35E-286	0.83	0.854	0.281	4.18E-282	Intercalating cell of collecting duct	Foxp1
2.89E-283	0.64	0.787	0.196	8.96E-279	Intercalating cell of collecting duct	Rap1gap
1.56E-280	1.40	0.887	0.318	4.82E-276	Intercalating cell of collecting duct	Atf3
2.28E-279	0.82	0.781	0.204	7.07E-275	Intercalating cell of collecting duct	Sox4
2.34E-279	0.57	0.794	0.183	7.24E-275	Intercalating cell of collecting duct	Hk1
9.74E-275	0.78	0.761	0.216	3.02E-270	Intercalating cell of collecting duct	Map3k1
1.23E-272	0.68	0.804	0.23	3.81E-268	Intercalating cell of collecting duct	Acsl5
4.01E-270	1.08	0.922	0.485	1.24E-265	Intercalating cell of collecting duct	Atp6ap2
7.18E-268	0.83	0.811	0.292	2.22E-263	Intercalating cell of collecting duct	Nedd4l
3.81E-265	0.91	0.819	0.229	1.18E-260	Intercalating cell of collecting duct	Gadd45b
1.82E-264	1.16	0.94	0.631	5.65E-260	Intercalating cell of collecting duct	Pgrmc1
3.82E-262	0.54	0.703	0.165	1.18E-257	Intercalating cell of collecting duct	Akr1c19
5.38E-261	0.63	0.688	0.167	1.67E-256	Intercalating cell of collecting duct	Hoxb9
3.34E-259	0.76	0.869	0.277	1.03E-254	Intercalating cell of collecting duct	Hoxd8
1.31E-258	1.31	0.932	0.415	4.06E-254	Intercalating cell of collecting duct	Sh3bgrl3
1.96E-257	0.66	0.797	0.244	6.07E-253	Intercalating cell of collecting duct	Bag4
4.99E-257	1.01	0.97	0.784	1.54E-252	Intercalating cell of collecting duct	Atp6v0e
1.11E-255	1.58	0.935	0.551	3.44E-251	Intercalating cell of collecting duct	Klf6
4.14E-254	0.55	0.724	0.182	1.28E-249	Intercalating cell of collecting duct	Hoxd9
2.28E-251	0.83	0.726	0.201	7.07E-247	Intercalating cell of collecting duct	Csrnp1
3.72E-251	0.62	0.669	0.173	1.15E-246	Intercalating cell of collecting duct	Fbxo33
4.79E-251	1.35	0.955	0.475	1.48E-246	Intercalating cell of collecting duct	Btg2
7.72E-251	0.57	0.603	0.132	2.39E-246	Intercalating cell of collecting duct	Prss23
1.81E-248	0.58	0.684	0.161	5.62E-244	Intercalating cell of collecting duct	Arl4d
1.90E-248	0.76	0.749	0.24	5.89E-244	Intercalating cell of collecting duct	Trak1
2.28E-246	1.54	0.917	0.479	7.06E-242	Intercalating cell of collecting duct	Egr1
1.12E-241	0.60	0.752	0.199	3.47E-237	Intercalating cell of collecting duct	Tspan8
1.31E-241	0.67	0.771	0.227	4.05E-237	Intercalating cell of collecting duct	Calb1
2.15E-241	0.67	0.774	0.222	6.67E-237	Intercalating cell of collecting duct	Pou3f3
1.81E-239	1.07	0.616	0.153	5.61E-235	Intercalating cell of collecting duct	Gm43305
4.02E-239	0.93	0.875	0.359	1.24E-234	Intercalating cell of collecting duct	Iqgap1
1.78E-237	0.95	0.987	0.835	5.51E-233	Intercalating cell of collecting duct	Atp6v1e1
6.74E-237	0.79	0.656	0.177	2.09E-232	Intercalating cell of collecting duct	Tbck
3.41E-236	0.93	0.786	0.279	1.06E-231	Intercalating cell of collecting duct	Irs2
6.66E-236	0.88	0.917	0.377	2.06E-231	Intercalating cell of collecting duct	Cd9
2.20E-234	0.79	0.997	0.99	6.81E-230	Intercalating cell of collecting duct	Itm2b
3.07E-233	0.78	0.834	0.288	9.50E-229	Intercalating cell of collecting duct	Ppp1r15a

5.73E-233	0.70	0.666	0.164	1.77E-228	Intercalating cell of collecting duct	Irf1
6.47E-231	0.69	0.769	0.24	2.00E-226	Intercalating cell of collecting duct	Litaf
1.64E-230	0.69	0.718	0.217	5.09E-226	Intercalating cell of collecting duct	Cish
5.04E-228	0.70	0.821	0.293	1.56E-223	Intercalating cell of collecting duct	Tanc1
1.93E-227	0.86	0.854	0.359	5.98E-223	Intercalating cell of collecting duct	Pmepa1
4.96E-226	0.98	0.894	0.461	1.54E-221	Intercalating cell of collecting duct	Eps8
1.00E-225	0.89	0.9	0.469	3.11E-221	Intercalating cell of collecting duct	Itgav
1.45E-225	1.15	0.967	0.685	4.49E-221	Intercalating cell of collecting duct	Serinc3
2.06E-225	0.69	0.786	0.254	6.38E-221	Intercalating cell of collecting duct	Cdkl1
1.24E-221	1.21	0.864	0.371	3.84E-217	Intercalating cell of collecting duct	Fosb
4.91E-219	0.71	0.839	0.326	1.52E-214	Intercalating cell of collecting duct	Pbxip1
7.24E-219	0.75	0.86	0.331	2.24E-214	Intercalating cell of collecting duct	Gadd45a
6.79E-217	0.99	0.915	0.513	2.10E-212	Intercalating cell of collecting duct	Adgrg1
3.70E-215	0.68	0.684	0.208	1.15E-210	Intercalating cell of collecting duct	Pard6b
1.60E-214	0.89	0.703	0.214	4.96E-210	Intercalating cell of collecting duct	Rnd3
4.85E-214	0.54	0.646	0.164	1.50E-209	Intercalating cell of collecting duct	Sbno2
3.21E-213	0.53	0.676	0.199	9.93E-209	Intercalating cell of collecting duct	Ptprj
1.36E-211	0.87	0.884	0.479	4.20E-207	Intercalating cell of collecting duct	Atp6ap1
7.75E-211	0.94	0.949	0.718	2.40E-206	Intercalating cell of collecting duct	Aplp2
1.60E-210	0.81	0.882	0.443	4.94E-206	Intercalating cell of collecting duct	Ptbp3
1.06E-208	1.51	0.904	0.532	3.29E-204	Intercalating cell of collecting duct	Chka
2.02E-208	0.73	0.919	0.341	6.25E-204	Intercalating cell of collecting duct	Pkm
7.18E-208	1.36	0.93	0.58	2.22E-203	Intercalating cell of collecting duct	Fos
7.68E-208	0.97	0.897	0.407	2.38E-203	Intercalating cell of collecting duct	Ier3
2.61E-204	1.15	0.89	0.48	8.08E-200	Intercalating cell of collecting duct	Hexb
5.15E-204	0.79	0.824	0.363	1.59E-199	Intercalating cell of collecting duct	Lsm6
1.17E-202	1.11	0.807	0.348	3.64E-198	Intercalating cell of collecting duct	Ifrd1
1.72E-202	0.59	0.728	0.237	5.33E-198	Intercalating cell of collecting duct	Snhg4
2.93E-199	0.53	0.749	0.241	9.07E-195	Intercalating cell of collecting duct	Laptm4b
1.11E-198	0.54	0.641	0.184	3.43E-194	Intercalating cell of collecting duct	Mafk
3.97E-198	0.62	0.772	0.275	1.23E-193	Intercalating cell of collecting duct	Trim2
3.32E-196	0.81	0.914	0.585	1.03E-191	Intercalating cell of collecting duct	Atp6v1d
7.59E-196	0.91	0.99	0.9	2.35E-191	Intercalating cell of collecting duct	Atp1b1
1.39E-195	0.82	0.846	0.388	4.30E-191	Intercalating cell of collecting duct	Clcn5
1.50E-194	0.92	0.887	0.369	4.66E-190	Intercalating cell of collecting duct	Wfdc2
1.27E-193	0.88	0.99	0.941	3.94E-189	Intercalating cell of collecting duct	Atp6v0c
2.62E-193	0.53	0.661	0.2	8.12E-189	Intercalating cell of collecting duct	Rela
4.88E-192	0.56	0.718	0.238	1.51E-187	Intercalating cell of collecting duct	Tbx2
3.82E-191	1.01	0.842	0.413	1.18E-186	Intercalating cell of collecting duct	Bhlhe40
1.26E-190	0.81	0.924	0.616	3.90E-186	Intercalating cell of collecting duct	Gclc
1.48E-190	0.56	0.744	0.266	4.58E-186	Intercalating cell of collecting duct	Clstn1
2.09E-190	0.72	0.784	0.321	6.48E-186	Intercalating cell of collecting duct	Map3k2
2.51E-188	0.52	0.733	0.229	7.77E-184	Intercalating cell of collecting duct	Cd82
3.49E-188	0.74	0.787	0.32	1.08E-183	Intercalating cell of collecting duct	Pdcd4
1.03E-187	1.00	0.949	0.647	3.20E-183	Intercalating cell of collecting duct	Aldh1l1
2.77E-187	0.79	0.821	0.374	8.57E-183	Intercalating cell of collecting duct	Stat3
5.58E-187	0.60	0.844	0.309	1.73E-182	Intercalating cell of collecting duct	Hes1
1.34E-186	0.71	0.975	0.888	4.15E-182	Intercalating cell of collecting duct	Atp6v1f
2.51E-186	0.96	0.673	0.235	7.78E-182	Intercalating cell of collecting duct	Mme
5.34E-186	0.80	0.885	0.464	1.65E-181	Intercalating cell of collecting duct	Ppargc1a
7.05E-186	0.75	0.824	0.396	2.18E-181	Intercalating cell of collecting duct	Dmxl1
5.13E-185	0.60	0.786	0.274	1.59E-180	Intercalating cell of collecting duct	Tacc1
3.42E-184	1.29	0.89	0.585	1.06E-179	Intercalating cell of collecting duct	Zfp36
1.26E-183	0.90	0.947	0.737	3.90E-179	Intercalating cell of collecting duct	Cdh16
3.64E-182	0.85	0.757	0.311	1.13E-177	Intercalating cell of collecting duct	Cdo1
1.79E-181	0.68	0.834	0.414	5.54E-177	Intercalating cell of collecting duct	Atp6v1h
2.92E-181	1.18	0.927	0.649	9.05E-177	Intercalating cell of collecting duct	Nudt4
1.02E-180	0.83	0.922	0.634	3.17E-176	Intercalating cell of collecting duct	Atp6v0a4
2.21E-180	0.80	0.995	0.962	6.84E-176	Intercalating cell of collecting duct	Actg1
7.08E-180	0.60	0.741	0.265	2.19E-175	Intercalating cell of collecting duct	Tuba1c
8.65E-179	0.84	0.957	0.809	2.68E-174	Intercalating cell of collecting duct	Sdc4
8.11E-178	0.67	0.862	0.458	2.51E-173	Intercalating cell of collecting duct	Mindy2
1.27E-175	0.74	0.937	0.665	3.94E-171	Intercalating cell of collecting duct	Camk2n1
1.31E-175	0.56	0.749	0.297	4.07E-171	Intercalating cell of collecting duct	Trp53inp2

2.47E-175	0.68	0.86	0.41	7.65E-171	Intercalating cell of collecting duct	Nfe2l1
2.90E-175	0.53	0.757	0.294	8.97E-171	Intercalating cell of collecting duct	Slc25a23
4.51E-175	0.91	0.925	0.546	1.40E-170	Intercalating cell of collecting duct	Junb
2.62E-174	0.59	0.611	0.161	8.11E-170	Intercalating cell of collecting duct	Pcolce
1.11E-171	0.73	0.915	0.525	3.45E-167	Intercalating cell of collecting duct	Btg1
4.31E-171	0.76	0.889	0.559	1.34E-166	Intercalating cell of collecting duct	Cd2ap
1.94E-170	0.76	0.899	0.53	6.02E-166	Intercalating cell of collecting duct	Gls
2.28E-170	0.63	0.829	0.38	7.05E-166	Intercalating cell of collecting duct	Dag1
4.30E-170	0.57	0.714	0.272	1.33E-165	Intercalating cell of collecting duct	Ralgapa2
5.44E-170	0.64	0.802	0.364	1.68E-165	Intercalating cell of collecting duct	Anxa11
3.37E-169	0.65	0.723	0.215	1.04E-164	Intercalating cell of collecting duct	Kcnj1
9.83E-168	0.54	0.792	0.338	3.05E-163	Intercalating cell of collecting duct	Spint1
2.96E-166	0.95	0.877	0.522	9.17E-162	Intercalating cell of collecting duct	Krt8
5.19E-166	0.64	0.771	0.312	1.61E-161	Intercalating cell of collecting duct	Fzd4
1.17E-165	0.65	0.874	0.425	3.62E-161	Intercalating cell of collecting duct	Paqr5
2.99E-165	0.70	0.746	0.309	9.26E-161	Intercalating cell of collecting duct	Tsc22d2
3.00E-165	0.69	0.849	0.407	9.31E-161	Intercalating cell of collecting duct	Tceal9
1.18E-164	0.80	0.827	0.303	3.66E-160	Intercalating cell of collecting duct	Cd24a
2.06E-164	0.60	0.784	0.351	6.38E-160	Intercalating cell of collecting duct	Elovl7
1.69E-162	0.58	0.762	0.28	5.24E-158	Intercalating cell of collecting duct	Hspa1b
1.82E-162	0.69	0.771	0.355	5.65E-158	Intercalating cell of collecting duct	Hif1a
2.25E-162	0.69	0.794	0.338	6.97E-158	Intercalating cell of collecting duct	Irf2bpl
4.25E-161	0.50	0.711	0.27	1.32E-156	Intercalating cell of collecting duct	Arhgap18
2.98E-160	0.59	0.822	0.41	9.24E-156	Intercalating cell of collecting duct	Enpp5
1.05E-159	0.52	0.754	0.301	3.26E-155	Intercalating cell of collecting duct	Iqgap2
2.30E-159	0.66	0.968	0.83	7.13E-155	Intercalating cell of collecting duct	Cystm1
3.09E-159	0.72	0.963	0.819	9.58E-155	Intercalating cell of collecting duct	Dstn
8.98E-159	0.76	0.887	0.529	2.78E-154	Intercalating cell of collecting duct	Slc43a2
6.47E-156	0.76	0.885	0.641	2.01E-151	Intercalating cell of collecting duct	Ptp4a2
2.52E-155	0.54	0.744	0.312	7.80E-151	Intercalating cell of collecting duct	Met
6.84E-153	0.58	0.973	0.918	2.12E-148	Intercalating cell of collecting duct	Gng5
2.93E-152	0.81	0.85	0.438	9.07E-148	Intercalating cell of collecting duct	Slc38a2
2.96E-152	0.63	0.877	0.475	9.17E-148	Intercalating cell of collecting duct	Etnk1
2.42E-151	0.63	0.713	0.265	7.49E-147	Intercalating cell of collecting duct	Ahnak
3.47E-150	1.08	0.786	0.405	1.07E-145	Intercalating cell of collecting duct	Hbegf
7.20E-150	0.70	0.895	0.58	2.23E-145	Intercalating cell of collecting duct	Wwc1
1.60E-149	0.65	0.721	0.307	4.94E-145	Intercalating cell of collecting duct	Srxn1
7.33E-148	0.66	0.897	0.589	2.27E-143	Intercalating cell of collecting duct	Nfe2l2
3.24E-146	0.51	0.731	0.312	1.00E-141	Intercalating cell of collecting duct	Pcyox1
4.87E-145	0.69	0.85	0.427	1.51E-140	Intercalating cell of collecting duct	Ier2
6.25E-144	0.56	0.865	0.423	1.93E-139	Intercalating cell of collecting duct	Anxa5
2.35E-143	0.76	0.912	0.705	7.27E-139	Intercalating cell of collecting duct	Ivns1abp
3.29E-143	0.71	0.953	0.813	1.02E-138	Intercalating cell of collecting duct	Atp6v0b
1.35E-142	0.57	0.847	0.465	4.17E-138	Intercalating cell of collecting duct	Mindy1
2.22E-142	0.51	0.748	0.334	6.87E-138	Intercalating cell of collecting duct	Crb3
3.47E-142	0.55	0.741	0.336	1.08E-137	Intercalating cell of collecting duct	Ube2h
5.27E-141	0.53	0.771	0.344	1.63E-136	Intercalating cell of collecting duct	Nfat5
9.35E-141	0.68	0.807	0.402	2.90E-136	Intercalating cell of collecting duct	Fam13a
1.12E-140	0.57	0.822	0.424	3.47E-136	Intercalating cell of collecting duct	Zmiz1
9.95E-139	0.52	0.816	0.408	3.08E-134	Intercalating cell of collecting duct	Mprip
2.87E-138	0.63	0.879	0.555	8.88E-134	Intercalating cell of collecting duct	Dst
2.06E-137	0.56	0.741	0.322	6.38E-133	Intercalating cell of collecting duct	Kdm6b
5.77E-137	0.53	0.791	0.394	1.79E-132	Intercalating cell of collecting duct	Cab39
1.37E-136	0.61	0.96	0.824	4.23E-132	Intercalating cell of collecting duct	Ctsd
3.41E-135	0.59	0.912	0.695	1.06E-130	Intercalating cell of collecting duct	Rtn3
7.50E-135	0.67	0.834	0.487	2.32E-130	Intercalating cell of collecting duct	Ccnl1
1.38E-134	0.66	0.874	0.572	4.29E-130	Intercalating cell of collecting duct	Cnn3
1.46E-133	0.60	0.872	0.52	4.53E-129	Intercalating cell of collecting duct	Gstt1
2.01E-131	0.58	0.792	0.423	6.24E-127	Intercalating cell of collecting duct	Arhgap24
3.71E-131	0.54	0.724	0.335	1.15E-126	Intercalating cell of collecting duct	Celsr2
2.50E-130	0.55	0.704	0.295	7.75E-126	Intercalating cell of collecting duct	Ddit3
4.81E-130	0.75	0.945	0.817	1.49E-125	Intercalating cell of collecting duct	S100a1
1.77E-126	0.57	0.844	0.53	5.48E-122	Intercalating cell of collecting duct	Ddx6
2.93E-126	0.58	0.92	0.672	9.07E-122	Intercalating cell of collecting duct	Spint2

4.62E-126	0.65	0.721	0.34	1.43E-121	Intercalating cell of collecting duct	Midn
5.33E-126	0.55	0.855	0.491	1.65E-121	Intercalating cell of collecting duct	Mbnl2
4.12E-125	1.87	0.949	0.816	1.28E-120	Intercalating cell of collecting duct	S100g
7.26E-123	0.71	0.862	0.51	2.25E-118	Intercalating cell of collecting duct	S100a11
7.94E-123	0.57	0.884	0.623	2.46E-118	Intercalating cell of collecting duct	Wls
1.98E-122	0.58	0.792	0.407	6.15E-118	Intercalating cell of collecting duct	Jup
5.49E-119	0.56	0.973	0.851	1.70E-114	Intercalating cell of collecting duct	Jund
6.13E-119	0.65	0.761	0.382	1.90E-114	Intercalating cell of collecting duct	Dnajb1
6.67E-119	0.62	0.831	0.522	2.07E-114	Intercalating cell of collecting duct	Col4a4
1.35E-118	0.52	0.875	0.407	4.17E-114	Intercalating cell of collecting duct	Acss1
9.45E-117	0.55	0.904	0.7	2.93E-112	Intercalating cell of collecting duct	Ctnnb1
5.73E-116	0.51	0.812	0.479	1.78E-111	Intercalating cell of collecting duct	Pkn2
1.02E-114	0.68	0.889	0.667	3.17E-110	Intercalating cell of collecting duct	Ddx3x
1.68E-114	0.97	0.952	0.734	5.20E-110	Intercalating cell of collecting duct	Spp1
3.16E-112	0.50	0.713	0.345	9.77E-108	Intercalating cell of collecting duct	Map2k3
8.08E-112	0.54	0.831	0.509	2.50E-107	Intercalating cell of collecting duct	Glg1
1.91E-110	0.53	0.816	0.493	5.93E-106	Intercalating cell of collecting duct	Plxnb2
6.95E-110	0.54	0.865	0.574	2.15E-105	Intercalating cell of collecting duct	Purb
2.48E-109	0.52	0.806	0.48	7.67E-105	Intercalating cell of collecting duct	900097C17Ri
6.52E-109	0.57	0.849	0.558	2.02E-104	Intercalating cell of collecting duct	Bicc1
7.42E-109	0.58	0.945	0.77	2.30E-104	Intercalating cell of collecting duct	Jun
1.27E-108	0.57	0.86	0.598	3.92E-104	Intercalating cell of collecting duct	Ctnna1
1.79E-108	0.59	0.935	0.783	5.54E-104	Intercalating cell of collecting duct	Ezr
2.96E-106	0.51	0.85	0.519	9.17E-102	Intercalating cell of collecting duct	Nfkbia
5.32E-105	0.51	0.924	0.735	1.65E-100	Intercalating cell of collecting duct	Ogdh
1.62E-104	0.53	0.841	0.558	5.02E-100	Intercalating cell of collecting duct	Nfic
4.30E-100	0.58	0.934	0.739	1.33E-95	Intercalating cell of collecting duct	Neat1
4.82E-100	0.51	0.865	0.578	1.49E-95	Intercalating cell of collecting duct	Alkbh5
4.05E-99	0.51	0.779	0.456	1.25E-94	Intercalating cell of collecting duct	Syne2
2.06E-98	1.10	0.673	0.351	6.39E-94	Intercalating cell of collecting duct	Npnt
4.52E-94	0.59	0.847	0.565	1.40E-89	Intercalating cell of collecting duct	Irf2bp2
1.30E-92	0.51	0.826	0.523	4.02E-88	Intercalating cell of collecting duct	Ptprf
1.67E-87	1.69	0.872	0.618	5.18E-83	Intercalating cell of collecting duct	Klk1
2.15E-87	0.53	0.841	0.463	6.67E-83	Intercalating cell of collecting duct	Ly6e
5.96E-78	0.62	0.764	0.49	1.85E-73	Intercalating cell of collecting duct	Lmna
2.85E-60	0.53	0.85	0.69	8.82E-56	Intercalating cell of collecting duct	Zfp36l1
3.19E-50	0.78	0.721	0.585	9.87E-46	Intercalating cell of collecting duct	Kl
2.21E-34	0.68	0.608	0.498	6.83E-30	Intercalating cell of collecting duct	Tmem229a
0.00E+00	3.19	0.974	0.063	0.00E+00	Principal cell of collecting duct	Hsd11b2
0.00E+00	3.18	0.847	0.041	0.00E+00	Principal cell of collecting duct	Aqp2
0.00E+00	2.74	0.645	0.076	0.00E+00	Principal cell of collecting duct	Fxyd4
0.00E+00	2.01	0.967	0.077	0.00E+00	Principal cell of collecting duct	Rhcg
0.00E+00	1.86	0.882	0.104	0.00E+00	Principal cell of collecting duct	Slc8a1
0.00E+00	1.80	0.944	0.045	0.00E+00	Principal cell of collecting duct	Scnn1b
0.00E+00	1.69	0.811	0.112	0.00E+00	Principal cell of collecting duct	Gm44120
0.00E+00	1.62	0.903	0.105	0.00E+00	Principal cell of collecting duct	Tacstd2
0.00E+00	1.61	0.992	0.266	0.00E+00	Principal cell of collecting duct	Tmem213
0.00E+00	1.61	0.893	0.043	0.00E+00	Principal cell of collecting duct	Rhbg
0.00E+00	1.60	0.928	0.185	0.00E+00	Principal cell of collecting duct	Lgals3
0.00E+00	1.54	0.783	0.05	0.00E+00	Principal cell of collecting duct	Cldn4
0.00E+00	1.47	0.852	0.142	0.00E+00	Principal cell of collecting duct	Pgam2
0.00E+00	1.45	0.777	0.085	0.00E+00	Principal cell of collecting duct	Krt18
0.00E+00	1.36	0.652	0.071	0.00E+00	Principal cell of collecting duct	Apela
0.00E+00	1.32	0.87	0.053	0.00E+00	Principal cell of collecting duct	Tmem45b
0.00E+00	1.31	0.872	0.027	0.00E+00	Principal cell of collecting duct	Scnn1g
0.00E+00	1.30	0.91	0.197	0.00E+00	Principal cell of collecting duct	Krt7
0.00E+00	1.27	0.885	0.117	0.00E+00	Principal cell of collecting duct	Tspan1
0.00E+00	1.25	0.893	0.087	0.00E+00	Principal cell of collecting duct	Cav2
0.00E+00	1.23	0.959	0.2	0.00E+00	Principal cell of collecting duct	Tspan8
0.00E+00	1.23	0.875	0.105	0.00E+00	Principal cell of collecting duct	Scd2
0.00E+00	1.19	0.939	0.181	0.00E+00	Principal cell of collecting duct	Scnn1a
0.00E+00	1.18	0.841	0.04	0.00E+00	Principal cell of collecting duct	Ptges
0.00E+00	1.12	0.818	0.134	0.00E+00	Principal cell of collecting duct	Cldn7
0.00E+00	1.12	0.875	0.134	0.00E+00	Principal cell of collecting duct	Cldn8

0.00E+00	1.02	0.793	0.067	0.00E+00	Principal cell of collecting duct	Ptgs1
0.00E+00	1.02	0.783	0.049	0.00E+00	Principal cell of collecting duct	Olfm1
0.00E+00	0.98	0.662	0.064	0.00E+00	Principal cell of collecting duct	Ehf
0.00E+00	0.97	0.701	0.06	0.00E+00	Principal cell of collecting duct	Gata2
0.00E+00	0.95	0.78	0.119	0.00E+00	Principal cell of collecting duct	Smim5
0.00E+00	0.94	0.757	0.059	0.00E+00	Principal cell of collecting duct	Gata3
0.00E+00	0.92	0.721	0.084	0.00E+00	Principal cell of collecting duct	Blnk
0.00E+00	0.92	0.65	0.055	0.00E+00	Principal cell of collecting duct	Sptbn2
0.00E+00	0.91	0.716	0.066	0.00E+00	Principal cell of collecting duct	Cav1
0.00E+00	0.91	0.621	0.024	0.00E+00	Principal cell of collecting duct	Avpr2
0.00E+00	0.91	0.765	0.06	0.00E+00	Principal cell of collecting duct	Phactr1
0.00E+00	0.89	0.621	0.065	0.00E+00	Principal cell of collecting duct	Rasd1
0.00E+00	0.88	0.78	0.115	0.00E+00	Principal cell of collecting duct	Kcnj10
0.00E+00	0.85	0.634	0.084	0.00E+00	Principal cell of collecting duct	Mal2
0.00E+00	0.82	0.77	0.098	0.00E+00	Principal cell of collecting duct	Atp6v1b1
0.00E+00	0.81	0.606	0.035	0.00E+00	Principal cell of collecting duct	Spink8
0.00E+00	0.76	0.662	0.048	0.00E+00	Principal cell of collecting duct	L1cam
0.00E+00	0.71	0.678	0.075	0.00E+00	Principal cell of collecting duct	Slc35g1
0.00E+00	0.64	0.662	0.077	0.00E+00	Principal cell of collecting duct	Kcnq1
5.56E-302	1.28	0.941	0.229	1.72E-297	Principal cell of collecting duct	Calb1
1.01E-300	1.23	0.964	0.216	3.14E-296	Principal cell of collecting duct	Kcnj1
3.43E-299	2.26	0.905	0.228	1.06E-294	Principal cell of collecting duct	Clu
1.95E-298	2.27	0.811	0.187	6.05E-294	Principal cell of collecting duct	Aqp3
1.16E-296	0.93	0.811	0.15	3.60E-292	Principal cell of collecting duct	Tesc
2.63E-291	1.69	0.882	0.202	8.14E-287	Principal cell of collecting duct	Tmem52b
6.47E-281	0.71	0.611	0.085	2.00E-276	Principal cell of collecting duct	Atp4a
1.61E-279	1.22	0.936	0.211	4.98E-275	Principal cell of collecting duct	Clcnkb
4.10E-276	1.58	0.99	0.318	1.27E-271	Principal cell of collecting duct	Mal
9.69E-275	0.89	0.701	0.131	3.00E-270	Principal cell of collecting duct	Tpd52l1
5.58E-272	0.88	0.839	0.163	1.73E-267	Principal cell of collecting duct	Tfap2b
7.52E-269	1.45	0.936	0.312	2.33E-264	Principal cell of collecting duct	Cdo1
1.37E-265	0.73	0.614	0.094	4.24E-261	Principal cell of collecting duct	Serpinb1a
2.33E-264	1.91	0.99	0.372	7.21E-260	Principal cell of collecting duct	Wfdc2
1.02E-261	0.64	0.675	0.111	3.15E-257	Principal cell of collecting duct	Slc29a1
3.16E-257	0.63	0.614	0.094	9.79E-253	Principal cell of collecting duct	Thbs1
5.16E-251	0.55	0.652	0.103	1.60E-246	Principal cell of collecting duct	Kitl
6.63E-250	1.62	1	0.372	2.05E-245	Principal cell of collecting duct	Defb1
1.25E-246	0.65	0.693	0.121	3.88E-242	Principal cell of collecting duct	Slit2
7.32E-244	1.11	0.885	0.257	2.27E-239	Principal cell of collecting duct	Cdkl1
2.48E-241	1.05	0.91	0.214	7.68E-237	Principal cell of collecting duct	Epcam
1.58E-237	1.39	0.982	0.38	4.89E-233	Principal cell of collecting duct	Cd9
2.71E-237	1.86	0.985	0.418	8.41E-233	Principal cell of collecting duct	Sh3bgrl3
1.15E-227	0.64	0.691	0.127	3.55E-223	Principal cell of collecting duct	Ckmt1
9.87E-225	0.77	0.752	0.179	3.06E-220	Principal cell of collecting duct	Tmem178
2.64E-221	0.64	0.726	0.147	8.19E-217	Principal cell of collecting duct	Tspan12
4.55E-221	1.03	0.89	0.283	1.41E-216	Principal cell of collecting duct	Cdh1
2.81E-220	0.64	0.668	0.129	8.71E-216	Principal cell of collecting duct	Casz1
2.12E-219	1.13	0.913	0.296	6.56E-215	Principal cell of collecting duct	Aif1l
1.42E-215	0.62	0.652	0.127	4.39E-211	Principal cell of collecting duct	Grb14
4.69E-213	0.72	0.711	0.154	1.45E-208	Principal cell of collecting duct	Slc2a1
1.55E-212	0.52	0.639	0.114	4.82E-208	Principal cell of collecting duct	St14
1.99E-212	0.72	0.742	0.169	6.16E-208	Principal cell of collecting duct	Akr1c19
6.05E-211	1.04	0.67	0.137	1.88E-206	Principal cell of collecting duct	Gstm2
8.84E-211	1.08	0.721	0.18	2.74E-206	Principal cell of collecting duct	Tbck
1.35E-209	0.76	0.834	0.189	4.17E-205	Principal cell of collecting duct	Slc16a7
9.48E-209	1.41	0.985	0.738	2.94E-204	Principal cell of collecting duct	Cdh16
1.36E-198	0.73	0.747	0.186	4.20E-194	Principal cell of collecting duct	Hoxd9
2.31E-196	0.70	0.785	0.174	7.14E-192	Principal cell of collecting duct	Sostdc1
4.30E-196	0.66	0.693	0.166	1.33E-191	Principal cell of collecting duct	C77080
1.02E-195	0.72	0.714	0.166	3.17E-191	Principal cell of collecting duct	Ermp1
1.76E-192	0.70	0.77	0.182	5.45E-188	Principal cell of collecting duct	Mecom
1.49E-184	0.75	0.793	0.241	4.62E-180	Principal cell of collecting duct	Tbx2
1.54E-184	0.85	1	0.99	4.76E-180	Principal cell of collecting duct	Itm2b
3.41E-180	1.38	0.992	0.65	1.06E-175	Principal cell of collecting duct	Nudt4

1.20E-179	1.16	0.946	0.516	3.72E-175	Principal cell of collecting duct	Adgrg1
4.98E-173	0.81	0.795	0.232	1.54E-168	Principal cell of collecting duct	Cd82
3.98E-172	0.94	0.857	0.351	1.23E-167	Principal cell of collecting duct	Tmprss2
4.77E-170	0.71	0.785	0.227	1.48E-165	Principal cell of collecting duct	Pou3f3
2.74E-169	0.83	0.852	0.282	8.50E-165	Principal cell of collecting duct	Hoxd8
5.18E-166	0.59	0.624	0.138	1.61E-161	Principal cell of collecting duct	Pea15a
2.72E-164	0.77	0.765	0.235	8.43E-160	Principal cell of collecting duct	Acsl5
1.50E-163	1.09	0.959	0.611	4.66E-159	Principal cell of collecting duct	Tfcp2l1
4.43E-163	1.03	1	0.9	1.37E-158	Principal cell of collecting duct	Atp1b1
7.22E-159	0.60	0.762	0.202	2.24E-154	Principal cell of collecting duct	Bcam
2.92E-152	0.77	0.949	0.345	9.04E-148	Principal cell of collecting duct	Pkm
6.43E-152	1.16	0.985	0.687	1.99E-147	Principal cell of collecting duct	Serinc3
4.27E-150	0.87	0.831	0.358	1.32E-145	Principal cell of collecting duct	Hif1a
1.96E-148	0.88	0.982	0.786	6.06E-144	Principal cell of collecting duct	Atp6v0e
2.24E-144	0.82	0.816	0.315	6.93E-140	Principal cell of collecting duct	Fzd4
4.65E-144	0.92	0.913	0.523	1.44E-139	Principal cell of collecting duct	Gstt1
9.37E-144	0.67	0.806	0.247	2.90E-139	Principal cell of collecting duct	Efhd1
1.92E-143	0.71	0.795	0.304	5.94E-139	Principal cell of collecting duct	Iqgap2
8.92E-141	0.89	0.864	0.363	2.76E-136	Principal cell of collecting duct	Iqgap1
3.56E-140	0.83	0.831	0.367	1.10E-135	Principal cell of collecting duct	Anxa11
1.43E-139	0.55	0.716	0.202	4.44E-135	Principal cell of collecting duct	Rap1gap
1.07E-135	0.87	0.76	0.268	3.31E-131	Principal cell of collecting duct	Ahnak
4.52E-133	0.65	0.793	0.295	1.40E-128	Principal cell of collecting duct	Col18a1
2.05E-132	1.31	0.816	0.352	6.35E-128	Principal cell of collecting duct	Npnt
5.34E-130	0.67	0.826	0.331	1.66E-125	Principal cell of collecting duct	Pbxip1
1.91E-129	0.60	0.632	0.184	5.93E-125	Principal cell of collecting duct	Emx1
1.63E-125	0.67	0.777	0.302	5.06E-121	Principal cell of collecting duct	Smco4
3.29E-125	0.80	0.982	0.81	1.02E-120	Principal cell of collecting duct	Sdc4
9.84E-125	0.64	0.803	0.335	3.05E-120	Principal cell of collecting duct	Ddr1
7.31E-124	0.65	0.752	0.28	2.26E-119	Principal cell of collecting duct	Trim2
1.19E-123	0.91	0.959	0.719	3.67E-119	Principal cell of collecting duct	Aplp2
1.04E-122	0.50	0.701	0.235	3.23E-118	Principal cell of collecting duct	Tgfbr1
9.81E-122	0.75	0.811	0.364	3.04E-117	Principal cell of collecting duct	Pmepa1
1.17E-120	0.74	0.678	0.233	3.63E-116	Principal cell of collecting duct	Rnf186
1.37E-119	0.79	0.893	0.523	4.25E-115	Principal cell of collecting duct	Col4a4
1.20E-118	0.75	0.867	0.307	3.72E-114	Principal cell of collecting duct	Cd24a
1.65E-118	0.77	0.959	0.706	5.11E-114	Principal cell of collecting duct	Ivns1abp
1.74E-117	0.69	0.772	0.324	5.38E-113	Principal cell of collecting duct	Pdcd4
4.25E-117	0.71	0.977	0.889	1.32E-112	Principal cell of collecting duct	Atp6v1f
1.10E-113	0.56	0.693	0.253	3.40E-109	Principal cell of collecting duct	Flnb
1.10E-113	0.64	0.754	0.298	3.41E-109	Principal cell of collecting duct	Tanc1
1.37E-113	2.86	0.949	0.817	4.23E-109	Principal cell of collecting duct	S100g
9.30E-113	0.76	0.964	0.826	2.88E-108	Principal cell of collecting duct	Ctsd
4.42E-112	1.03	0.934	0.465	1.37E-107	Principal cell of collecting duct	Ly6e
2.02E-109	0.68	0.859	0.414	6.27E-105	Principal cell of collecting duct	Nfe2l1
1.96E-105	1.29	0.862	0.584	6.08E-101	Principal cell of collecting duct	Kl
2.48E-105	0.51	0.68	0.242	7.69E-101	Principal cell of collecting duct	Snhg4
2.75E-104	0.73	0.957	0.818	8.52E-100	Principal cell of collecting duct	S100a1
3.67E-103	0.57	0.698	0.271	1.14E-98	Principal cell of collecting duct	Clstn1
8.24E-102	0.69	0.908	0.625	2.55E-97	Principal cell of collecting duct	Wls
1.16E-98	0.52	0.749	0.279	3.59E-94	Principal cell of collecting duct	Tacc1
9.07E-98	0.64	0.734	0.338	2.81E-93	Principal cell of collecting duct	Celsr2
9.77E-96	0.61	0.857	0.428	3.03E-91	Principal cell of collecting duct	Paqr5
3.08E-95	0.72	0.88	0.6	9.55E-91	Principal cell of collecting duct	Ctnna1
1.05E-94	0.68	0.957	0.831	3.26E-90	Principal cell of collecting duct	Cystm1
1.60E-94	0.53	0.67	0.269	4.96E-90	Principal cell of collecting duct	Peli2
5.65E-94	0.62	0.729	0.294	1.75E-89	Principal cell of collecting duct	Gsta4
1.53E-92	0.66	0.834	0.457	4.75E-88	Principal cell of collecting duct	Syne2
3.79E-92	0.59	0.77	0.314	1.17E-87	Principal cell of collecting duct	Hes1
5.56E-92	0.53	0.616	0.223	1.72E-87	Principal cell of collecting duct	Map3k1
1.19E-91	0.75	0.959	0.698	3.69E-87	Principal cell of collecting duct	Txnip
6.90E-91	0.54	0.772	0.364	2.14E-86	Principal cell of collecting duct	Pkhd1
2.77E-90	0.76	0.887	0.528	8.58E-86	Principal cell of collecting duct	Tmsb4x
1.86E-89	0.57	0.703	0.316	5.77E-85	Principal cell of collecting duct	Pcyox1

1.99E-89	0.67	0.788	0.426	6.17E-85	Principal cell of collecting duct	Arhgap24
6.91E-88	0.54	0.714	0.326	2.14E-83	Principal cell of collecting duct	Pigt
7.42E-88	0.55	0.696	0.298	2.30E-83	Principal cell of collecting duct	Nedd4l
2.22E-87	0.68	0.859	0.534	6.89E-83	Principal cell of collecting duct	Gls
3.27E-87	0.59	0.816	0.439	1.01E-82	Principal cell of collecting duct	Lman1
6.12E-87	2.96	0.885	0.62	1.90E-82	Principal cell of collecting duct	Klk1
1.32E-85	0.67	0.857	0.535	4.10E-81	Principal cell of collecting duct	ccdc198
1.85E-85	0.60	0.839	0.495	5.73E-81	Principal cell of collecting duct	Plxnb2
1.95E-84	0.64	0.934	0.763	6.04E-80	Principal cell of collecting duct	Atp6v1a
8.86E-83	0.52	0.68	0.307	2.74E-78	Principal cell of collecting duct	Rragd
3.44E-82	0.54	0.703	0.326	1.06E-77	Principal cell of collecting duct	Cbx5
1.15E-81	0.51	0.977	0.93	3.57E-77	Principal cell of collecting duct	Aldoa
1.57E-81	0.60	0.951	0.674	4.87E-77	Principal cell of collecting duct	Spint2
4.59E-81	0.59	0.785	0.449	1.42E-76	Principal cell of collecting duct	Pgp
1.05E-78	1.21	0.923	0.736	3.25E-74	Principal cell of collecting duct	Spp1
1.25E-78	0.66	0.99	0.964	3.87E-74	Principal cell of collecting duct	Atp1a1
4.66E-77	0.60	0.867	0.575	1.44E-72	Principal cell of collecting duct	Cnn3
1.95E-74	0.57	0.675	0.34	6.05E-70	Principal cell of collecting duct	Ube2h
1.07E-73	0.55	0.862	0.56	3.31E-69	Principal cell of collecting duct	Nfic
2.18E-73	0.53	0.803	0.464	6.76E-69	Principal cell of collecting duct	F11r
1.43E-72	0.55	0.826	0.512	4.43E-68	Principal cell of collecting duct	Glg1
1.70E-72	0.63	0.762	0.411	5.27E-68	Principal cell of collecting duct	Tceal9
3.53E-72	0.51	0.76	0.414	1.09E-67	Principal cell of collecting duct	Enpp5
1.43E-67	0.68	0.757	0.354	4.42E-63	Principal cell of collecting duct	Kng2
6.78E-67	0.52	0.806	0.469	2.10E-62	Principal cell of collecting duct	Ppargc1a
4.62E-65	0.50	0.926	0.726	1.43E-60	Principal cell of collecting duct	Tspo
8.82E-65	0.54	0.852	0.584	2.73E-60	Principal cell of collecting duct	Wwc1
1.21E-61	0.55	0.711	0.385	3.75E-57	Principal cell of collecting duct	Rogdi
1.27E-61	0.61	0.634	0.314	3.92E-57	Principal cell of collecting duct	Tsc22d2
1.48E-59	0.58	0.826	0.563	4.58E-55	Principal cell of collecting duct	Cd2ap
3.45E-59	0.51	0.813	0.525	1.07E-54	Principal cell of collecting duct	Ptprf
1.99E-58	0.94	0.703	0.498	6.15E-54	Principal cell of collecting duct	Tmem229a
1.91E-53	0.52	0.844	0.643	5.91E-49	Principal cell of collecting duct	Ptp4a2
4.77E-53	0.87	0.785	0.526	1.48E-48	Principal cell of collecting duct	Krt8
3.27E-52	1.12	0.795	0.537	1.01E-47	Principal cell of collecting duct	Chka
1.24E-48	0.53	0.606	0.327	3.85E-44	Principal cell of collecting duct	Map3k2
2.85E-48	0.74	0.813	0.58	8.83E-44	Principal cell of collecting duct	Wnk1
2.18E-44	0.72	0.908	0.741	6.76E-40	Principal cell of collecting duct	Neat1
1.39E-42	0.60	0.708	0.414	4.29E-38	Principal cell of collecting duct	Ier3
4.56E-40	0.55	0.962	0.819	1.41E-35	Principal cell of collecting duct	Pdzk1ip1
2.24E-34	0.51	0.765	0.481	6.94E-30	Principal cell of collecting duct	Btg2
1.85E-28	0.54	0.739	0.515	5.72E-24	Principal cell of collecting duct	S100a11
4.39E-25	0.67	0.662	0.487	1.36E-20	Principal cell of collecting duct	Egr1
1.91E-21	0.82	0.685	0.587	5.92E-17	Principal cell of collecting duct	Fos
3.19E-14	0.70	0.67	0.559	9.87E-10	Principal cell of collecting duct	Klf6
0.00E+00	3.79	0.873	0.042	0.00E+00	Fibroblast	Lyz2
0.00E+00	2.98	0.994	0.037	0.00E+00	Fibroblast	Tyrobp
0.00E+00	2.94	0.898	0.034	0.00E+00	Fibroblast	Il1b
0.00E+00	2.81	0.981	0.045	0.00E+00	Fibroblast	Cd52
0.00E+00	2.69	0.914	0.045	0.00E+00	Fibroblast	Fcer1g
0.00E+00	2.38	0.997	0.129	0.00E+00	Fibroblast	Srgn
0.00E+00	2.27	0.94	0.036	0.00E+00	Fibroblast	Coro1a
0.00E+00	2.27	0.927	0.141	0.00E+00	Fibroblast	Ifitm3
0.00E+00	2.22	0.975	0.107	0.00E+00	Fibroblast	Fxyd5
0.00E+00	2.21	0.651	0.023	0.00E+00	Fibroblast	Wfdc17
0.00E+00	2.16	0.803	0.019	0.00E+00	Fibroblast	Ccl6
0.00E+00	2.15	0.933	0.031	0.00E+00	Fibroblast	Alox5ap
0.00E+00	2.13	0.93	0.021	0.00E+00	Fibroblast	Lsp1
0.00E+00	2.11	0.724	0.026	0.00E+00	Fibroblast	Cybb
0.00E+00	2.08	0.819	0.104	0.00E+00	Fibroblast	Vim
0.00E+00	2.01	0.914	0.175	0.00E+00	Fibroblast	Pim1
0.00E+00	2.00	0.943	0.037	0.00E+00	Fibroblast	Laptm5
0.00E+00	1.98	0.686	0.056	0.00E+00	Fibroblast	Cd14
0.00E+00	1.96	0.933	0.135	0.00E+00	Fibroblast	Cytip

0.00E+00	1.94	0.946	0.041	0.00E+00	Fibroblast	Ptprc
0.00E+00	1.94	0.838	0.044	0.00E+00	Fibroblast	Slfn2
0.00E+00	1.92	0.933	0.082	0.00E+00	Fibroblast	Lcp1
0.00E+00	1.89	0.841	0.118	0.00E+00	Fibroblast	S100a6
0.00E+00	1.88	0.886	0.019	0.00E+00	Fibroblast	Plek
0.00E+00	1.87	0.825	0.016	0.00E+00	Fibroblast	Cd44
0.00E+00	1.87	0.844	0.076	0.00E+00	Fibroblast	Rel
0.00E+00	1.84	0.886	0.037	0.00E+00	Fibroblast	Ctss
0.00E+00	1.80	0.613	0.046	0.00E+00	Fibroblast	Ifi27l2a
0.00E+00	1.80	0.863	0.062	0.00E+00	Fibroblast	Marcks
0.00E+00	1.80	0.787	0.037	0.00E+00	Fibroblast	Cd83
0.00E+00	1.77	0.87	0.021	0.00E+00	Fibroblast	Spi1
0.00E+00	1.74	0.698	0.032	0.00E+00	Fibroblast	Traf1
0.00E+00	1.70	0.667	0.018	0.00E+00	Fibroblast	Ms4a6c
0.00E+00	1.69	0.737	0.042	0.00E+00	Fibroblast	Dusp2
0.00E+00	1.66	0.702	0.026	0.00E+00	Fibroblast	Lst1
0.00E+00	1.63	0.67	0.079	0.00E+00	Fibroblast	Marcksl1
0.00E+00	1.61	0.797	0.086	0.00E+00	Fibroblast	Emp3
0.00E+00	1.61	0.867	0.054	0.00E+00	Fibroblast	Arhgdib
0.00E+00	1.58	0.635	0.076	0.00E+00	Fibroblast	H2-DMa
0.00E+00	1.54	0.749	0.022	0.00E+00	Fibroblast	Mpeg1
0.00E+00	1.52	0.873	0.026	0.00E+00	Fibroblast	Cd53
0.00E+00	1.51	0.657	0.028	0.00E+00	Fibroblast	Stap1
0.00E+00	1.49	0.708	0.032	0.00E+00	Fibroblast	Fyb
0.00E+00	1.49	0.61	0.023	0.00E+00	Fibroblast	Pou2f2
0.00E+00	1.48	0.778	0.02	0.00E+00	Fibroblast	Snx20
0.00E+00	1.47	0.819	0.102	0.00E+00	Fibroblast	Dennd4a
0.00E+00	1.41	0.676	0.011	0.00E+00	Fibroblast	Samsn1
0.00E+00	1.41	0.829	0.095	0.00E+00	Fibroblast	Stk17b
0.00E+00	1.41	0.717	0.045	0.00E+00	Fibroblast	Plbd1
0.00E+00	1.37	0.756	0.114	0.00E+00	Fibroblast	Samhd1
0.00E+00	1.35	0.603	0.017	0.00E+00	Fibroblast	Bcl2a1d
0.00E+00	1.34	0.768	0.096	0.00E+00	Fibroblast	Tgfb1
0.00E+00	1.34	0.787	0.108	0.00E+00	Fibroblast	Psmb8
0.00E+00	1.32	0.638	0.018	0.00E+00	Fibroblast	Cd300a
0.00E+00	1.31	0.825	0.111	0.00E+00	Fibroblast	Gmfg
0.00E+00	1.31	0.784	0.023	0.00E+00	Fibroblast	Selplg
0.00E+00	1.30	0.67	0.019	0.00E+00	Fibroblast	Pid1
0.00E+00	1.27	0.698	0.015	0.00E+00	Fibroblast	Itgb2
0.00E+00	1.27	0.74	0.109	0.00E+00	Fibroblast	Lyn
0.00E+00	1.27	0.813	0.129	0.00E+00	Fibroblast	Fam49b
0.00E+00	1.26	0.746	0.027	0.00E+00	Fibroblast	Rac2
0.00E+00	1.26	0.606	0.026	0.00E+00	Fibroblast	Tlr2
0.00E+00	1.26	0.781	0.021	0.00E+00	Fibroblast	Pld4
0.00E+00	1.24	0.651	0.028	0.00E+00	Fibroblast	Ncf2
0.00E+00	1.15	0.702	0.015	0.00E+00	Fibroblast	Gpr132
0.00E+00	1.15	0.619	0.012	0.00E+00	Fibroblast	Csf2rb
0.00E+00	1.15	0.759	0.103	0.00E+00	Fibroblast	Unc93b1
0.00E+00	1.14	0.644	0.025	0.00E+00	Fibroblast	Tgfbi
0.00E+00	1.11	0.644	0.06	0.00E+00	Fibroblast	Gm8995
0.00E+00	1.10	0.711	0.041	0.00E+00	Fibroblast	Plekho2
0.00E+00	1.08	0.679	0.056	0.00E+00	Fibroblast	Csf2ra
0.00E+00	1.08	0.66	0.017	0.00E+00	Fibroblast	Pirb
0.00E+00	1.04	0.625	0.024	0.00E+00	Fibroblast	Cd300c2
0.00E+00	1.03	0.711	0.039	0.00E+00	Fibroblast	Tm6sf1
0.00E+00	1.01	0.635	0.037	0.00E+00	Fibroblast	Cd68
0.00E+00	1.00	0.654	0.029	0.00E+00	Fibroblast	Hcls1
0.00E+00	0.99	0.648	0.025	0.00E+00	Fibroblast	Nrros
0.00E+00	0.98	0.648	0.05	0.00E+00	Fibroblast	Agpat4
0.00E+00	0.96	0.667	0.074	0.00E+00	Fibroblast	Celf2
0.00E+00	0.96	0.613	0.038	0.00E+00	Fibroblast	Irf5
0.00E+00	0.94	0.629	0.024	0.00E+00	Fibroblast	Cyth4
0.00E+00	0.94	0.603	0.023	0.00E+00	Fibroblast	Ly86
0.00E+00	0.76	0.61	0.065	0.00E+00	Fibroblast	Gpsm3

2.27E-301	1.17	0.689	0.092	7.02E-297	Fibroblast	Flna
1.69E-289	1.33	0.762	0.12	5.23E-285	Fibroblast	Adgre5
1.79E-264	1.04	0.61	0.081	5.55E-260	Fibroblast	Tnfaip3
9.39E-259	1.93	0.86	0.188	2.91E-254	Fibroblast	Lgals3
4.97E-251	1.22	0.752	0.138	1.54E-246	Fibroblast	Ptpn18
6.95E-242	2.16	0.676	0.12	2.15E-237	Fibroblast	Plac8
5.43E-238	1.83	0.956	0.319	1.68E-233	Fibroblast	Arpc1b
2.47E-229	0.82	0.648	0.101	7.65E-225	Fibroblast	Cmtm7
1.82E-224	2.10	0.863	0.234	5.65E-220	Fibroblast	Tmsb10
2.11E-220	1.26	0.794	0.188	6.54E-216	Fibroblast	Efhd2
1.93E-210	2.73	1	0.528	5.99E-206	Fibroblast	Tmsb4x
6.73E-208	2.03	0.775	0.182	2.08E-203	Fibroblast	Crip1
6.80E-193	1.04	0.733	0.16	2.11E-188	Fibroblast	Lrrfip1
3.62E-191	2.00	0.984	0.529	1.12E-186	Fibroblast	Btg1
1.54E-187	1.28	0.765	0.171	4.77E-183	Fibroblast	Nr4a1
6.75E-183	1.60	0.956	0.42	2.09E-178	Fibroblast	Sh3bgrl3
1.35E-182	1.56	0.886	0.325	4.17E-178	Fibroblast	Kdm6b
1.98E-181	0.89	0.603	0.108	6.14E-177	Fibroblast	Trf
8.89E-180	1.38	1	0.985	2.75E-175	Fibroblast	Fau
1.60E-178	1.84	1	0.964	4.96E-174	Fibroblast	Actb
2.51E-177	1.09	0.686	0.145	7.79E-173	Fibroblast	Ctsc
1.76E-171	1.15	0.695	0.168	5.46E-167	Fibroblast	Map4k4
2.12E-171	2.48	0.743	0.198	6.56E-167	Fibroblast	Cd74
5.16E-170	1.40	0.994	0.967	1.60E-165	Fibroblast	Rps9
2.88E-169	1.39	0.924	0.364	8.93E-165	Fibroblast	Iqgap1
1.73E-167	1.66	0.997	0.624	5.36E-163	Fibroblast	H2-D1
1.89E-166	1.52	0.99	0.635	5.87E-162	Fibroblast	Mcl1
3.20E-166	0.88	0.616	0.128	9.92E-162	Fibroblast	Rhog
1.53E-162	1.22	0.797	0.246	4.74E-158	Fibroblast	Litaf
1.01E-158	1.89	0.917	0.417	3.14E-154	Fibroblast	Ifitm2
6.11E-157	1.26	0.997	0.919	1.89E-152	Fibroblast	H3f3a
6.69E-157	2.32	0.984	0.836	2.07E-152	Fibroblast	Cst3
1.24E-156	0.95	0.638	0.152	3.86E-152	Fibroblast	Gpcpd1
1.48E-156	1.02	0.727	0.172	4.59E-152	Fibroblast	Anxa2
9.25E-155	1.13	0.806	0.275	2.86E-150	Fibroblast	Ifngr1
1.08E-154	1.80	0.949	0.521	3.33E-150	Fibroblast	Nfkbia
3.02E-154	1.09	0.676	0.176	9.36E-150	Fibroblast	Nfkb1
1.98E-150	1.23	0.892	0.356	6.13E-146	Fibroblast	Msn
1.71E-148	1.17	0.997	0.976	5.30E-144	Fibroblast	Rps27a
1.19E-144	1.30	0.784	0.236	3.68E-140	Fibroblast	Gadd45b
1.44E-144	1.40	0.978	0.668	4.46E-140	Fibroblast	B2m
8.92E-144	1.16	0.994	0.972	2.76E-139	Fibroblast	Rps16
1.26E-143	1.26	0.997	0.963	3.90E-139	Fibroblast	Actg1
1.53E-139	1.15	0.978	0.828	4.74E-135	Fibroblast	Pfn1
3.42E-139	1.20	0.962	0.757	1.06E-134	Fibroblast	Arpc2
1.40E-134	1.12	0.987	0.944	4.34E-130	Fibroblast	Rpl34
2.28E-130	0.97	1	0.976	7.06E-126	Fibroblast	Rps27
3.63E-129	1.16	0.984	0.965	1.13E-124	Fibroblast	Rpl18a
4.79E-129	1.24	0.603	0.162	1.48E-124	Fibroblast	Malt1
6.28E-129	1.42	0.975	0.975	1.95E-124	Fibroblast	Rps11
2.80E-128	1.21	0.908	0.462	8.68E-124	Fibroblast	Ucp2
5.60E-128	1.32	0.959	0.695	1.73E-123	Fibroblast	H2-K1
2.35E-127	1.62	0.771	0.304	7.27E-123	Fibroblast	Nfkbiz
6.99E-127	1.18	0.968	0.979	2.16E-122	Fibroblast	Rps24
4.13E-126	1.13	0.984	0.946	1.28E-121	Fibroblast	Rpl17
4.14E-125	1.04	0.962	0.637	1.28E-120	Fibroblast	Gnai2
6.03E-125	1.22	0.952	0.902	1.87E-120	Fibroblast	Rpl18
9.24E-125	1.13	0.965	0.947	2.86E-120	Fibroblast	Rps13
1.80E-124	1.31	0.914	0.548	5.57E-120	Fibroblast	Rap1b
2.57E-124	0.96	0.813	0.297	7.96E-120	Fibroblast	Ier5
9.30E-123	1.16	0.994	0.932	2.88E-118	Fibroblast	H3f3b
1.74E-122	1.03	0.987	0.973	5.40E-118	Fibroblast	Rps3a1
3.92E-122	0.99	0.978	0.964	1.21E-117	Fibroblast	Rpl9
1.68E-121	0.99	0.746	0.272	5.19E-117	Fibroblast	Skil

6.50E-121	1.38	0.952	0.951	2.01E-116	Fibroblast	Rpsa
6.66E-121	1.02	0.959	0.798	2.06E-116	Fibroblast	Cfl1
8.52E-121	1.09	0.94	0.716	2.64E-116	Fibroblast	Arpc3
6.62E-120	0.97	0.978	0.976	2.05E-115	Fibroblast	Rps14
4.23E-119	1.07	0.959	0.953	1.31E-114	Fibroblast	Rps15a
3.74E-118	0.88	0.632	0.187	1.16E-113	Fibroblast	Vasp
5.32E-118	1.15	0.978	0.961	1.65E-113	Fibroblast	Rplp0
6.24E-118	1.11	0.971	0.956	1.93E-113	Fibroblast	Rpl13
8.22E-118	1.09	0.975	0.959	2.55E-113	Fibroblast	Rpl10
1.06E-117	0.90	0.648	0.179	3.27E-113	Fibroblast	Trib1
1.60E-117	1.13	0.848	0.482	4.96E-113	Fibroblast	Actr3
3.15E-116	1.00	0.975	0.975	9.77E-112	Fibroblast	Rps12
3.18E-116	1.11	0.711	0.267	9.86E-112	Fibroblast	Ptpn1
4.29E-115	1.17	0.962	0.908	1.33E-110	Fibroblast	Rps7
9.11E-115	0.94	0.752	0.275	2.82E-110	Fibroblast	Tpm4
2.52E-114	0.94	0.962	0.962	7.81E-110	Fibroblast	Rplp2
1.76E-113	0.92	0.971	0.939	5.45E-109	Fibroblast	Rpl30
2.72E-113	1.00	0.978	0.974	8.43E-109	Fibroblast	Rpl19
1.15E-112	1.54	0.714	0.292	3.57E-108	Fibroblast	Zeb2
1.77E-112	0.95	0.987	0.961	5.47E-108	Fibroblast	Rpl8
4.06E-112	1.01	0.854	0.444	1.26E-107	Fibroblast	Tpm3
2.69E-111	1.10	0.965	0.957	8.34E-107	Fibroblast	Rps4x
4.60E-111	1.17	0.905	0.592	1.43E-106	Fibroblast	Nfe2l2
1.10E-109	1.09	0.949	0.964	3.42E-105	Fibroblast	Rps5
8.54E-109	0.86	0.994	0.99	2.65E-104	Fibroblast	Rps29
2.74E-108	0.77	0.625	0.199	8.48E-104	Fibroblast	Grk2
3.47E-108	1.34	0.603	0.167	1.07E-103	Fibroblast	Tnfaip2
8.70E-107	1.08	0.898	0.466	2.69E-102	Fibroblast	Ly6e
3.41E-106	0.93	0.956	0.955	1.06E-101	Fibroblast	Rps23
4.10E-106	1.42	0.87	0.514	1.27E-101	Fibroblast	S100a11
4.83E-106	0.96	0.94	0.735	1.50E-101	Fibroblast	Cdc42
7.76E-105	1.00	0.644	0.21	2.40E-100	Fibroblast	Icam1
1.59E-104	0.97	0.981	0.946	4.91E-100	Fibroblast	Rpl21
1.77E-104	0.97	0.94	0.929	5.49E-100	Fibroblast	Rps19
5.86E-104	0.82	0.981	0.986	1.81E-99	Fibroblast	Rps8
2.94E-102	0.97	0.971	0.958	9.12E-98	Fibroblast	Rps3
1.17E-101	1.00	0.917	0.934	3.63E-97	Fibroblast	Rack1
1.19E-101	1.19	0.879	0.698	3.68E-97	Fibroblast	Sub1
1.48E-101	0.98	0.86	0.536	4.60E-97	Fibroblast	Arpc5
2.17E-101	1.00	0.959	0.947	6.72E-97	Fibroblast	Rps26
4.41E-101	1.00	0.898	0.59	1.37E-96	Fibroblast	Clic1
5.48E-101	1.30	0.702	0.27	1.70E-96	Fibroblast	Ahnak
1.91E-100	0.81	0.956	0.971	5.90E-96	Fibroblast	Rpl35a
2.54E-100	0.81	0.994	0.981	7.86E-96	Fibroblast	Rpl37
5.84E-100	0.86	0.733	0.297	1.81E-95	Fibroblast	Tnfrsf1a
1.36E-99	1.03	0.93	0.859	4.23E-95	Fibroblast	Eef1b2
3.84E-99	0.94	0.975	0.97	1.19E-94	Fibroblast	Rpl39
7.89E-98	0.79	1	0.99	2.44E-93	Fibroblast	Rpl23
1.21E-97	1.40	0.902	0.785	3.73E-93	Fibroblast	H2az1
1.92E-96	0.74	0.99	0.984	5.95E-92	Fibroblast	Rpl37a
1.47E-95	1.02	0.943	0.978	4.54E-91	Fibroblast	Rpl32
1.41E-94	0.99	0.825	0.463	4.38E-90	Fibroblast	Tln1
2.26E-94	0.82	0.952	0.977	7.01E-90	Fibroblast	Rpl26
4.09E-93	0.91	0.937	0.966	1.27E-88	Fibroblast	Rps18
7.20E-93	1.14	0.883	0.62	2.23E-88	Fibroblast	Gm2a
6.29E-92	0.64	1	0.996	1.95E-87	Fibroblast	Tpt1
1.04E-91	0.83	0.962	0.973	3.23E-87	Fibroblast	Rpl11
2.53E-91	0.96	0.717	0.277	7.85E-87	Fibroblast	Cdkn1a
1.50E-90	0.98	0.908	0.743	4.64E-86	Fibroblast	Rbm3
2.17E-90	0.91	0.927	0.858	6.71E-86	Fibroblast	Rpl27a
3.36E-90	0.98	0.876	0.746	1.04E-85	Fibroblast	Eif3f
6.15E-90	0.83	0.721	0.329	1.90E-85	Fibroblast	Grb2
7.27E-90	0.88	0.898	0.772	2.25E-85	Fibroblast	Pabpc1
3.16E-88	0.87	0.933	0.55	9.79E-84	Fibroblast	Junb

4.35E-88	1.08	0.94	0.811	1.35E-83	Fibroblast	Calm1
5.23E-88	0.78	0.956	0.92	1.62E-83	Fibroblast	Rpl29
1.03E-87	0.87	0.946	0.976	3.18E-83	Fibroblast	Rps20
3.08E-87	0.62	1	0.985	9.55E-83	Fibroblast	Eif1
1.24E-86	0.80	0.644	0.203	3.85E-82	Fibroblast	Socs3
2.09E-86	0.87	0.695	0.314	6.47E-82	Fibroblast	Zyx
4.41E-86	0.96	0.889	0.769	1.36E-81	Fibroblast	Rps10
6.55E-86	0.76	0.632	0.238	2.03E-81	Fibroblast	Bcl10
7.73E-86	0.85	0.857	0.491	2.40E-81	Fibroblast	Ccnl1
8.21E-86	0.85	0.622	0.211	2.54E-81	Fibroblast	Kctd12
1.01E-85	0.93	0.851	0.595	3.13E-81	Fibroblast	Capzb
1.67E-85	0.81	0.937	0.48	5.17E-81	Fibroblast	Btg2
1.68E-85	0.61	0.717	0.247	5.20E-81	Fibroblast	Klf2
2.01E-85	0.99	0.937	0.916	6.23E-81	Fibroblast	Rpl3
1.32E-84	0.78	0.952	0.966	4.09E-80	Fibroblast	Rpl6
1.47E-84	0.83	0.654	0.261	4.55E-80	Fibroblast	Jpt1
1.66E-83	0.88	0.794	0.461	5.14E-79	Fibroblast	Pitpna
3.56E-83	0.79	0.911	0.874	1.10E-78	Fibroblast	Rpl4
3.95E-82	0.80	0.99	0.988	1.22E-77	Fibroblast	Rplp1
6.28E-81	0.87	0.841	0.533	1.94E-76	Fibroblast	Cd47
1.63E-80	1.24	0.752	0.418	5.05E-76	Fibroblast	Cdk2ap2
1.82E-80	1.05	0.905	0.897	5.64E-76	Fibroblast	Rpl12
2.55E-78	0.99	0.73	0.354	7.90E-74	Fibroblast	Ifrd1
1.08E-77	1.15	0.841	0.667	3.36E-73	Fibroblast	Npc2
1.26E-76	0.78	0.914	0.902	3.89E-72	Fibroblast	Rpl7
4.54E-76	0.82	0.644	0.277	1.41E-71	Fibroblast	Ikbkb
5.49E-75	0.76	0.943	0.94	1.70E-70	Fibroblast	Rpl22
2.39E-74	0.98	0.721	0.362	7.40E-70	Fibroblast	Dleu2
7.34E-74	0.67	0.946	0.962	2.27E-69	Fibroblast	Rps15
1.06E-73	2.09	0.784	0.575	3.28E-69	Fibroblast	Cebpb
4.66E-73	1.43	0.714	0.427	1.44E-68	Fibroblast	Ifi30
1.73E-72	0.93	0.686	0.323	5.35E-68	Fibroblast	Klf13
1.90E-70	0.79	0.905	0.915	5.88E-66	Fibroblast	Rpl10a
2.09E-70	0.73	0.603	0.252	6.47E-66	Fibroblast	Kdm7a
2.95E-70	0.88	0.857	0.775	9.14E-66	Fibroblast	Rps6
4.99E-70	0.82	0.968	0.852	1.55E-65	Fibroblast	Jund
7.62E-70	0.71	0.689	0.301	2.36E-65	Fibroblast	Tsc22d3
1.57E-69	0.77	0.705	0.355	4.87E-65	Fibroblast	Akap13
4.07E-68	0.81	0.794	0.494	1.26E-63	Fibroblast	Rap1a
7.73E-68	0.70	0.93	0.939	2.39E-63	Fibroblast	Rps28
1.26E-67	0.84	0.844	0.685	3.89E-63	Fibroblast	Sec61b
1.69E-67	0.64	0.978	0.982	5.23E-63	Fibroblast	Rpl28
2.02E-67	0.80	0.721	0.404	6.24E-63	Fibroblast	Tmed5
5.15E-67	0.65	0.946	0.922	1.60E-62	Fibroblast	Rps25
1.71E-66	0.70	0.924	0.924	5.30E-62	Fibroblast	Rpl35
4.38E-66	1.05	0.778	0.539	1.36E-61	Fibroblast	Tpd52
1.53E-65	0.65	0.93	0.838	4.73E-61	Fibroblast	Sem1
2.28E-65	0.85	0.892	0.739	7.07E-61	Fibroblast	Taldo1
9.19E-65	0.58	0.968	0.985	2.85E-60	Fibroblast	Rps21
3.11E-64	0.73	0.825	0.551	9.64E-60	Fibroblast	Cotl1
2.52E-63	0.58	0.975	0.977	7.82E-59	Fibroblast	Rpl38
9.97E-63	1.01	0.768	0.517	3.09E-58	Fibroblast	Atp2b1
1.48E-62	0.71	0.895	0.825	4.58E-58	Fibroblast	Rpl23a
3.70E-62	0.64	0.937	0.819	1.15E-57	Fibroblast	Rhoa
9.93E-61	0.68	0.876	0.808	3.08E-56	Fibroblast	Rpl27
9.99E-61	0.82	0.952	0.922	3.10E-56	Fibroblast	Psap
1.15E-60	0.67	0.613	0.27	3.57E-56	Fibroblast	Clec2d
1.94E-60	0.65	0.654	0.334	6.02E-56	Fibroblast	Skap2
8.66E-60	0.52	0.997	0.997	2.68E-55	Fibroblast	Eef1a1
9.36E-59	0.55	0.946	0.973	2.90E-54	Fibroblast	Rpl36
1.90E-58	0.72	0.819	0.607	5.90E-54	Fibroblast	Capza2
2.96E-58	0.79	0.825	0.589	9.16E-54	Fibroblast	Ctsz
3.63E-58	0.71	0.933	0.947	1.12E-53	Fibroblast	Rpl14
5.69E-58	0.68	0.797	0.432	1.76E-53	Fibroblast	Ier2

8.33E-58	0.61	0.892	0.89	2.58E-53	Fibroblast	Btf3
1.24E-57	0.74	0.822	0.621	3.84E-53	Fibroblast	Picalm
5.06E-57	0.82	0.886	0.615	1.57E-52	Fibroblast	Dusp1
9.95E-57	0.62	0.898	0.816	3.08E-52	Fibroblast	Eif4a1
3.70E-56	0.65	0.851	0.705	1.15E-51	Fibroblast	Arf5
4.06E-56	0.55	0.975	0.905	1.26E-51	Fibroblast	Ddx5
2.09E-55	0.73	0.746	0.441	6.47E-51	Fibroblast	Ankrd11
2.13E-55	0.67	0.629	0.333	6.58E-51	Fibroblast	Kpna4
4.08E-55	0.86	0.752	0.479	1.26E-50	Fibroblast	Prr13
4.45E-55	0.85	0.711	0.446	1.38E-50	Fibroblast	Psme1
2.31E-54	0.62	0.857	0.649	7.15E-50	Fibroblast	Sh3glb1
2.40E-54	0.71	0.829	0.732	7.43E-50	Fibroblast	Rpl15
5.19E-54	0.68	0.883	0.868	1.61E-49	Fibroblast	Rpl5
5.93E-54	0.72	0.781	0.621	1.84E-49	Fibroblast	Nsa2
1.48E-53	0.72	0.717	0.471	4.58E-49	Fibroblast	Mbnl1
4.15E-53	0.71	0.651	0.353	1.29E-48	Fibroblast	Atp1b3
4.31E-53	0.70	0.867	0.588	1.33E-48	Fibroblast	Zfp36
5.19E-53	0.70	0.759	0.532	1.61E-48	Fibroblast	Man2b1
5.33E-52	0.66	0.692	0.38	1.65E-47	Fibroblast	Stat3
6.91E-52	0.64	0.879	0.836	2.14E-47	Fibroblast	Rpl24
3.99E-51	0.56	0.743	0.439	1.23E-46	Fibroblast	Tmem50a
5.50E-51	0.69	0.781	0.622	1.70E-46	Fibroblast	Erp29
7.87E-51	0.64	0.708	0.436	2.44E-46	Fibroblast	Shisa5
9.40E-51	0.78	0.632	0.363	2.91E-46	Fibroblast	Rilpl2
2.11E-50	0.65	0.644	0.378	6.54E-46	Fibroblast	Sec11c
3.38E-50	0.98	0.762	0.556	1.05E-45	Fibroblast	Tagln2
7.56E-50	0.57	0.93	0.935	2.34E-45	Fibroblast	Rpl7a
1.19E-49	0.75	0.73	0.494	3.67E-45	Fibroblast	Mapkapk2
3.19E-49	0.56	0.603	0.305	9.87E-45	Fibroblast	Sh3bgrl
5.91E-49	0.65	0.629	0.35	1.83E-44	Fibroblast	Map2k3
1.17E-48	0.85	0.673	0.438	3.63E-44	Fibroblast	Ehd1
1.70E-48	0.67	0.622	0.334	5.25E-44	Fibroblast	H2-T23
5.53E-48	0.62	0.775	0.534	1.71E-43	Fibroblast	Ostf1
7.86E-48	0.52	0.975	0.98	2.43E-43	Fibroblast	Rps2
1.03E-46	0.51	0.883	0.751	3.21E-42	Fibroblast	Gnb2
7.80E-46	0.66	0.635	0.36	2.42E-41	Fibroblast	Kras
9.28E-46	0.62	0.698	0.44	2.88E-41	Fibroblast	Myl12b
1.15E-45	0.64	0.714	0.496	3.56E-41	Fibroblast	Ccdc12
2.53E-45	0.60	0.8	0.624	7.83E-41	Fibroblast	Srsf3
1.20E-44	0.57	0.819	0.708	3.72E-40	Fibroblast	Ssr4
2.44E-44	0.74	0.67	0.454	7.54E-40	Fibroblast	Syngr2
4.29E-44	0.76	0.756	0.58	1.33E-39	Fibroblast	Ctsh
1.59E-43	0.56	0.616	0.355	4.94E-39	Fibroblast	Arpc4
1.63E-43	0.63	0.644	0.379	5.05E-39	Fibroblast	Trps1
2.31E-43	0.58	0.902	0.769	7.15E-39	Fibroblast	Hspa5
3.52E-43	0.58	0.746	0.565	1.09E-38	Fibroblast	Rpl13a
1.26E-42	0.60	0.784	0.632	3.90E-38	Fibroblast	Serp1
6.74E-42	0.64	0.721	0.464	2.09E-37	Fibroblast	Hmgb2
7.64E-42	0.58	0.73	0.507	2.37E-37	Fibroblast	Chmp4b
1.01E-41	0.58	0.81	0.75	3.13E-37	Fibroblast	Eif3h
2.38E-40	0.54	0.851	0.731	7.36E-36	Fibroblast	Hnrnpf
3.13E-40	0.68	0.711	0.59	9.71E-36	Fibroblast	Macroh2a1
4.44E-40	0.54	0.705	0.43	1.37E-35	Fibroblast	Anxa5
5.36E-40	0.51	0.99	0.832	1.66E-35	Fibroblast	Cyba
6.60E-39	0.51	0.778	0.552	2.04E-34	Fibroblast	Srsf7
1.05E-38	0.60	0.622	0.394	3.25E-34	Fibroblast	Psme2
1.21E-38	0.79	0.794	0.627	3.73E-34	Fibroblast	Mrpl33
4.37E-38	0.53	0.638	0.366	1.35E-33	Fibroblast	Rbms1
1.81E-37	0.56	0.702	0.508	5.62E-33	Fibroblast	Actr2
2.55E-37	0.58	0.663	0.413	7.90E-33	Fibroblast	Myh9
8.19E-37	0.70	0.632	0.406	2.54E-32	Fibroblast	Nadk
8.42E-37	0.51	0.717	0.453	2.61E-32	Fibroblast	Tra2a
1.28E-36	0.78	0.698	0.415	3.98E-32	Fibroblast	Ier3
6.37E-36	0.61	0.724	0.577	1.97E-31	Fibroblast	Spag9

3.06E-35	0.54	0.644	0.429	9.48E-31	Fibroblast	Rnf130
1.28E-33	0.50	0.673	0.45	3.97E-29	Fibroblast	Ptbp3
5.84E-32	0.56	0.686	0.516	1.81E-27	Fibroblast	Sdcbp
6.25E-32	0.61	0.759	0.584	1.94E-27	Fibroblast	Zfand5
1.26E-31	0.53	0.749	0.652	3.89E-27	Fibroblast	Eif3e
7.96E-31	0.55	0.644	0.456	2.47E-26	Fibroblast	Pak2
8.71E-31	0.55	0.698	0.501	2.70E-26	Fibroblast	Ndel1
1.96E-30	0.52	0.689	0.546	6.06E-26	Fibroblast	Snrpg
2.46E-26	0.53	0.61	0.436	7.63E-22	Fibroblast	Mycbp2
4.92E-26	0.61	0.743	0.664	1.53E-21	Fibroblast	410006H16Ri
2.66E-25	0.52	0.749	0.649	8.23E-21	Fibroblast	Top1
6.37E-25	0.56	0.616	0.386	1.97E-20	Fibroblast	Cd9
1.44E-24	0.74	0.635	0.507	4.47E-20	Fibroblast	Zfp36l2
5.38E-23	0.50	0.689	0.602	1.67E-18	Fibroblast	Rpl36al
2.44E-12	0.57	0.848	0.827	7.57E-08	Fibroblast	Sat1
9.20E-12	0.83	0.73	0.731	2.85E-07	Fibroblast	Msrb1
1.32E-11	1.29	0.622	0.589	4.09E-07	Fibroblast	Apoe
8.67E-11	0.69	0.597	0.622	2.69E-06	Fibroblast	Gsr
0.00E+00	2.41	0.934	0.047	0.00E+00	Lymphocyte	Cd52
0.00E+00	1.82	0.78	0.028	0.00E+00	Lymphocyte	Rac2
0.00E+00	1.79	0.788	0.056	0.00E+00	Lymphocyte	Arhgdib
0.00E+00	1.76	0.74	0.045	0.00E+00	Lymphocyte	Ptprc
0.00E+00	1.76	0.846	0.039	0.00E+00	Lymphocyte	Coro1a
0.00E+00	1.67	0.791	0.097	0.00E+00	Lymphocyte	Stk17b
0.00E+00	1.57	0.923	0.131	0.00E+00	Lymphocyte	Srgn
0.00E+00	1.50	0.777	0.04	0.00E+00	Lymphocyte	Laptm5
0.00E+00	1.47	0.667	0.072	0.00E+00	Lymphocyte	Ets1
0.00E+00	1.41	0.626	0.032	0.00E+00	Lymphocyte	Arhgap45
0.00E+00	1.32	0.63	0.03	0.00E+00	Lymphocyte	Cd53
1.80E-288	1.75	0.832	0.138	5.56E-284	Lymphocyte	Ptpn18
8.79E-283	1.73	0.733	0.104	2.72E-278	Lymphocyte	Dennd4a
3.21E-267	2.59	0.967	0.234	9.96E-263	Lymphocyte	Tmsb10
3.98E-265	2.45	0.824	0.155	1.23E-260	Lymphocyte	Vps37b
2.63E-236	1.22	0.634	0.079	8.14E-232	Lymphocyte	Gimap6
2.47E-226	1.30	0.7	0.11	7.65E-222	Lymphocyte	Psmb8
1.09E-222	1.44	0.755	0.138	3.38E-218	Lymphocyte	Cytip
1.72E-203	1.14	0.711	0.111	5.34E-199	Lymphocyte	Fxyd5
2.37E-202	1.42	0.733	0.137	7.35E-198	Lymphocyte	H2-Q7
1.11E-151	2.01	1	0.529	3.44E-147	Lymphocyte	Tmsb4x
1.28E-150	1.68	1	0.979	3.98E-146	Lymphocyte	Rps24
2.01E-143	1.44	0.996	0.972	6.23E-139	Lymphocyte	Rps16
2.19E-143	1.59	0.996	0.953	6.78E-139	Lymphocyte	Rps15a
3.20E-143	1.61	1	0.95	9.91E-139	Lymphocyte	Rpsa
1.94E-142	1.46	0.996	0.946	6.02E-138	Lymphocyte	Rps13
6.84E-142	1.46	1	0.965	2.12E-137	Lymphocyte	Rpl18a
3.39E-138	1.39	1	0.956	1.05E-133	Lymphocyte	Rpl13
8.28E-138	1.79	0.989	0.896	2.57E-133	Lymphocyte	Rpl12
2.23E-137	1.54	1	0.976	6.92E-133	Lymphocyte	Rps27
3.02E-137	1.32	1	0.973	9.35E-133	Lymphocyte	Rpl19
1.15E-135	1.44	0.996	0.907	3.56E-131	Lymphocyte	Rps7
1.67E-135	1.31	0.993	0.974	5.18E-131	Lymphocyte	Rps11
1.91E-135	1.20	0.996	0.978	5.91E-131	Lymphocyte	Rpl32
2.55E-135	1.29	0.993	0.944	7.90E-131	Lymphocyte	Rpl34
2.91E-134	1.37	0.989	0.902	9.03E-130	Lymphocyte	Rpl18
1.23E-133	1.40	0.996	0.946	3.80E-129	Lymphocyte	Rpl17
8.90E-133	1.27	0.996	0.957	2.76E-128	Lymphocyte	Rps3
1.13E-132	1.76	0.982	0.695	3.51E-128	Lymphocyte	H2-K1
2.17E-132	1.31	0.996	0.985	6.72E-128	Lymphocyte	Fau
1.03E-131	1.28	1	0.973	3.19E-127	Lymphocyte	Rps3a1
1.46E-131	1.20	0.996	0.976	4.52E-127	Lymphocyte	Rps14
1.91E-131	1.29	1	0.956	5.93E-127	Lymphocyte	Rps4x
1.57E-130	1.19	1	0.966	4.86E-126	Lymphocyte	Rps18
8.16E-129	1.16	0.711	0.184	2.53E-124	Lymphocyte	Crip1
4.31E-127	1.24	0.993	0.961	1.34E-122	Lymphocyte	Rplp2

1.39E-126	1.26	0.996	0.961	4.32E-122	Lymphocyte	Rpl8
2.53E-126	1.34	0.996	0.946	7.85E-122	Lymphocyte	Rpl21
4.53E-124	1.29	1	0.964	1.40E-119	Lymphocyte	Rps5
6.89E-124	1.21	0.993	0.964	2.13E-119	Lymphocyte	Rpl9
7.65E-124	1.30	1	0.975	2.37E-119	Lymphocyte	Rps20
1.50E-123	1.46	0.978	0.624	4.63E-119	Lymphocyte	H2-D1
1.22E-122	1.10	0.996	0.988	3.78E-118	Lymphocyte	Rplp1
3.09E-118	1.12	0.989	0.955	9.57E-114	Lymphocyte	Rps23
1.90E-117	1.21	1	0.967	5.89E-113	Lymphocyte	Rps9
1.57E-116	1.50	0.853	0.435	4.85E-112	Lymphocyte	Shisa5
8.16E-115	0.99	0.996	0.976	2.53E-110	Lymphocyte	Rps27a
4.00E-111	1.16	0.989	0.939	1.24E-106	Lymphocyte	Rpl30
2.51E-110	1.18	1	0.97	7.79E-106	Lymphocyte	Rpl39
2.93E-110	0.99	0.996	0.99	9.08E-106	Lymphocyte	Rpl23
4.85E-110	0.96	1	0.973	1.50E-105	Lymphocyte	Rpl11
5.59E-109	1.12	0.993	0.934	1.73E-104	Lymphocyte	Rack1
6.42E-108	1.26	0.956	0.668	1.99E-103	Lymphocyte	B2m
1.35E-106	1.06	0.996	0.975	4.17E-102	Lymphocyte	Rps12
1.57E-106	1.01	0.993	0.984	4.87E-102	Lymphocyte	Rpl37a
1.70E-106	0.96	0.996	0.986	5.27E-102	Lymphocyte	Rps8
4.48E-106	1.09	0.996	0.961	1.39E-101	Lymphocyte	Rplp0
4.89E-106	1.14	0.996	0.915	1.51E-101	Lymphocyte	Rpl3
5.09E-106	0.80	1	0.996	1.58E-101	Lymphocyte	Tpt1
1.02E-105	1.27	0.894	0.422	3.15E-101	Lymphocyte	Sh3bgrl3
1.35E-104	0.96	1	0.946	4.19E-100	Lymphocyte	Rpl14
1.78E-102	1.09	0.989	0.946	5.53E-98	Lymphocyte	Rps26
1.69E-101	0.85	1	0.971	5.24E-97	Lymphocyte	Rpl35a
2.26E-101	0.97	1	0.985	7.01E-97	Lymphocyte	Rps21
1.34E-99	1.08	0.967	0.857	4.14E-95	Lymphocyte	Rpl27a
5.07E-98	1.45	0.89	0.53	1.57E-93	Lymphocyte	Btg1
7.35E-97	0.90	0.996	0.966	2.28E-92	Lymphocyte	Rpl6
7.23E-96	0.94	1	0.99	2.24E-91	Lymphocyte	Rps29
9.81E-96	0.88	0.996	0.977	3.04E-91	Lymphocyte	Rpl38
1.26E-95	1.06	0.96	0.867	3.90E-91	Lymphocyte	Rpl5
7.53E-95	1.21	0.974	0.929	2.33E-90	Lymphocyte	Rps19
1.77E-94	0.88	0.993	0.972	5.47E-90	Lymphocyte	Rpl36
5.50E-93	0.85	0.985	0.977	1.70E-88	Lymphocyte	Rpl26
6.45E-93	0.92	1	0.961	2.00E-88	Lymphocyte	Rps15
3.19E-92	1.04	0.982	0.901	9.88E-88	Lymphocyte	Rpl7
4.01E-92	0.96	0.982	0.92	1.24E-87	Lymphocyte	Rpl29
9.02E-92	0.97	0.996	0.932	2.80E-87	Lymphocyte	H3f3b
3.30E-89	1.10	1	0.964	1.02E-84	Lymphocyte	Actb
5.99E-88	1.74	0.674	0.277	1.85E-83	Lymphocyte	Ifngr1
1.20E-87	1.20	0.916	0.769	3.72E-83	Lymphocyte	Rps10
4.92E-86	0.87	0.985	0.939	1.52E-81	Lymphocyte	Rpl22
3.49E-84	0.83	0.996	0.981	1.08E-79	Lymphocyte	Rpl37
3.72E-83	0.67	1	0.997	1.15E-78	Lymphocyte	Eef1a1
2.05E-82	0.82	0.993	0.959	6.34E-78	Lymphocyte	Rpl10
3.75E-82	0.97	0.952	0.859	1.16E-77	Lymphocyte	Eef1b2
2.34E-81	1.21	0.861	0.468	7.26E-77	Lymphocyte	Ly6e
1.97E-80	1.03	0.747	0.322	6.12E-76	Lymphocyte	Arpc1b
3.51E-80	1.29	0.875	0.699	1.09E-75	Lymphocyte	Sub1
1.94E-79	0.92	0.967	0.914	5.99E-75	Lymphocyte	Rpl10a
2.46E-78	0.90	0.967	0.918	7.62E-74	Lymphocyte	Rpl36a
3.62E-78	1.07	0.919	0.829	1.12E-73	Lymphocyte	Pfn1
1.42E-77	1.15	0.703	0.301	4.39E-73	Lymphocyte	Tsc22d3
4.12E-76	1.01	0.908	0.808	1.28E-71	Lymphocyte	Rpl27
7.81E-75	1.01	0.89	0.775	2.42E-70	Lymphocyte	Rps6
6.43E-74	0.68	0.996	0.985	1.99E-69	Lymphocyte	Eif1
4.97E-71	0.88	0.93	0.873	1.54E-66	Lymphocyte	Rpl4
7.87E-71	0.88	0.945	0.923	2.44E-66	Lymphocyte	Rpl35
2.65E-67	0.90	0.908	0.799	8.22E-63	Lymphocyte	Cfl1
3.00E-66	0.98	0.875	0.746	9.30E-62	Lymphocyte	Eif3f
1.41E-62	0.98	0.883	0.722	4.37E-58	Lymphocyte	Pnrc1

1.75E-62	1.08	0.747	0.471	5.42E-58	Lymphocyte	Mbnl1
3.56E-62	0.88	0.908	0.825	1.10E-57	Lymphocyte	Rpl23a
4.27E-62	0.73	0.967	0.921	1.32E-57	Lymphocyte	Rps25
1.54E-57	0.62	1	0.98	4.76E-53	Lymphocyte	Rps2
1.72E-57	0.72	0.982	0.938	5.33E-53	Lymphocyte	Rps28
9.96E-56	0.92	0.868	0.482	3.09E-51	Lymphocyte	Btg2
7.52E-55	0.76	0.883	0.773	2.33E-50	Lymphocyte	Pabpc1
1.06E-54	0.57	1	0.982	3.29E-50	Lymphocyte	Rpl28
4.68E-53	1.00	0.945	0.852	1.45E-48	Lymphocyte	Jund
1.34E-52	0.96	0.758	0.565	4.16E-48	Lymphocyte	Rpl13a
9.62E-52	0.83	0.945	0.92	2.98E-47	Lymphocyte	H3f3a
2.42E-46	0.60	0.934	0.889	7.50E-42	Lymphocyte	Btf3
7.26E-45	1.08	0.817	0.552	2.25E-40	Lymphocyte	Junb
9.69E-45	0.69	0.883	0.804	3.00E-40	Lymphocyte	Rbm39
2.65E-44	0.74	0.828	0.732	8.21E-40	Lymphocyte	Rpl15
9.14E-44	0.55	0.945	0.935	2.83E-39	Lymphocyte	Rpl7a
1.33E-43	0.64	0.886	0.836	4.12E-39	Lymphocyte	Rpl24
2.84E-43	0.79	0.81	0.744	8.79E-39	Lymphocyte	Rbm3
8.64E-42	1.15	0.692	0.506	2.68E-37	Lymphocyte	Zfp36l2
9.95E-42	0.81	0.634	0.36	3.08E-37	Lymphocyte	Msn
1.38E-41	1.09	0.674	0.465	4.28E-37	Lymphocyte	Hmgb2
4.78E-41	0.94	0.608	0.357	1.48E-36	Lymphocyte	Akap13
1.17E-40	0.82	0.736	0.552	3.61E-36	Lymphocyte	Srsf7
2.14E-40	0.58	0.938	0.909	6.64E-36	Lymphocyte	Pfdn5
3.82E-40	0.98	0.652	0.443	1.18E-35	Lymphocyte	Ankrd11
9.26E-40	0.82	0.791	0.74	2.87E-35	Lymphocyte	Uba52
9.76E-40	0.81	0.674	0.446	3.02E-35	Lymphocyte	Tpm3
2.01E-38	0.87	0.689	0.501	6.22E-34	Lymphocyte	Jak1
3.25E-38	0.91	0.689	0.434	1.01E-33	Lymphocyte	Ier2
8.37E-38	0.81	0.744	0.589	2.59E-33	Lymphocyte	Clk1
1.03E-37	0.84	0.791	0.718	3.19E-33	Lymphocyte	Arpc3
3.45E-37	0.61	0.883	0.812	1.07E-32	Lymphocyte	Calm1
6.28E-37	0.90	0.645	0.447	1.94E-32	Lymphocyte	Fam107b
1.84E-36	0.80	0.711	0.55	5.71E-32	Lymphocyte	Rap1b
6.89E-36	0.60	0.897	0.853	2.14E-31	Lymphocyte	Npm1
1.75E-35	0.58	0.982	0.97	5.41E-31	Lymphocyte	Ptma
3.28E-35	0.76	0.667	0.454	1.02E-30	Lymphocyte	Tra2a
4.44E-34	0.58	0.93	0.906	1.37E-29	Lymphocyte	Ddx5
5.34E-33	0.82	0.678	0.546	1.66E-28	Lymphocyte	Snrpg
7.32E-33	0.66	0.777	0.751	2.27E-28	Lymphocyte	Eif3h
1.54E-30	0.73	0.729	0.592	4.77E-26	Lymphocyte	Clic1
2.54E-30	0.58	0.817	0.759	7.86E-26	Lymphocyte	Arpc2
7.24E-29	0.62	0.733	0.63	2.24E-24	Lymphocyte	Hnrnpa0
2.21E-27	0.53	0.747	0.64	6.83E-23	Lymphocyte	Gnai2
5.62E-27	0.70	0.813	0.76	1.74E-22	Lymphocyte	S100a10
8.55E-27	0.78	0.604	0.448	2.65E-22	Lymphocyte	Psme1
1.07E-26	0.61	0.766	0.737	3.32E-22	Lymphocyte	Cdc42
1.09E-26	0.58	0.821	0.807	3.37E-22	Lymphocyte	Rpl22l1
1.28E-25	0.57	0.813	0.825	3.97E-21	Lymphocyte	Rpl31
6.53E-25	0.67	0.63	0.505	2.02E-20	Lymphocyte	Nop53
9.27E-25	0.68	0.659	0.536	2.87E-20	Lymphocyte	Cd47
4.75E-24	0.60	0.703	0.687	1.47E-19	Lymphocyte	Sec61b
4.06E-23	0.54	0.63	0.457	1.26E-18	Lymphocyte	Selenow
6.47E-23	0.75	0.791	0.786	2.01E-18	Lymphocyte	H2az1
4.44E-22	0.72	0.663	0.601	1.37E-17	Lymphocyte	Dnajb6
2.47E-21	0.51	0.795	0.787	7.65E-17	Lymphocyte	Dad1
1.06E-18	0.55	0.619	0.539	3.30E-14	Lymphocyte	Arpc5
2.50E-18	0.53	0.736	0.751	7.75E-14	Lymphocyte	Sumo2
5.13E-17	0.53	0.736	0.762	1.59E-12	Lymphocyte	Cnbp
5.34E-17	0.59	0.674	0.652	1.65E-12	Lymphocyte	Sfpq
2.05E-16	0.52	0.674	0.638	6.35E-12	Lymphocyte	Sf3b1
8.67E-16	0.69	0.714	0.752	2.68E-11	Lymphocyte	Dnaja1
1.69E-15	0.63	0.619	0.584	5.22E-11	Lymphocyte	Prrc2c
2.53E-15	0.56	0.7	0.732	7.84E-11	Lymphocyte	Hnrnpf

6.51E-15	0.56	0.659	0.688	2.02E-10	Lymphocyte	Eef1g
8.30E-15	0.69	0.623	0.634	2.57E-10	Lymphocyte	Serp1
2.91E-14	0.67	0.615	0.558	9.01E-10	Lymphocyte	Tagln2
3.57E-14	0.54	0.659	0.626	1.11E-09	Lymphocyte	Srsf3

Supplemental Table 2. KEGG pathway analysis for podocyte clusters

Cluster Podocyte-1 ANALYSIS
Significant Pathways (73)

Link to KEGG Pathway	Pathway Description (KEGG)	Number Genes in Pathway	Number Regulated Genes	P-value	FDR
mmu04514	Cell adhesion molecules (CAMs)	169	9	2.42E-05	1.36E-03
mmu04010	MAPK signaling pathway	294	11	7.93E-05	3.20E-03
mmu04350	TGF-beta signaling pathway	84	5	1.00E-03	2.28E-02
mmu04014	Ras signaling pathway	233	8	1.32E-03	2.76E-02
mmu04550	Signaling pathways regulating pluripotency of stem cells	137	6	1.57E-03	3.17E-02
mmu04151	PI3K-Akt signaling pathway	358	10	1.62E-03	3.25E-02

Cluster Podocyte-2 ANALYSIS
Significant Pathways (37)

Link to KEGG Pathway	Pathway Description (KEGG)	Number Genes in Pathway	Number Regulated Genes	P-value	FDR
mmu04512	ECM-receptor interaction	83	7	6.37E-07	3.36E-04
mmu04510	Focal adhesion	199	8	2.57E-05	4.51E-03
mmu04810	Regulation of actin cytoskeleton	215	7	3.08E-04	2.26E-02
mmu04530	Tight junction	167	6	4.99E-04	3.10E-02
mmu04514	Cell adhesion molecules (CAMs)	169	6	5.32E-04	3.23E-02
mmu04974	Protein digestion and absorption	90	4	2.08E-03	8.27E-02

Cluster Podocyte-3 ANALYSIS
Significant Pathways (60)

Link to KEGG Pathway	Pathway Description (KEGG)	Number Genes in Pathway	Number Regulated Genes	P-value	FDR
mmu05418	Fluid shear stress and atherosclerosis	143	8	1.98E-05	3.13E-03
mmu04668	TNF signaling pathway	110	7	2.91E-05	4.09E-03
mmu04514	Cell adhesion molecules (CAMs)	169	7	4.26E-04	2.64E-02
mmu04010	MAPK signaling pathway	294	9	6.10E-04	3.20E-02
mmu04657	IL-17 signaling pathway	91	5	8.20E-04	3.81E-02
mmu04371	Apelin signaling pathway	139	6	8.89E-04	3.98E-02

Cluster Podocyte-4 ANALYSIS
Significant Pathways (50)

Link to KEGG Pathway	Pathway Description (KEGG)	Number Genes in Pathway	Number Regulated Genes	P-value	FDR
mmu04022	cGMP-PKG signaling pathway	168	11	2.18E-09	7.65E-07
mmu04024	cAMP signaling pathway	198	11	1.22E-08	3.69E-06
mmu04510	Focal adhesion	199	11	1.29E-08	3.69E-06
mmu04371	Apelin signaling pathway	139	9	7.48E-08	1.97E-05
mmu04810	Regulation of actin cytoskeleton	215	10	3.07E-07	6.93E-05
mmu04530	Tight junction	167	6	3.28E-04	2.47E-02

Cluster Podocyte-5 ANALYSIS
Significant Pathways (26)

Link to KEGG Pathway	Pathway Description (KEGG)	Number Genes in Pathway	Number Regulated Genes	P-value	FDR
mmu04512	ECM-receptor interaction	83	4	3.95E-05	1.54E-02
mmu04510	Focal adhesion	199	4	1.12E-03	1.38E-01
mmu04657	IL-17 signaling pathway	91	3	1.21E-03	1.38E-01
mmu04350	TGF-beta signaling pathway	84	2	1.61E-02	6.89E-01
mmu04974	Protein digestion and absorption	90	2	1.84E-02	7.08E-01
mmu04620	Toll-like receptor signaling pathway	99	2	2.20E-02	7.53E-01

Cluster Podocyte-6 ANALYSIS
No significant Pathways
