# Supplementary material for: PIK3CA inhibition in models of proliferative glomerulonephritis and lupus nephritis
Source: J Clin Invest. 2024 Jun 6;134(15):e176402. doi: 10.1172/JCI176402 (PMC11290976; doi:10.1172/JCI176402)

Full unedited gel for Figure 1O

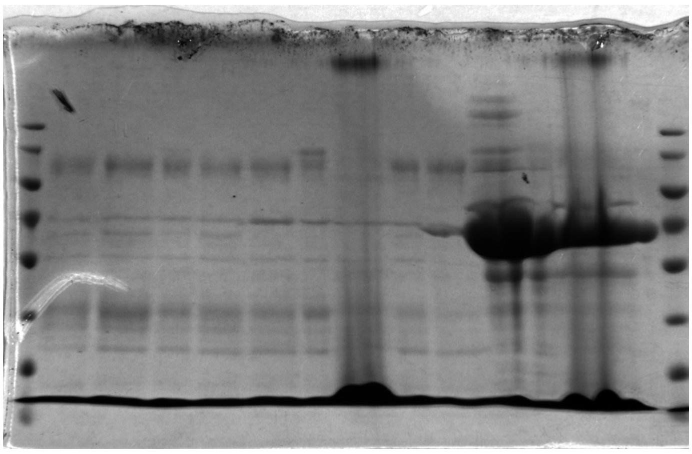

Full unedited blot for Figure 9K

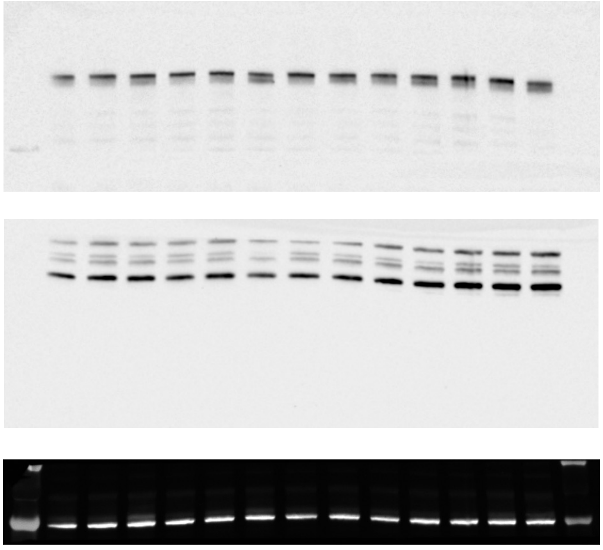

Nephrin

Podocin

α-Tubulin

Full unedited blot for Figure 10S

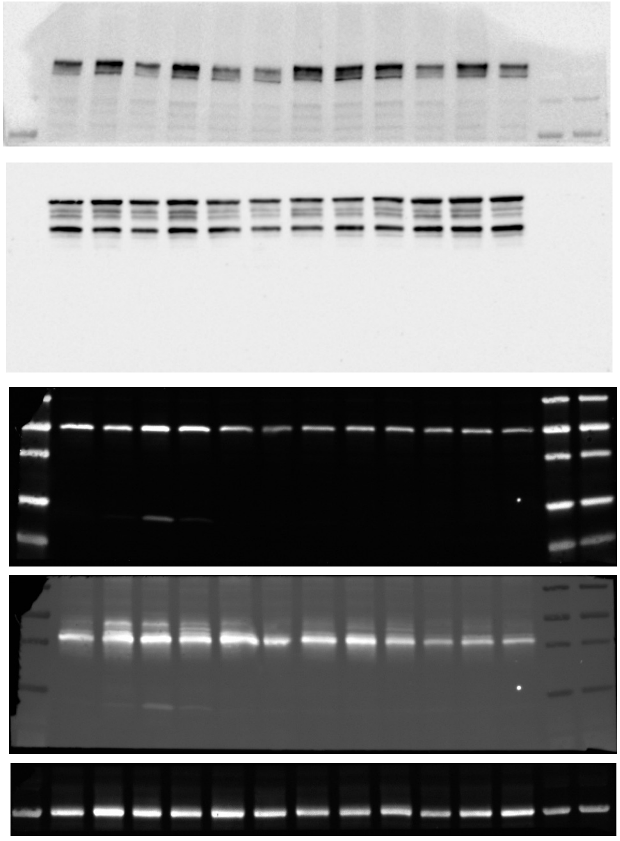

Nephrin

Podocin

P-S6RP

S6RP

α-tubulin

Full unedited gel for Supplemental Figure 1F

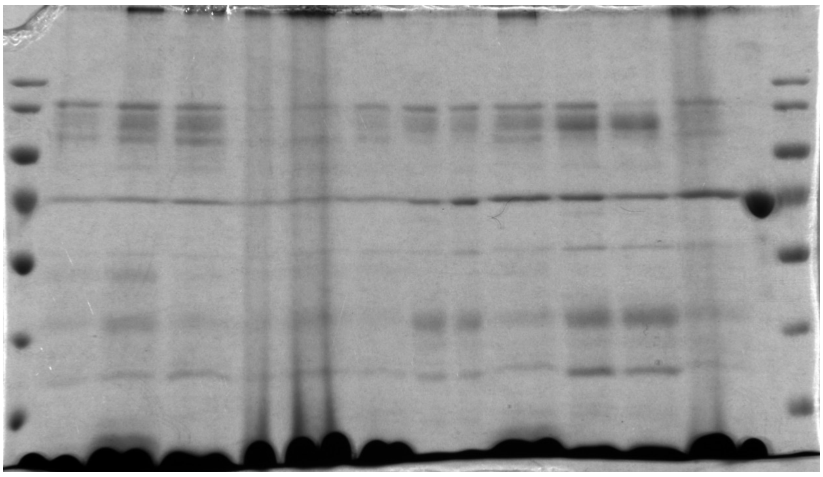

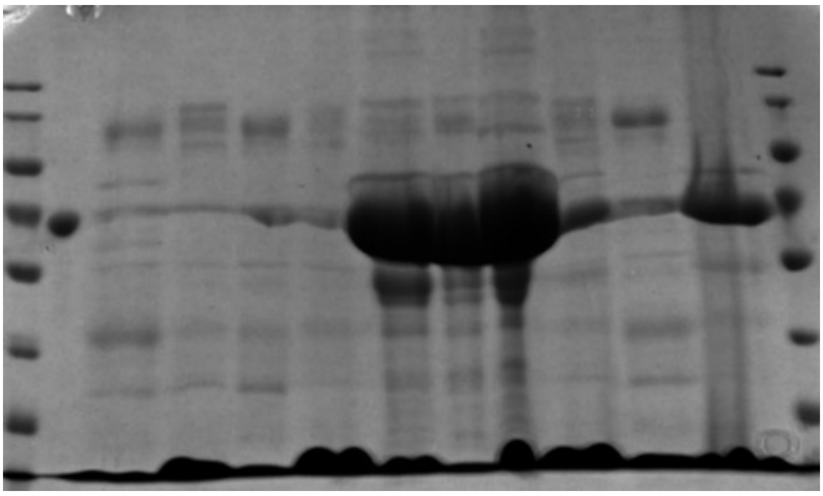

Full unedited gel for Supplemental Figure 5C

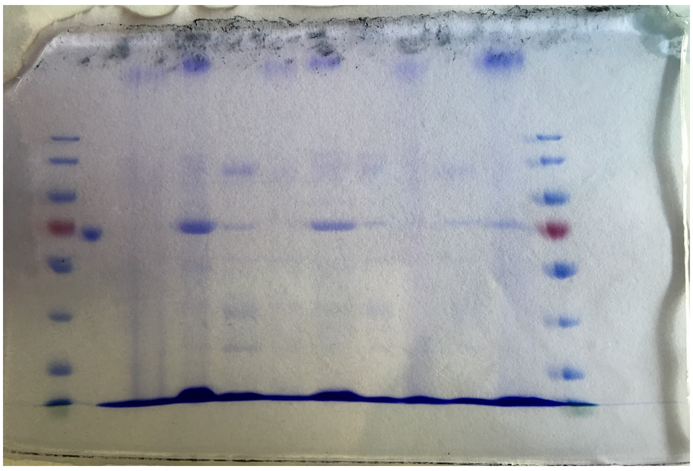

Full unedited blot for Supplemental Figure 9F

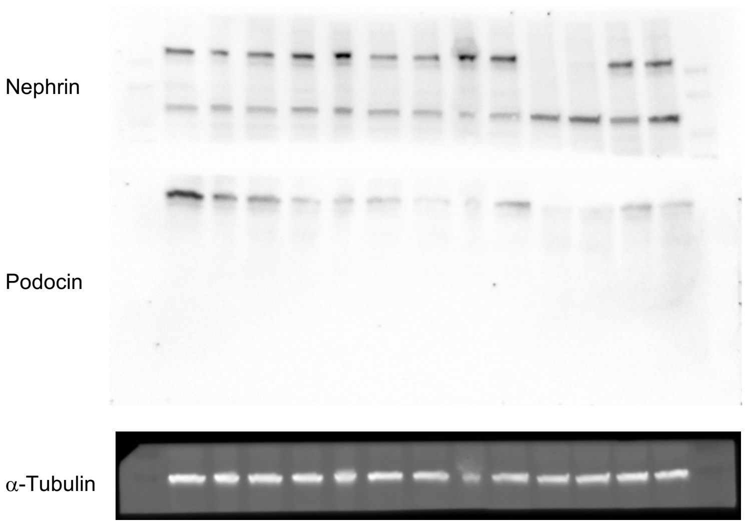

Supplement: Unedited blot and gel images [file jci-134-176402-s110.pdf]
